# Supplementary material for: Neoadjuvant T-DM1/pertuzumab and paclitaxel/trastuzumab/pertuzumab for HER2+ breast cancer in the adaptively randomized I-SPY2 trial
Source: Nat Commun. 2021 Nov 5;12:6428. doi: 10.1038/s41467-021-26019-y (PMC8571284; doi:10.1038/s41467-021-26019-y)
Supplement: Supplementary file 1 — Supplementary Information [file 41467_2021_26019_MOESM1_ESM.pdf]

SUPPLEMENTARY MATERIALS

Clark et al.

Neoadjuvant T-DM1/pertuzumab and paclitaxel/trastuzumab/pertuzumab in HER2-positive breast cancer in the adaptively randomized I-SPY2 trial

1. Supplementary Figures & Tables

Supplementary Table 1: Final predictive probabilities of pCR rate for TDM1/P and THP vs TH. Prob(>Ctl) is the probability that the combination is more effective than the control; Prob(Ph3) is the probability that the treatment would be successful in a hypothetical 300-patient randomized controlled Phase III study specific to the relevant breast cancer subtype.

	TH Control Arm	T-DM1/P Arm			THP Arm		
	**pCR rate** % (95% PI)	**pCR rate** % (95% PI)	**Prob(>Ctl)** %	**Prob(Ph3) %**	**pCR rate** % (95% PI)	**Prob(>Ctl)** %	**Prob(Ph3) %**
**HER2+**	25 (11-38)	55 (41-69)	99.9	96	56 (42-70)	99.9	97
**HR+/HER2+**	20 (7-33)	50 (33-66)	99.7	95	47 (30-64)	99.5	93.1
**HR-/HER2+**	33 (15-52)	63 (43-84)	98.8	91	72 (53-90)	99.9	97.8

Supplementary Table 2: Grade 1/2 Adverse events occuring during TDM1/P, THP or TH.

	T-DM1/P (n = 52)		THP (n=45)		TH CONTROL (n = 31)	
	T-DM1/P (n = 52)	AC (n = 49)	THP (n=45)	AC (n=40)	Paclitaxel (n = 31)	AC (n = 28)
Abdominal Pain	5(9.6%)	5 (10.2%)	2 (4.4%)	0 (0.0%)	4 (12.9%)	0 (0.0%)
Alanine aminotransferase incr.	16 (30.8%)	2 (4.08%)	2 (4.4%)	1 (2.5%)	3 (9.7%)	0 (0.0%)
Alopecia	5 (9.6%)	11 (22.5%)	30 (66.7%)	3 (7.5%)	21 (67.7%)	3 (10.7%)
Anaemia	3 (5.8%)	10 (20.4%)	10 (22.2%)	4 (10.0%)	5 (12.5%)	5 (17.9%)
Anorexia	9 (17.35)	3 (6.15)	6 (13.3%)	4 (10.0%)	2 (6.5%)	3 (10.7%)
Anxiety	4 (7.7%)	2 (4.1%)	4 (8.9%)	2 (5.0%)	3 (9.7%)	7 (25.0%)
Arthralgia	4 (7.7%)	5 (10.2%)	1 (2.2%)	0 (0.0%)	4 (12.9%)	3 (10.7%)
Aspartate aminotransferase incr.	17 (32.3%)	3 (6.1%)	2 (4.4%)	1 (2.5%)	3 (9.7%	0 (0.0%)
Back pain	2 (3.9%)	1 (2.0%)	1 (2.2%)	2 (5.0%)	5 (16.15)	2 (7.1%)
Bone pain	2 (3.9%)	4 (8.2%)	1 (2.2%)	2 (5.0%)	3 (9.7%)	7 (25.0%)
Breast Pain	3 (5.8%)	1 (2.0%)	1 (2.2%)	0 (0.0%)	4 (12.9%)	1 (3.6%)
Chills	9 (17.35)	2 (4.15)	3 (6.7%)	3 (7.5%)	1 (3.25)	0 (0.0%)
Constipation	8 (15.4%)	13 (26.5%)	6 (13.3%)	7 (17.5%)	9 (29.0%)	8 (28.6%)
Cough	7 (13.5%)	6 (12.25)	6 (13.3%)	5 (12.5%)	6 (19.4%)	2 (7.1%)
Dermatitis acneiform	10 (19.2%)	3 (6.1%)	14 (31.1%)	2 (5.0%)	7 (22.3%)	0 (0.0%)
Depression	3 (5.8%)	1 (2.0%)	2 (4.4%)	0 (0.0%)	4 (12.9%)	3 (10.7%)
Diarrhea	26 (50.0%)	15 (30.6%)	35 (77.8%)	8 (20.0%)	15 (48.4%)	6 (21.4%)
Dizziness	2 (3.9%)	2 (4.1%)	4 (8.9%)	4 (10.0%)	2 (6.5%)	3 (10.7%)
Dry Eye	5 (9.6%)	5 (10.2%)	1 (2.2%)	3 (7.5%)	3 (9.7%)	1 (3.4%)
Dry Skin	2 (3.9%)	0 (0.0%)	5 (11.1%)	0 (0.0%)	5 (16.1%)	1 (3.6%)
Dysgeusia	6 (11.5%)	6 (12.2%)	7 (15.65)	2 (5.0%)	4 (12.9%)	2 (7.1%)
Dyspepsia	5 (9.6%)	2 (4.1%)	6 (13.3%)	2 (5.0%)	6 (19.3%)	1 (3.6%)
Dyspnoea	6 (11.5%)	5 (10.2%)	6 (13.3%)	5 (12.5%)	8 (25.8%)	3 (10.7%)
Epistaxis	10 (19.2%)	2 (4.1%)	18 (40.0%)	0 (0.0%)	11 (35.5%)	1 (3.4%)
Fatigue	34 (65.4%)	20 (40.8%)	29 (64.4%)	11 (27.5%)	20 (64.5%)	18 (64.3%)
Folliculitis	0 (0.0%)	1 (2.0%)	1 (2.2%)	0 (0.0%)	5 (16.1%)	0 (0.0%)
Gastroesophageal reflux	7 (13.5%)	4 (8.2%)	3 (6.7%)	3 (7.5%)	4 (12.9%)	3 (10.7%)
Headache	22 (42.3%)	5 (10.2%)	14 (31.1%)	5 (12.5%)	12 (38.7%)	9 (32.1%)
Haemorrhoids	0 (0.0%)	1 (2.0%)	3 (6.7%)	2 (5.0%)	0 (0.0%)	3 (10.7%)
Hot Flush	3 (5.8%)	1 (2.0%)	10 (22.2%)	3 (7.5%)	6 (19.4%)	7 (25.0%)
Hypertension	7 (13.5%)	5 (10.2%)	6 (13.3%)	2 (5.0%)	11 (35.5%)	6 (21.4%)
Hypokalaemia	4 (7.7%)	5 (10.2%)	4 (8.9%)	5 (12.5%)	0 (0.0%)	1 (3.4%)
Infusion related reaction	6 (11.5%)	0 (0.0%)	2 (4.4%)	0 (0.0%)	4 (12.9%)	0 (0.0%)
Insomnia	10 (19.2%)	7 (14.3%)	6 (13.3%)	5 (12.5%)	8 (25.8%)	4 (14.3%)
Localized oedema	2 (3.9%)	2 (4.1%)	5 (11.1%)	0 (0.0%)	1 (3.25)	0 (0.0%)
Myalgia	9 (17.3%)	2 (4.1%)	5 (11.1%)	2 (5.0%)	8 (25.8%)	2 (7.1%)
Nail discolouration	0 (0.0%)	4 (8.2%)	4 (8.9%)	7 (17.5%)	3 (9.7%)	2 (7.1%)
Nail disorder	0 (0.0%)	0 (0.0%)	1 (2.25)	1 (2.55)	0 (0.0%)	4 (14.3%)
Nail infection	1 (1.9%)	1 (2.0%)	0 (0.0%)	0 (0.0%)	0 (0.0%)	4 (14.3%)
Nasal congestion	3 (5.8%)	2 (4.1%)	8 (17.8%)	2 (5.0%)	1 (3.2%)	0 (0.0%)
Nasal dryness	0 (0.0%)	0 (0.0%)	1 (2.2%)	0 (0.0%)	4 (12.9%)	0 (0.0%)

Nausea	29 (55.8%)	22 (44.9%)	27 (60%)	16 (40%)	12 (38.7%)	17 (60.7%)
Oedema peripheral	0 (0.0%)	2 (4.1%)	4 (8.9%)	3 (7.5%)	5 (16.1%)	3 (10.7%)
Onychalgia	0 (0.0%)	1 (2.0%)	1 (2.2%)	4 (10.0%)	1 (3.2%)	1 (3.45)
Onychomadesis	0 (0.0%)	0 (0.0%)	1 (2.25)	3 (7.5%)	1 (3.2%)	3 (10.7%)
Oropharyngeal pain	2 (3.9%)	2 (4.1%)	8 (17.8%)	4 (10.0%)	2 (6.5%)	2 (7.1%)
Pain	4 (7.75)	3 (6.1%)	4 (8.9%)	5 (12.5%)	3 (9.7%)	2 (7.15)
Pain in extremity	1 (1.9%)	1 (2.0%)	4 (8.9%)	1 (2.5%)	6 (19.4%)	1 (3.6%)
Palpitations	4 (7.7%)	0 (0.0%)	2 (4.45)	1 (2.5%)	4 (12.9%)	2 (7.1%)
Paraesthesia	3 (5.8%)	2 (4.1%)	5 (11.1%)	0 (0.0%)	2 (6.5%)	0 (0.0%)
Peripheral sensory neuropathy	9 (17.3%)	9 (18.4%)	21 (46.7%)	5 (12.5%)	15 (48.4%)	2 (7.1%)
Platelet count decrease	1 (1.9%)	6 (12.2%)	1 (2.2%)	0 (0.0%)	0 (0.0%)	1 (3.6%)
Pruritis	3 (5.8%)	1 (2.0%)	4 (8.9%)	0 (0.0%)	5 (16.1%)	1 (3.6%)
Pyrexia	8 (15.4%)	6 (12.25)	4 (8.9%)	2 (5.0%)	4 (12.9%)	0 (0.0%)
Rash maculo-papular	8 (15.4%)	3 (6.1%)	15 (33.3%)	2 (5.0%)	8 (25.8%)	4 (14.3%)
Rhinorrhoea	3 (5.8%)	3 (6.1%)	1 (2.2%)	4 (10.0%)	4 (12.9%)	2 (7.1%)
Skin Hyperpigmentation	0 (0.0%)	8 (16.3%)	2 (4.4%)	4 (10.0%)	0 (0.0%)	3 (10.7%)
Stomatitis	10 (19.2%)	13 (26.5%)	17 (37.8%)	6 (15%)	7 (22.6%)	9 (32.1%)
Upper respiratory tract infection	5 (9.6%)	0 (0.0%)	5 (11.1%)	3 (7.5%)	4 (12.9%)	4 (14.3%)
Vision Blurred	4 (7.7%)	5 (10.2%)	3 (8.9%)	3 (7.5%)	5 (16.1%)	2 (7.1%)
Vomiting	10 (19.2%)	8 (16.35)	2 (4.4%)	6 (15.0%)	3 (9.7%)	4 (14.3%)

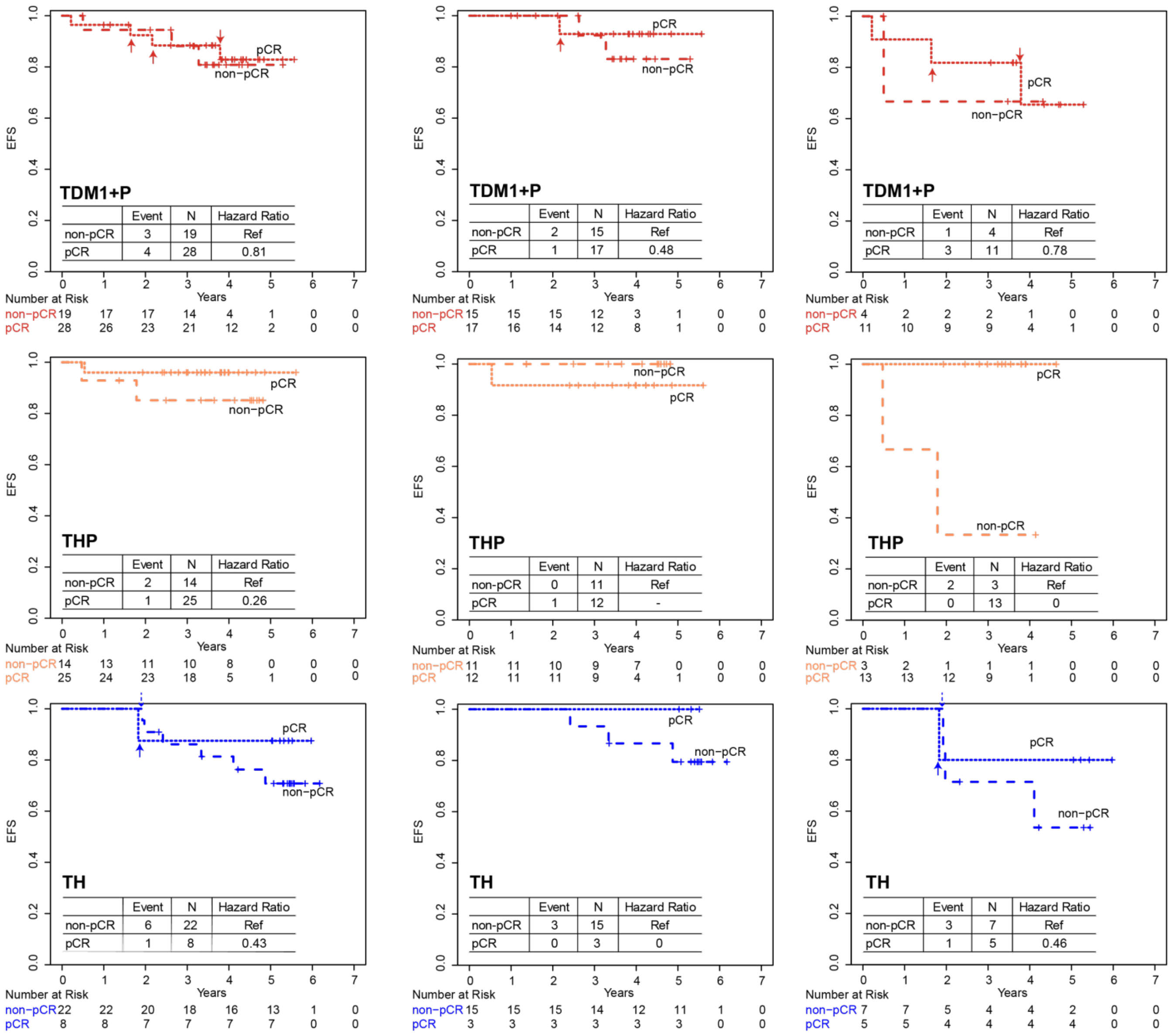

Supplementary Figure 1: Kaplan-meier survival plots of event-free survival for each experimental arm compared to control in all patients and in HR subtypes by pCR status. Left column: All HER2+ patients; middle column: HR+HER2+; Right column: HR-HER2+

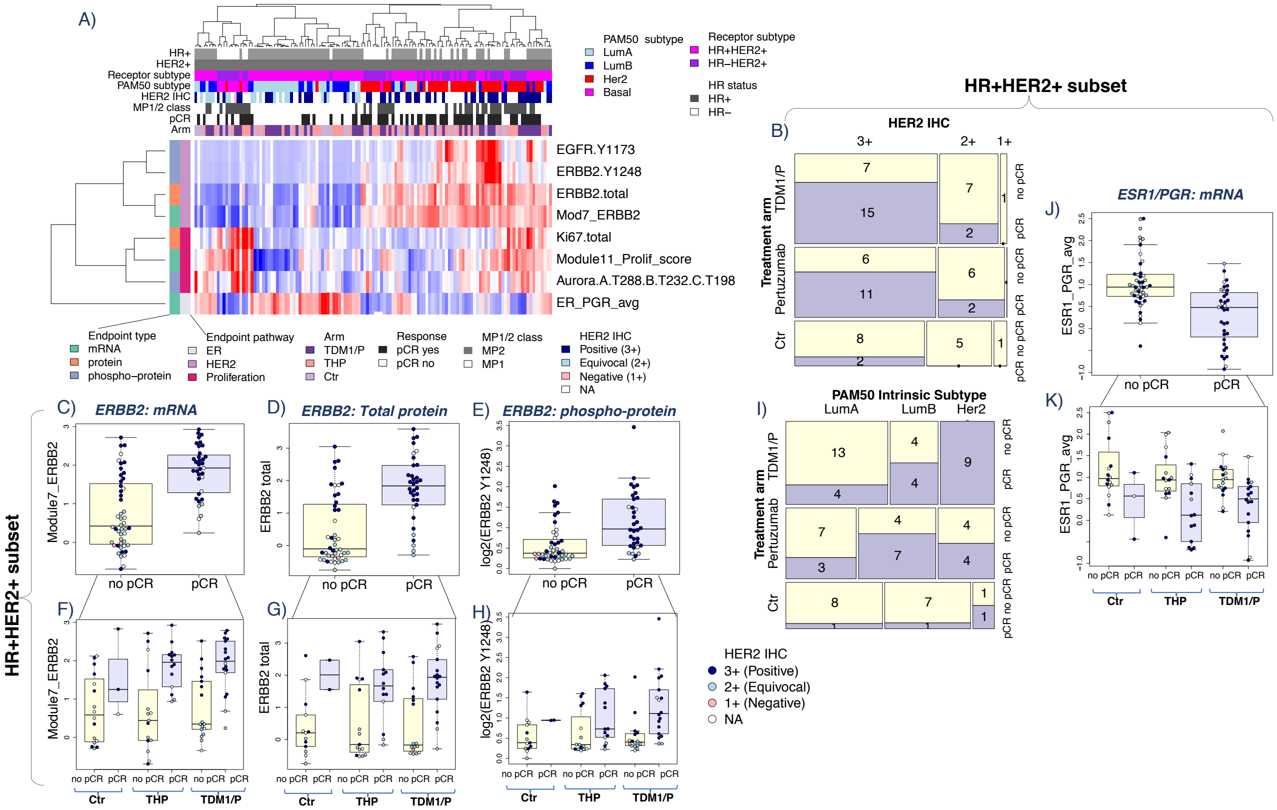

Supplementary Figure 2: All-arm biomarker heatmap and HR+HER2+ subset associations between HER2 and ER family biomarkers and pCR. A) Clustered heatmap of biomarkers (rows) and patient samples (columns), with samples annotated by HR status/receptor subtype, PAM50 subtype, HER2 IHC level, MP1/2class, response, and arm; and biomarkers annotated by pathway (ER, HER2, or Proliferation) and type (mRNA, protein, or phospho-protein). In the heatmap, red=high and blue=low expression. HR+HER2+ subset: B) Mosaic plot showing the proportion of patients achieving pCR (purple) as a function of HER2 IHC level (1+, 2+, 3+; left to right), in the TDM1/P (top), THP (middle) and control (bottom) arms. (C-H) show response-association boxplots of HER2 biomarkers on the mRNA (C), protein (D), and phospho-protein (E) levels in all HR+HER2+ patients (C-E) and by arm (F-H). (I) Mosaic plot showing the proportion of patients achieving pCR (purple) as a function of PAM50 subtype and arm. (J-K) show response-association boxplots of HR expression (ESR1 and PGR averaged) in all HR+HER2+ patients (J) and by arm (K). For box plots, centre line is group median; upper and lower limits of the box correspond to the 1st and 3rd quartile with whiskers extending to 1.5 times the interquartile range from top/bottom of the box

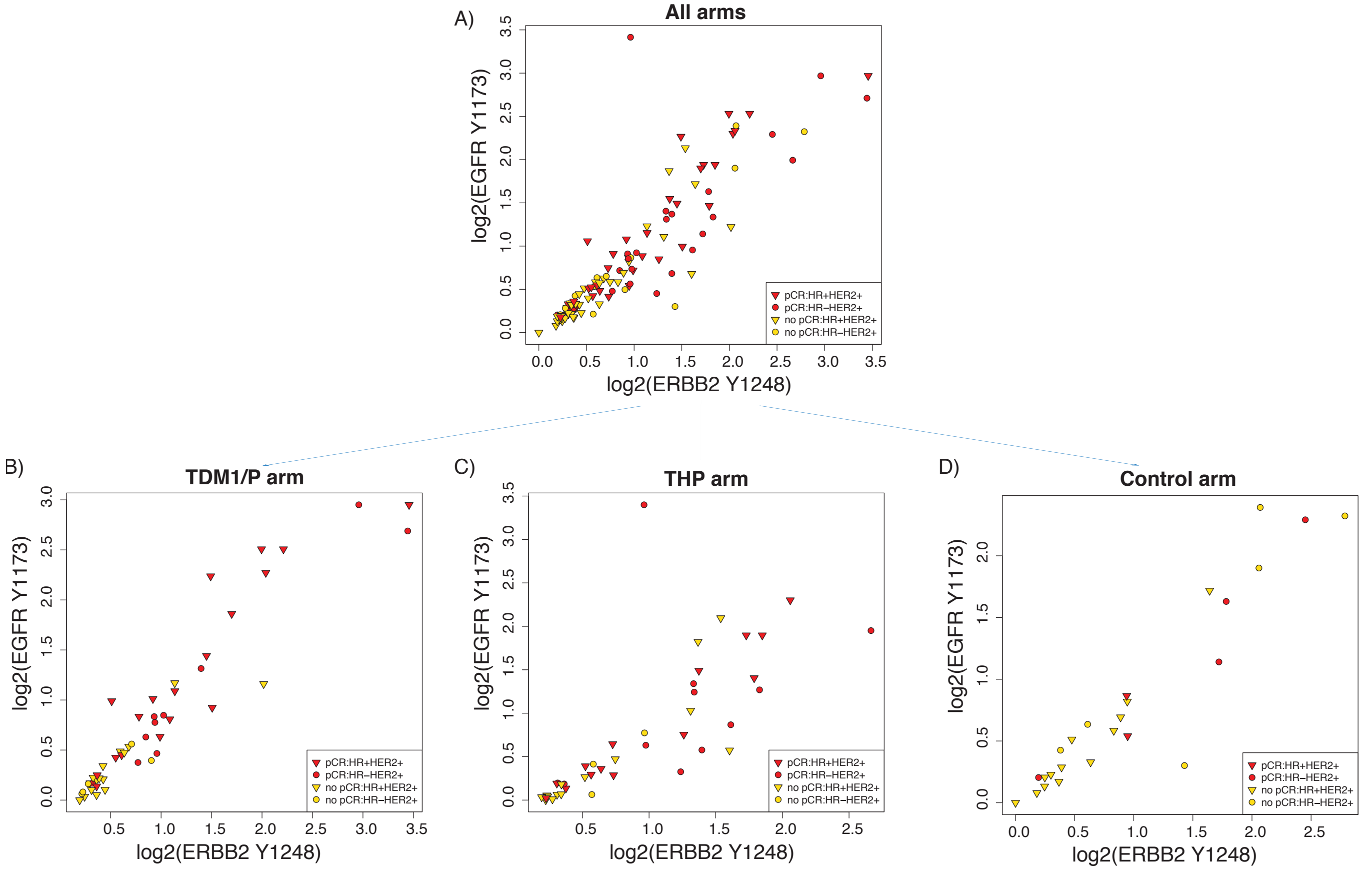

Supplementary Figure 3: ERBB2/EGFR activation vs. response. (A-D) show scatter plots of phospho-ERBB2 vs. phospho-EGFR in all patients (A) and by arm (B-D). Symbol colors/shapes denote response (pCR=orange) and HR status (HR+=triangles))

Supplementary Table 3: Use of Adjuvant Therapy

	TH (n=30)		TDM1+P (n=46)		THP (n=36)	
**HER2+**	**non-pCR** (n=22)	**pCR** (n=8)	**non-pCR** (n=19)	**pCR** (n=27)	**Non-pCR** (n=13)	**pCR** (n=23)
**Trastuzumab**	21 (95%)	8 (100%)	18 (95%)	26 (96%)	11 (85%)	22 (96%)
**Chemotherapy**	0 (0%)	0 (0%)	7 (37%)	0 (0%)	0 (0%)	0 (0%)
**Other HER2-targeted therapy**	1 (5%)	0 (0%)	4 (21%)	0 (0%)	2 (15%)	0 (0%)
**HR+**	**non-pCR** (n=15)	**pCR** (n=3)	**non-pCR** (n=15)	**pCR** (n=16)	**Non-pCR** (n=10)	**pCR** (n=12)
**Endocrine therapy**	15 (100%)	3 (100%)	14 (93%)	15 (93%)	10 (100%)	11 (92%)

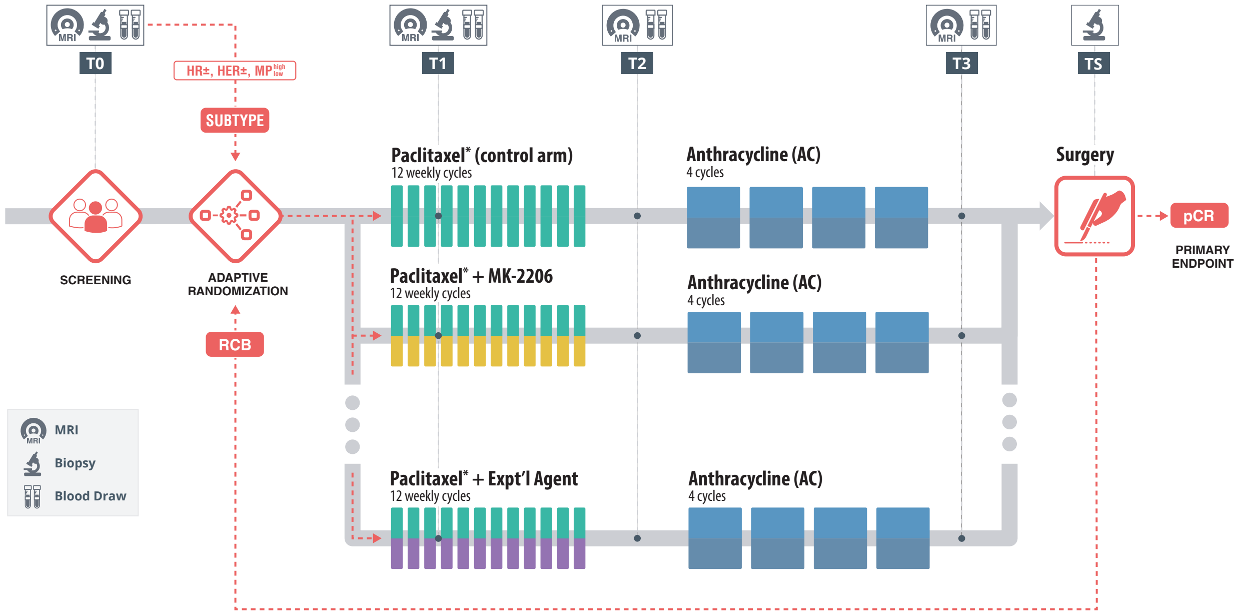

* Patients who are HER2+ may also receive tastuzumab (Herceptin)

Supplementary Figure 4: I-SPY2 study schema

The I-SPY2 TRIAL is an ongoing standing platform trial with multiple drug arms, which enter and leave the trial at different times. New agents are selected and added to the trial as others leave the trial for success (graduate) or futility based on their efficacy in targeted patients. A drug arm may also leave the trial for safety concerns as recommended by the independent Data Safety Monitoring Board (DSMB). A Master Protocol governs the entire trial with each drug arm protocol as a separate Appendix. The Master Protocol contains the statistical analysis plan in Appendix A.

This publication covers drug arms that entered the platform trial from the beginning of the study until Amendment 14. The last drug arm introduced in Amendment 14 that is included in this analysis exited the randomization engine on August 17, 2015. We cannot share information or appendices of drugs which are still currently in the trial or in process for a peer-reviewed publication and thus have redacted these drug arms.

The supplemental contains the following:

1) The last protocol amendment for the last drug arm used in this publication, I-SPY2 TRIAL standing platform Master Protocol Amendment 14, October 5, 2015 (Redacted), including:
    a. Appendix A Statistical Considerations -further details of statistical design and analysis
2) Summary of changes to the protocol until Protocol Amendment 14, October 5,2015.

Quantum Leap Healthcare Collaborative

Summary of Changes within the I-SPY2 TRIAL Master Protocol until Amendment 16.

Clinicaltrials.gov Identifier: NCT01042379

Master Protocol Version	Protocol Date	Description of Changes
Amendment 1	9/30/2009	• **Protocol development**, includes investigational agent Paclitaxel + Figitumumab (CP-751,871) followed by AC and Paclitaxel + Neratinib (HKI-272) followed by AC
Amendment 2	11/6/2019	• **Protocol development**
Amendment 2.1	11/13/2009	• **Protocol development.**<br>• Addition of investigational drug regimen Appendixes and consents (Appendix F Paclitaxel + ABT-888 (Veliparib) + Carboplatin followed by AC)
Amendment 3	2/1/2010	• Modifications to the Model Screening Informed Consent, Treatment Informed Consent, Trastuzumab Informed Consent, Figitumumab Informed Consent, Neratinib Informed Consent, ABT-888 Informed Consent<br>• Update Appendix D – F in various sections (Investigational Agent Information Summary, Investigational Study Agent Administration, Concomitant Medications, Investigational Study Agent)<br>• Administrative changes.
Amendment 4	4/27/2010	• Addition of investigational drug regimen Appendixes and consents (Appendix G Paclitaxel + Conatumumab (AMG 655) followed by AC, Appendix H HER2- Paclitaxel + AMG-386 (Trebananib) followed by AC)<br>• Amended Section 4.1.2 inclusion criteria to better define D. No uncontrolled or severe cardiac disease and E. No clinical or imaging evidence of distant metastases<br>• Amended Section 4.2 exclusion criteria Uncontrolled intercurrent illness

		• Clarification of eligibility criteria source of information<br>• Clarification of MammaPrint score cut-points for eligibility and randomization<br>• Update to Section 6 “Investigational Agent Information (Appendices C and D-x)” to describe process for adding or dropping/graduating a drug from the study<br>• Modified Section 8.2 “Baseline Testing/Pre-treatment Evaluation” Added laboratory blood tests: CBC with differential, Electrolyte Panel, Kidney function tests<br>• Revised Section 8.5 “Post-surgery Follow-up”, the timeframe for collection of new adverse events, except for a subset, was revised from 6 and 12 months to 30- and 60-days post-surgery; continuing AEs will be monitored until resolution.<br>• Modifications to the Model Screening Informed Consent, Standard Chemotherapy Consent, Figitumumab Treatment Consent, Neratinib Treatment Consent, ABT-888 Treatment Consent, ABT-888 and Carboplatin Treatment Consent,<br>• Updated Appendix A I-SPY 2 Statistical Considerations and changed assumption of accrual rate from 30 to 15 patients per month.<br>• Clarification of dose modifications regarding missed doses of paclitaxel in Appendix B Modified NCCN Guidelines, Dose Modifications, and Management of Standard Therapy Toxicity<br>• Title change and restructuring of Appendix C “Overview of Investigational Agents and Biomarkers”<br>• Updates to Appendix D Figitumumab, Appendix E Neratinib, Appendix F ABT-888<br>• Administrative changes.
Amendment 5	2/1/2011	• Added Section 5.5 Doxorubicin Shortage Guidelines - Inclusion of guidelines for substitution of epirubicin instead of doxorubicin in case of national shortage.<br>• Clarifications in Section 4 Participant Selection

		• Added Section 7.2.1 MRI Scan Protocol<br>• Clarifications in Section 8 Clinical Evaluations and Procedures<br>• Removed new adverse event collection at 60 days post-surgery<br>• Clarifications on when lab tests can be done<br>• New participant materials.<br>• Clarification of all eligibility requirements in Appendix C "Summary of All Additional Eligibility Criteria Required for All Investigational Agents"; in addition to the eligibility criteria in Section 4.1.2.C of the main protocol, participants must also meet all additional eligibility criteria described in Table 2 in order to be eligible for the treatment phase of I-SPY 2.<br>• Updated Appendix E Neratinib with additional required safety labs and clarified dose modification tables<br>• Updated Appendix G AMG 655 (Conatumumab) "This Drug is Released and No Longer Active"<br>• Administrative changes.
Amendment 6	7/6/2011	• Updated Appendix H HER2- Paclitaxel + AMG-386 (Trebananib) followed by AC) given May 2011 Investigator's Brochure<br>• Added to Section 7.3 "When an increase in CEP17 copy number is observed by FISH (i.e., "polysomy"), the patient will be considered HER2+ if the ratio of HER2 signals/nucleus is greater than 6."<br>• Clarification of when screening labs can be used before start of therapy in Section 8.3<br>• Clarification on collection of new adverse events in Section 11.1.5: "New AEs will be collected at 30 days following the patient's surgery; continuing AEs will be monitored until resolution/baseline or 12 months post-surgery, whichever occurs first"<br>• Updated Section 11.2.1 SAE Definition to refer to the new final rule on IND safety reporting (*Fed. Reg.* 75: 59935-59963, September 2010)<br>• Administrative changes.

Amendment 7	11/15/2011	• Addition of new investigational drug regimen Appendixes and consents (Appendix I Paclitaxel + Ganitumab (AMG 479) + Metformin followed by AC)<br>• Added to Section 4.1.1.B Ultrasound was added to the list of methods that could be used to verify radiographic size.<br>• Clarifications in Section 4.1.1 Eligibility Criteria for Initial Screening Phase of I-SPY 2 TRIAL<br>• Clarified in Section 7.3 "A tumor will be considered ER+ and/or PgR+ if there is five percent or greater positive tumor staining."<br>• Added three eligibility criteria and dosing schedule to Appendix C given new AMG 479 arm<br>• Update to Appendix and consents (Appendix E Neratinib, Appendix F ABT-888, Appendix H AMG-386)<br>• Administrative changes.<br>• Update to Supplement 1 "I-SPY 2 Registry Study for Low-risk Subjects" - follow up time period extended from 10 years to 15 years<br>• Addition of Supplement 2 sub-study "Quality of Life Measurement within the I-SPY 2 Study", where paper-based Quality of Life questionnaire introduced.
Amendment 7.1	1/16/2012	• Clarification in Section 8 of timing of assessments and safety reporting<br>• Clarification in Appendix I on metformin use in the Ganitumab arm in context of other oral hypoglycemic drugs or in patients with insulin dependent diabetes and update to eligibility criteria<br>• Administrative changes.
Amendment 8	4/10/2012	• Addition of Section 7.2.1.2 to describe co-study ACRIN 6698 MRI Scan Protocol (a diffusion weighted MRI study) for participating sites.<br>• Clarification on safety reporting including fatal outcome events 30 days post-surgery<br>• Clarification of audiology assessments in Appendix I

Amendment 9	5/31/2012	• Addition of new investigational drug regimen Appendixes and consents (Appendix J Paclitaxel + MK-2206 +/- Trastuzumab followed by AC)<br>• Addition of Section 5.4 "Concomitant Medication".<br>• Clarification on follow up visit window in Section 8.5<br>• Update to Section 11 "Reporting Adverse Events" regarding follow-up AE data collection.<br>• Information in Appendix D Figitumamab and Appendix G Conatumamab, and their respective informed consent forms removed since agents were not activated in the study<br>• Update to dose modification tables in Appendix B<br>• Updates to Supplemental 2 Low-risk Registry
Amendment 10	1/16/2013 (released 3/6/2013)	• Addition of new investigational drug regimen Appendixes and consents (Appendix H HER2+ Paclitaxel + AMG 386 (Trebananib) + Trastuzumab followed by AC, Appendix K HER2+ Paclitaxel + Pertuzumab + Trastuzumab followed by AC, Appendix L HER2+ Trastuzumab Emtansine (T-DM1, Trastuzumab-MCC-DM1) + Pertuzumab followed by AC)<br>• Updated Section 3.4 "Adaptive Randomization of Investigational Agents" and Section 6 "Investigational Agent Information" - Updated number of investigational agents from five to eight<br>• Added new Section 8.6 "Evaluations for Premature Discontinuation of Investigational Agent(s)" - Participants who discontinue their randomized treatment assignment prematurely for any reason will be offered the option of remaining "on study" to complete the remaining study procedures and follow-up. Regardless of whether a participant chooses to stay on study, we will collect safety labs and assessment tests 30 days after last dose of investigational agent.<br>• Update Section 4.1.2 "Inclusion Criteria for Treatment Phase of I-SPY 2 TRIAL" and Section 8.2 "Baseline Testing/Pretreatment Evaluation" - Defined parameters around allowable time to complete screening tests

		• Administrative changes.
Amendment 11	7/28/2014	• Addition of new investigational drug regimen Appendixes and consents (Appendix M Paclitaxel + Ganetespib (STA-9090) followed by AC)<br>• Administrative changes.<br>• Update of non-profit sponsor from Foundation for the National Institutes of Health (FNIH) to Quantum Leap Healthcare Collaborative (QLHC).
Amendment 12	12/19/2014	• Administrative changes.<br>• Addition of sub-study SURMOUNT (Surveillance Markers of Utility for Recurrence after Neoadjuvant Therapy for Breast Cancer). Added collection of circulating tumor cells (CTCs) in peripheral blood longitudinally and/or disseminated tumor cells (DTCs) in bone marrow at surgery/recurrence
Amendment 13	5/15/2015	• Addition of new investigational drug regimen Appendixes and consents (Appendix N Paclitaxel + PLX3397 (Pexidartinib) followed by AC)<br>• Update to Section 3.4 "Adaptive Randomization of Investigational Agents", 13 "Statistical Considerations", Appendix A "Statistical Considerations". Revised and updated with new language to reflect the change in minimum and maximum number of participants to be enrolled if an investigational agent is only open to either HER2 positive or HER2 negative participants (n=75 participant cap).<br>• Update to Section 8.6 "Evaluations for Premature Discontinuation of Investigational Agents(s)" to clarify process for participants who discontinue study treatment prematurely and procedures that must still be followed (including S/AE and follow up period)<br>• Update to Section 11 "Reporting Adverse Events" to clarify safety collection and follow up time period<br>• Added Section 8.7 "Disease Progression"<br>• Administrative changes.<br>• Close accrual to co-study ACRIN6698.

Amendment 14	10/5/2015	• Addition of new investigational drug regimen Appendixes and consents (Appendix O Paclitaxel + Pembrolizumab (4-cycles) followed by AC)<br>• Administrative changes.

Study Timeline:

1. March 29, 2010: First patient screened on Protocol Amendment 3, version 2/1/2010
2. April 12, 2010: First patient randomized and consented to drug regimen (treatment or control)
3. August 7, 2015: Efficacy reports using the *time adjusted model* is evaluated by the independent DSMB to graduate arm regimens.
4. May 2, 2016: Time adjusted model is used in the randomization engine to randomize participants to drug regimens. Formally updated into the master protocol amendment 16, Appendix A.

I-SPY 2 TRIAL
(**I**nvestigation of **S**erial Studies to **P**redict **Y**our **T**herapeutic **R**esponse with **I**maging **A**nd mo**L**ecular Analysis **2**)

UCSF Protocol #: *097517*

Organization Name: University of California, San Francisco

Protocol Principal Investigator:
Laura Esserman, MD, MBA
Dept of Surgery M2 Box 1710
San Francisco, CA 94143
Phone: 415-885-7691 FAX: 415-353-9571
Laura.Esserman@ucsf.edu

Organization: University of California, San Francisco
Co-Investigator(s):
Amy Jo Chien, MD
Helen Diller Family Comprehensive Cancer Center
Box 1710
San Francisco, CA 94143-1710
Tel: 415-443-4296 FAX: 415-353-9592
Jo.Chien@ucsf.edu

University of Pennsylvania
Angela DeMichele, MD, MSCE
3 Perelman Center
3400 Civic Center Blvd.
Philadelphia, PA 19104
Tel: 215-614-1850 FAX: 215-614-3349
Angela.DeMichele@uphs.upenn.edu

University of Minnesota
Doug Yee, MD
420 Delaware Dt., SE, MMC 480
Minneapolis, MN, 55455
Tel: 612-626-8487 FAX: 612-626-3069
yeexx006@umn.edu

Arizona Cancer Center at UMC-North
Rebecca Viscusi, MD
The University of Arizona Medical Center
Surgical Oncology
PO Box 245131
Tucson, AZ 85724-5131
Tel: 520-626-4441 FAX: 520-626-7785
rviscusi@surgery.arizona.edu

Robert Livingston, M.D.
Arizona Cancer Center at UMC North
3838 N. Campbell Ave
Tucson, AZ 85719

CONFIDENTIAL

Tel: 520-626-4175 FAX: 520-626-3754
rlivingston@azcc.arizona.edu

British Columbia Cancer Agency
Stephen Chia
Tel: (604) 877-6000
schia@bccancer.bc.ca

Emory University Winship Cancer Institute
Amelia Zelnak, MD
Winship Cancer Institute, Emory University
1365-C Clifton Road, NE
Atlanta, GA 30322
Tel: 404-778-1835 FAX: 404-778-4389
Amelia.zelnak@emory.edu

Georgetown University Lombardi Cancer Center
Claudine Isaacs, MD
3800 Reservoir Rd, NW, 2nd Level Podium B
Washington, DC 20007
Tel: 202-444-3677 FAX: 202-444-9429
isaacsc@georgetown.edu

Inova Fairfax Hospital Cancer Center
Kirsten K, Edmiston MD, FACS
3300 Gallows Rd
Fairfax, VA 22042
Tel: 703-776-8675 FAX: 703-776-8713
Kirsten.Edmiston@inova.org

Loyola University Cardinal Bernardin Cancer Center
Kathy S. Albain, MD
2160 South First Ave, Room 109
Maywood, IL 60153
Tel: 708-327-3102 FAX: 708-327-2210
kalbain@lumc.edu

Mayo Clinic–Scottsdale
Donald Northfelt, MD
13400 E. Shea Blvd
Scottsdale, AZ 85259
Tel: 480-301-8335 FAX 480-301-6993
northfelt.donald@mayo.edu

Mayo Clinic Breast Cancer Center–Rochester
Judy C. Boughey, MD
200 First St, SW
Rochester, MN 55905
Tel: 507-284-8392 FAX: 507-284-5196
boughey.judy@mayo.edu

CONFIDENTIAL

Moffitt Cancer Center
Susan E. Minton, DO
2902 USF Magnolia Drive,
Tampa, FL 33612
Tel: (813) 745-2105
susan.minton@moffitt.org

Oregon Health and Science University
Kathleen Kemmer
3181 SW Sam Jackson Park Rd
Portland, OR 97239
Tel: (503) 494-8573
Kemmerk@ohsu.edu

Swedish Cancer Institute
Henry Kaplan, MD
1221 Madison Street
Seattle, WA 98104
Tel: 206 386-2828
Hank.Kaplan@swedish.org

University of Alabama at Birmingham
Comprehensive Cancer Center
Andres Forero
1802 Sixth Avenue South
2510 North Pavilion
Birmingham, AL 35294-3300
Tel:(205) 975-2837 FAX: (205) 996-7560
andres.forero@ccc.uab.edu

University of California San Diego Moores Cancer Center
Anne Wallace, MD
3855 Health Sciences Dr., M/C 0698
La Jolla, CA 92093
Tel: 858-822-6194 FAX: 822-6194
amwallace@ucsd.edu

University of Chicago Medical Center
Rita Nanda, MD
5841 S. Maryland Avenue, MC 2115
Chicago, IL 60437
Tel: 773-834-2756 FAX: 773-702-9268
rnanda@medicine.bsd.uchicago.edu

University of Colorado Cancer Center
Anthony Elias, MD
1665 Aurora Ct., Rm. 3200, MS F700
Aurora, CO 80045
Tel: 720-848-1622 FAX: 720-848-0671
Anthony.Elias@ucdenver.edu

CONFIDENTIAL

University of Kansas Medical Center
Qamar Khan, MD
2330 Shawnee Mission Pkwy, Ste 210
Westwood, KS 66205
Tel: 913-588-7791 FAX: 913-588-3679
qkhan@kumc.edu

University of Southern California,
Norris Comprehensive Cancer Center
Julie Lang, MD
1450 Biggy Street
Norris Research Tower (NRT) 3505
Norris Comprehensive Cancer Center
Los Angeles CA 90033
Tel: (323) 865-3900
julie.lang@med.usc.edu

University of Texas, M. D. Anderson Cancer Center
Stacy Moulder, MD,
Breast Medical Oncology Dept. – Unit 1354
1515 Holcombe Blvd.
Houston, TX 77030
Tel: 713-792-2817FAX: 713-794-4385
smoulder@mdanderson.org

University of Texas, Southwestern Medical Center
Barbara Haley, MD
5323 Harry Hines Blvd, Bldg E6.222D
Dallas, TX 75390-9155
Tel: 214-648-4180 FAX: 214-648-7965
barbara.haley@utsouthwestern.edu

University of Washington
Larissa Korde
825 Eastlake Ave East
Seattle, WA 98109-1023
Tel: (206) 288-7409 FAX: (206) 288-2054
lkorde@uw.edu

Organization: **University of Texas M.D. Anderson Cancer Center**
Statistician: *Donald Berry, PhD*
1515 Holcombe Ave
Houston, TX 77030-4009
Tel: 713-745-5509 FAX: 713-792-4252
dberry@mdanderson.org

IND Sponsor: **QuantumLeap Healthcare Collaborative**
3450 California Street
San Francisco, CA 94118
415-476-0270

CONFIDENTIAL

**IND#**	**105,139**
**Agent(s)/Supplier:**	**See Appendices D−N**
**Protocol Version Date:**	October 05, 2015
**Protocol Revision or Amendment No.:**	**14**

CONFIDENTIAL

SCHEMA

Figure A: I-SPY 2 TRIAL(**I**nvestigation of **S**erial Studies to **P**redict **Y**our **T**herapeutic **R**esponse with **I**maging **A**nd mo**L**ecular Analysis **2**)

n

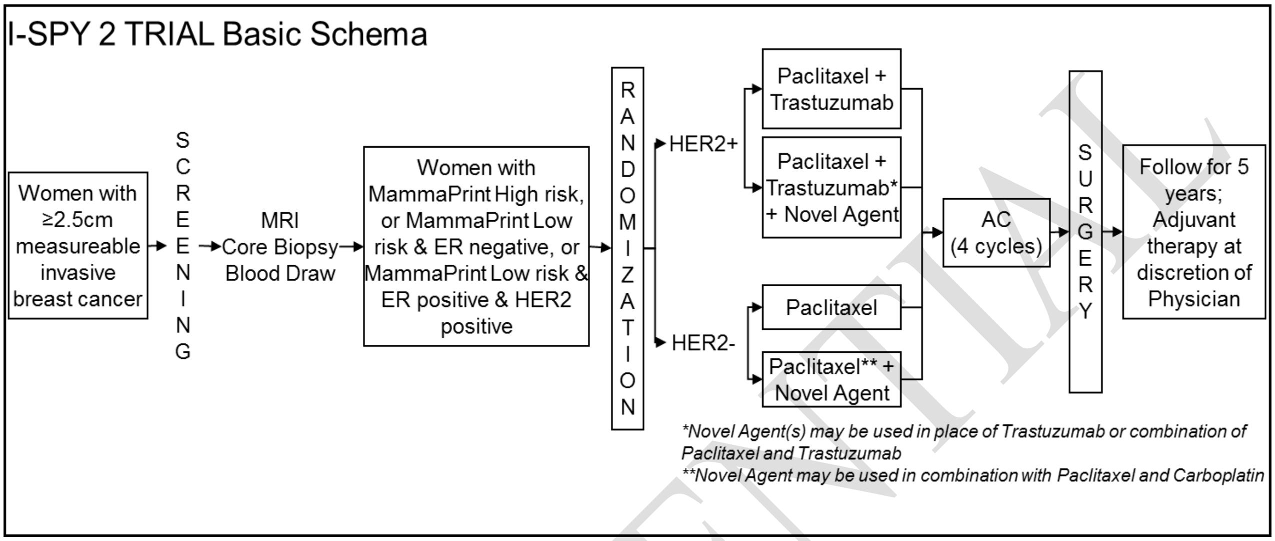

Figure B: I-SPY 2 Adaptive TRIAL Schema: Screening Tumor Eligibility & Randomization

CONFIDENTIAL

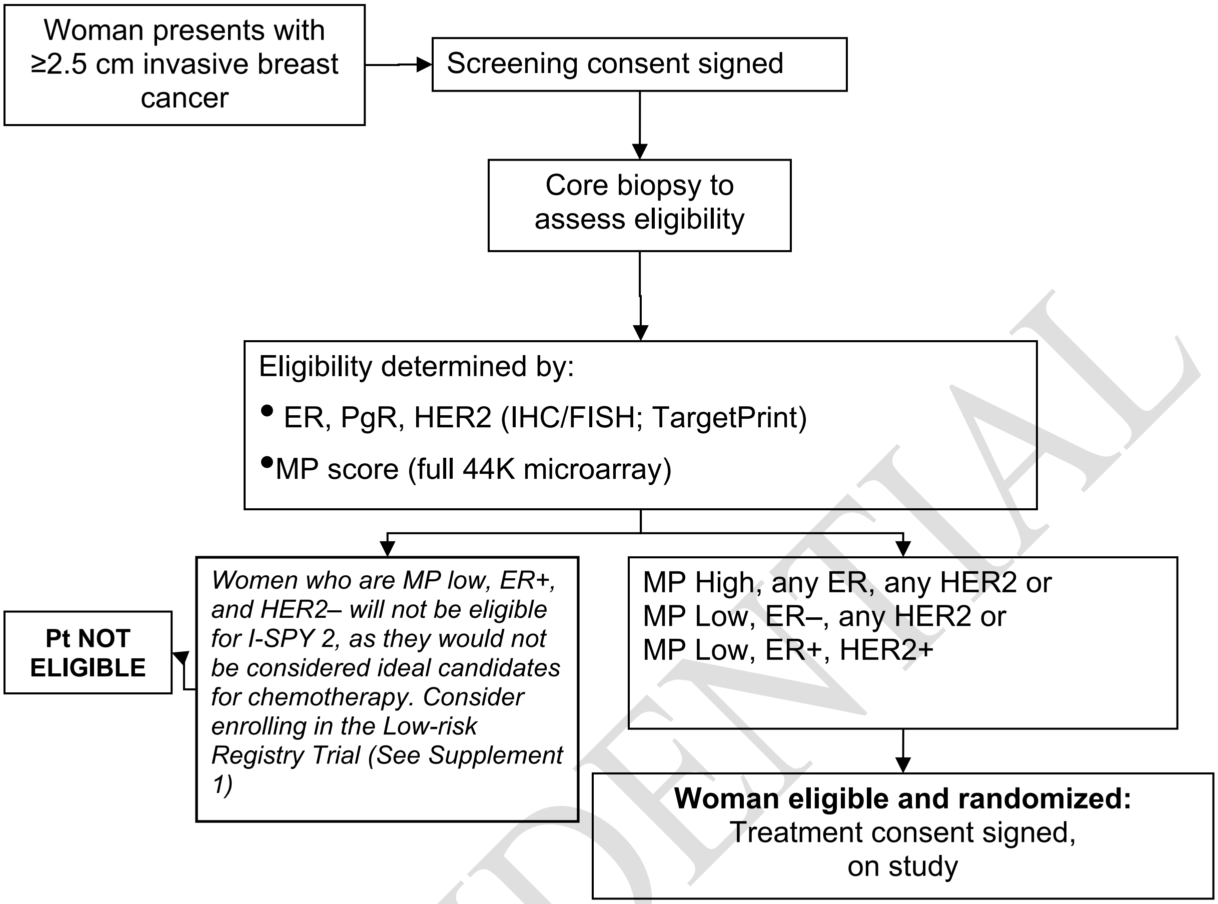

CONFIDENTIAL

<u>**Figure C: Schedule of Study Procedures**</u>

Pretreatment (Time Point 0)	Early Paclitaxel (Time Point 1)	Inter-regimen (Time Point 2)	Pre-surgery/Surgery (Time Point 3)
MRI *(within 30 days prior to randomization)*	MRI *(end of week 3, prior to cycle 4)*	MRI *(prior to AC, at least 1 day after last paclitaxel)*	MRI *(prior to surgery, at least 2–3 weeks after AC)*
Core Biopsy *(prior to randomization)*	Core Biopsy *(end of wk 3, prior to cycle 4)*	---- ---- ----	Surgical Tissue *(at time of surgery)*
Blood Draw *(prior to randomization)*	Blood Draw *(end of week 3, prior to cycle 4)*	Blood Draw *(prior to AC, at least 1 week after last paclitaxel)*	Blood Draw *(prior to surgery, at least 2–3 weeks after AC)*

Abbreviations: AC = anthracycline; MRI = magnetic resonance imaging.

<u>**Figure D: I-SPY 2 TRIAL, Adaptive Overall Study Schema**</u>

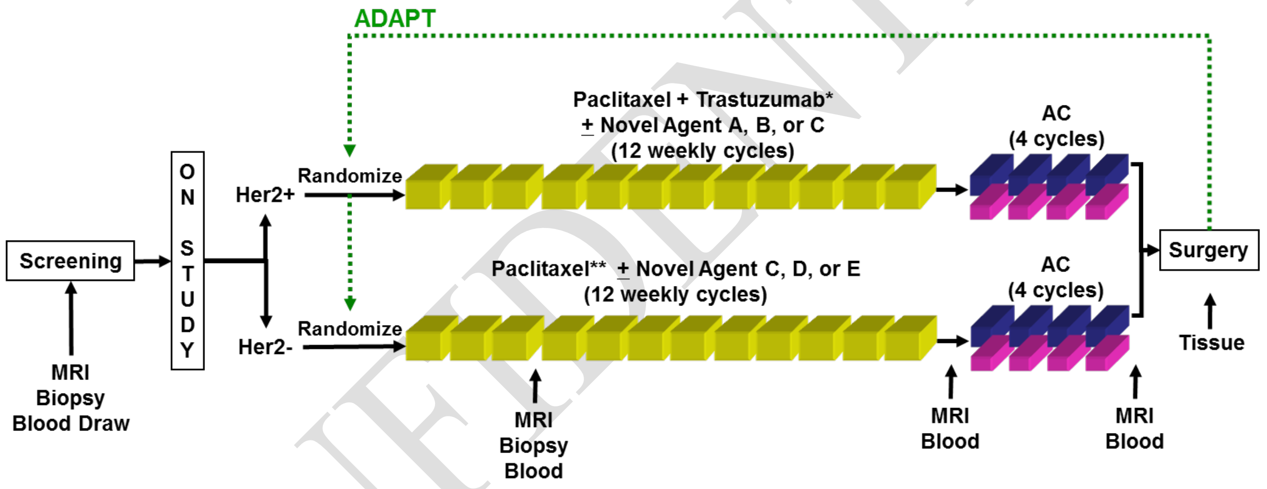

**Novel Agent may be used in place of Trastuzumab or combination of Paclitaxel and Trastuzumab in Experimental Treatment Arm (see specific Novel Agent Appendix)*
***Novel Agent may be used in combination with Paclitaxel and Carboplatin in Experimental Treatment Arm (see specific Novel Agent Appendix)*

CONFIDENTIAL

TABLE OF CONTENTS

SCHEMA vi
TABLE OF CONTENTS ix
1. OBJECTIVES 1
1.1 Primary Objective 1
1.2.1 Predictive and Prognostic Indices 1
1.2.2 Biological Specimen Resource and Imaging Data Base 1
1.2.3 Relapse-free Survival 1
1.2.4 Investigational Agent Safety 1
2. BACKGROUND 2
2.1 Breast Cancer 2
2.1.1 Neoadjuvant Therapy for Breast Cancer 2
2.2. The I-SPY TRIAL 4
2.2.1. Results 4
2.3 Rationale: Neoadjuvant Adaptive Design Approach 9
2.4 Study Agent Rationale 10
2.4.1 Agent Selection 10
2.4.2 Designation of Tier 1 versus Tier 2 Agents for the I-SPY 2 TRIAL Process 11
2.4.3 I-SPY 2 Investigational Tier 1 Agents 11
2.5 General Approach in Evaluating Agent(s) and Biomarkers 12
3. SUMMARY OF STUDY PLAN 13
3.1 Screening Phase 13
3.2 Treatment Phase 14
3.3 Biomarkers 14
3.4 Adaptive Randomization of Investigational Agents 15
3.5 Trial Informatics 16
4. PARTICIPANT SELECTION 17
4.1 Inclusion Criteria Overview 17
4.1.1 Eligibility Criteria for Initial Screening Phase of I-SPY 2 TRIAL 17
4.1.2 Inclusion Criteria for Treatment Phase of I-SPY 2 TRIAL 18
4.2 Exclusion Criteria 19
4.3 Inclusion of Women and Minorities 19
4.4 Recruitment and Retention Plan 20
5. CHEMOTHERAPY ADMINISTRATION 21
5.1 Standard Chemotherapy Treatment Plan for Control Arm, HER2– Tumors 21
5.2 Standard Chemotherapy Treatment Plan for Control Arm, HER2+ Tumors 21
5.3 Investigational Agent Treatment Plan 22
5.4 Premedications for Paclitaxel, Trastuzumab, and Doxorubicin plus Cyclophosphamide 22
5.5 Concomitant Medication 22
5.6 Toxicity Management and Dose Modifications 22
5.7 Doxorubicin Shortage Guidelines 22
5.8 Adjuvant Treatment Recommendations 23
6. INVESTIGATIONAL AGENT INFORMATION (See Appendices C and D–x) 24
7. BIOMARKERS FOR ELIGIBILITY, STRATIFICATION, AND RESPONSE MONITORING 26
7.1 Tissue and Blood Specimens for Biomarker Assessment 26
7.2 MRI and MRI Functional Tumor Volume (FTV) for Biomarker Assessment 26
7.2.1 MRI Scan Protocol 26

CONFIDENTIAL

7.3 Incorporation of Established Biomarkers....28
7.4 Incorporation of Qualifying Biomarkers....28
7.5 Incorporation of Exploratory Biomarkers....29
7.6 Repository for Storing, Analyzing, and Comparing Assay Results....30

8. CLINICAL EVALUATIONS AND PROCEDURES....31

8.1 Schedule of Events....31
8.2 Baseline Testing/Pretreatment Evaluation....31
8.3 Evaluations During Neoadjuvant Chemotherapy Treatment....32
8.4 Evaluations at Completion of Neoadjuvant Chemotherapy Treatment....34
8.5 Postsurgery Follow-up....34
8.6 Evaluations for Premature Discontinuation of Study Treatment....35
8.7 Disease Progression....35

9. CRITERIA FOR EVALUATION AND ENDPOINT DEFINITION....36

9.1 Primary Endpoint....36
9.2 Secondary Endpoints....36
9.2.1 MRI FTV....36
9.2.2 Residual Cancer Burden....36
9.2.3 Three- and Five-year Relapse/RFS and OS....37
9.3 Off-Investigational Agent Criteria....37
9.4 Off-study Criteria....37
9.5 Study Termination....37

10. SPECIMEN MANAGEMENT....38

10.1 Central Laboratories....38
10.2 Specimen Collection, Handling, and Shipping Procedures....38
10.3 Ancillary Laboratories....39
10.3.1 Agendia....39
10.3.2 George Mason University (GMU)....39
10.3.3 UCSF I-SPY Laboratory....40
10.3.4 UCSF CLIA Pharmacogenomics....40
10.3.5 MDACC Molecular Laboratory....40
10.3.6 Investigational Agent Response Biomarkers – IGFR Inhibitor....40
10.3.7 Investigational Agent Response Biomarkers – Pan ErbB Inhibitor....41
10.3.8 Additional Samples....41

11. REPORTING ADVERSE EVENTS....42

11.1 Adverse Events....42
11.1.1 Reportable AEs....42
11.1.2 AE Data Elements....42
11.1.3 Severity of AEs....42
11.1.4 Assessment of Relationship of AE to Treatment....43
11.1.5 Follow-up of AEs....44
11.2 Serious Adverse Events....44
11.2.1 SAE Definition....44
11.2.2 Reporting Serious Adverse Events to QLHC*....44*

12. STUDY OVERSIGHT AND MONITORING....45

CONFIDENTIAL

12.1 Data Management....46
12.2 Case Report Forms....46
12.3 Source Documents....47
12.4 Data and Safety Monitoring Plan....47
12.5 QLHC or FDA Monitoring....48
12.6 Record Retention....48
12.7 Clinical Trials Agreement....48

13. I-SPY 2 STATISTICAL CONSIDERATIONS....49

13.1 Sample Size/Accrual Rate....49
13.2 Randomization and Stratification Using Biomarker Signatures....49
13.3 Primary Endpoint and Probability of Success by Biomarker Signature....51
13.4 Design Algorithm Overview....51
13.5 Assessing Operating Characteristics of the Design via Simulation....52

14. ETHICAL AND REGULATORY CONSIDERATIONS....68

14.1 Form FDA 1572....68
14.2 Other Required Documents....68
14.3 IRB Approval....69
14.4 Informed Consent....69
14.5 Submission of Regulatory Documents....70
14.6 Other....70

15. FINANCING, EXPENSES, AND/OR INSURANCE....71

REFERENCES....72

Model Informed Consent Forms....75

SCREENING INFORMED CONSENT....75
STANDARD CHEMOTHERAPY TREATMENT INFORMED CONSENT....88
SUPPLEMENT FOR TRASTUZUMAB TREATMENT....101
SUPPLEMENT FOR FIGITUMUMAB TREATMENT....104
SUPPLEMENT FOR NERATINIB TREATMENT....105
SUPPLEMENT FOR ABT 888 AND CARBOPLATIN TREATMENT....110
SUPPLEMENT FOR CONATUMUMAB TREATMENT....113
SUPPLEMENT FOR AMG 386 TREATMENT....114
SUPPLEMENT FOR TRASTUZUMAB (HERCEPTIN) PLUS AMG 386 TREATMENT....118
SUPPLEMENT FOR GANITUMAB (AMG 479) TREATMENT....123
SUPPLEMENT FOR MK-2206 TREATMENT....128
SUPPLEMENT FOR TRASTUZUMAB (HERCEPTIN) PLUS MK-2206 TREATMENT....134
SUPPLEMENT FOR PERTUZUMAB AND TRASTUZUMAB TREATMENT....141
SUPPLEMENT FOR T-DM1 (TRASTUZUMAB-EMTANSINE) AND PERTUZUMAB TREATMENT....145
SUPPLEMENT FOR GANETESPIB TREATMENT....149
SUPPLEMENT FOR PLX3397 TREATMENT....154
SUPPLEMENT FOR PEMBROLIZUMAB TREATMENT....162
I-SPY 2 SUBSTUDY: SURMOUNT....168

Appendix A I-SPY 2 Statistical Considerations....180

Appendix B Modified National Comprehensive Cancer Network (NCCN) Guidelines, Dose Modifications, and Management of Standard Therapy Toxicity....184

Appendix C Overview of Investigational Agents and Biomarkers....209

Appendix K Pertuzumab with Trastuzumab....213

Appendix L Pertuzumab + Trastuzumab Emtansine (T-DM1, Trastuzumab-MCC-DM1)....241

Supplement 1 I-SPY 2 Registry Study for Low-risk Subjects.... **Error! Bookmark not defined.**

CONFIDENTIAL

Supplement 2 Quality of Life Measurement within the I-SPY 2 Study...................... **Error! Bookmark not defined.**
Supplement 3 SURMOUNT study within the I-SPY 2 Study 288

CONFIDENTIAL

LIST OF ABBREVIATIONS

AC	Doxorubicin/cyclophosphamide
ACRIN	American College of Radiology Imaging Network
AE	Adverse event
ALP	Alkaline phosphatase
ALT	Alanine transaminase
AMPK	AMP-activated protein kinase
AST	Aspartate aminotransferase
ATP	Adenosine triphosphatase
AUC	Area under the concentration-time curve
BCS	Breast-conserving surgery
BSI	Brief Symptom Inventory
BUN	Blood urea nitrogen
caBIG	Cancer Biomedical Informatics Grid
CALGB	Cancer and Leukemia Group B
CAPMM	Center for Applied Proteomics and Molecular Medicine
CBC	Complete blood count
CBIIT	Center for Biomedical Informatics and Information Technology
CDE	Common Data Element
cCR	Clinical complete response
cDNA	Complementary DNA
CGH	Comparative genomic hybridization
CHF	Congestive heart failure
CI	Confidence interval
CIS	Cancer *in situ*
CL	Clearance
CLIA	Clinical Laboratory Improvement Amendment
CRADA	Cooperative Research and Development Agreement
CRF	Case report form
CT	Computed tomography
CTA	Clinical Trials Agreement
CTDC	Clinical Trial Data Capture
CTCAE	Common Terminology Criteria for Adverse Events
CV	Cardiovascular
CXR	Chest radiograph
DAPC	Data Access and Publications Committee
DCC	Data Coordinating Center
DFS	Disease-free survival
DLT	Dose-limiting toxicity
DSMB	Data Safety Monitoring Board
DT	Distress thermometer
EBC	Early breast cancer
ECOG	Eastern Cooperative Oncology Group
EGFR	Epidermal growth factor receptor
ER	Estrogen receptor
FDA	Food and Drug Administration
FFPE	Formalin fixation and paraffin embedding
FISH	Fluorescence *in situ* hybridization
FNA	Fine-needle aspiration
FNIH	Foundation for the National Institutes of Health

CONFIDENTIAL

List of Abbreviations *(continued)*

FTV	Functional tumor volume
FWA	Federalwide Assurance
5-FU	5-fluorouracil
GFR	Glomerular filtration rate
GMU	George Mason University
GWAS	Genome-wide association study
H&E	Hematoxylin and eosin
HADS	Hospital Anxiety and Depression Scale
HAHA	Human antihuman antibodies
HIPAA	Health Insurance Portability and Accountability Act
HER2	Human epidermal growth factor receptor
HNSCC	Head and neck squamous cell carcinoma
HNSTD	Highest non-severely toxic dose
HR	Hormone receptor (ER + PgR)
HRG	Heregulin
IASC	Independent Agent Selection Committee
ICH GCP	International Conference on Harmonisation Good Clinical Practice
IDE	Investigational Device Exemption
IGF	Insulin-like growth factor
IGF-1	Insulin-like growth factor 1
IGF-1R	Insulin-like growth factor 1 receptor
IGFR	Insulin-like growth factor receptor
IgG_1	Human monoclonal antibody
IHC	Immunohistochemistry
ILD	Interstitial lung disease
IND	Investigational New Drug
IR	Insulin receptor
IRB	Institutional Review Board
I-SPY TRIAL	**I**nvestigation of **S**erial Studies to **P**redict **Y**our **T**herapeutic **R**esponse with **I**maging **A**nd mo**L**ecular Analysis
K-M	Kaplan-Meier
LABC	Locally advanced breast cancer
LVEF	Left ventricular ejection fraction
LKB1	Liver kinase B1
LN	Lymph nodes
MAPK	Mitogen-activated protein kinase
MBC	Metastatic breast cancer
MCC	4-[*N*-maleimidomethyl]cyclohexane-1-carboxylate
MDACC	MD Anderson Cancer Center
MP	MammaPrint
MP–	MammaPrint High1
MP+	MammaPrint High2
MR	Magnetic resonance
MRI	Magnetic resonance imaging
MTD	Maximum tolerated dose
mTOR	Mammalian target of rapamycin
MUGA	Multigated acquisition scan
NCI	National Cancer Institute
NCCN	National Comprehensive Cancer Network

CONFIDENTIAL

List of Abbreviations *(continued)*

NDA	New Drug Application
NIH	National Institutes of Health
NKI 70	Netherlands Cancer Institute 70-gene signature
NOAEL	No observed adverse effect level
NRH	Nodular regenerative hyperplasia
NSABP	National Surgical Adjuvant Breast and Bowel Project
NSCLC	Non-small cell lung carcinoma
OHRP	Office of Human Research Protections
OIVD	Office of *In Vitro* Diagnostics
ORR	Objective response rate
OS	Overall survival
PARP	Polyadenosine diphosphate ribose polymerase
pCR	Pathologic complete response
PD	Progressive disease
PE	Percent enhancement
PFS	Progression-free survival
PET	Positron emission tomography
PgP	P-glycoprotein
PgR	Progesterone receptor
pHER2	Phosphorylated HER2
PI	Principal Investigator
PI3K	Phosphatidylinositol-3-kinase
PK	Pharmacokinetics
PNET	Primitive neuroectodermal tumors
PR	Partial response
PSA	Prostate-specific antigen
QLHC	QuantumLeap Healthcare Collaborative
QOL	Quality of life
RCB	Residual cancer burden
RFS	Relapse-free survival
RPMA	Reverse phase protein microarray
ROC	Receiver operating characteristic
ROR	Risk of recurrence
ROR-S	Retinoid-related orphan receptors
SAE	Serious adverse event
SCC	Squamous cell carcinoma
SD	Stable disease
SER	Signal enhancement ratio
SI	Signal intensity
SMCC	Succinimidyl MCC
SNP	Single-nucleotide polymorphism
SPORE	Specialized Programs of Research Excellence
T4	Tumor growing into the chest wall or skin, including inflammatory breast cancer
TEAE	Treatment-emergent adverse event
TFAC	Paclitaxel (Taxol®), doxorubicin (Adriamycin™) and cyclophosphamide
TRANSCEND	**TRAN**slational Informatics **S**ystem to **C**oordinate **E**merging Biomarkers, **N**ovel Agents and Clinical **D**ata
UCSC	University of California, Santa Cruz
UCSF	University of California, San Francisco

CONFIDENTIAL

List of Abbreviations *(continued)*

VEGF	Vascular endothelial growth factor
VEGFR	Vascular endothelial growth factor receptor
WBC	White blood cell

CONFIDENTIAL

1. OBJECTIVES

1.1 Primary Objective

To determine whether adding investigational agents to standard neoadjuvant paclitaxel (with or without trastuzumab), and/or doxorubicin and cyclophosphamide, increases the probability of pathologic complete response (pCR) over standard neoadjuvant chemotherapy alone, for each biomarker signature established at trial entry, and to determine for each experimental agent used, the predictive probability of success in a subsequent phase 3 trial for each possible biomarker signature.

1.2 Secondary Objectives

1.2.1 Predictive and Prognostic Indices

To build predictive and prognostic indices based on qualification and exploratory markers to predict pCR and residual cancer burden (RCB).

1.2.2 Biological Specimen Resource and Imaging Data Base

To initiate the creation of a Biological Specimen Repository, consisting of tumor tissue, RNA, DNA, serum, and cells, as well as corresponding magnetic resonance (MR) and pathology images of these specimens for ongoing translational studies in genomics, proteomics, and imaging in order to establish their relationship to overall survival (OS).

1.2.3 Relapse-free Survival

To determine three- and five-year relapse-free survival (RFS) and OS among the treatment arms.

1.2.4 Investigational Agent Safety

To determine incidence of adverse events (AEs), serious adverse events (SAEs), and laboratory abnormalities of each investigational agent tested.

CONFIDENTIAL

2. BACKGROUND

2.1 Breast Cancer

Breast cancer is diagnosed in almost 200,000 women annually in the United States alone; 45,000 women still die annually of this disease. Although many women now present with Stage I and II mammographically detected cancers and have excellent outcomes, 10–20% of newly diagnosed breast cancers present as locally advanced breast cancer (LABC) in which the risk of recurrence and death is significantly higher [1]. The absolute numbers of these cancers has not decreased over time [2] and successful treatment options remain limited. Women with LABC are distinct from those with screen-detected cancers. Up to 26% of LABC presents in women under the age of 40, prior to the age when screening is recommended [3]. In women who are being screened, the majority of women with LABC today (84%) present as "interval" cancers, where a palpable mass develops within one to two years of a normal screening mammogram [3]. Women with LABC represent a disproportionately large fraction of those who die from their disease. Since standard of care for these women increasingly includes neoadjuvant therapy prior to surgical resection, this population and setting represent a unique opportunity to learn how to tailor treatment for high-risk breast cancers.

The last decade of cancer research has shown breast cancer to be a heterogeneous disease, suggesting that directing agents to molecular pathways that characterize the disease in subsets of participants will improve treatment efficacy. Today, however, new breast cancer agents are first tested in phase 2 and 3 trials in the metastatic setting, followed by randomized phase 3 registration trials in the adjuvant setting. For the most part, these trials do not consider the specific molecular characteristics of the participant's disease. Moreover, adjuvant trials require long follow-up and many thousands of participants [4]. This process can typically take 15−20 years before marketing approval is gained for successful agents, and a substantial investment in time and resources is often put into agents that ultimately fail. The development and use of biomarkers for early measures of therapeutic response would facilitate the efficient evaluation of new agents in focused early clinical trials [5] and enable the development of more informed, smaller phase 3 trials. Although the use of biomarkers (molecular profiles, protein pathways, imaging, *etc.*) holds promise to enable the tailoring of agents to specific participant populations, developing translational approaches in clinical trials to predict agent response presents a major challenge.

2.1.1 Neoadjuvant Therapy for Breast Cancer

Development of multi-agent adjuvant chemotherapy regimens over the last two decades has substantially improved both disease-specific and OS outcomes for women with breast cancer [6]. The most effective adjuvant combination regimens include anthracyclines, such as doxorubicin or epirubicin (topoisomerase II inhibitors), the alkylating agent cyclophosphamide, and taxanes (currently docetaxel or paclitaxel), which are microtubule stabilizers. These different mechanisms of action often produce synergistic tumor shrinkage and avoid the development of resistance to single-agent treatment [7]. In the case of human epidermal growth factor receptor (HER2)-positive (HER2+) participants, a HER2 targeted monoclonal antibody, trastuzumab, has been shown to significantly improve survival when combined with a taxane-containing regimen [8]. Despite the gains in disease-free survival (DFS) and OS from these combination regimens, especially in hormone receptor-negative (HR–) participants[6], a substantial fraction of participants still relapse and die of breast cancer.

Although adjuvant therapy remains the mainstay of treatment for breast cancer, neoadjuvant chemotherapy is increasingly being used in women with large cancers or LABC. Several large trials have assessed the efficacy of neoadjuvant therapy when compared to standard adjuvant chemotherapy. A meta-analysis of 11 neoadjuvant trials was performed by the Early Breast Cancer Trialists Collaborative Group

CONFIDENTIAL

[9]. Preliminary results from this meta-analysis were presented at the National Cancer Institute (NCI) neoadjuvant conference. Eleven randomized trials performed from 1981–1993, encompassing 4675 women, were included in the analysis. Preoperative therapy was associated with 18% fewer mastectomies and no significant difference in any breast cancer recurrence, breast cancer mortality, or death within 10 years of follow-up.

Two of these large randomized trials were undertaken by the National Surgical Adjuvant Breast and Bowel Project (NSABP) and provide the largest randomized data to date comparing preoperative to standard adjuvant chemotherapy. The NSABP B18 trial randomized 1523 women to either preoperative or postoperative doxorubicin/cyclophosphamide for a total of four cycles [10]. Breast tumor size was reduced in 80% of participants after preoperative therapy; 36% had a clinical complete response (cCR). The absolute pCR rate was 13%. Tumor size and clinical nodal status were independent predictors of cCR. Twenty-six percent of women with a cCR had a pCR. Clinical nodal response occurred in 89% of node-positive participants: 73% had a cCR and 44% of those had a pCR. There was a 37% increase in the incidence of pathologically negative nodes. Before randomization, lumpectomy was proposed for 86% of women with tumors ≤2 cm, 70% with tumors 2.1–5.0 cm, and 3% with tumors ≥5.1 cm. Clinical tumor size and nodal status influenced the physician's decision. Overall, 12% more lumpectomies were performed in the preoperative therapy group; in women with tumors ≥5.1 cm, there was a 175% increase. The NSABP-B27 trial was designed to determine the effects of adding docetaxel to preoperative doxorubicin and cyclophosphamide on breast cancer response rates and OS [11]. Women with operable breast cancer (n = 2411) were randomly assigned to receive preoperative doxorubicin and cyclophosphamide followed by surgery, doxorubicin and cyclophosphamide followed by docetaxel and surgery, or doxorubicin and cyclophosphamide followed by surgery and then docetaxel. Tamoxifen was initiated concurrently with chemotherapy. Median time on study for 2404 participants with follow-up was 77.9 months. Adding docetaxel to doxorubicin and cyclophosphamide did not significantly impact DFS or OS. There were trends toward improved DFS with the addition of docetaxel, which reduced the incidence of local recurrences as first events (p=0.0034). Preoperative, but not postoperative, docetaxel significantly improved DFS in participants who had a clinical partial response after doxorubicin and cyclophosphamide (hazard ratio = 0.71; 95% confidence interval (CI), 0.55–0.91; p=0.007). Thus, the primary benefit of neoadjuvant chemotherapy is to downstage tumors, thereby improving optimal surgical resection and increasing the probability of breast conservation [12].

However, an additional important finding in these studies was that the achievement of a pCR (*i.e.*, elimination of tumor in breast and axillary lymph nodes, as assessed at surgery) is a useful surrogate for prognosis in breast cancer participants overall, suggesting that chemotherapy sensitivity, in and of itself, is an independent predictor of DFS and OS. pCR, which occurred in 27% of participants in NSABP B-27, was doubled by addition of preoperative docetaxel, and was a significant predictor of OS regardless of treatment (hazard ratio = 0.33; 95% CI, 0.23–0.47; p<0.0001). Pathologic nodal status after chemotherapy was a significant predictor of OS (p <0.0001). The pCR rates of doxorubicin-containing regimens are in the range of 12%; combining doxorubicin and taxanes leads to pCR rates in the 25–27% range. However, the prognosis is still poor for those participants who present with very large tumors and those with significant residual disease in breast or lymph nodes [13–16]. Moreover, recent data that have incorporated molecular phenotyping into the classification of tumors undergoing neoadjuvant therapy have shown that response to treatment differs significantly by phenotype, as does the suitability of pCR as a useful surrogate [17]. Participants with strongly estrogen receptor-positive (ER+) tumors (those of the luminal A subtype) may have a low pCR rate, but subsequently have a favorable prognosis due to both the indolent natural history of the disease and the responsiveness of these tumors to anti-estrogen therapies. Conversely, many participants with triple-negative breast cancer (*i.e.*, ER/progesterone receptor (PgR)/HER2– by immunohistochemistry (IHC), or basal by molecular phenotyping) may have an

CONFIDENTIAL

excellent response to neoadjuvant chemotherapy, likely due to the high proliferative rate of these tumors. However, many of these participants subsequently relapse, and salvage strategies are lacking.

The optimal combination, sequencing, and schedule for neoadjuvant chemotherapy have not been established. In general, any regimen that is appropriate for *adjuvant* chemotherapy is also appropriate as a *neoadjuvant* regimen, and should be given in the same dose, combination, and schedule. For example, four cycles of doxorubicin/cyclophosphamide followed by paclitaxel is a common regimen for women with HER2/*neu* non-overexpressing LABC, and the addition of trastuzumab on a weekly schedule in combination with paclitaxel has been shown to be safe and effective [18]. However, several studies have examined variations to the standard regimen, with or without trastuzumab. The Eastern Cooperative Oncology Group (ECOG) 1193 trial compared doxorubicin followed by paclitaxel to the reverse sequence of paclitaxel followed by doxorubicin; no difference was observed in response rate [19]. ECOG subsequently performed a pilot trial in HER2 overexpressing early-stage breast cancer participants that added trastuzumab to weekly paclitaxel for 12 cycles followed by standard doxorubicin/cyclophosphamide (E2198). There was no safety signal suggesting increased cardiac or other toxicity with this sequence; the rate of clinical congestive heart failure was 3% [20]. Thus, clinical data to date suggest that the sequence of taxane and doxorubicin is of little importance. Interestingly, preclinical data have suggested that paclitaxel-induced inhibition of heat shock proteins and upregulation of topoisomerase II may lead to sensitization of tumor cells to doxorubicin [21].

2.2. The I-SPY TRIAL

The **I**nvestigation of **S**erial Studies to **P**redict **Y**our **T**herapeutic **R**esponse with **I**maging **A**nd mo**L**ecular analysis (I-SPY TRIAL) was designed to integrate clinical, laboratory and bioinformatics investigators in a new model to evaluate neoadjuvant chemotherapy in the setting of LABC—bringing together data from multiple molecular biomarker studies with imaging. The intent was to evaluate and develop biomarkers of early response to standard chemotherapy, and to develop a strategy to improve outcomes of women who do not have an optimal response to current standard therapy. The I-SPY TRIAL was designed as an inter-SPORE collaboration (NCI Specialized Programs of Research Excellence), with two cooperative clinical trial groups, the American College of Radiology Imaging Network (ACRIN), the Cancer and Leukemia Group B (CALGB), and the NCI Center for Biomedical Informatics and Information Technology (CBIIT) (protocols ACRIN 6657, CALGB 150007/150012). All participants received neoadjuvant chemotherapy to test a comprehensive panel of biomarkers, including MR imaging (MRI), for their ability to predict tumor response. Early endpoints being tested as predictors of three-year survival included MR changes (volume and longest diameter) and changes in gene expression. The intermediate endpoint was pCR (absence of invasive tumor in breast or lymph nodes at time of surgery), and the longer term endpoint was three-year RFS.

2.2.1. Results

Mechanics and demographics: Starting in the summer of 2002, 237 participants were enrolled in the I-SPY TRIAL. Of these, 216 successfully completed the trial and were considered eligible for evaluation. The participant population in I-SPY was diverse: 75% Caucasian, 19% African American, 4% Asian, and 2% other [22]. All treatment started with four cycles of doxorubicin; 95% of participants proceeded to a taxane regimen of four cycles. Serial biopsies, serum samples, and MR images were obtained prior to therapy, within two weeks of starting chemotherapy, between regimens, and at the time of surgical resection. We optimized methods of efficiently collecting and distributing tissue, and optimized the number and types of assays that could be performed. Expression array analysis was conducted on 149 participants on the Agilent 44K array, 118 participants on the Affymetrix U113A array, and 141 participants on a spotted complementary DNA (cDNA) array. Comparative genomic hybridization (CGH)

CONFIDENTIAL

arrays were conducted on 158 participants, and reverse-phase tissue protein lysate arrays on 149 participants[23]. Assay yield improved significantly by the end of the trial. Adding image guidance improved the yield of evaluable biopsies from 85% to 95%, depending on the type of imaging used [24].

Results: Of the 237 participants accrued in the I-SPY TRIAL, 215 participants had pathologic assessment available for analysis. RCB was calculated retrospectively on 201 participants (RCB calculator: http://www.mdanderson.org/breastcancer_RCB) [16]. RNA Agilent arrays were available on 149 participants. Participants that comprised the I-SPY population were biologically high risk, as defined by the Netherlands Cancer Institute 70-gene signature (NKI 70)-gene profile, with 91% of participants characterized as having a poor prognosis [22]. The mean tumor size was 6.0 cm and minimum size was 3.0 cm. The rate of pCR was 27%; 36% had RCB 0 or 1. Both pCR and RCB were predictive of three-year RFS with 3.9 years mean follow-up (p=0.04 and 0.01, respectively). HR and HER2/*neu* receptor (HER2) status were highly predictive of rates of pCR, ranging from 10% for HR+/HER2– to 50% for HR–/HER2–. The pCR rates for the intrinsic subtypes ranged from 2% to 50% as shown below in Table 2.1 [23].

Table 2.1 pCR, RCB, and Three-year RFS by Molecular Subtypes

IHC	Distribution (n = 194)	pCR (n = 188)	P-value	RCB (0 or 1) (n = 133)	P-value	3-yr RFS (n = 188)
HR+HER2-	48%	10%		21%		86%
HR+HER2+	12%	32%		48%		82%
HR-HER2+	12%	50%		79%		75%
HR-HER2-	28%	33%		41%		68%
**Gene Profiles**	**Distribution (n = 149)**	**pCR (n = 144)**	**P-value**	**RCB (0 or 1) (n = 133)**	**P-value**	**3-yr RFS (n = 144)**
**Intrinsic Subtypes**						
Luminal A	29%	2%		11%		97%
Luminal B	19%	15%		19%		80%
HER2-enriched	15%	52%		76%		90%
Basal	32%	34%		41%		59%
Normal-like	5%	43%	4.0 x 10-5	57%	7.5 x10-6	71%
**ROR-S**						
Low	26%	5%		19%		100%
Moderate	38%	22%		29%		80%
High	37%	40%	8.8 x 10-4	51%	0.0078	65%
**NKI**						
Good Outcome	9%	0%		18%		100%
Poor Outcome	91%	27%	0.038	37%	0.33	77%
**Wound Healing**						
Quiescent	23%	6%		30%		100%
Activated	77%	30%	0.0049	37%	0.52	73%
**p53 Mutation Gene signature**						
Wildtype	50%	11%		24%		93%
Mutation	50%	38%	3.7 x 10-4	46%	0.011	66%

The low-risk subsets had low rates of pCR and RCB, whereas high-risk subsets had high rates of pCR. DNA profiles showed similar findings in that high-risk subsets had high rates of pCR.

The most important finding is that pCR and RCB have very different abilities to predict outcome depending on the context of molecular profiles. For those participants showing favorable profiles, the outcome was good, regardless of RCB. For poor-risk profiles, pCR and especially RCB are predictive of

CONFIDENTIAL

outcome. This is true for the NKI 70-gene poor-risk profile, basal [24], wound healing (activated) signature (Figures 2.1, 2.2, and 2.3), and p53 mutation signature.

Figure 2.1: RCB as a Predictor of RFS Among NKI High-risk Participants

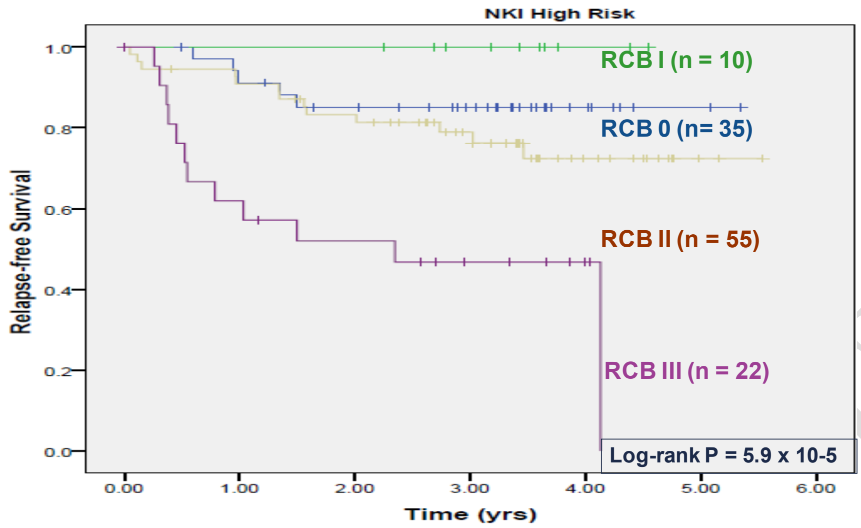

Figure 2.2: RCB as a Predictor of RFS Among Tumors with Wound Healing (Activated) Signature

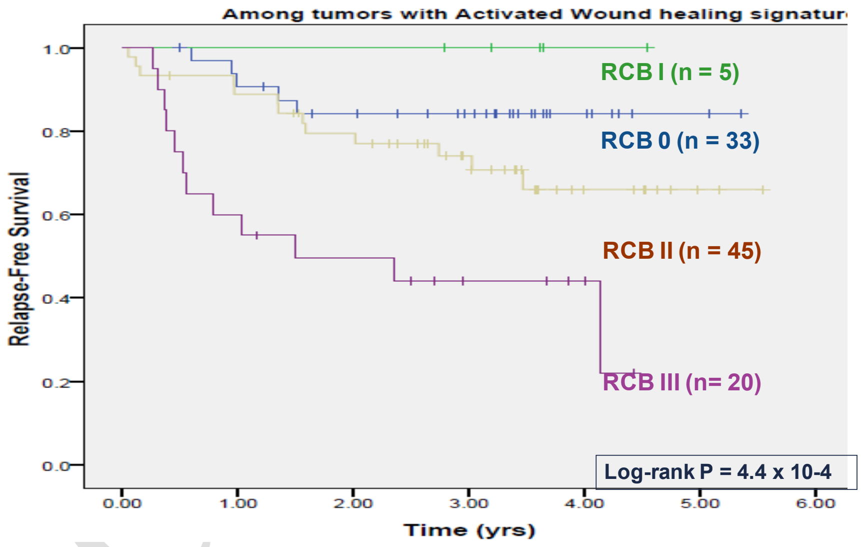

Figure 2.3: RCB as a Predictor of RFS Among Basal-like Tumors

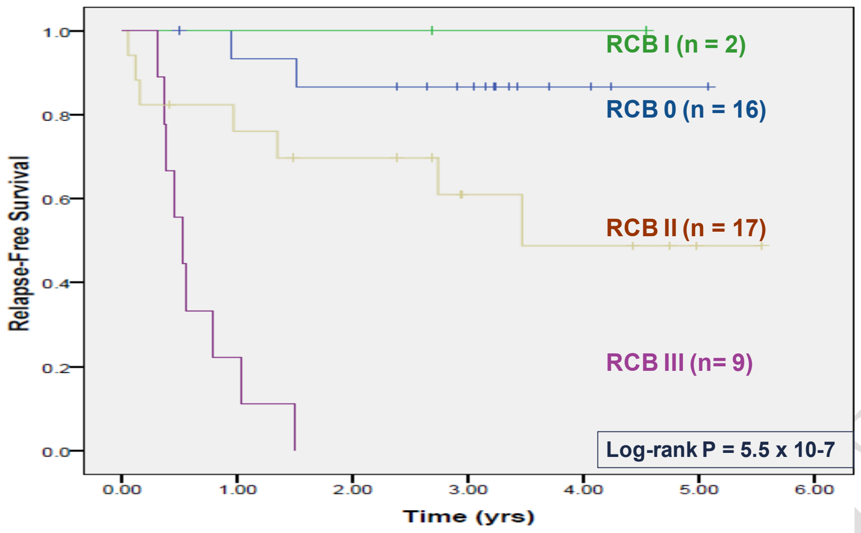

There was considerable variation in how the RNA signatures classified participants as low- and high-risk. A composite signature to identify robust low- and high-risk subsets was developed, assigning a score of 1 or –1 to signatures with two categories (wound healing activated vs. quiescent; NKI 70-gene signature low-risk vs. high-risk) and a score of 1, 0, or –1 when three categories were defined (risk of recurrence (ROR)-S low vs. medium vs. high). The low-risk category has excellent outcomes, regardless of RCB. Intermediate and poor-risk molecular profile categories have significantly worse outcomes (Figure 2.4) (Van't Veer and Das, submitted).

Figure 2.4: Integrated Scoring as a Predictor of RFS

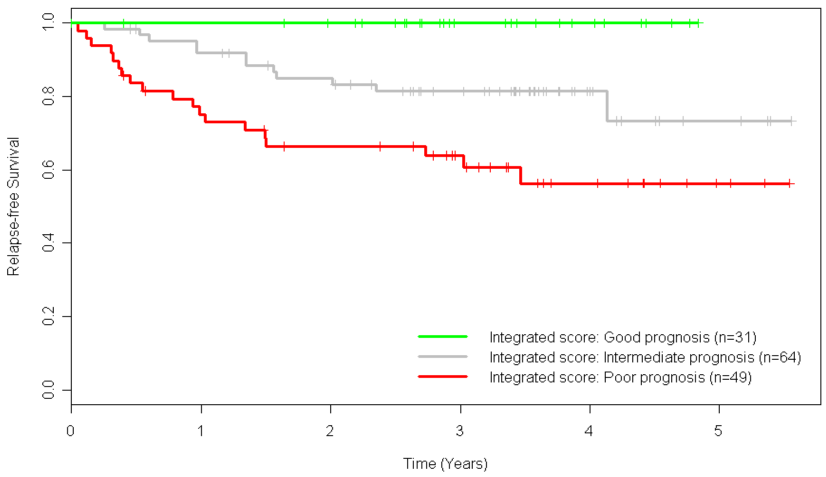

Figure 2.5: Integrated Score for Poor Prognosis Participants Associated with RCB

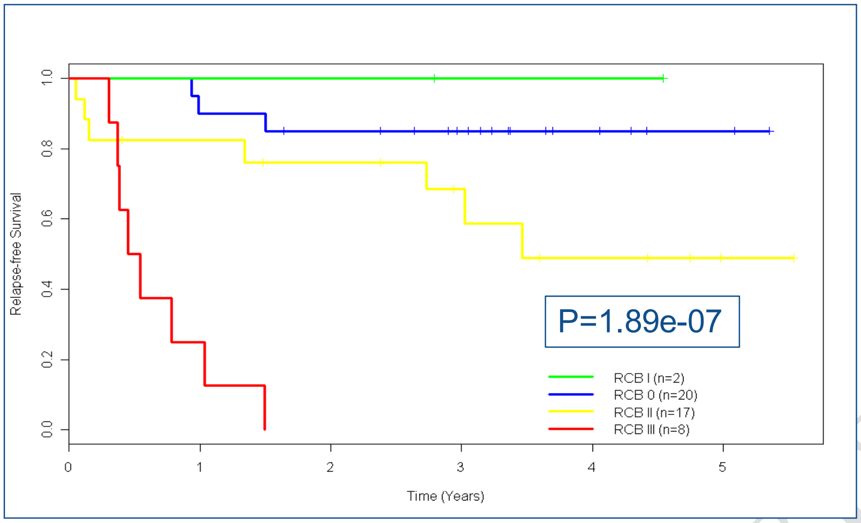

RCB in the poor-risk subsets is highly predictive of three-year RFS (Figure 2.5).

Given these data, we have elected to use the NKI 70-gene profile to exclude participants with a good prognosis. Those participants with a good prognosis by NKI 70-gene profile signature have an excellent outcome early on, and no participants with this profile had pCR. The selection of the poor-risk participants focuses on those participants in whom response to therapy is very predictive of outcome and who have a high risk for recurrence early in the course of their disease (Figure 2.5).

I-SPY 1 data show that improvement in pCR or RCB may be a rapid way to screen for the effectiveness of promising new targeted therapeutics. Moreover, it is anticipated that the most informative way to interpret the results will be by combining pCR and RCB evaluations with molecular subgroup analysis.

Of note, seven recurrences were reported among those who achieved pCR. Five of seven were HER2+ participants who were not treated with trastuzumab, since they were treated prior to the time that trastuzumab was clinically available in the adjuvant or neoadjuvant setting. An equivalent number of participants have now been given trastuzumab in the neoadjuvant setting, and there have subsequently been no early recurrences in the HER2+ group among participants with RCB = 0 or RCB = 1.

MRI volume is the imaging measure that best correlates with residual tumor in the breast, and change in MR volume (early and overall) is a strong predictor of pCR [25–27]. This supports the ACRIN 6657 (I-SPY TRIAL) hypothesis that MR volume can serve as a non-invasive measure of tumor burden and aid in evaluating response to therapy. Response to therapy was accurately captured by MR volume change among those participants who received trastuzumab in combination with taxane. Based on these data, we hypothesize that MR volume change can help us non-invasively determine response to investigational agents with chemotherapy.

The I-SPY TRIAL demonstrated that a collaborative group of investigators could effectively integrate biomarkers and imaging into the course of care by agreeing on standards for data collection, biomarker assessment, and MRI evaluations. The group also developed and shared methods to optimize assays; devised ways to obtain small amounts of frozen core biopsy material; agreed to use already established data-based tools for tissue tracking; as well as developed and utilized common platforms for information management and tissue repositories [2, 24, 28–30]. This robust infrastructure will be leveraged to support

CONFIDENTIAL

this trial as further described in the **TRAN**slational Informatics **S**ystem to **C**oordinate **E**merging Biomarkers, **N**ovel Agents and Clinical **D**ata (TRANSCEND) Users Manual.

I-SPY 1 Infrastructure: The I-SPY TRIAL was one of the first prospective trials in which all data from participating laboratories was submitted electronically to a common database and centrally housed at the NCI CBIIT using a Cancer Biomedical Informatics Grid (caBIG) tool, caINTEGRATOR. The purpose of caINTEGRATOR was to enable cross-platform comparison among participants and to increase our understanding of the molecular heterogeneity of LABC for all assays performed [31]. We implemented a new strategy for biomarker discovery and data sharing, enabling access to the data for all investigators to accelerate the pace of learning. Once accrual of participants and samples was complete, outside investigators could submit requests to the I-SPY TRIAL Publications Committee (for samples or data) to conduct additional analyses and discoveries. All investigators were required to submit their data to caINTEGRATOR, thereby continuing the growth and enrichment of the data set. Over the course of three and a half years, 10 clinical centers accrued the participants. To date, over 15 laboratories have contributed data. Currently, clinical and laboratory data are submitted to caINTEGRATOR. The collection and aggregation of the data has enabled us to build upon our knowledge base as well as use novel analytical tools such as the Cancer Genome Browser [32].

2.3 Rationale: Neoadjuvant Adaptive Design Approach

Studies of neoadjuvant chemotherapy in Stage II and III breast cancer participants suggest that some have significant benefit from chemotherapy while others appear to derive much less value. Because breast cancer is a genetically and clinically heterogeneous disease, the ability to identify markers that predict early responders to standard chemotherapy and long-term survival would markedly improve the breast cancer treatment paradigm. A variety of histopathologic, genomic, proteomic, and imaging strategies have the potential to predict response to standard therapy and to provide a framework for testing investigational-targeted agents in the context of unique molecular subtypes of breast cancer. Rational matching of investigational agents with cohorts of participants whose disease characteristics suggest they might benefit from this “personalized” therapy requires an understanding of fundamental regulatory pathways that control breast cancer pathology as well as development of validated assay methods to reproducibly identify tissue or serum markers that predict response. The neoadjuvant setting provides the perfect opportunity to target agents to the biology of specific signatures and to rapidly assess the impact of these agents during the course of chemotherapeutic exposure, with confirmation of response at the time of surgical excision. Further, integrating imaging enables a non-invasive way of measuring response and accelerating learning about specific response to treatment.

The infrastructure of the I-SPY TRIAL enables us to take an important step toward systematically assigning phase 2 agents and rapidly learning about the impact of these agents on participants based on specific molecular characteristics (signatures) of their tumors. As stated previously, women who present with LABC are at high risk for recurrence; however, unlike women with metastatic disease, they are still potentially curable. A large number of phase 2 biologically targeted therapies need to be efficiently evaluated. Fortunately, most of them do not appear to be toxic, even in combination with chemotherapy. Further, emerging data suggests that these agents will be most efficacious in combination with chemotherapy. It is absolutely critical that we shorten the knowledge turns and lifecycle for evaluating new agents [33], and get the most promising agents to those most likely to benefit. Introducing these agents in the neoadjuvant setting, where we have a short-term (six-month) intermediate endpoint to assess efficacy, will provide the proper time horizon for agent evaluation. Furthermore, the introduction of these agents to women with curable high-risk disease carries the promise of improving survival rates in women most at risk of death due to their disease. However, the classic method of randomized trials evaluating one agent at a time for a set number of individuals is still inefficient, and will not allow us to rapidly learn

CONFIDENTIAL

for whom the new agents are most effective. To address this problem, the I-SPY 2 TRIAL team, in collaboration with the NCI, Food and Drug Administration (FDA), and the Foundation for the National Institutes of Health (FNIH) Biomarker Consortium, collaborated to design an adaptive randomized phase 2 trial based on biomarker signatures where multiple agent classes can be evaluated simultaneously on a backbone of molecularly profiled participants. The trial process we present is explicitly intended to eliminate some of the enormous inefficiencies in our current trial designs.

Using Bayesian methods of adaptive randomization [34], agents will be assigned to participants who have higher probability of efficacy. Therefore, agents which show the appropriate beneficial changes within a specific molecular signature will be preferentially assigned within that signature and move through the trial more rapidly. Agents that do not show the likelihood of improved pCR rate in any predefined biomarker signature will be dropped from the trial. Each agent's Bayesian predictive probability [34] of being successful in a phase 3 confirmatory trial will be calculated for each possible signature. Agents will be dropped from the trial for futility when this probability drops sufficiently low for all signatures. Agents will be graduated at an interim point should this probability reach a sufficient level for one or more signatures. Those agents with high Bayesian predictive probability of being more effective than standard therapy will graduate along with their corresponding biomarker signatures, allowing these agent-biomarker combinations to be tested in smaller phase 3 trials. At graduation, an agent's predictive probability will be provided to its sponsoring company for all signatures tested. Depending on participant accrual rates, new agents can be added at any time during the trial to replace the agents that are dropped or graduated.

The Biomarkers Consortium of FNIH supported the development plan for this adaptive design protocol in breast cancer based on promising results to date of the I-SPY TRIAL. FNIH was established by the US Congress to support the mission of the National Institutes of Health (NIH). The mission of FNIH is to foster public health through scientific discovery, translational research, and dissemination of research results through specially configured, high-impact public-private partnerships consistent with NIH priorities. The Biomarkers Consortium is a collaborative public-private partnership managed by FNIH in an effort to create fundamental change in how healthcare research and medical product developments are conducted. This expectation is being accomplished by bringing together leaders from biotechnology and pharmaceutical industries, government, academia, and non-profit organizations to work together to accelerate identification, development, and regulatory acceptance of biomarkers.

2.4 Study Agent Rationale

The I-SPY 2 TRIAL investigational agents are described in appendices D–x. As investigational agents are added to the trial, they will appear as subsequent appendices.

2.4.1 Agent Selection

The initial process of candidate agent review began with the I-SPY 2 Agent Selection Working Group. This group consisted of I-SPY oncologists, phase 1 and 2 trialists, and interested industry representatives, who generated an initial list of agents that would be potentially appropriate for I-SPY 2. The list included agents targeting biologic pathways thought to be upregulated in breast cancer, including HER2 (*e.g.,* HER2 monoclonal cytotoxin conjugates, pan-Erb2 inhibitors), insulin-like growth factor (IGF)-1 receptor, phosphatidylinositol-3-kinase (PI3K), mTOR, cMET, apoptosis (BCL-2 inhibitors), angiogenesis inhibitors, DNA damage repair mechanisms (polyadenosine diphosphate ribose polymerase [PARP] inhibitors), and the death receptor pathway (Apo2L/TRAIL agonists). The list was evaluated in a series of teleconferences during 2008 and the agents under development were reviewed for their potential to improve breast cancer outcomes. From an initial nominated list of >65 agents within at least 10 major

CONFIDENTIAL

molecular pathway target classes, 20 priority agents were selected for further detailed assessment. A summary list of the agents, their targets, and molecular signature targets (*e.g.*, HER2+ *vs.* HER2–) was made available in a follow-up meeting with additional pharmaceutical company representatives, and further comments on the selections were invited. Literature reviews were prepared and current development status details, including efficacy and safety information and ongoing or planned clinical trial data, were solicited from the relevant pharmaceutical companies. Data were assembled into worksheets on each agent. First consideration was given to agents that had completed phase 1 safety testing in combination with a taxane, and clinical evidence or preclinical rationale for activity against breast cancer. The non-industry members of the Agent Selection Working Group conducted a complete review and the committee members recommended to either Approve (high, medium or low priority) or Reject (secondary to safety) the selections. These agents were then sent to the Independent Agent Selection Committee (IASC).

Final prioritization of Tier 1 agents is made by the IASC, whose members come from industry, academia, research institutions, foundations, and patient advocacy groups not directly involved in the trial. An agreement at the outset of the design process was made to test or select only one agent within a therapeutic class (*e.g.*, only one IGF receptor (IGFR)-1 antibody-based inhibitor will be tested).

2.4.2 Designation of Tier 1 *versus* Tier 2 Agents for the I-SPY 2 TRIAL Process

Over the course of the trial, agents will be dropped or graduated and new agents will be needed to replace them. For this reason, agents need to be qualified for the trial over the course of the study period. Those agents ready for use when the trial opens are designated as Tier 1 agents. Tier 1 agents must have appropriate safety data alone and in combination with a taxane. Tier 2 agents are promising agents in the process of going through phase 1 testing alone or in combination with taxanes that will be evaluated for appropriateness during the course of the trial as data in combination with paclitaxel become available.

2.4.3 I-SPY 2 Investigational Tier 1 Agents

The Tier 1 agents being tested include agents that target HER2, IGF-IR, angiogenesis pathways, DNA damage repair mechanisms, and death receptors. After each agent, the signature being targeted is listed (See Appendix C–x). For HER2 targeted agents or agent combinations, if there are phase 2 data suggesting efficacy equal to or greater to that of trastuzumab or a combination of paclitaxel plus trastuzumab, the investigational agent or agent combination will be used in place of trastuzumab or paclitaxel plus trastuzumab; without such data, agents or agent combinations will be used in addition to paclitaxel plus trastuzumab.

For each Tier 1 agent, we have organized the presentation of data to include:

- MECHANISM
- IN VITRO STUDIES
- ANIMAL STUDIES
- HUMAN STUDIES, including phase 1 data and phase 1 data in combination with taxanes
- ONGOING STUDIES
- TOXICITY and SAFETY
- PHARMACOKINETICS

Each Tier 1 agent has an assigned Chaperone and co-Chaperone. Chaperones are assigned by the Agent Selection Working Group chairs, and are selected based on their preclinical and/or clinical experience with a specific pathway and/or agent. Chaperones are responsible for overseeing the agent/agent

CONFIDENTIAL

combination within the trial (*i.e*., participate in the development and maintenance of the agent appendix and overall safety of the participants receiving the agent).

Please refer to Appendices D–x for detailed information for the I-SPY 2 tier 1 investigational study agents.

Example: Agent X, Specifications for Use

Target:	e.g*., HER2, angiogenesis pathway, etc.*
Signature:	e.g*., HER2+, ER+, – – –*
Schedule:	*Given weekly with paclitaxel*
Trastuzumab:	e.g*., Used in place of trastuzumab or in addition to trastuzumab*
Manufacturer:	*List pharmaceutical manufacturer*

2.5 General Approach in Evaluating Agent(s) and Biomarkers

I-SPY 2 TRIAL will examine the efficacy of at least ten investigational agents/agent combinations in women with locally advanced Stage II or III breast cancer. The randomization and agent assignments will be based on the MammaPrint 44K Array, HR, and HER2 status. Each participant randomized to an experimental arm will be assigned to one investigational agent (plus paclitaxel/trastuzumab) until protocol completion or removal; re-randomization is not planned under the current proposed design. An important objective of the study is to identify MR imaging and molecular characteristics predictive of pCR and survival in these participants. The goal is to determine an optimal biomarker profile for each experimental regimen being considered, and to graduate these regimens from the trial into phase 3 pivotal studies. Regimens will be dropped for a specific profile if they are not sufficiently effective from that profile.

The Master Investigational New Drug (IND) application will be amended to include additional investigational agents/agent combinations as updated safety and efficacy information becomes available and initial treatment groups are dropped for futility or graduated. Investigational Device Exemptions (IDEs) for Agendia's MammaPrint 44K Array, Agendia's TargetPrint HER2 44K Array, and for Hologic's MR volume Aegis software are included as part of the Master IND. Additional biomarker assessment methods proposed for qualification include protein assays such as IHC/fluorescence *in situ* hybridization (FISH) as well as reverse phase protein microarrays (RPMA) specific for targeted pathways (to identify pathways driving participant's tumor); mRNA array assay for agent and prognostic predictors; and Affymetrix or Agilent gene expression arrays (*e.g.*, paclitaxel (Taxol®), doxorubicin (Adriamycin™) and cyclophosphamide (TFAC) RCB) for prediction of response. Under the proposed study plan, IDEs for these biomarkers may also be prepared for submission to the Office of *In Vitro* Diagnostics (OIVD) as the data are generated. Other possible exploratory biomarkers include DNA methylation, DNA sequencing, genome-wide association studies (GWAS), pharmacogenomics, and microRNAs, as well as blood/serum/plasma/cell-based assays evaluating tumor cell or proteins in circulation.

Access to I-SPY 2 TRIAL data and biospecimen repository is governed by the I-SPY 2 Data Access and Publications Committee (DAPC). Researchers interested in obtaining access to the I-SPY 2 dataset for analysis should submit a completed concept sheet to the DAPC. Concept sheets can be obtained at www.ispy2trial.org. Those researchers interested in evaluating a biomarker platform in the I-SPY 2 TRIAL will be designated the Platform Chaperone once their concept sheet is approved by the DAPC. The Platform Chaperone will have continued involvement with other I-SPY 2 researchers interested in utilizing their platform; however, the Platform Chaperone will not own the data obtained by the other I-SPY 2 researchers. Requests for biospecimens are sent to the I-SPY 2 Biomarker Committee who will review and recommend requests for biospecimens to the I-SPY 2 DAPC for final approval.

CONFIDENTIAL

3. SUMMARY OF STUDY PLAN

3.1 Screening Phase

I-SPY 2 TRIAL is a neoadjuvant trial making use of adaptive design to identify successful treatment regimens for Stage II/III breast cancer. Women with ≥2.5 cm invasive breast cancer by palpation or imaging are eligible for study screening (see Schema, Figures A and B). Tumor ER and PgR status, and HER2 by community IHC and/or FISH conducted at a local laboratory, will be done as part of a routine diagnostic work-up. After the participant consents to be screened, a core biopsy will be performed, and sections will be sent to Agendia for MammaPrint score and TargetPrint HER2 gene expression assay using the Agendia 44K full genome microarray (MammaPrint 44K Array Low and High scores are determined per FDA label for the cleared MammaPrint device). A tumor will be considered HER2+ if any one of the three assays (IHC, FISH, TargetPrint) is positive (as defined in §7.3). Participants will also undergo a pretreatment MRI for determination of maximum tumor dimension.

Women who are low risk by MammaPrint 44K Array and also ER+ and HER2– will be excluded from the trial as shown in Table 3.1.

Table 3.1 Table of Eligibility for Randomization

	MammaPrint Low*		MammaPrint High*	
	ER+	ER–	ER+	ER–
HER2+	Eligible	Eligible	Eligible	Eligible
HER2–	Not Eligible	Eligible	Eligible	Eligible

*MammaPrint 44K Array Low and High are determined per FDA label for the cleared MammaPrint device

NOTE: Participants not eligible to participate in the treatment phase of I-SPY 2 because they are ER+, HER2-, MammaPrint Low are eligible to participate in the Low-risk Registry Trial (see Supplement 1).

Participants eligible for randomization will be recategorized according to biomarker profiles shown in Table 3.2. A participant's ER and PgR status are used to determine their HR status. For instance, ER+ and PgR– is HR+; ER– and PgR+ is HR+. Additionally, a participant's MammaPrint 44K Array Low/High score is adjusted to either the MammaPrint High1 or High2 class. MammaPrint High1 (MP–) and High2 (MP+) classes were determined by the pre-defined median cut-point of I-SPY 1 participants who fit the eligibility criteria of I-SPY 2.

Table 3.2 Biomarker Profiles for Treatment

	**MammaPrint High1***		**MammaPrint High2***	
	HR+	HR–	HR+	HR–
HER2+				
HER2–				

*MammaPrint High1 and MammaPrint High2 are determined by the predefined median cut-point of I-SPY 1 participants who fit the eligibility criteria of I-SPY 2.

CONFIDENTIAL

These biomarker profiles are used for randomizing each participant to a treatment arm (see Schema, Figure D). For every participant that is randomized, there is a 20% chance the participant will be randomized to the control arm (paclitaxel or paclitaxel plus trastuzumab if HER2+), regardless of how many investigational agents are in the study. Each investigational agent will have an initial biomarker signature that will determine which participants will be randomized to that investigational agent. A biomarker signature as predefined by the agent manufacturer can range from a maximum of all participants to a more limited signature (see §13.2). For example, participants with tumors considered to be HER2+ will receive paclitaxel plus trastuzumab (Herceptin) as part of the control arm, and HER2-directed agents in the experimental arm, where they will receive paclitaxel plus trastuzumab plus new agent A; paclitaxel plus trastuzumab plus new agent B; or paclitaxel plus trastuzumab plus new agent C. If a new agent being considered has phase 2 data showing equivalent or improved efficacy to trastuzumab or paclitaxel plus trastuzumab, the new agent will replace trastuzumab or paclitaxel plus trastuzumab in the experimental arm. The HER2– group will be randomized to receive either weekly paclitaxel alone or weekly paclitaxel plus new agent C; paclitaxel plus new agent D; or paclitaxel plus new agent E.

Due to the recent accelerated approval of pertuzumab in the neoadjuvant setting, the Her2+ control arm (paclitaxel plus trastuzumab) has been temporarily closed in accordance with the recommendations of the I-SPY 2 DSMB and Investigators.

3.2 Treatment Phase

Investigational agents will be given in 12 weekly intervals or at other intervals over a 12-week period. After a participant completes three weekly cycles or one three-week cycle of therapy, she will undergo a repeat MRI, core biopsy of the tumor, and blood draw. She will continue treatment for nine more weekly cycles (or nine weeks for a total of 12), and undergo a third MRI and blood draw. She will then receive four cycles of doxorubicin and cyclophosphamide at two- or three-week intervals prior to surgery. The participant will have an MRI and blood draw prior to surgery, and tumor tissue will be collected at surgery. The primary endpoint is pCR (defined as absence of invasive tumor in breast or lymph nodes at the completion of all neoadjuvant chemotherapy). A more complex and detailed pathologic evaluation, RCB [16], will be used to evaluate surgical specimens. RCB is estimated from routine pathologic sections of the primary breast tumor site and the regional lymph nodes after completion of neoadjuvant therapy. Six variables are included in a formula including tumor bed size, cellularity, and extent of the disease in the breast and nodes; it is calculated using automated software (www.mdanderson.org/breastcancer_RCB). RCB is potentially a better predictor of five-year RFS [16].

3.3 Biomarkers

MRI will be used as a noninvasive serial measurement of response during the course of treatment; MRI longest diameter and volume will be measured at each time point and will be used for early evaluation of response. MRI volume measured just prior to surgical resection has been correlated with residual tumor; change in MRI volume was well correlated with pCR in I-SPY 1. MRI volume is an automated measurement which will be obtained directly from the MRI workstation using a CAD software system by Hologic. As part of a joint R-01-Small Business Innovation Research Grant under the auspices of Dr. Nola Hylton (Principal Investigator, ACRIN 6657, I-SPY TRIAL), MR software workstations will be placed at all participating I-SPY 2 sites.

Peripheral blood samples will be collected pretreatment, early in paclitaxel treatment (end of week 3), inter-regimen, and presurgery. Tissue samples will be collected from core biopsies done pretreatment and early in paclitaxel treatment (end of week 3), and surgical tissue will be collected at the time of surgery if sufficient tumor remains. Tissue obtained during the screening phase of the trial will be used to generate

CONFIDENTIAL

molecular profiles for initial randomization assignment and to generate further qualifying biomarkers, including gene and protein measurements by Agilent 44K and Affymetrix arrays, mRNA arrays, and RPMA assays. These molecular characteristics (biomarkers/pathways) will be used to correlate with pathologic, imaging, or RCB response measures in the neoadjuvant setting with the investigational therapeutic agents. Tissue and blood will be used for exploratory research and to generate molecular data on next-generation technology platforms. Finally, participants will be followed post surgery for five years for recurrence-free and overall survival.

Over the course of the trial, additional qualifying biomarkers will be put forward and tested for their ability to predict tumor response to specific classes of investigational targeted therapeutics, and exploratory biomarkers that have future promise for better stratifying tumor type and response will also be incorporated into the trial (see §7 for details).

3.4 Adaptive Randomization of Investigational Agents

Agents will be assigned to all signatures where they might be effective. The control arm will apply to all profiles. Randomization probabilities are determined based on the accumulating data about all agents in the trial. The trial is designed to study over time which profiles predict response to each agent. Each agent's probability of being successful in a phase 3 confirmatory trial will be calculated:

- Agents will be graduated at an interim point should one or more of these probabilities reach a sufficient level.
- Agents will be dropped from the trial for futility when probabilities drop sufficiently low.

If the maximum sample size of 120 participants assigned to a regimen (over all biomarker types) is reached, assignments to the regimen will end. (With the exception if assignment of a regimen is to be restricted to patients with tumors that are either HER2+ or HER2– then the maximum total sample size for that regimen is 75.)

If an investigational agent reaches a threshold for graduation, the Data Safety Monitoring Board (DSMB) will review the findings and make a recommendation to Study Principal Investigators (PIs) for final approval. During the review by the DSMB and PIs, participants will continue to be randomized to the regimen or agent. Once the agent graduates, no additional participants will be randomized to that agent. Participants currently receiving that agent will continue on the regimen until they complete the entire course of treatment.

If the maximum sample size of 120 participants (75 patients if a regimen is restricted to patients with HER2+ or HER2-) is reached, no additional participants will be randomized to that agent. Participants currently receiving the agent will continue on the regimen until they complete the entire course of treatment.

If an agent is found not to reach a specified threshold of improvement in response, it may be dropped for futility; the DSMB will review the findings, and if they agree, will recommend to the PIs that the agent be dropped from the trial. During review by the DSMB and PIs, no participants will be randomized to that regimen or agent. Participants who have not completed the course of the agent will continue to receive the agent until a determination is made. Once an agent is dropped from the trial, the option to continue or drop the agent will be at the discretion of the participant and her treating physician. Participants who do not continue on the agent will continue on-study but will revert to the standard/control regimen; their outcomes will remain part of the arm to which they were assigned.

CONFIDENTIAL

If an investigational agent is removed from the trial due to serious side effects from the agent, use of that agent for all participants will be stopped. Participants will continue on-study but will revert to the standard/control regimen and their outcomes will remain part of the arm to which they were randomized.

The above assignment and stopping rules for randomization apply to all regimens in the trial irrespective of study entry.

Up to eight investigational agents will be active at any given time; the first set of agents is currently being selected. The number of agents considered will be restricted by the ability to "process" the trial agents expeditiously in order to give companies timely information concerning the potential role of the agent in treating breast cancer. Trial data will also be used to test, qualify, and validate biomarkers as predictors of response to specific therapeutic agents. This trial is an opportunity to integrate information from emerging biomarkers and thereby accelerate identification of optimal therapies for women at highest risk of progression.

Randomization will be adaptive to maximize information about better-performing therapies and minimize the time it takes to identify optimal biomarker profiles. Using MRI results as a biomarker during various stages of treatment, our design will build a longitudinal model of tumor response. Such a model is critical for the adaptive aspect of the trial to enable early assessment of therapeutic benefit. The number of participants required to evaluate each investigational agent will range from a of 20 participants to a maximum of 120 (75, if assignment of a regimen is restricted to patients with tumors that are either HER2+ or HER2–).
We anticipate evaluating at least 10 investigational agents or combinations of agents over the course of the I-SPY 2 TRIAL process.

3.5 Trial Informatics

The bioinformatics system (caBIG-based collaborative interface) developed and applied by the I-SPY TRIAL will continue. The infrastructure put in place from the I-SPY TRIAL study consortium and NCI is intact and includes web-based participant registration and randomization as well as data sharing and analysis. The adaptive trial software bundle developed as part of the TRANSCEND project includes software for tissue tracking using caTISSUE, the caBIG biospecimen management system, among others. After qualifying to participate in the I-SPY 2 TRIAL process, all sites are trained to use the software tools (available at www.ispy2web.org) and provided a user manual (see TRANSCEND User Manual).

CONFIDENTIAL

4. PARTICIPANT SELECTION

4.1 Inclusion Criteria Overview

The I-SPY 2 TRIAL will enroll women through a two-stage process that includes the initial screening phase followed by the treatment phase for those women eligible for enrollment. Separate consent forms will be used for each phase, and eligibility for each phase is detailed below.

4.1.1 Eligibility Criteria for Initial Screening Phase of I-SPY 2 TRIAL

A. **Histologically confirmed** invasive cancer of the female breast. Histologic confirmation can be obtained by fine-needle aspiration (FNA), core needle biopsy, or incisional biopsy (allowed if residual tumor is 2.5cm). Metaplastic and inflammatory carcinomas are eligible, and synchronous bilateral primaries are eligible if the more advanced tumor meets staging criteria. Participants who have an FNA for diagnosis must have histological documentation of invasive carcinoma by the start of chemotherapy.

B. **Clinically or radiologically measureable** disease in the breast after diagnostic biopsy, defined as longest diameter greater than or equal to 25 mm (2.5 cm).If a tumor meets this criteria by clinical exam only, the tumor must also be accurately measured in at least one dimension (longest diameter to be recorded) as >20 mm (2 cm) with conventional techniques (positron emission tomography (PET), computed tomography (CT), MRI, ultrasound, or x-ray) or as >10 mm (1 cm) with spiral CT scan. All tumor measurements must be recorded in metric notation.

C. **Prior therapy**: No prior cytotoxic regimens are allowed for this malignancy. Participants may not have had prior chemotherapy, other targeted anticancer therapies, or prior radiation therapy to the ipsilateral breast for this malignancy. Prior bis-phosphonate therapy is allowed.

D. **Age ≥18 years**: Because no dosing or AE data are currently available on use of experimental trial agents for participants <18 years of age, children are excluded from this study.

E. **Performance status:** ECOG performance status 0−1.

F. **Core biopsy:** Willing and able to undergo core biopsy of the primary breast lesion to assess baseline biomarkers to determine eligibility for treatment phase of I-SPY 2 TRIAL.

G. **Nonpregnant and non-breastfeeding:** Effects on a developing human fetus of phase 2 agents under study at the recommended therapeutic dose are unknown. For this reason and because these agents may be teratogenic, women of child-bearing potential must agree to use adequate contraception (double barrier methods of birth control or abstinence) prior to study entry and for the duration of study treatment phase. Should a woman become pregnant or suspect she is pregnant while participating in this study, she should inform her study physician immediately.

   If a participant is of child-bearing potential (women are considered not of childbearing potential if they are at least one year postmenopausal and/or surgically sterile), she must have documented negative serum or negative urine pregnancy tests within 14 days of entry to screening phase.

CONFIDENTIAL

H. **No ferromagnetic prostheses**: Participants who have metallic surgical implants that are not compatible with an MRI machine are not eligible. Otherwise eligible participants should be asked if they have any heart valves, aneurysm clips, orthopedic prosthesis, or any metallic fragments anywhere in their body prior to enrolling in the study.

I. **Ability to understand and willingness to sign** a written informed consent document (I-SPY 2 TRIAL Screening Consent).

4.1.2 Inclusion Criteria for Treatment Phase of I-SPY 2 TRIAL

Participants successfully enrolled on the screening phase of I-SPY 2 TRIAL will be evaluated for eligibility for the treatment phase of I-SPY 2 based on the results of several tumor biomarker assays. In addition to the eligibility criteria outlined in §4.2, participants who have completed the Initial Screening phase must meet the following eligibility criteria:

A. **Eligible breast tumors** must also meet one of the following criteria:

- Stage II or III
- T4, any N, M0, including clinical or pathologic inflammatory cancer
- Regional Stage IV, where supraclavicular lymph nodes are the only sites of metastasis, will be evaluated at the time of surgery.

B. **Breast Hormone status:** Any tumor ER/PgR status, any HER2/*neu* status as measured by local hospital pathology laboratory, and meets any tumor assay profile described in 4.1.2 F.
Tumors will be considered positive when:
- ≥5% tumor staining for ER and/or PgR is seen.
- Any one of the following three conditions for HER2 are met:
  - IHC 3+;
  - Overexpression by FISH (as defined by FDA-cleared/approved tests used at each institution). When an increase in CEP17 copy number is observed by FISH (*i.e.*, polysomy), the participant will be considered HER2+ if the ratio of HER2 signals/nucleus is greater than 6;
  - TargetPrint HER2+.

C. **Normal organ and marrow function** as defined below (test results can be used if done within 30 days of consenting to treatment phase):

- Leukocytes ≥3000/μL
- Absolute neutrophil count ≥1500/μL
- Platelets ≥100,000/μL
- Total bilirubin within normal institutional limits, unless participant has Gilbert's disease, for which bilirubin must be ≤2.0 × ULN
- Aspartate aminotransferase (AST) (SGOT) or alanine transaminase (ALT) (SGPT) ≤1.5 × institutional ULN
- Creatinine <1.5 × institutional ULN
- For agent-specific criteria, see Appendix C §2.

D. **No uncontrolled or severe cardiac disease** (history of diagnosis of unstable angina, myocardial infarction, symptomatic congestive heart failure, serious uncontrolled cardiac

CONFIDENTIAL

arrhythmia [including atrial flutter/fibrillation], requirement for inotropic support or use of devices for cardiac conditions [pacemakers/defibrillators]). Baseline ejection fraction (by nuclear imaging or echocardiography) must be ≥50%.

E. **No clinical or imaging evidence of distant metastases by** CT with or without PET, PA and lateral chest radiograph (CXR), radionuclide bone scan, and/or LFTs including total bilirubin, ALT, and AST within ranges defined in §4.1.2 C(test results can be used if done within 30 days of consenting to treatment phase).

F. **Breast tumor assay profile** must include one of the following:

- MammaPrint High, any ER status, any HER2 status
- MammaPrint Low, ER– (<5%), any HER2 status
- MammaPrint Low, ER+, HER2/*neu* positive by any one of the three methods used (IHC, FISH, TargetPrint). See §3, Table of Eligibility

G. **Ability to understand and willingness to sign** a written informed consent document (I-SPY 2 TRIAL Treatment Consent)

H. **All additional applicable investigational agent-specific eligibility criteria can be found in §2 of Appendix C. Each investigational agent-specific eligibility criteria can also be found in §2.2 of each investigational agent-specific appendix**.

4.2 Exclusion Criteria

A. **Use of any other investigational agents** within 30 days of starting study treatment.

B. **History of allergic reactions** attributed to compounds of similar chemical or biologic composition to Study Agent or accompanying supportive medications.

C. **Uncontrolled intercurrent illness** including, but not limited to, ongoing or active infection, diabetes, or psychiatric illness/social situations that would limit compliance with study requirements.

D. **Sentinel lymph node dissection/biopsy** on the nodes draining from the study index tumor site is not allowable prior to the start of chemotherapy. Any participant who has undergone sentinel lymph node dissection/biopsy procedure on the side of the study index tumor prior to start of chemotherapy is not eligible. Clinical evaluation of the axilla and FNA and/or core biopsy of any suspicious nodes detected clinically or radiologically should be performed prior to starting chemotherapy.

4.3 Inclusion of Women and Minorities

This study will be carried out in women. In the I-SPY 1 TRIAL, there was 19% participation of African American women, 4% Asian, and 2% other. If, during the initial phases of accrual, this distribution is not achieved, the study will specifically recruit participants through the usual channels for medical center research participant advertising (newsletter, posters) to achieve the desired participant mix. The following information will be reported in compliance with FDA annual reporting requirements.

CONFIDENTIAL

4.4 Recruitment and Retention Plan

Participant eligibility will be systematically assessed at each of the participating I-SPY 2 study sites. A screening log will be kept in TRANSCEND documenting the review of potentially eligible participants as well as reasons for non-enrollment. Sites will provide detailed information to all relevant treating physicians on the conduct of the trial to optimize physician participation. Monthly conference calls will review recruitment at each site so that sites not meeting recruitment goals can be identified early and interventions to improve recruitment can be instituted.

The I-SPY 2 TRIAL is listed on the NIH website Clinicaltrials.gov to enable referring physicians to identify local sites for participant referral (NCT01042379). Participants will also be able to find the I SPY-2 TRIAL sites through breastcancertrials.org, a clinical trial matching web site. Women diagnosed with breast cancer can go to this national-service web site and enter their information to find clinical trials appropriate for them. Once they find a trial, they can contact a research site and send their information through TRIAL CONNECT, which includes their contact information and their eligibility screened against the trial eligibility. In addition, the study will work with advocate groups across the country to improve awareness.

A large, organized cadre of experienced participant advocates will participate within community participant support services locations to help educate participants about the trial and assist in the recruitment and retention process. These advocates will be experts on the trial design and conduct and will assist potentially eligible participants in understanding the informed consent process as well as assisting those enrolled in navigating the various steps within the trial assessment and treatment process.

CONFIDENTIAL

5. CHEMOTHERAPY ADMINISTRATION

5.1 Standard Chemotherapy Treatment Plan for Control Arm, HER2– Tumors

Participants randomized to the standard chemotherapy treatment arm who are HER2– will receive 12 cycles of paclitaxel at 80 mg/m^2 once every seven days (q1w) ± 1 day. For paclitaxel, Filgrastim can be used at investigators discretion. A minimum of seven days after completing the paclitaxel regimen, participants will receive four cycles of doxorubicin at 60 mg/m^2 plus cyclophosphamide at 600 mg/m^2 once every 14 days (q2w) ± 1 day or once every 21 days (q3w) ± 1 day at physician discretion. For AC, Filgrastim or Pegfilgrastim can be used at investigator's discretion. **All treatment doses should be based on actual body weight and not ideal body weight. If participant's body weight increases or decreases by ≥10% from baseline during the course of the treatment phase, the body surface area and agent dose should be recalculated.**

Table 5.1 T (q1w) followed by AC (q2w or q3w) Administration

Agent	Dose	Route	Cycle
Paclitaxel	80 mg/m^2	IV	1–12
Doxorubicin	60 mg/m^2	IV	13–16
Cyclophosphamide	600 mg/m^2	IV	13–16

5.2 Standard Chemotherapy Treatment Plan for Control Arm, HER2+ Tumors

Participants randomized to the standard chemotherapy treatment arm who are HER2+ will receive 12 cycles of paclitaxel at 80 mg/m^2 q1wk ± 1 day plus trastuzumab q1w ± 1 day. Trastuzumab is given every week at a loading dose of 4 mg/kg for cycle 1 and maintenance dose of 2 mg/kg for cycles 2–12. For paclitaxel, Filgrastim can be used at investigators discretion. A minimum of seven days after completing the paclitaxel regimen, participants will receive four cycles of doxorubicin at 60 mg/m^2 plus cyclophosphamide at 600 mg/m^2 q2w ± 1 day or q3w ± 1 day at physician discretion. For AC, Filgrastim or Pegfilgrastim can be used at investigator's discretion. **All treatment doses should be based on actual body weight and not ideal body weight. If participant's body weight increases or decreases by ≥10% from baseline during the course of the treatment phase, the body surface area and agent dose should be recalculated.**

Due to recent accelerated approval of pertuzumab, the paclitaxel plus trastuzumab control arm has temporarily been closed in accordance with the recommendations of the I-SPY 2 DSMB and Investigators.

Table 5.2 TH (q1w) Followed by AC (q2w or q3w) Administration

Agent	Dose	Route	Cycle
Paclitaxel	80 mg/m^2	IV	1–12
Trastuzumab	4 mg/kg	IV	1
Trastuzumab	2 mg/kg	IV	2–12
Doxorubicin	60 mg/m^2	IV	13–16

CONFIDENTIAL

Cyclophosphamide	600 mg/m^2	IV	13–16

5.3 Investigational Agent Treatment Plan

Participants randomized to an investigational agent may receive 12 cycles of paclitaxel at 80 mg/m^2 q1wk ± 1 day in addition to the investigational agent; however, for administration details of the investigational agent regimen, refer to the specific agent appendix(D–x). A minimum of seven days after completing the investigational agent regimen, participants will receive four cycles of doxorubicin at 60 mg/m^2 plus cyclophosphamide at 600 mg/m^2 q2w ± 1 day or q3w ± 1 day at physician discretion. For AC, Filgrastim or Pegfilgrastim can be used at investigator's discretion. **All treatment doses should be based on actual body weight and not ideal body weight. If participant's body weight increases or decreases by ≥10% from baseline during the course of the treatment phase, the body surface area and agent dose should be recalculated.**

5.4 Premedications for Paclitaxel, Trastuzumab, and Doxorubicin plus Cyclophosphamide

It is recommended that the treating physician follows National Comprehensive Cancer Network guidelines (www.nccn.org). The guidelines are provided in Appendix B.

5.5 Concomitant Medication

ER positive participants ONLY:
Any form of ovarian ablation is prohibited (*e.g.*, gonadotropin-releasing hormone (GNRH) agonist, oophorectomy, radiation, *etc.*) before surgery.

For all participants:
Birth control usage and an oophorectomy at the time of primary breast surgery are acceptable. Aromatase inhibitor for limited use to harvest oocytes prior to starting neoadjuvant chemotherapy is allowed.

5.6 Toxicity Management and Dose Modifications

Toxicity management and dose modifications for standard therapy are outlined in Appendix B.
Toxicity management and dose modifications for investigational agents are outlined in each specific agent appendix (Appendices D–x).

5.7 Doxorubicin Shortage Guidelines

Due to ongoing supply issues, shortages of doxorubicin may occur intermittently. The following guidelines should be followed with regard to doxorubicin administration:

CONFIDENTIAL

Participant Status when Doxorubicin Shortage is Experienced	When to use Epirubicin	Epirubicin Dosage	Dose Modification and Monitoring for Epirubicin	Doxorubicin Supply Restored
Currently Receiving Doxorubicin	• Switch to epirubicin at the next scheduled cycle<br>• Continue treatment per study schedule	Starting dose: 90mg/m^2	• Epirubicin dose modification can be made using percentage adjustments specified in Appendix B, Table 3 of the protocol.<br>• All aspects of therapy and monitoring should be followed as specified in the protocol.<br>• Document in research record that substitution was due to doxorubicin shortage	Return to using doxorubicin at an equivalent dose to the previously administered epirubicin.
Not Yet Receiving Doxorubicin<br>• Site cannot guarantee at least 2 cycles of doxorubicin at time of initiating anthracycline treatment	• Use epirubicin for all 4 cycles<br>• Continue treatment per study schedule	Starting dose: 90mg/m^2	• Epirubicin dose modification can be made using percentage adjustments specified in Appendix B, Table 3 of the protocol.<br>• All aspects of therapy and monitoring should be followed as specified in the protocol	Continue with epirubicin for all cycles
• Site can guarantee at least 2 cycles of doxorubicin	• Use doxorubicin as instructed in §5 of the protocol.<br>• If shortage occurs, follow guidelines above for participants receiving doxorubicin			

NOTE: As participants in I-SPY 2 receiving neoadjuvant therapy are potentially curable, and since epirubicin has been shown to be an effective agent for breast cancer with no additional side effects, the change to epirubicin can be made immediately to avoid withholding chemotherapy treatment in this potentially curable participant population.

5.8 Adjuvant Treatment Recommendations

There are no adjuvant treatment requirements for this trial. Adjuvant therapy is the discretion of the treating physician and participant. However, it is recommended that participants receive the standard of care following neoadjuvant chemotherapy and surgery, including hormonal therapy for a minimum of five years if HR+, one year of trastuzumab if HER2+, and radiation therapy if indicated.

CONFIDENTIAL

6. INVESTIGATIONAL AGENT INFORMATION (See Appendices C and D–x)

The I-SPY 2 TRIAL protocol and IND are structured to enable the seamless addition and release of investigational agents over the course of the trial. When an investigational agent is added or released from use in this trial, only relevant appendices require updating, specifically Appendix C and the corresponding investigational agent's appendix.

Appendix C: Overview of all investigational agents in the study
Appendix D–x: All investigational agent-specific information (one investigational agent per appendix). As investigational agents are added to the trial, they will appear as subsequent appendices.

Each investigational agent falls into one of three categories as described in Appendix C: 1) Agents approved, pending activation for randomization, 2) Agents approved, activated for randomization, and 3) Agents graduated or dropped, no longer activated for randomization.

Adding an Investigational Agent to the Trial
To add a new investigational agent or (new dose or combination of agents) when eight arms are active, the trial team prepares the protocol amendment containing: 1) the new investigational agent's appendix, 2) the corresponding supplemental informed consent, and 3) an updated Appendix C showing the new agent in Table 1.1 (Investigational Agents Approved, Pending Activation for Randomization).

The protocol amendment will be considered a major modification to the protocol and will require a full IRB committee review; however, it will not require stopping accrual to the trial because there will be no change to Table 1.2 (Investigational Agents Approved, Activated for Randomization). New investigational agents will remain in this category until all trial sites have received IRB approval and there is space in the randomization engine for the new agent.

When the randomization engine has room for a new agent to be added, a protocol amendment will be generated updating Appendix C by moving the new investigational agent from Table 1.1 (Investigational Agents Approved, Pending Activation for Randomization) to Table 1.2 (Investigational Agents Approved, Activated for Randomization).

The protocol amendment will be considered a minor modification to the protocol and will require an expedited review by the IRB, which should take about 1 to 2 weeks. The trial will not have to stop accruing during this period, because participants will not be randomized to the new investigational agent until all the sites have IRB approval for the agent.

In summary, two steps must occur at the site level to add and use an investigational agent to the trial when eight arms are active:

1) A protocol amendment to ***add*** the new investigational agent must be submitted and approved by the full IRB committee at each site
2) A second protocol amendment to ***activate*** the new investigational agent for randomization must be approved by expedited IRB review at each site

When fewer than eight arms are active, it is possible to add and activate an agent in one subsequent amendment. In that case, the trial team prepares the protocol amendment containing: 1) the new investigational agent's appendix, 2) the corresponding supplemental informed consent, and 3) an updated Appendix C showing the new agent in Table 1.2 (Investigational Agents Approved, Activated for

CONFIDENTIAL

Randomization). After the protocol undergoes full IRB committee reviews and approval is obtained by all sites, the arm is considered activated.

Releasing an Agent from the Trial
When an investigational agent is graduated or dropped from the trial, a protocol amendment will be generated. Appendix C will be updated moving the agent from Table 1.2 (Investigational Agents Approved, Activated for Randomization) to Table 1.3 (Investigational Agents Graduated or Dropped, No Longer Active for Randomization).

The protocol amendment will be considered a minor modification to the protocol. The protocol will be submitted concurrently to all the study site's IRBs and will only require expedited IRB review and approval, which should take 1 to 2 weeks. The trial will not have to stop accruing to the other treatment arms during this period.

CONFIDENTIAL

7. BIOMARKERS FOR ELIGIBILITY, STRATIFICATION, AND RESPONSE MONITORING

7.1 Tissue and Blood Specimens for Biomarker Assessment

As standard of care, a diagnostic pretreatment core will be evaluated by a local pathologist according to local histopathologic standards. This includes a hematoxylin and eosin (H&E) stain and FDA-cleared/approved IHC/FISH assessment for ER, PgR, and HER2.

Tissue will be acquired at three time points during the trial for this study. Pretreatment (time point 0) and at the end of the third cycle of paclitaxel (time point 1), four 16-gauge core needle biopsies will be taken using an image-guided 16-gauge biopsy device. At the time of surgery (time point 3), a representative sample of tumor will be collected and cut into two pieces, only if sufficient tumor remains. The surgical specimen will be assessed using standard histopathologic parameters and the RCB technique to evaluate the extent of residual disease by the site pathologist. The surgical assessment will be used to determine the primary endpoint, pCR.

Pretreatment cores will serve to assess biomarkers that are used to evaluate eligibility as well as stratification in the trial (§7.2). The remainder of the pretreatment cores, as well as all tissue samples from time points 1 and 3, will be used to perform qualifying biomarker assays (§7.3) and for further research (exploratory biomarkers, §7.4).

All I-SPY 2 TRIAL tissue specimens will be embedded in OCT in a cryostat mold and frozen at –80°C, then sent to the I-SPY Lab at University of California, San Francisco (UCSF) on dry ice (§10). When applicable, a frozen core can be thawed and processed for formalin fixation and paraffin embedding (FFPE). If there is no tumor on the research tissue samples, a surgical pathology block will be provided as needed.

Blood samples will be drawn pretreatment (time point 0), early paclitaxel (time point 1), inter-regimen (time point 2), and before surgery (time point 3); see 'Schema' section, Figure C. Serum, plasma, and buffy coat cells will be stored at –80°C for research (§10). Samples will be shipped batch-wise to the I-SPY Lab at UCSF.

7.2 MRI and MRI Functional Tumor Volume (FTV) for Biomarker Assessment

All MRI exams will be performed as described in Figure C, Schedule of Study Procedures. Each participant should have all MRI exams performed using the same magnetic configuration (manufacturer; field strength; breast coil model).

7.2.1 MRI Scan Protocol

The breast MRI protocol includes a T2-weighted sequence, diffusion-weighted imaging (DWI) sequence, and dynamic contrast-enhanced (DCE) series using a bilateral, 3D, fat-suppressed, and T1-weighted sequence with 80–100 second temporal resolution.

7.2.1.1 General Requirements

- 1.5T or 3.0T whole body MRI scanner
- Dedicated breast radiofrequency coil
- Participant scanned in prone position with in-dwelling IV catheter

CONFIDENTIAL

- Contrast agent injection with FDA-approved gadolinium-based contrast agent; **the same contrast agent brand should be used for all MRI exams for the same participant**.
- The MRI exam will include a localization scan, a T2-weighted sequence, and a diffusion-weighted sequence, followed by a contrast-enhanced T1-weighted series:
  - T2-weighted sequence performed before contrast
  - Diffusion-weighted sequence performed before contrast
  - T1-weighted sequence with 80–100 second temporal resolution, performed once pre-contrast and multiple times post-injection using identical sequence parameters (see MOP §Imaging procedures); transmit and receive **gain settings should remain constant** for pre- and post-contrast T1-weighted imaging
  - Pre-contrast T1 images should be checked prior to contrast injection to confirm acceptable fat-suppression
  - Post-contrast imaging should continue for at least 8 minutes following contrast agent injection
  - Care should be taken to **select the smallest field of view (FOV) and slice coverage that completely encompasses both breasts and axilla**

NOTE: For guidelines on the specific pulse sequence parameters, see MOP §Imaging procedures.

7.2.1.2 ACRIN 6698 Co-study for Participating Sites—Closed to Accrual

- Sites participating in ACRIN 6698 will use the MRI protocol as specified in the ACRIN 6698 protocol. Language in the model consent about the ACRIN 6698 co-study is only included for those sites participating in this co-study.

NOTE: The study is closed to accrual. It has met its accrual goals.

7.2.2 MRI Functional Tumor Volume Assessment

All MR volume measurements will be automated and measured using software on the Hologic work station. Directly following each MRI examination, image data will be transferred to the local Aegis workstation for processing by the radiology technologist or study coordinator, who places rectangular regions-of-interest on cranio-caudal and medial-lateral projection views surrounding the tumor, in order to restrict the volume of calculation.

Data acceptability for processing will be assessed by the technologist based on several quality factors (success of contrast injection, absence of participant motion, or other artifacts). Tumor volume measurements will be computed according to the signal enhancement ratio (SER) method developed at UCSF [35]. The subsequent calculation of FTV is automated as part of the Aegis with SER plug-in software, with verification by the study radiologist.

Specifically, using the series of high-resolution T1-weighted images acquired before and following the injection of gadolinium-based contrast agent, the percent enhancement (PE), defined as the change in signal intensity at 2.5 minutes post-contrast relative to pre-contrast signal intensity (or PE = $[(S_1–S_0)/S_0] \times 100$, where S_0 and S_1 represent the signal intensities [SI] of each voxel in the pre-contrast and first post-contrast images) will be measured at every pixel in the image. Tumor volume will be calculated as the sum of all pixels meeting a predefined threshold of 70%*. Tumor pixels will be further characterized by their SER value, defined as the ratio of early enhancement to late enhancement $(S_1–S_0)/(S_2–S_0)$, where S_0

CONFIDENTIAL

and S_1 are as defined previously and S_2 represents the signal intensity in the late post-contrast image. Tumor volume will be segmented into sub-volumes with high, moderate, and low SER values. MRI FTV is computed by summing all eligible voxels with SER values above a threshold value of 0.9*.

Following verification by the study radiologist, tumor volume measurements will be sent to the I-SPY 2 electronic clinical trial data capture system (TRANSCEND) in the MRI volume case report form.

*Threshold may vary by imaging site depending on equipment and site-specific protocol variations.

7.3 Incorporation of Established Biomarkers

IHC markers based on community standards (FDA-cleared/approved tests at each site's participating pathology laboratory) will be collected from a participant's diagnostic breast core biopsy as part of the standard of care. These markers include the expression of tumor ER, PgR, and HER2, complemented by HER2 FISH as appropriate (for HER2 IHC 2+ cases, perform FISH testing as indicated by FDA). A study pretreatment frozen core will be sectioned to generate one H&E section. Appropriate sections will be further processed by Agendia Inc for RNA expression assessment of the TargetPrint HER2 and MammaPrint using the Agendia MammaPrint 44K full genome microarray manufactured by Agilent Technologies. A tumor will be considered ER+ and/or PgR+ if there is five percent or greater positive tumor staining. A tumor will be considered HER2+ if any one of the following three conditions are met: a) HER2 IHC 3+; b) overexpression by FISH (as defined by FDA-cleared/approved tests used at each institution); or c) TargetPrint HER2+. When an increase in CEP17 copy number is observed by FISH (*i.e.*, "polysomy"), the participant will be considered HER2+ if the ratio of HER2 signals/nucleus is greater than 6.

Women who are low-risk by MammaPrint and ER+ as well as HER2– will not be eligible for this trial (see eligibility scheme). Eligible women who are MammaPrint high-risk or MammaPrint low-risk and ER–, or MammaPrint low-risk and ER+ and HER2+ will be stratified based on their HER2, HR, and MammaPrint expression and subsequently randomized according to the adaptive randomization scheme (see §3).

In addition to pCR, MR volume change will also be used to inform the adaptive randomization.
The established biomarkers used will fulfill FDA regulatory requirements, including FDA clearance, or FDA IDE. The IDEs filed with the Master IND will include additional technical details.

7.4 Incorporation of Qualifying Biomarkers

Pretreatment (time point 0) and early paclitaxel (time point 1) biopsies will be used to generate qualifying biomarkers. Qualifying biomarkers are defined as those assays with promise to predict response to standard chemotherapy and investigational agents. These will include gene and protein (pathway) measurements by Agilent 44K and Affymetrix arrays, mRNA arrays, and RPMA assays to determine if molecular characteristics (biomarkers/pathways) correlate with pathologic, imaging, or RCB response measures in the neoadjuvant setting using the investigational therapeutic agents. Qualifying biomarkers will be performed under Clinical Laboratory Improvement Amendment (CLIA) conditions and have the potential to be used during the course of the trial for participant stratification, for which they then need to acquire IDE status.

Over the course of the trial, additional qualifying biomarkers will be put forward and tested for their ability to predict tumor response to specific classes of investigational targeted therapeutics, in addition

CONFIDENTIAL

to validating molecular profiles proposed by investigators that predict pCR to standard anthracycline and taxane-containing neoadjuvant chemotherapy [36–39].

Cell Line Agent Response Profiling: All agents being used in the trial will be tested against a 60-cell-line panel using an *in vitro* system created by Joe Gray, PhD, Professor, Laboratory Medicine and Radiation Oncology, UCSF; Director, Division of Life Sciences, Lawrence Berkeley National Laboratory. Gene expression patterns found on Affymetrix arrays that identify likelihood of response or resistance to a given agent will be transferred to a high throughput mRNA array assay [40].

RPMA: Five 8-micron sections of tissue from a core will be used for protein lysate arrays. The cut sections of the frozen core will be used for pathway analysis, *e.g.*, total HER2 and phosphorylated HER2 (pHER2) assay by RPMA by George Mason University [41].

TFAC/RCB predictor: A gene expression profile predicting likelihood of response to standard chemotherapy, TFAC, has been established. Sections of a frozen core will be processed for mRNA expression analysis on Affymetrix arrays to validate this predictor by MD Anderson Cancer Center (MDACC) Molecular Laboratory [42].

Phase 2 agent response biomarkers: Biomarker response to the various investigational agents will be tested on the appropriate biospecimen, *e.g.,* tumor tissue, blood, and will include gene expression patterns derived from single gene/protein assessments or will encompass patterns/pathways as captured by multiple index assays. For the scientific rational for the biomarkers selected, see specific investigational agent appendix.

Pharmacogenomics by single-nucleotide polymorphism (SNP) and GWAS: Paclitaxel toxicity has been correlated to gene variant alleles that affect metabolism of the agents. Specific SNPs will be evaluated on DNA extracted from blood samples, as well as by GWAS analysis.

Candidate gene SNPs: In addition to the GWAS approach to toxicity, we will utilize the Illumina Human 610-Quad BeadChip technology to evaluate SNPs. SNP data will be generated to examine candidate SNPs, SNP signatures, and haplotypes predicting response to other standard agents being utilized, as well as investigational agents being tested in the I-SPY 2 TRIAL. For example, CYP3A4 and glutathione-*S*-transferase polymorphisms previously shown to modulate response to cyclophosphamide [43] will be correlated to imaging response to the anthracycline/cyclophosphamide regimen in all participants receiving this therapy. In addition, an interleukin-6 haplotype signature associated with early relapse in ER+ participants will be examined in those participants with ER+ disease [44]. Generation of genome-wide SNP data will enable additional germline SNP signatures to be identified for the investigational agents being tested in I-SPY 2 as exploratory biomarkers.

7.5 Incorporation of Exploratory Biomarkers

Additional tissue will be used to enable molecular assays to be performed using next generation technology platforms. Exploratory biomarkers for response prediction can be evaluated in a pure research setting. Likely assays include sequencing, methylation, microRNA, *etc.*; new technologies will be explored once they become available and are judged valuable. Once the new assays are performed, they can be compared to molecular assays already performed on the Agilent platforms and reverse phase tissue arrays, as well as by IHC.

CONFIDENTIAL

Peripheral blood samples: At various time points as indicated in the trial scheme, peripheral blood sampling will be performed. Blood samples will be processed to enable further biomarker research employing different techniques. Serum, plasma, and buffy coat will be stored in multiple aliquots.

Circulating tumor cells: Methods will be chosen that are compatible with the FNIH Biomarker Consortium circulating tumor cell trial (http://www.fnih.org).

7.6 Repository for Storing, Analyzing, and Comparing Assay Results

All specimens and specimen transformation will be recorded and tracked using caTISSUE, the web-based tissue tracking and shipping tool developed by the NCI. Specimen tracking numbers will be used to track and store assay results. Assay results will be stored in caINTEGRATOR , the NCI data repository built for cross-platform analysis. In addition, we will employ other analytic tools such as the University of California-Santa Cruz (UCSC) Cancer Genome Browser.

CONFIDENTIAL

8. CLINICAL EVALUATIONS AND PROCEDURES

8.1 Schedule of Events

Table 8.1 Study Calendar

Evaluation/ Procedure	Registration & Screening (Pre-randomization)	Paclitaxel Regimen[a]	AC Regimen	Pre-Surgery	Follow-up
Informed Consent	X	X (post-randomization)			
Assess Eligibility	X				
Medical History	X				
Physical Exam	X	X	X	X	X
Laboratory Blood Tests[b]	X	X	X		
Pregnancy Test	X				
Investigational Agent-specific Laboratory and Assessment Tests[c]	X	X	X	X	
Metastatic Evaluation	X				
Breast MRI	X	X (end of week 3)	X (pre-AC)	X	
ECHO/MUGA	X	X[c] (end of week 12)	X[d] (post-AC)		X[e]
Study Biopsy/Tissue Collection	X	X (end of week 3)		X (at time of surgery)	
Study Blood Draw for Serum, Plasma, and Buffy Coat	X	X (end of week 3)	X (pre-AC)	X	
Clinical Assessment	X	X	X	X	X
Administration of Investigational Agent		X			
Adverse Event Collection		X	X	X	X (30 days, 6 and 12 months post-surgery)

[a]For participants randomized to an investigational arm that does not include paclitaxel, see §2.5 of each agent-specific appendix for modifications to standard procedures(Appendices D-N).
[b]See§8.3.
[c]For investigational agent-specific evaluations, refer to the appropriate Appendix.
[d]ECHO or MUGA performed on HER2+ participants (*i.e.*, receiving trastuzumab and/or specific investigational agents). For investigational agent-specific evaluations, refer to the appropriate Appendix.
[e]ECHO or MUGA performed once every 3 months for as long as HER2+ participant continues on trastuzumab or pertuzumab post-surgery as standard of care, *e.g.*, 9–12 months.

8.2 Baseline Testing/Pretreatment Evaluation

The following procedures will be done before a participant is randomized for the study:

- Medical history and physical exam (including collection of height, weight, ECOG score)
- Histologically confirmed invasive breast cancer (including hormone evaluation by local pathology laboratory)
- Laboratory blood tests including (if applicable, within ranges defined in §4):
  - CBC with differential, including:
    - White blood cell (WBC) count

CONFIDENTIAL

- WBC differential (neutrophils, lymphocytes, monocytes, eosinophils, basophils)
        - Platelet count
        - Hemoglobin
        - Hematocrit
    - Electrolyte panel, including:
        - Sodium
        - Potassium
        - Chloride
        - Total carbon dioxide
        - Anion gap
    - Liver function tests, including:
        - Total bilirubin
        - ALT
        - AST
        - ALP
    - Kidney function tests, including:
        - Blood urea nitrogen (BUN)
        - Creatinine
    - Pregnancy test for participant of child-bearing potential within 14 days of entry to screening phase
- Other laboratory and assessment tests as needed based upon investigational agent requirements; see Appendix C §2
- ECHO or multigated acquisition scan (MUGA) evaluation
- Metastatic evaluation (tests can be used if done within 30 days of consenting to treatment phase)—required testing for this trial to rule out distant metastatic disease (as defined in §4.1.2. E); includes any of the following:
    - PA and lateral CXR
    - CT with or without PET
    - Radionuclide bone scan
    - LFTs including total bilirubin, ALT, and AST within ranges defined in §4.1.2 C
- Breast MRI (to be completed within 30 days of starting study treatment)
- Study breast core biopsy (used for MammaPrint, TargetPrint HER2, qualifying, and exploratory biomarkers)
- Study blood draw

8.3 Evaluations During Neoadjuvant Chemotherapy Treatment

The following procedures will be done during the participant's paclitaxel regimen:

- Paclitaxel, if given based upon randomization, administered weekly
- Trastuzumab, if given based upon randomization, administered weekly
- Investigational agent, if given based upon randomization, administered on agent's specific dosing schedule
- Clinical assessment, breast MRI, blood draw, and core biopsy at the end of week 3, prior to the fourth paclitaxel infusion
- ECHO/MUGA evaluation post-paclitaxel and trastuzumab for all HER2+ participants (every three months in conjunction with standard of care)
- Laboratory blood tests q1w, in conjunction with paclitaxel dosing (can be done within 2* days of paclitaxel administration):
    - CBC with differential, including:

CONFIDENTIAL

        - White blood cell (WBC) count
        - WBC differential (neutrophils, lymphocytes, monocytes, eosinophils, basophils)
        - Platelet count
        - Hemoglobin
        - Hematocrit
    - Electrolyte panel, including:
        - Sodium
        - Potassium
        - Chloride
        - Total carbon dioxide
        - Anion gap
    - Liver function tests, including:
        - Total bilirubin
        - ALT
        - AST
        - ALP
    - Kidney function tests, including:
        - Blood urea nitrogen (BUN)
        - Creatinine

* NOTE: For C1D1 of paclitaxel treatment, labs drawn during the screening phase can be used if done within 3 weeks (21 days) of treatment start.

- Other laboratory and assessment tests as needed based upon investigational agent requirements, see investigational agent appendices (§2.5).
- AE reporting every three cycles in conjunction with the participant's weekly clinic visit.

Treatment or visit delays for public holidays or weather conditions do not constitute a protocol violation

The following procedures will be done during the participant's AC regimen:

- Clinical assessment, breast MRI and blood draw prior to starting AC treatment
- AC administered q2w or q3w at physician discretion
- Standard laboratory blood tests q2w or q3w, in conjunction with AC dosing (can be done within 2 days of AC administration):
    - CBC with differential, including:
        - WBC count
        - WBC differential (neutrophils, lymphocytes, monocytes, eosinophils, basophils)
        - Platelet count
        - Hemoglobin
        - Hematocrit
    - Electrolyte panel, including:
        - Sodium
        - Potassium
        - Chloride
        - Total carbon dioxide
        - Anion gap
    - Liver function tests, including:
        - Total bilirubin
        - ALT
        - AST
        - ALP

CONFIDENTIAL

- Kidney function tests, including:
    - BUN
    - Creatinine
- AE reporting every cycle of AC in conjunction with participant's two- or three-week clinic visit
- ECHO/MUGA evaluation post-AC for all HER2+ participants (every three months in conjunction with standard of care)

Treatment or visit delays for public holidays or weather conditions do not constitute a protocol violation

8.4 Evaluations at Completion of Neoadjuvant Chemotherapy Treatment

The following procedures will be done following completion of the participant's neoadjuvant chemotherapy:

- Breast MRI prior to surgery
- Study blood draw
- Tissue collected for the study at the time of surgery, if tumor remains on gross examination and sampling will not impact the final diagnostic evaluation

8.5 Postsurgery Follow-up

The participants will be followed for five years following the date of surgery for survival and recurrence. Follow-up will be collected every six *(± one month)* months for five years. New S/AEs will be collected up to 30 days *(± one week)* following the participant's surgery; continuing S/AEs will be monitored until resolution/baseline or 12 months postsurgery, whichever occurs first (See table 8.2 for specifics). For HER2+ participants receiving trastuzumab or pertuzumab postsurgery, ECHO or MUGA will be performed once every three months for 9–12 months during the follow-up period as standard of care.

Table 8.2: Follow-up S/AE Collection Criteria

**Related S/AEs: Follow until one of the following occurs:**	**Unrelated severe or life threatening S/AEs: Follow until one of the following occurs:**
– Resolved or improved to baseline	– Resolved or improved to baseline
– Relationship is reassessed as unrelated	– Severity improved to Grade 2
– Death	– Death
– Start of new anti-cancer regimen	– Start of new anti-cancer regimen
– Investigator confirms that no further improvement can be expected	– Investigator confirms that no further improvement can be expected
– Clinical or safety data will no longer be collected, or final database closure	– Clinical or safety data will no longer be collected, or final database closure

CONFIDENTIAL

The final outcome of each adverse event must be recorded on the eCRF

Since OS is a study endpoint, AEs with a fatal outcome more than 30 days following surgery will be collected only as survival status (not as an SAE), unless there is evidence suggesting a causal relationship between the protocol treatment and the event with a fatal outcome. See specifics regarding progression in section 8.7.

8.6 Evaluations for Premature Discontinuation of Study Treatment

Since this is an intent-to-treat trial participants who discontinue their randomized treatment assignment prematurely for any reason will remain "on study" to complete the remaining study procedures and follow-up, including the following:

- Breast MRI, blood draw, and core biopsy at the end of week 3, prior to the fourth weeks of treatment
- Clinical assessment, breast MRI and blood draw prior to starting AC treatment
- Clinical assessment, breast MRI, and study blood draw prior to surgery
- Tissue collection at the time of surgery, if tumor remains on gross examination and sampling will not impact the final diagnostic evaluation
- For HER2+ participants continuing to receive trastuzumab or pertuzumab, ECHO or MUGA performed once every three months for 9–12 months during the follow-up period as standard of care
- All new S/AEs will be collected up to 30 days following the last administration of study treatment.
    - SAEs resulting from a study procedure (breast MRI, core biopsy, blood draw) will continue to be collected until surgery.
- Ongoing S/AEs will be monitored until resolution/baseline or 12 months, whichever occurs first. (See table 8.2 for specifics).
- For participants that were randomized to an investigational agent:
    - Collect safety labs and assessment tests 30 days after last dose of investigational agent, see investigation agent appendices (§2.5).

- For participants who choose to forgoe any of the above described procedures, a protocol deviation should be filed.

If a participant withdraws from the study prematurely, we will collect new adverse events, safety labs and assessment tests 30 days after last dose of study treatment; see specific investigational agent appendices (D-N).

8.7 Disease Progression

If progression of the primary tumor or evidence of metastasis requires discontinuation from study medication, then local practice should be followed. For participants who progress on-study treatment, new S/AEs will be collected 30 days following last study treatment administration (paclitaxel and/or investigational agent, or AC); continuing S/AEs will be monitored until resolution/baseline or 12 months following last study agent administration, whichever occurs first. See table 8.2

Progression should not be reported as an adverse event if it is clearly consistent with the suspected progression of the underlying cancer. Hospitalization due solely to the progression of

CONFIDENTIAL

underlying malignancy should not be reported as a serious adverse event. If there is any uncertainty about an adverse event being due only to the disease under study, please contact the DCC at (855)-889-5170 for further guidance on reporting.

9. CRITERIA FOR EVALUATION AND ENDPOINT DEFINITION

9.1 Primary Endpoint

To assess objective response rates, as measured by pCR, of investigational agents in combination with standard neoadjuvant paclitaxel, doxorubicin, and cyclophosphamide. This measurement will occur after the end of chemotherapy during pathologic assessment of residual disease.

pCR is defined as no residual invasive cancer in the breast (at the time of definitive surgical resection)or in the lymph nodes (no invasive tumor by H&E). A study-trained pathologist will evaluate pCR. The Study Lead Pathologist will make the final determination on any indeterminate or contested results.

9.2 Secondary Endpoints

Additional information on the response to paclitaxel plus or minus a targeted agent will be obtained by measuring the change in MRI volume from the baseline MRI to the MRI performed following the completion of paclitaxel based therapy and by measuring RCB at time of pathologic assessment of residual disease.

9.2.1 MRI FTV

Measured from dynamic contrast-enhanced (DCE) MR images of the breast, FTV calculation is based on the SER breast MRI technique developed at UCSF and used in the ACRIN 6657 multi-center clinical trial [45–47]. High spatial resolution contrast-enhanced MRI is performed using a three-time point method that acquires images before, immediately following contrast injection, and in the late phase of contrast passage, using a small molecular weight gadolinium-containing contrast agent, administered intravenously. DCE-MRI data are transferred to the Hologic Aegis workstation for FTV processing immediately following the MRI examination. The measurement is verified by the study local radiologist.

9.2.2 Residual Cancer Burden

RCB was proposed as a determinant of the extent of residual disease in the post-treatment surgical resection specimen of participants with breast cancer who received preoperative chemotherapy [16]. Six variables are included in the formula. An RCB index value can also be calculated and involves the categorization into one of four RCB classes (RCB 0 or pCR, RCB I or near pCR, RCB II, RCB III). The calculation formula and detailed description can be found at: www.mdanderson.org/breastcancer_RCB. In brief, the variables include cross-sectional dimensions of the residual tumor bed (d1 and d2), estimate of the proportion of that residual tumor bed area that is involved by cancer (% CA), estimate the proportion of the cancer that is *in situ* component (% CIS), number of positive lymph nodes (LN), and measure of the diameter of the largest nodal metastasis (dmet). RCB variables will be collected by the study local pathologist, who will be trained in the technique. The Study Lead Pathologist will make the final determination on any indeterminate and contested results.

CONFIDENTIAL

9.2.3 Three- and Five-year Relapse/RFS and OS

Three- and five-year RFS and OS will also be assessed. RFS is defined as local/regional invasive recurrence, invasive ipsilateral breast tumor recurrence, distant recurrence, inoperable (meaning no surgery because of progression), and/or death from breast cancer RFS will be calculated from the time of treatment to event. OS is defined by death from breast cancer, non-breast cancer, unknown, or any other cause and will be calculated from the time of study entry to event [48].

9.3 Off-Investigational Agent Criteria

Off-investigational agent criteria is specified in the agent-specific appendix. Participants may stop taking study agent for the following reasons: toxicity; inadequate agent supply; or participant preference. This is an intent-to-treat trial, so participants will continue to be followed in order to continue to collect study data according to the schedule of events. See MOP for instructions on how to document participant's preference on discontinuing the investigational agent early (see section 8.6).

If an investigational agent reaches a threshold for graduation or is dropped for futility, no additional participants will be randomized to that agent. Participants currently receiving that agent will continue on the regimen until they complete the entire course of treatment (for graduating agents), or the option to continue or drop the agent will be at the discretion of the participant and her treating physician (for agents dropped for futility). In the later instance, participants will continue on-study but will revert to the standard/control regimen and will remain part of the arm to which they were assigned. See §3.4 for additional details regarding participant treatment options when an agent leaves the trial.

9.4 Off-study Criteria

Participants may go off-study either because the protocol intervention and any protocol-required follow-up period is completed or because the participant withdraws consent. See the individual agent appendices for further instruction. See MOP for further instructions for participants withdrawing consent.

9.5 Study Termination

QuantumLeap Healthcare Collaborative (QLHC) as the study Sponsor has the right to discontinue the study at any time.

CONFIDENTIAL

10. SPECIMEN MANAGEMENT

10.1 Central Laboratories

All study samples will be sent to the I-SPY Laboratory, as part of the UCSF CLIA facility, for processing of the samples in a timely manner. The frozen cores will be sectioned to determined presence of tumor and tumor density by H&E stain (results will be available in caTISSUE for all users to access). A core with sufficient tumor will be sectioned and distributed to appropriate labs listed in §10.3. A frozen core with tumor cells present will be FFPE, with sections distributed to appropriate labs listed in §10.3.

Blood samples will be aliquoted for distribution as listed in §10.3.

All quality and quantity specimen data will be stored in caTISSUE, as well as shipment tracking information. Each laboratory involved in the analysis of samples will be responsible for entering appropriate assay data.

10.2 Specimen Collection, Handling, and Shipping Procedures

The frozen tissue specimens will be collected in the following manner:

- Four 16-gauge core biopsies will be collected at time points 0 and 1. If tumor remains, a representative section of tumor will be collected at the time of surgery (time point 3), and cut into two pieces. If there is no tumor on the research tissue samples, a surgical pathology FFPE block will be provided as needed.
- All tissue collected will be frozen in OCT. A single core is placed in the bottom of a cryo-mold and embedded with OCT (avoid bubbles around the tissue). The mold is then placed on dry ice until the OCT has frozen solid (this should be within five minutes of collecting the tissue to preserve the RNA). Store OCT-embedded tissue at –80ºC. Using a permanent marker, frozen samples will be labeled with: I-SPY 2, the specimen ID generated from the Tissue Specimen Collection case report form, and the date of collection.
- Samples should be placed in a biohazard-labeled plastic bag to ensure samples are in contact with dry ice during shipment (one participant's samples per bag). Ship frozen samples to the I-SPY Lab at UCSF on dry ice. Pretreatment cores should be shipped within one business day of collection. DO NOT SHIP SAMPLES ON FRIDAYS.

The blood samples for each time point will be collected in the following manner:

- One 5 ml marble/tiger-top vacutainer (containing no anticoagulant, only serum separator) tube of venous blood is collected.
    - Let marble/tiger-top tube sit for at least 15 minutes to properly clot.
    - Within two hours of collection, centrifuge the tube at 2500 rpm for 20 minutes at room temperature. Keeping the tube upright, aliquot approximately 1 ml of serum per 2 ml cryovial, for a total of three cryovials. Immediately freeze serum at –80ºC.
- Two (2) 5–10 ml EDTA/lavender-top tubes of venous blood are collected for plasma and buffy coat.
    - Invert EDTA tubes eight or more times immediately after collection.
    - Within two hours of collection, centrifuge the tubes at 1500 rpm for 20 minutes at room temperature. Keeping the tubes upright, aliquot 1ml of plasma per 2 ml cryovial, for a total of three cryovials.

CONFIDENTIAL

- Remove tiny white layer (buffy coat) and aliquot into two 2 ml cryovial tubes (it is acceptable to have a few red blood cells in the buffy coat). Immediately freeze plasma and buffy coat at –80ºC.
- Using a permanent marker, all samples will be labeled with: I-SPY 2, the specimen ID generated from the Blood Specimen Collection case report form, and the date of collection. All cryovials per participant should be placed in a biohazard-labeled plastic bag. All accumulated blood samples are shipped once a month on dry ice to the UCSF I-SPY Lab. DO NOT SHIP SAMPLES ON FRIDAYS.

All samples collected are sent to the I-SPY Lab at UCSF:

UCSF I-SPY Lab
2511 Bush Street, Room S441
San Francisco, CA 94115
Lab Contact: Lamorna Brown-Swigart
Email: Lamorna.Swigart@ucsf.edu

Lab Phone: 415-514-1035
Lab Email: ispylab@ucsf.edu

10.3 Ancillary Laboratories

The following laboratories will receive the following study samples for analysis from the I-SPY Lab at UCSF:

10.3.1 Agendia

Agendia will receive sections of tumor for RNA isolation and analysis on Agilent 44K microarray. The raw 44K array data and normalized 44K array data will be made available on caARRAY for other Investigators to access. MammaPrint and TargetPrint HER2 results will be available in caIntegrator2. Samples will be sent to the Huntington Beach facility at the address below. The Agendia Netherlands Main Office information is provided for reference only; samples will not be sent to this facility unless instructed:

Agendia, Inc
22 Morgan
Irvine, CA 92618
United States
Main Office:1-888-321-2732
Local contact: George Pounds

Agendia BV
Science Park 406
1098XH, Amsterdam
The Netherlands
Main Office: +31 (20) 462-1500
Local contact: Arno Floore

10.3.2 George Mason University (GMU)

The Center for Applied Proteomics and Molecular Medicine (CAPMM) at GMU will receive five frozen 8-micron sections of tissue for RPMA analysis. Samples will be sent to the following address:

Dr. Julie Wulfkuhle
George Mason University
Bull Run Hall Room 351
10900 University Blvd.
Manassas, VA 20110

CONFIDENTIAL

Phone: 703-993-4114
Lab contact: Dr. Julie Wulfkuhle

10.3.3 UCSF I-SPY Laboratory

UCSF I-SPY laboratory will receive two 8-micron sections of FFPE core for analysis of the mRNA array assay to predict agent sensitivity. Samples will be sent to the following address:

Drs. Joe Gray and Laura van't Veer
UCSF I-SPY Lab
2340 Sutter Street, Room S441
San Francisco, CA 94115
Lab Contact: Sarah Davis, MS
Phone: 415-885-7490
FAX: 415-353-7503

10.3.4 UCSF CLIA Pharmacogenomics

CLIA Pharmacogenomics laboratory will receive 400 nanograms of germline and tumor DNA (TBD) for Genome-wide SNP analysis. Samples will be sent to the following address:
Dr. Kathy Giacomini
UCSF
1550 4th Street
Mission Bay, RH 581
San Francisco, CA 94158
Phone: 415-514-4363
FAX: 415-514-4361

10.3.5 MDACC Molecular Laboratory

MDACC CLIA molecular laboratory will receive 1 microgram of isolated tumor RNA for analysis on Affymetrix U133A microarray and TFAC/RCB predictor. The raw array data and normalized array data will be made available on caARRAY. Samples will be sent to the following address:

MDACC
Attn: Dr. Fraser Symmans
8515 Fannin Street
Room NAO1.053
Houston, TX 77054-2512
Lab contact: Feng Lin
Phone: 713-792-2512

10.3.6 Investigational Agent Response Biomarkers – IGFR Inhibitor

Circulating bioactive IGF biomarkers will be evaluated in 100 µL of serum for those participants treated with an IGFR Inhibitor. The biomarker test includes IGF-IR and IGF-2. Samples will be sent to the following address:

Dr. Mary Hixon
Brown University Molecular Medicine Labs

CONFIDENTIAL

Attn: Caitlin Brown
70 Ship Street, Room 513
Chestnut Street Loading Dock
Providence, RI 02903
Lab contact: Caitlin Brown
Phone: 401-863-6125
FAX: 401-863-9008

10.3.7 Investigational Agent Response Biomarkers – Pan ErbB Inhibitor

Biomarkers that will be assessed for participants treated with a Pan ErbB Inhibitor. The biomarker test includes AKT-S308, PTEN, Stathmin by IHC, and HER2 by serum analysis. Samples will be sent to the following address:

TBD

10.3.8 Additional Samples

Additional samples will be approved for other qualifying and exploratory assays. The I-SPY 2 Biomarker Committee will receive, review, and recommend requests for samples to the I-SPY 2 Data Access and Publication Committee for final approval.

Biomarker data will be made available through the I-SPY 2 Data Portal (caINTEGRATOR), as indicated by the I-SPY 2 Data Access and Publication Guidelines.

CONFIDENTIAL

11. REPORTING ADVERSE EVENTS

Definition: An AE is any untoward medical occurrence in a study participant that does not necessarily have a causal relationship with the treatment or study participant. An AE can therefore be any unfavorable and unintended sign (including an abnormal laboratory finding), symptom, or disease temporally associated with participation in a study, whether or not related to that participation.

A list of AEs that have occurred or might occur (Reported Adverse Events and Potential Risks) can be found in the specific agent appendix as noted under §6.0, Investigational Agent Information, as well as the Investigator Brochure or package insert.

11.1 Adverse Events

11.1.1 Reportable AEs

Baseline symptoms will be collected once a participant signs the screening informed consent. All AEs are reported whether or not related to a study procedure or study treatment.

11.1.2 AE Data Elements

- AE reported date
- AE verbatim term
- Event onset date and event ended date
- Severity grade
- Attribution to study agent (relatedness)
- Whether or not the event was reported as an SAE
- Action taken with the study agent
- Outcome of the event
- Whether or not the participant dropped from the study due to AE
- Comments

11.1.3 Severity of AEs

Identify the AE using the NCI Common Terminology Criteria for Adverse Events (CTCAE) version 4.0. The CTCAE 4.0 provides a grading scale for each AE listed. A copy of the CTCAE 4.0 can be found at http://ctep.cancer.gov.

AEs will be assessed for severity according to the CTCAE 4.0, which provides unique clinical descriptions of grade for each AE term. These are based on the following general guidelines, which can be used to assess the severity of AE terms not listed in the CTCAE 4.0:

Grade 0 No AE
- Sign/symptom within normal limits

Grade 1 Mild AE
- Asymptomatic **or**
- Mild or minor symptoms **or**
- Marginal clinical relevance **or**
- Clinical or diagnostic observations only **or**

CONFIDENTIAL

- Intervention not indicated **or**
- Non-prescription intervention indicated

Grade 2 Moderate AE
- Intervention indicated **or**
- Minimal, local, noninvasive intervention (*e.g.,* packing, cautery) **or**
- Limiting instrumental activities of daily living (*e.g.*, shopping; laundry; transportation; ability to conduct finances)

Grade 3 Severe AE
- Medically significant but not life-threatening **or**
- Inpatient or prolongation of hospitalization indicated **or**
- Important medical event that does not result in hospitalization but may jeopardize the participant **or** may require intervention either
  - to prevent hospitalization **or**
  - to prevent the AE from becoming life-threatening or potentially resulting in death
- Disabling
- Results in persistent or significant disability or incapacity **or**
- Limiting self care activities of daily living (*e.g.*, getting in and out of bed; dressing; eating; getting around inside; bathing; using the toilet)

Grade 4 Life-threatening AE
- Life-threatening consequences
- Urgent intervention indicated
- Urgent operative intervention indicated
- Participant is at risk of death **at the time of the event** if immediate intervention is not undertaken
- Blindness or deafness (need to decide if unilateral or bilateral)

Grade 5 Fatal AE
- Death

All AEs and SAEs will be coded using MedDRA version 12.0 for reporting to the FDA, DSMB, and Institutional Review Boards (IRBs), as required.

11.1.4 Assessment of Relationship of AE to Treatment

The possibility that the AE is related to study agent will be classified as one of the following: unrelated, unlikely, possible, probable, and definite as described below:

- Unrelated (There is no evidence of causal relationship). Previous term was "Not Related."
- Unlikely (There is *little* evidence to suggest there is a causal relationship (*e.g.*, the event did not occur within a reasonable time after administration of the trial medication). There is *another reasonable explanation* for the event (*e.g.*, the participant's clinical condition, other concomitant treatments).
- Possible (There is *some* evidence to suggest a causal relationship (*e.g.*, the event occurred within a reasonable time after administration of the trial medication). However, the influence of *other factors may have contributed* to the event (*e.g.*, the participant's clinical

CONFIDENTIAL

condition, other concomitant events).

- Probable (There *is evidence* to suggest a causal relationship, and the influence of other factors is *unlikely).*
- Definite (There is *clear* evidence to suggest a causal relationship and other possible contributing factors can be *ruled out*).

11.1.5 Follow-up of AEs

All AEs, including lab abnormalities that in the opinion of the investigator are clinically significant, will be followed according to good medical practices, until resolution/baseline or 12 months, whichever occurs first. These events will be collected based on clinic visits planned for months six and 12 (± one month).

11.2 Serious Adverse Events

11.2.1 SAE Definition

Fed. Reg. 75, Sept. 29, 2010 defines an SAE as an event, occurring at any dose, which meets any of the following criteria:

- Results in death
- Is life threatening (*Note: the term life-threatening refers to an event in which the participant was at risk of death at the time of the event; it does not refer to an event which hypothetically might have caused death if it were more severe).*
- Requires inpatient hospitalization or prolongation of existing hospitalization
- Results in persistent or significant incapacity or substantial disruption of the ability to conduct normal life functions
- Is a congenital abnormality/birth defect
- Important medical events that may not result in death, be life-threatening, or require hospitalization may be considered serious when, based upon the appropriate medical judgment, they may jeopardize the participant or subject and may require medical or surgical intervention to prevent one of the outcomes listed.

11.2.2 Reporting Serious Adverse Events to QLHC

The organizations will report SAEs on the I-SPY 2 TRIAL SAE Report Form. The DCC safety department must be notified within 24 hours of knowledge of the event. The organization must submit the SAE report form within 48 hours of knowledge of the event. Please refer to the I-SPY 2 Manual of Operations for completion and submission guidelines.

Include the following information when calling:

- Date and time of the SAE
- Date and time of the SAE report
- Name of reporter
- Call-back phone number
- Affiliation/Institution conducting the study

CONFIDENTIAL

- Protocol number, title of protocol
- Description of the SAE, including reason serious and attribution to drug(s)

DCC will triage the reported information and inform the study interim Medical Monitor, below:

Sausan Abouharb, MD
Assistant Professor, Breast Medical Oncology, The University of Texas MD Anderson Cancer Center
15151 Holcombe Boulevard, Unit 1354
Houston, TX 77030 USA
Phone: (713) 745-3543
Email: sabouharb@mdanderson.org

The Medical Monitor and safety/regulatory staff will determine which SAEs require expedited FDA submission as safety reports. SAEs and AEs will be communicated to the relevant agent manufacturer per their individual safety reporting requirements regarding timing, frequency, and format.

All investigational sites will comply with applicable regulatory requirements related to reporting SAEs to the IRB/IEC.

Follow-up of SAE: Site staff should send follow-up reports as requested when additional information is available. Additional information should be entered on the study-specific SAE Report Form in the appropriate format. Follow-up information should be sent to the DCC Safety Department as soon as available. Ongoing SAEs will be followed until resolved or up to 12 months. These events will be collected in the study database.

12. STUDY OVERSIGHT AND MONITORING

This study is sponsored by QLHC, a 501(c)3 dedicated to the delivery of innovative healthcare solutions. Other partners involved in the trial's design and development include the FDA, University of California, San Francisco (UCSF), Berry Consultants, LLC and other collaborating regulatory agencies and investigators. I-SPY 2 Project Oversight Team ensures the effective, efficient and ethical conduct of the trial on behalf of QLHC and all participating agencies and sectors. The I-SPY 2 Executive Operations Group is responsible for the scripting and execution of the study protocol, as well as oversight of the trial operations. The I-SPY Committees (Agents, Biomarkers, Informatics, Pathology, Publications, Regulatory and Site Operations) in conjunction with the I-SPY 2 Project Management team will guide the trial's conduct both in the United States and internationally. Strict monitoring guidelines for phase 2 trials will apply and the utmost effort will be paid to the collection of data that is in compliance with FDA and other participating regulatory agency guidelines.

CONFIDENTIAL

12.1 Data Management

This study, based on the established I-SPY 2 TRIAL Data Access and Publication Guidelines, will collect and report clinical trial data using the NCI-sponsored TRANSCEND platform managed by UCSF. The Clinical Trial Data Capture (CTDC) application, a component of TRANSCEND, will be the database of record for the clinical trial data participant for the sponsor and FDA to audit. Due to the complex nature of the statistical design, a randomization engine with webservice was developed and is included as a component of TRANSCEND. In order to perform the required calculations for decision making and to perform randomization, all data relevant to the statistical modeling will be de-identified and flow from the CTDC application to the randomization engine via webservice; results will be returned to the CTDC. All participant case report forms will be submitted according to Table 12.1 in §12.2 and the DCC will review data in real time. All application users will be trained to use the system and will comply with the instructions in the protocol-specific "User Manual" provided by the UCSF/TRANSCEND team as well as applicable regulatory requirements such as 21 CFR Part 11. De-identified study data will be available in the caINTEGRATOR analysis portal for approved users, as outlined in the I-SPY 2 TRIAL Data Access and Publication Guidelines posted on the I-SPY 2 website: www.ispy2trial.org.

12.2 Case Report Forms

Participant data will be collected using protocol-specific case report forms (CRFs) utilizing cancer data standards registry and repository (caDSR) common data elements (CDEs). Study staff will enter data into the web-based eCRF in TRANSCEND; see submission schedule in Table 12.1. Instructions on how to use the TRANSCEND data capture system are provided in the TRANSCEND user's manual as part of the MOP.

The ability to randomize a participant and to ensure the randomization engine is updated with the most current response data is dependent on timely completion of the CRFs listed in Table 12.1. For a detailed submission schedule, refer to the MOP §TRANSCEND User Manual, I-SPY 2 Case Report Form Instructions.

Table 12.1 Case Report Form Submission Schedule

Form	When form is to be completed
Pre-eligibility Checklist	Completed for each participant considered for I-SPY 2 (also used to track pre-screen failures)
Registration Form	For each participant signing a screening consent form.
Menopausal Status Form	For each participant signing a screening consent form.
Tissue Specimen Form	For each core biopsy done for the study (pre-treatment, early paclitaxel treatment, surgery).
On-study Eligibility Form	Once before randomization.
On-study Pathology Form	Once before randomization.
MammaPrint Form	Agendia will complete before randomization.
MRI Volume Form	For each MRI done for the study (pretreatment, early paclitaxel treatment, inter-regimen, presurgery).
Blood Specimen Form	For each blood collection done for the study (pretreatment, early paclitaxel treatment, inter-regimen, presurgery).
Response Evaluation Form	Before baseline core biopsy, before first AC infusion, presurgery.
Baseline Symptom Form	Within 2 weeks after signing screening consent.

CONFIDENTIAL

Form	When form is to be completed
Lab and Test Form	Baseline: Laboratory blood test and ECHO/MUGA Treatment: Laboratory blood test for each paclitaxel*,AC cycle. ECHO/MUGA every 3 months for HER2+ participants (or as required for investigational agents).
Randomization Form	Once after participant has been randomized
AE Form	Paclitaxel Regimen*: cycle 1, 4, 7, 10<br>AC Regimen: cycle 1, 2, 3, 4 (study cycles 13–16).<br>Surgery: within 2 weeks before surgery.<br>Follow-up:30 days, 6 and 12 months post-surgery.
Chemo Treatment Form	Every treatment:<br>Paclitaxel*: cycle 1–12<br>AC: cycle 1-4 (study cycles 13-16)
Chemo Summary From	Once after neoadjuvant chemotherapy is complete
Post Surgery Summary Form	After primary surgery following neoadjuvant chemotherapy; update RCB if second surgery has tumor present.
Follow-up Form	Every 6 months for 5 years from date of initial surgery and at death.
Lost to Follow-up Form and<br>No Longer Lost to Follow-Up Form	As needed.
Off-study Form	As needed.
Protocol Violation Form	As needed.

*For participants randomized to an investigational arm that does not include paclitaxel, refer to agent-specific appendix for additional submission guidelines. (Appendices D-N)

NOTE: If a participant withdraws from the Screening Phase, complete all screening CRFs up to the point of withdrawal and complete the Randomization CRF indicating why the participant withdrew (see TRANSCEND user's manual in MOP for more details).

NOTE: If a participant is approached for the study but declines to join the study, see TRANSCEND user's manual in MOP for how to document this.

12.3 Source Documents

All source documents will be maintained at the investigational sites in the TRANSCEND clinical trial data capture system, as specified in the TRANSCEND user's manual. Participants' research charts or electronic medical records containing the source documents, including laboratory records for verification of eligibility and data to confirm molecular classification, as well as other data which will be entered into the eCRFs. As instructed, source documents will be de-identified to maintain participant confidentiality, digitized, and electronically stored in the eCRF as part of the TRANSCEND database. The source documents will be used for off-site quality control and verification by the DCC.

12.4 Data and Safety Monitoring Plan

A DSMB has been formed to assure participant safety in this clinical trial. As outlined in the I-SPY 2 TRIAL DSMB Charter, DSMB members will also have additional responsibility for assurance that the trial is conducted to a high standard, and they may be involved in conduct and interpretation of data analyses for efficacy in addition to their primary responsibility for participant safety. The responsibilities of this group include reviewing quantitative recruitment and compliance progress for the study, and recommending modifications of the trial protocol and/or administrative structure in the event these goals are not met. The committee will also review tabulated aggregate toxicity and endpoint data. The

CONFIDENTIAL

committee will submit written recommendations on the progress of the study to the study Principal Investigator and Biomarkers Consortium Project Team.

The DSMB will include a panel of experts recruited from outside of the institutions involved in this study. The DSMB will meet monthly during the study.

12.5 QLHC or FDA Monitoring

QLHC (or their designee), the lead clinical site (UCSF), or FDA may monitor/audit various aspects of the study. These monitors will be given access to facilities, databases, supplies and records to review and verify data pertinent to the study.

12.6 Record Retention

Clinical records for all participants, including CRFs, all source documentation (containing evidence to study eligibility, history and physical findings, laboratory data, results of consultations, *etc.*), as well as IRB records and other regulatory documentation, will be retained by the investigator in a secure storage facility in compliance with Health Insurance Portability and Accountability Act (HIPAA), Office of Human Research Protections (OHRP), FDA regulations and guidances, and NIH requirements unless the standard at the site is more stringent. The records for all studies performed under an IND will be maintained, at a minimum, for two years after the approval of a New Drug Application (NDA). QLHC will be notified prior to the planned destruction of any materials. The records should be accessible for inspection and copying by authorized persons of the FDA. If any part of the study is done outside of the US, applicable regulatory requirements for the specific country participating in the study also apply.

12.7 Clinical Trials Agreement

Investigational agents are provided under a Clinical Trials Agreement (CTA) between Agent Manufacturer and the QLHC.

Data, Intellectual Property, and publications are available in the I-SPY 2 Project Plan available on the I-SPY 2 website: www.ispy2trial.org.

CONFIDENTIAL

13. I-SPY 2 STATISTICAL CONSIDERATIONS

13.1 Sample Size/Accrual Rate

For any given agent, a minimum of 20 and a maximum of 120 participants will accrue prior to agent being dropped or graduated from the trial. (If assignment of a regimen is to be restricted to patients with tumors that are either HER2+ or HER2– then the maximum total sample size for that regimen is 75). Under a Master IND, new agents will replace the agents that leave the trial. 20% of participants will be randomized to a control arm. Up to 25 sites will participate with an anticipated minimum monthly accrual of 15–20, or 180–240 per year. We anticipate that I-SPY 2 will be a standing trial constantly replacing agents leaving the trial with new agents.

13.2 Randomization and Stratification Using Biomarker Signatures

Upon entry to the trial, participants will be categorized according to their disease subtypes based on three standard biomarkers: hormone receptor status (both ER– and PgR–, either ER+ or PgR+, HER2 status (normal [–], positive [+]), and MammaPrint status High1 [MP–] or High2 [MP+]). MammaPrint High 1 and High 2 are determined by the predefined median cut-point of I-SPY 1 participants who fit the eligibility criteria for I-SPY 2. Therefore, there are eight (= 2x2x2) subtypes possible. The goal of the trial is to determine for which disease subtypes—if any—each experimental regimen is an improvement over control therapy. Table 1 shows the eight possible subtypes with prevalences observed in I-SPY 1.

Table 13.1 Prevalence of Eight Disease Subtypes in I-SPY 1

	**MammaPrint High 1 (MP–)**		**MammaPrint High 2 (MP+)**	
	**Hormone Receptor+**	**Hormone Receptor–**	**Hormone Receptor+**	**Hormone Receptor–**
**HER2+**	**16%**	**7%**	**4%**	**10%**
**HER2–**	**23%**	**6%**	**6%**	**28%**

Limited sample sizes and low prevalences make it impossible to carry out inferences within disease subtypes considered separately [34, 49]. For example, it would be impossible to decide that a regimen benefits participants with specifically HR+, HER2+, MP+ tumors, because the prevalence of such tumors in I-SPY 1 was only 4%. Moreover, such a small subpopulation would not be of marketing interest to any collaborating pharmaceutical company. Further, if a regimen were effective in HR+, HER2+, MP– tumors, HR–, HER2+, MP– tumors, and HR–, HER2+, MP+ tumors, it would almost certainly be effective in HR+, HER2+, MP+ tumors. A limited number of subsets of disease subtypes are of scientific and marketing interest; we call these “biomarker signatures.” We consider 10 signatures, listed in Table 13.2, with prevalences estimated from I-SPY 1. “X” indicates that the respective disease subtype is included in each signature.

CONFIDENTIAL

Table 13.2 Ten Candidate Biomarker Signatures

Biomarker Signature	Types (Hormone Receptor, HER2, MP)								Estimated prevalence
	+++	++–	+–+	+– –	–++	–+–	– –+	– – –	
1 (All)	X	X	X	X	X	X	X	X	100%
2 (HR+)	X	X	X	X					49%
3 (HR–)					X	X	X	X	51%
4 (HER2+)	X	X			X	X			37%
5 (HER2–)			X	X			X	X	63%
6 (MammaPrint+)	X		X		X		X		48%
7 (– –)*							X	X	34%
8 (–+)					X	X			17%
9 (++)	X	X							20%
10 (+–)			X	X					29%

*Signature #7 is called "triple negative" because ER, PgR, and HER2 are all negative.
Estimated prevalences are from I-SPY 1 (Table 1).

Some signatures overlap. Indeed, some signatures are subsets of other signatures. Signature #1 contains every other signature as a subset. Signature #10 is relatively small and is contained in Signatures #2 and #5 as well as #1.

A participant's category is the subtype of her tumor—the eight possible subtypes (for driving treatment assignment in this trial) shown in Table 13.1. Assignments to therapy will be on the basis of subtype using the current information about the efficacy of the various regimens for that subtype (see analysis of covariance in Appendix A for I-SPY 2 Statistical Considerations). Experimental regimens will be evaluated for their efficacy relative to control within individual subtypes (see analysis of covariance in the (see Appendix A for I-SPY 2 Statistical Considerations). However, the utility of each treatment regimen will be evaluated only for the 10 biomarker signatures shown in Table 13.2 and not within individual subtypes.

Some of the 10 possible signatures may be inappropriate for a particular experimental regimen. A pharmaceutical company collaborator may request that its agent not be used for some subtypes. Should the Agents Working Group agree, the agent will be a candidate for the trial only if the subtypes considered form one of the signatures in Table 13.2. For example, an agent that targets HER2 may not be appropriate for participants with HER2– disease. Such an agent would have Signature #4 as its "base signature." It would be evaluated for Signatures #4, #8, and #9 only. Agents entering the trial without restrictions have Signature #1 as their base signature.

It is theoretically possible to restrict signatures to those that are highly prevalent. For example, a company might request that its agent be evaluated for Signature #1 only. A consequence would be that if the agent does poorly (relative to control) for HR+ tumors, it may be dropped from the trial for lack of efficacy even if it shows a benefit for HR– tumors. However, if signature #3 (HR–) had been allowed in addition to signature #1, the agent might continue in the trial but would be not used for participants who are HR+. Despite this theoretical possibility, we specifically disallow such restrictions. In particular, if an agent is open to being assigned to any participant subtype, it will be evaluated on the basis of all 10 possible signatures.

The "control base signature" is #1 (all participants) in the sense that throughout the trial, control will have a positive assignment probability for each biomarker type.

CONFIDENTIAL

Any number of experimental regimens may be considered simultaneously in the trial, however, for practical purposes the maximum number of experimental regimens considered at any given time will be limited to 5. A smaller number is likely, especially early in the trial. Regimens may be added over time by the I-SPY 2 Agent Selection Committee (after review and approval by the Independent Agent Selection Committee). In deciding whether to add a regimen, the I-SPY 2 Agent Selection Committee will consider the trial's accrual rate and the number of regimens currently being considered for each of the eight biomarker subtypes. A tentative guideline is that no experimental regimen should be under active consideration (being assigned to participants) for more than 18 months, and preferably less. If the present and projected accrual and the current number of regimens in the trial mean that this goal will not be met, a new regimen should not be added.

13.3 Primary Endpoint and Probability of Success by Biomarker Signature

Participant assignment to one of the regimens in the trial will be randomized and adaptive. Namely, regimens that are performing better for the participant's biomarker type (assessed based on modeling described below) will have a greater assignment probability. A consequence is that participants in the trial are likely to receive better therapy and the better-performing regimens will proceed more rapidly through the trial. Regimens that perform sufficiently poorly will be dropped from the trial, as described below.

Continuously throughout the trial, each regimen's (Bayesian predictive) probability of being successful in a phase 3 confirmatory trial will be calculated for each biomarker signature [34]. These probabilities will be used in making trial decisions (recommendations to the trial's DSMB), as follows:

- A regimen will be dropped from the trial for futility should its predictive probability drop sufficiently low for all biomarker signatures. A minimum of 20 participants will be enrolled on a regimen before dropping a regimen.
- A regimen will graduate from the trial should one or more of its predictive probabilities reach a sufficiently high level. A minimum of 60 participants will be enrolled on a regimen before it can graduate. The exception is when assignment to an experimental regimen is restricted to patients with tumors that are either HER2+ or HER2– then the minimum number of patients assigned to the regimen is 35.
- If the maximum sample size of 120 participants assigned to a regimen (over all biomarker types) is reached, assignments to the regimen will end. The exception is when assignment of a regimen is to be restricted to patients with tumors that are either HER2+ or HER2– then the maximum total sample size for that regimen is 75.

Six months after a regimen graduates or when the maximal sample size is achieved, all participants are expected to have had surgery and the primary endpoint of pCR assessed. At that time the Bayesian predictive probabilities of a successful phase 3 trial for all biomarker signatures will be provided to the appropriate collaborating companies.

13.4 Design Algorithm Overview

The primary endpoint in the trial is pCR. We will use Bayesian logistic regression to model the relationship between six-month pCR rate and the predictor variables—including treatment and biomarkers HR, HER2, and MP statuses. This model will account for the control treatment effect (depending on biomarker subtype), baseline biomarker type, and for the possibility of an interaction between biomarker and experimental treatment. In addition, we also include terms that allow the interaction of biomarkers with each other. By including an interaction term for each experimental

CONFIDENTIAL

treatment and biomarkers, we can identify treatment effects for each potential signature. This model is described in the attached Appendix A for I-SPY 2 Statistical Considerations—including the mathematical details.

We use an outcome-adaptive randomization trial design. The assignment of a participant to therapy will depend on all available data that will be updated on a regular basis. The goal is to learn as rapidly as possible which treatments are effective for which biomarker types [34]. Since pCR is assessed at surgery which will be approximately six months after treatment is initiated, waiting until each participant's outcome has been assessed is less than ideal. To enable using earlier information about the primary endpoint, we will employ a longitudinal model of outcome based on MRI measurements assessed at baseline, end of week 3 after starting paclitaxel, end of week 12 after starting paclitaxel, and end of doxorubicin/cyclophosphamide (weeks 28–30). These measurements are not perfectly predictive of pCR, but are correlated with it. Our longitudinal model will incorporate the empirical information about the predictability of pCR from MRI measurements over time and will be based on the longitudinal information from I-SPY 1 results as a prior distribution. The MRI measurements are not endpoints in themselves but serve as "auxiliary markers" for the primary endpoint of pCR [34].

The logistic model of pCR (see Appendix A for I-SPY 2 Statistical Considerations) will be used to assess the performance of experimental treatments relative to control and by biomarker type. Throughout the trial, decisions must be made for each experimental treatment to either "graduate" it and recommend progressing to phase 3, terminate it for futility, or continue it in the trial to accrue more information. Moreover, when an experimental therapy is continued in the trial, its assignment probabilities by biomarker type must be updated based on currently available results, including MRI measurements. This updating is based on the logistic model. Determining the course of action utilizes the predictive probability of future success in a phase 3 study. If the predictive probability of future success is sufficiently high, the regimen will "graduate" and its various signature predictive probabilities reported.

Information from I-SPY 1 will provide a basis for building the prior distribution for the control pCR rate, the baseline biomarker effects, and longitudinal model parameters. We will analyze the data from the I-SPY 1 clinical trial with the model described in Appendix A for I-SPY 2 Statistical Considerations, and obtain I-SPY 1 posterior distributions for the standard of care effect, baseline biomarker effects, and longitudinal model effects. For the current trial, we assume that prior distributions for the model parameters are normally distributed with means equal to the means obtained for the analysis of the I-SPY 1 data. The standard deviations of these distributions will be suitably inflated in recognition of the possible differences in the two trial populations.

13.5 Assessing Operating Characteristics of the Design via Simulation

The false-positive rate is the major scientific/inferential stumbling block in a study investigating the benefits of many treatment arms in the presence of many biomarker profiles [34]. False positives must be controlled. On the other hand, they cannot be controlled so tightly as to dramatically lower the true-positive rate. Since the design used in the trial will be completely prescribed in advance, we can address these issues and related design performance issues via simulation.

Simulations require inputs. We call each set of inputs a "scenario." This is a specification of the pCR rates for each regimen (including control) and for each of the eight biomarker subtypes.

We consider a variety of possible scenarios ranging from very pessimistic, in which the null hypothesis of no benefit holds for every regimen and every signature considered, to optimistic cases in which several of the regimens are truly effective, and for several signatures each. For each scenario, and following the

CONFIDENTIAL

design we build, we enter participants into a virtual trial and simulate outcomes for them. When the "trial" is over we record various summaries of the trial results, including the duration of the period in which participants were randomized to each experimental regimen, whether the results were "positive" and for which regimens, numbers of participants within the various biomarker types who were assigned to each regimen, and the predictive probability that each regimen would be successful in a phase 3 study. We repeat this trial simulation procedure 1000 times to find the operating characteristics of the design.

The results of the simulation study are presented below. For each scenario, we report the average number of participants on the control and each agent, average time from the first participant to the last participant treated with each agent, and various probabilities of "graduation". As part of the simulation process, we have calibrated the decision cutoffs to obtain a 10% false-positive rate. The Technical Details section below describes in detail the statistical model and the decision rules implemented in the design.

We assume that each experimental regimen will be assigned a maximum of 120 participants. If assignment of a regimen is to be restricted to patients with tumors that are either HER2+ or HER2– then the maximum total sample size for that regimen is 75.

In the scenarios below when a treatment provides a benefit, the true pCR rate is bolded and has a log-odds-ratio of 1.5 compared to the standard treatment. The results for each scenario are presented in three tables. The first table, labeled "True pCR rates", provides the true pCR rates as well as the "true" signature for a therapy that provides benefit. The second table givens the average number of participants enrolled in each subtype for each arm and the total enrolled to each arm. The third table provides the "Graduation" probabilities for each signature. Since an arm may graduate for a subset of the population, we cannot simply report a single probability of graduating but rather categorize the probability of graduation as follows:

P1 = Pr(Graduating for the true signature)—*e.g.*, we graduate a therapy for the HR+ signature when the therapy benefits only the HR+ participants.

P2 = Pr(Graduating for a signature that contains all subtypes that benefit)—*e.g.*, for a therapy that benefits the HR+ participants, we graduate the therapy for the HR+ signature or the All signature.

P3 = Pr(Graduating for a signature that contains only subtypes that benefit)—*e.g.*, for a therapy that benefits the HR+ participants we graduate the therapy for the HR+ or the (++) signature.

P4 = Pr(Graduating for a signature that contains at least one subtype that benefits)—*e.g.*, for a therapy that benefits the HR+ participants, we graduate the therapy for any signature that contains a subtype also contained in the HR+ signature.

P5 = Pr(Graduating for a signature that contains only subtypes that do NOT benefit)—*e.g.*, for a therapy that benefits the HR+ we graduate the HR– signature. This is a false positive conclusion.

How well the design does at graduating therapies depends on the increase in pCR rate as well as the size of the signature for which it provides an improvement. We consider a signature larger than another signature if it has a larger estimated prevalence. For example, the HER2– signature is larger than the HER2+ signature because it has a larger prevalence, even though the two signatures contain the same number of subtypes (Table 1). We begin with a few key points for select scenarios, then provide an overall interpretation of how the design performs.

CONFIDENTIAL

In general, as the prevalence of the true signature increases so does the probability that the design graduates the treatment for the true signature or graduates it for any signature. The probability of graduating an arm for the true signature ranges from 0.76 for the triple negative signature (prevalence = 34%) to 0.86 for the All signature (prevalence = 100%) and the probability of graduating an arm for a signature that contains at least one subtype that benefits ranges from 0.87 for the triple negative signature to 0.99 for the All signature. In contrast, the prevalence of the true signature is inversely related to the average time an arm remains in the trial and the average sample size for an arm. That is, as the prevalence increase from 34% to 100%, the average time in the trial decreases from 16.3 months to 12.9 months and the average sample size for the experimental arm decreases from 83 to 74. In other words, treatments that benefit a larger portion of the population are more likely to graduate sooner and with fewer participants than treatments that benefit a smaller portion of the population. In the scenarios that have two treatments providing a benefit, we observed little reduction in the design's performance when the two treatments have the same true signature or signatures that overlapped in a few subtypes; see scenarios 7 and 9. In scenarios where E1 and E2 had true signatures that did not overlap a slight improvement was observed.

In Scenario 1, none of the experimental treatments provide an improvement over the standard treatment. The average number of participants enrolled per experimental treatment is 73.4 and the average time each therapy remains in the trial is about 17 months. The probability that we make an error and graduate an experimental treatment is approximately 10%. That is, the false-positive rate is approximately 10%.

In Scenario 2, E1 provides an improvement for all participants and as expected E1 graduates very quickly from the trial in about 13 months with an average of 74.2 participants treated. The design graduates E1 86% of the time for the true signature and 99% of the time graduates for a signature that contains only subtypes that benefit. The average sample size for E1 is nearly equal to all other treatments because the adaptive randomization assigns a larger portion of the participants to it, thus graduating it quickly whereas the other treatments remain in the study accruing participants for a longer duration. During the time that E1 is in the study it receives approximately half of the participants that enter the study.

In Scenario 3, the true signature is HER2+, which has an estimated prevalence of 37%. Since the prevalence for the true signature is low, this is a difficult scenario. The design graduates E1 for the HER2+ 76.6% of the time and graduates the arm for a signature that contains at least one subtype that benefits 87% of the time.

In Scenario 4 the true signature for E1 is HR+, which has an estimated prevalence of 49%. On average, E1 remains in the trial for 15.7 months and enrolls 79.8 participants. The design graduates E1 for the true HR+ signature 72% of the time and graduates E1 for a signature that contains at least one subtype that benefits 83% of the time. It is important to note that there a 12% chance of graduating E1 for a signature that contains only subtypes that do not benefit.

In Scenario 5 the true signature for E1 is triple negative, which has an estimated prevalence of 33%. On average, E1 remains in the trial for 16 months and enrolls 82.8 participants. The design graduates E1 for the true triple negative signature 74% of the time and graduates E1 for a signature that contains at least one subtype that benefits 82% of the time. It is important to note that there a 16% chance of graduating E1 for a signature that contains only subtypes that do not benefit.

In Scenarios 7–9 there is more than one treatment that provides a benefit for the participants. In Scenarios 7 and 9, the true signatures for E1 and E2 were the same or overlapped. If we have two treatments where the true signature is All, the probability of graduating for the true signature does not reduce when compared to a trial with only one treatment that has the true signature of All. However, the treatments do

CONFIDENTIAL

remain in the study for about 1 month longer than they would if there was no other treatment that provided a benefit.

Scenarios 10–13 allow for treatment to enter the study at times other than the start of the trial and have more than experimental treatment. In Scenario 10, E3 enters the study at the start of month 7 and true signature for both E1 and E3 is All. By allowing E3 to enter the study at month 7 rather than the start of the study, the average number of participants enrolled to E3 is reduced and the amount of time E3 remains in the study is reduced. This is due to the fact the participants are randomized to fewer arms and thus more information can accrue in each arm in a shorter period of time. The probability of graduating E3 for the All signature is about 5% higher than it is for E1.

In Scenario 11 E1 enters the study at month 7 and E4 at month 9. The true signature for E2 is HER2+ and E4 is HER2–. This scenario is comparable to Scenario 8 in terms of the signatures the arms provide a benefit for, except that the treatments do not all start at the beginning of the study. The probability of graduating E1 for the HER2+ signature is reduced by about 10% when compared to Scenario 8. This is largely due to the addition of the fourth experimental arm. The performance for the arm that benefits the HER2– (E3) participants is much less because shortly after the arm enters the study, E1 and E2 exit the study.

Table 13.3 Scenario 1, True pCR Rates

	True pCR Rates			
Cell	S	E1	E2	E3
HR+HER2+MP+	0.41	0.41	0.41	0.41
HR+HER2+MP-	0.17	0.17	0.17	0.17
HR+HER2-MP+	0.23	0.23	0.23	0.23
HR+HER2-MP-	0.037	0.037	0.037	0.037
HR-HER2+MP+	0.61	0.61	0.61	0.61
HR-HER2+MP-	0.56	0.56	0.56	0.56
HR-HER2-MP+	0.37	0.37	0.37	0.37
HR-HER2-MP-	0.24	0.24	0.24	0.24
True Signature		None	None	None

Hormone Receptor (HR); MammaPrint High 2 (MP+); MammaPrint High 1 (MP-); Investigational agent 1, 2, 3 (E1, E2, E3)

Table 13.4 Scenario 1, Average Number of Participants by Subtype

**Cell**	**S**	**E1**	**E2**	**E3**
HR+HER2+MP+	1.9	3	3	3
HR+HER2+MP-	7.6	11.7	11.7	11.7
HR+HER2-MP+	2.9	4.5	4.5	4.5
HR+HER2-MP-	10.9	16.9	16.9	16.9
HR-HER2+MP+	4.9	7.4	7.4	7.4
HR-HER2+MP-	3.4	5.1	5.1	5.1
HR-HER2-MP+	13.3	20.5	20.5	20.5
HR-HER2-MP-	2.8	4.3	4.3	4.3
Total	47.8	73.4	73.4	73.4

Table 13.5 Scenario 1, Graduation Probabilities (P)

CONFIDENTIAL

Arm	P1	P2	P3	P4	P5	Ave. Time in Trial
E1	0	0	0	0	0.097	17.2
E2	0	0	0	0	0.097	17.2
E3	0	0	0	0	0.097	17.2

Scenario 2
Prevalence of true signature for E1 = 100%

True pCR Rates

Cell	S	E1	E2	E3
HR+HER2+MP+	0.41	**0.757**	0.41	0.41
HR+HER2+MP-	0.17	**0.479**	0.17	0.17
HR+HER2-MP+	0.23	**0.572**	0.23	0.23
HR+HER2-MP-	0.037	**0.147**	0.037	0.037
HR-HER2+MP+	0.61	**0.875**	0.61	0.61
HR-HER2+MP-	0.56	**0.85**	0.56	0.56
HR-HER2-MP+	0.37	**0.724**	0.37	0.37
HR-HER2-MP-	0.24	**0.59**	0.24	0.24
True Signature		All	None	None

Average Number of Participants

Cell	S	E1	E2	E3
HR+HER2+MP+	1.9	**3.1**	2.9	2.9
HR+HER2+MP-	7.6	**11.5**	11.5	11.5
HR+HER2-MP+	2.8	**4.4**	4.3	4.3
HR+HER2-MP-	10.7	**16.1**	16.9	16.9
HR-HER2+MP+	4.7	**7.7**	7.3	7.3
HR-HER2+MP-	3.4	**5**	5.2	5.2
HR-HER2-MP+	13	**22.1**	19.2	19.2
HR-HER2-MP-	2.9	**4.4**	4.3	4.3
Total	47	74.2	71.4	71.4

Graduation Probabilities

Arm	P1	P2	P3	P4	P5	Ave. Time in Trial
E1	0.86	0.86	0.993	0.993	0	12.9
E2	0	0	0	0	0.095	17.2
E3	0	0	0	0	0.095	17.2

CONFIDENTIAL

Scenario 3

Prevalence of true signature for E1 = 37%

	True pCR Rates			
**Cell**	**S**	**E1**	**E2**	**E3**
HR+HER2+MP+	0.41	**0.757**	0.41	0.41
HR+HER2+MP-	0.17	**0.479**	0.17	0.17
HR+HER2-MP+	0.23	0.23	0.23	0.23
HR+HER2-MP-	0.037	0.037	0.037	0.037
HR-HER2+MP+	0.61	**0.875**	0.61	0.61
HR-HER2+MP-	0.56	**0.85**	0.56	0.56
HR-HER2-MP+	0.37	0.37	0.37	0.37
HR-HER2-MP-	0.24	0.24	0.24	0.24
True Signature		HER2+	None	None

	Average Number of Participants			
**Cell**	**S**	**E1**	**E2**	**E3**
HR+HER2+MP+	2	**3.9**	2.6	2.6
HR+HER2+MP-	7.7	**16.2**	9.6	9.6
HR+HER2-MP+	2.9	4.4	4.4	4.4
HR+HER2-MP-	10.9	17.7	16.6	16.6
HR-HER2+MP+	4.9	**9.7**	6.4	6.4
HR-HER2+MP-	3.4	**6.8**	4.4	4.4
HR-HER2-MP+	13.3	20	20.5	20.5
HR-HER2-MP-	2.9	4.5	4.3	4.3
Total	47.9	83.1	68.8	68.8

			Graduation Probabilities			
**Arm**	**P1**	**P2**	**P3**	**P4**	**P5**	**Ave. Time in Trial**
E1	0.766	0.783	0.816	0.873	0.062	16.3
E2	0	0	0	0	0.096	17.3
E3	0	0	0	0	0.096	17.3

CONFIDENTIAL

Scenario 4

Prevalence of true signature for E1 = 49%

True pCR Rates				
**Cell**	**S**	**E1**	**E2**	**E3**
HR+HER2+MP+	0.41	**0.757**	0.41	0.41
HR+HER2+MP-	0.17	**0.479**	0.17	0.17
HR+HER2-MP+	0.23	**0.572**	0.23	0.23
HR+HER2-MP-	0.037	**0.147**	0.037	0.037
HR-HER2+MP+	0.61	0.61	0.61	0.61
HR-HER2+MP-	0.56	0.56	0.56	0.56
HR-HER2-MP+	0.37	0.37	0.37	0.37
HR-HER2-MP-	0.24	0.24	0.24	0.24
True Signature		HR+	None	None

Average Number of Participants				
**Cell**	**S**	**E1**	**E2**	**E3**
HR+HER2+MP+	2	**3.6**	2.7	2.7
HR+HER2+MP-	7.6	**14.7**	10.3	10.3
HR+HER2-MP+	2.8	**5.1**	4.0	4.0
HR+HER2-MP-	10.9	**21.2**	14.8	14.8
HR-HER2+MP+	4.8	7.1	7.6	7.6
HR-HER2+MP-	3.4	5	5.2	5.2
HR-HER2-MP+	13.4	19.1	21.1	21.1
HR-HER2-MP-	2.7	4.2	4.4	4.4
Total	47.7	79.8	70.0	70.0

Graduation Probabilities						
**Arm**	**P1**	**P2**	**P3**	**P4**	**P5**	**Ave. Time in Trial**
E1	0.721	0.758	0.791	0.834	0.123	15.7
E2	0	0	0	0	0.10	17.3
E3	0	0	0	0	0.10	17.3

CONFIDENTIAL

Scenario 5

Prevalence of true signature for E1 = 34%

True pCR Rates

Cell	S	E1	E2	E3
HR+HER2+MP+	0.41	0.41	0.41	0.41
HR+HER2+MP-	0.17	0.17	0.17	0.17
HR+HER2-MP+	0.23	0.23	0.23	0.23
HR+HER2-MP-	0.037	0.037	0.037	0.037
HR-HER2+MP+	0.61	0.61	0.61	0.61
HR-HER2+MP-	0.56	0.56	0.56	0.56
HR-HER2-MP+	0.37	**0.724**	0.37	0.37
HR-HER2-MP-	0.24	**0.59**	0.24	0.24
True Signature		(--) Triple Negative	None	None

Average Number of Participants

Cell	S	E1	E2	E3
HR+HER2+MP+	1.9	2.7	3.0	3.0
HR+HER2+MP-	7.7	10.2	12.5	12.5
HR+HER2-MP+	2.9	4.9	4.3	4.3
HR+HER2-MP-	11	17.6	16.5	16.5
HR-HER2+MP+	4.8	7.7	7.2	7.2
HR-HER2+MP-	3.4	5	5.3	5.3
HR-HER2-MP+	13.4	**28.8**	16.5	16.5
HR-HER2-MP-	2.8	**5.8**	4.0	4.0
Total	48	82.8	69.1	69.1

Graduation Probabilities

Arm	P1	P2	P3	P4	P5	Ave. Time in Trial
E1	0.743	0.797	0.743	0.822	0.16	16
E2	0	0	0	0	0.088	17.2
E3	0	0	0	0	0.088	17.2

CONFIDENTIAL

Scenario 6
Prevalence of true signature for E1 = 48%

	True pCR Rates			
**Cell**	**S**	**E1**	**E2**	**E3**
HR+HER2+MP+	0.41	**0.757**	0.41	0.41
HR+HER2+MP-	0.17	0.17	0.17	0.17
HR+HER2-MP+	0.23	**0.572**	0.23	0.23
HR+HER2-MP-	0.037	0.037	0.037	0.037
HR-HER2+MP+	0.61	**0.875**	0.61	0.61
HR-HER2+MP-	0.56	0.56	0.56	0.56
HR-HER2-MP+	0.37	**0.724**	0.37	0.37
HR-HER2-MP-	0.24	0.24	0.24	0.24
True Signature		MP2+	None	None

	Average Number of Participants			
**Cell**	**S**	**E1**	**E2**	**E3**
HR+HER2+MP+	2	**3.6**	2.7	2.7
HR+HER2+MP-	7.5	11	12.3	12.3
HR+HER2-MP+	2.9	**5.6**	3.8	3.8
HR+HER2-MP-	11.1	16.9	17.0	17.0
HR-HER2+MP+	4.8	**9.4**	6.4	6.4
HR-HER2+MP-	3.3	4.7	5.4	5.4
HR-HER2-MP+	13.5	**29.4**	16.1	16.1
HR-HER2-MP-	2.8	4.6	4.3	4.3
Total	47.9	85.1	67.8	67.8

			Graduation Probabilities			
**Arm**	**P1**	**P2**	**P3**	**P4**	**P5**	**Ave. Time in Trial**
E1	0.847	0.855	0.847	0.924	0	15.8
E2	0	0	0	0	0.095	17.3
E3	0	0	0	0	0.095	17.3

CONFIDENTIAL

Scenario 7
Prevalence of true signature for E1 = E2 = 100%, arm E2 enters the study at month 7.

True pCR Rates

Cell	S	E1	E2	E3
HR+HER2+MP+	0.41	**0.757**	**0.757**	0.41
HR+HER2+MP-	0.17	**0.479**	**0.479**	0.17
HR+HER2-MP+	0.23	**0.572**	**0.572**	0.23
HR+HER2-MP-	0.037	**0.147**	**0.147**	0.037
HR-HER2+MP+	0.61	**0.875**	**0.875**	0.61
HR-HER2+MP-	0.56	**0.85**	**0.85**	0.56
HR-HER2-MP+	0.37	**0.724**	**0.724**	0.37
HR-HER2-MP-	0.24	**0.59**	**0.59**	0.24
True Signature		All	All	None

Average Number of Participants

Cell	S	E1	E2	E3
HR+HER2+MP+	1.9	**2.9**	**2.9**	2.8
HR+HER2+MP-	7.5	**11.6**	**11.6**	10.9
HR+HER2-MP+	2.8	**4.4**	**4.4**	4.1
HR+HER2-MP-	10.7	**16.3**	**16.3**	16.1
HR-HER2+MP+	4.6	**7.3**	**7.3**	7.2
HR-HER2+MP-	3.2	**5.0**	**5.0**	5.1
HR-HER2-MP+	12.8	**20.8**	**20.8**	18.4
HR-HER2-MP-	2.7	**4.4**	**4.4**	4.1
Total	46.1	**72.6**	**72.6**	68.6

Graduation Probabilities

Arm	P1	P2	P3	P4	P5	Ave. Time in Trial
E1	0.86	0.86	0.99	0.99	0	13.8
E2	0.86	0.86	0.99	0.99	0	13.8
E3	0	0	0	0	0.072	17.2

CONFIDENTIAL

Scenario 8
Prevalence of true signature for E1 = 37% and E2 = 67%

True pCR Rates

Cell	S	E1	E2	E3
HR+HER2+MP+	0.41	**0.757**	0.41	0.41
HR+HER2+MP-	0.17	**0.479**	0.17	0.17
HR+HER2-MP+	0.23	0.23	**0.572**	0.23
HR+HER2-MP-	0.037	0.037	**0.147**	0.037
HR-HER2+MP+	0.61	**0.875**	0.61	0.61
HR-HER2+MP-	0.56	**0.85**	0.56	0.56
HR-HER2-MP+	0.37	0.37	**0.724**	0.37
HR-HER2-MP-	0.24	0.24	**0.59**	0.24
True Signature		HER2+	HER2-	None

Average Number of Participants

Cell	S	E1	E2	E3
HR+HER2+MP+	2	**3.9**	2.6	2.5
HR+HER2+MP-	7.6	**16.9**	9	9.6
HR+HER2-MP+	2.9	3.9	**5.4**	3.8
HR+HER2-MP-	10.8	16.3	**19.7**	14.7
HR-HER2+MP+	4.8	**9.6**	6.3	6.2
HR-HER2+MP-	3.5	**7.1**	3.8	4.5
HR-HER2-MP+	13.3	16.8	**27.3**	17.2
HR-HER2-MP-	2.9	4	**5.3**	3.8
Total	47.7	78.6	79.2	62.3

Graduation Probabilities

Arm	P1	P2	P3	P4	P5	Ave. Time in Trial
E1	0.802	0.81	0.843	0.888	0.046	16.6
E2	0.759	0.781	0.893	0.93	0.089	15.2
E3	0	0	0	0	0.09	17.3

CONFIDENTIAL

Scenario 9
Prevalence of true signature for E1 = 49% and E2 = 63%

True pCR Rates

Cell	S	E1	E2	E3
HR+HER2+MP+	0.41	**0.757**	0.41	0.41
HR+HER2+MP-	0.17	**0.479**	0.17	0.17
HR+HER2-MP+	0.23	**0.572**	**0.572**	0.23
HR+HER2-MP-	0.037	**0.147**	**0.147**	0.037
HR-HER2+MP+	0.61	0.61	0.61	0.61
HR-HER2+MP-	0.56	0.56	0.56	0.56
HR-HER2-MP+	0.37	0.37	**0.724**	0.37
HR-HER2-MP-	0.24	0.24	**0.59**	0.24
True Signature		HR+	HER2-	None

Average Number of Participants

Cell	S	E1	E2	E3
HR+HER2+MP+	1.9	**3.6**	2.7	2.5
HR+HER2+MP-	7.8	**15.5**	9.4	10.3
HR+HER2-MP+	2.8	**4.7**	**5**	3.6
HR+HER2-MP-	11	**19.6**	**17.6**	13.3
HR-HER2+MP+	4.8	7.3	7.4	7.5
HR-HER2+MP-	3.2	5.3	4.7	5.5
HR-HER2-MP+	13.3	16.5	**28.2**	16.7
HR-HER2-MP-	2.8	3.8	**5.4**	3.8
Total	47.6	76.2	80.4	63.2

Graduation Probabilities

Arm	P1	P2	P3	P4	P5	Ave. Time in Trial
E1	0.67	0.695	0.773	0.802	0.105	16.2
E2	0.741	0.768	0.883	0.927	0.117	15.4
E3	0	0	0	0	0.09	17.4

Arms enter the study at times other than the start of the trial.

CONFIDENTIAL

Scenario 10

Prevalence of true signature for E1 = E3 = 100%, arm E3 enters the study at month 7.

		True pCR Rates		
**Cell**	**S**	**E1**	**E2**	**E3**
HR+HER2+MP+	0.41	**0.757**	0.41	**0.757**
HR+HER2+MP-	0.17	**0.479**	0.17	**0.479**
HR+HER2-MP+	0.23	**0.572**	0.23	**0.572**
HR+HER2-MP-	0.037	**0.147**	0.037	**0.147**
HR-HER2+MP+	0.61	**0.875**	0.61	**0.875**
HR-HER2+MP-	0.56	**0.85**	0.56	**0.85**
HR-HER2-MP+	0.37	**0.724**	0.37	**0.724**
HR-HER2-MP-	0.24	**0.59**	0.24	**0.59**
True Signature		All	None	All

		Average Number of Participants		
**Cell**	**S**	**E1**	**E3**	**E2**
HR+HER2+MP+	2.2	**2.9**	2.9	**2.9**
HR+HER2+MP-	8.9	**11**	11.8	**12**
HR+HER2-MP+	3.3	**4.2**	4.4	**4.5**
HR+HER2-MP-	12.6	**15**	17.4	**17.7**
HR-HER2+MP+	5.6	**7.3**	7.6	**7.1**
HR-HER2+MP-	3.9	**4.9**	5.6	**4.9**
HR-HER2-MP+	15.3	**20.4**	19.6	**21**
HR-HER2-MP-	3.3	**4.2**	4.5	**4.4**
Total	55.1	70	73.9	74.6

Graduation Probabilities

**Arm**	**P1**	**P2**	**P3**	**P4**	**P5**	**Ave. Time in Trial**
E1	0.876	0.876	0.995	0.995	0	12.1
E3	0	0	0	0	0.084	17.2
E2	0.918	0.918	0.998	0.998	0	10.3

CONFIDENTIAL

Scenario 11
Prevalence of true signature for E1 = 37% and E3 = 67%. E3 enters the study at month 7 and E4 month 9.

True pCR Rates

Cell	S	E1	E2	E3	E4
HR+HER2+MP+	0.41	**0.757**	0.41	0.41	0.41
HR+HER2+MP-	0.17	**0.479**	0.17	0.17	0.17
HR+HER2-MP+	0.23	0.23	0.23	**0.572**	0.23
HR+HER2-MP-	0.037	0.037	0.037	**0.147**	0.037
HR-HER2+MP+	0.61	**0.875**	0.61	0.61	0.61
HR-HER2+MP-	0.56	**0.85**	0.56	0.56	0.56
HR-HER2-MP+	0.37	0.37	0.37	**0.724**	0.37
HR-HER2-MP-	0.24	0.24	0.24	**0.59**	0.24
True Signature		HER2+	None	HER2-	None

Average Number of Participants

Cell	S	E1	E2	E3	E4
HR+HER2+MP+	2.9	**3.1**	2.6	2.8	4
HR+HER2+MP-	11.6	**13.1**	10.1	10.3	15.6
HR+HER2-MP+	4.3	3.7	4.1	**5.5**	5.5
HR+HER2-MP-	16.7	14.2	15.5	**20.7**	20.4
HR-HER2+MP+	7.4	**8.1**	6.6	6.7	9.8
HR-HER2+MP-	5	**6.1**	4.6	4.3	6.8
HR-HER2-MP+	20.3	16.6	18.7	**27.4**	22.3
HR-HER2-MP-	4.4	3.8	4.2	**5.4**	4.8
Total	72.6	68.8	66.5	83	89.3

Graduation Probabilities

Arm	P1	P2	P3	P4	P5	Ave. Time in Trial
E1	0.696	0.709	0.739	0.809	0.053	16.5
E3	0	0	0	0	0.103	17.2
E2	0.747	0.762	0.884	0.914	0.108	14.7
E4	0	0	0	0	0.138	16.8

CONFIDENTIAL

Scenario 12
Prevalence of true signature for E1 = 37% and E3 = 67%. E3 enters the study at month 6, E4 at month 13, E5 at month 14.

True pCR Rates

Cell	S	E1	E2	E3	E4	E5
HR+HER2+MP+	0.41	**0.757**	0.41	0.41	**0.757**	0.41
HR+HER2+MP-	0.17	**0.479**	0.17	0.17	**0.479**	0.17
HR+HER2-MP+	0.23	**0.572**	**0.572**	0.23	**0.572**	0.23
HR+HER2-MP-	0.037	**0.147**	**0.147**	0.037	**0.147**	0.037
HR-HER2+MP+	0.61	0.61	0.61	0.61	**0.875**	0.61
HR-HER2+MP-	0.56	0.56	0.56	0.56	**0.85**	0.56
HR-HER2-MP+	0.37	0.37	**0.724**	0.37	**0.724**	0.37
HR-HER2-MP-	0.24	0.24	**0.59**	0.24	**0.59**	0.24
True Signature		HR+	HER2-	None	All	None

Average Number of Participants

Cell	S	E1	E2	E3	E4	E5
HR+HER2+MP+	3.5	**3.1**	2.3	2.4	**3**	4
HR+HER2+MP-	13.7	**12.6**	8.3	9.7	**12.2**	15.2
HR+HER2-MP+	5.3	**4.3**	**4.1**	3.6	**4.3**	5.7
HR+HER2-MP-	19.6	**17.6**	**14.9**	13.2	**16.1**	21.3
HR-HER2+MP+	8.6	6.3	6.3	6.8	**7.7**	9.9
HR-HER2+MP-	5.9	4.6	4	4.9	**5.1**	6.9
HR-HER2-MP+	23.6	16.7	**22.2**	17.4	**20.7**	23.1
HR-HER2-MP-	5.1	3.8	**4.7**	3.8	**4.1**	5
Total	85.4	69	66.7	61.7	73.3	91.2

Graduation Probabilities

Arm	P1	P2	P3	P4	P5	Ave. Time in Trial
E1	0.584	0.607	0.681	0.72	0.107	15.7
E2	0.663	0.681	0.819	0.863	0.139	14.7
E3	0	0	0	0	0.091	17.2
E4	0.829	0.829	0.994	0.994	0	12.5
E5	0	0	0	0	0.150	16.5

CONFIDENTIAL

Scenario 13
The following arms were not in the study at the beginning but joined at the following months. E3 at month 6, E4 at month 13, E5 at 17, E6 at 24, E7 at 30, E8 at 30

True pCR Rates

Cell	S	E1	E2	E3	E4	E5	E6	E7	E8
HR+HER2+MP+	0.41	**0.757**	0.41	0.41	**0.757**	0.41	0.41	0.41	**0.757**
HR+HER2+MP-	0.17	**0.479**	0.17	0.17	**0.479**	0.17	0.17	0.17	**0.479**
HR+HER2-MP+	0.23	**0.572**	0.23	**0.572**	**0.572**	0.23	0.23	0.23	0.23
HR+HER2-MP-	0.037	**0.147**	0.037	**0.147**	**0.147**	0.037	0.037	0.037	0.037
HR-HER2+MP+	0.61	0.61	0.61	0.61	**0.875**	0.61	0.61	0.61	**0.875**
HR-HER2+MP-	0.56	0.56	0.56	0.56	**0.85**	0.56	0.56	0.56	**0.85**
HR-HER2-MP+	0.37	0.37	0.37	**0.724**	**0.724**	0.37	**0.724**	0.37	0.37
HR-HER2-MP-	0.24	0.24	0.24	**0.59**	**0.59**	0.24	**0.59**	0.24	0.24
True Signature		HR+	None	HER2-	All	None	Trip-	Null	HER2+

Average Number of Participants

Cell	S	E1	E2	E3	E4	E5	E6	E7	E8
HR+HER2+MP+	4.9	**3.1**	2.5	2.3	**2.8**	2.5	2.6	2.2	**3.9**
HR+HER2+MP-	19.2	**13**	9.7	8.5	**11.3**	10.1	10.2	8.4	**15.3**
HR+HER2-MP+	7.3	**4.4**	3.6	**4.2**	**3.9**	3.5	4.6	4	4.5
HR+HER2-MP-	27.6	**17.7**	13.3	**15.2**	**14.6**	14	16.8	14.8	17.6
HR-HER2+MP+	11.9	6.4	7	6.5	**7.4**	5.9	7.1	5.4	**9**
HR-HER2+MP-	8.3	4.5	5	4.1	**5.1**	4.4	4.8	3.8	**6.4**
HR-HER2-MP+	33.4	16.7	17.6	**22.9**	**19.1**	14.6	**24.1**	16.6	18.1
HR-HER2-MP-	7.2	3.7	4	**4.7**	**4**	3.4	**5.1**	3.5	3.9
Total	119.8	69.4	62.7	68.4	68.3	58.3	75.4	58.7	78.7

Graduation Probabilities

Arm	P1	P2	P3	P4	P5	Ave. Time in Trial
E1	0.613	0.634	0.699	0.742	0.108	15.5
E2	0	0	0	0	0.099	17.2
E3	0.689	0.714	0.846	0.888	0.152	14.2
E4	0.834	0.834	0.994	0.994	0	12
E5	0	0	0	0	0.14	16.8
E6	0.774	0.826	0.774	0.851	0.248	13.5
E7	0	0	0	0	0.074	14.8
E8	0.665	0.684	0.704	0.733	0.13	14.1

CONFIDENTIAL

14. ETHICAL AND REGULATORY CONSIDERATIONS

Regulatory documents are essential to clinical research. They serve to demonstrate the regulatory approval(s) and compliance of the Sponsor, Investigator, Monitor, and IRB with the current federal and state regulations and the International Conference on Harmonization Good Clinical Practice (ICH GCP) guidelines.

Study sites selected for participation in I-SPY 2 TRIAL will be responsible for submitting essential regulatory documents to DCC. The collection of regulatory documents will take place in accordance with applicable ICH GCP guidelines, state and federal regulations. Regulatory documents must be maintained per all applicable institutional and federal regulations. Any and all questions related to regulatory document submission should be directed to the attention of DCC Regulatory as outlined in protocol §14.5. Please see the I-SPY 2 Manual of Operations, §*Essential Regulatory Document Collection Process*, for more detailed information and links for downloading required forms from the I-SPY 2 website.

All study-specific forms (Form FDA 1572 template, Financial Disclosure, Delegation of Responsibilities, Investigator's Brochure Acknowledgment) can be accessed via the I-SPY 2 website at www.ispy2trial.org.

The following documents comprise the essential regulatory document packet required for agent shipment authorization and study site activation.

14.1 Form FDA 1572

Prior to initiating this study at any site, the Principal Investigator will provide an original signed Form FDA 1572 stating that the study will be conducted in compliance with regulations for clinical investigations and listing all the investigators at the organization at each site that will participate in the protocol.

14.2 Other Required Documents

Signed and dated current (within two years) CV/Biosketch for the site Principal Investigator and all subinvestigators listed on Form FDA 1572.

Current professional licenses (where applicable) for the site Principal Investigator and all subinvestigators listed on Form FDA 1572.

Original signed and dated I-SPY 2 Financial Disclosure Form for the site Principal Investigator and all subinvestigators listed on Form FDA 1572.

Certification of Human Subjects Protection Training (NIH or institution-based training program certificate) for the site Principal Investigator and all subinvestigators listed on Form FDA 1572.

Delegation of Responsibilities Log signed by the site Principal Investigator which lists the names and responsibilities of all study staff, including all subinvestigators listed on Form FDA 1572.

Lab certifications(CLIA and CAP) and lab normal ranges for all labs listed on each site's Form FDA 1572.

CONFIDENTIAL

Documentation of Federalwide Assurance (FWA). A print-out of the institutional FWA number may be accessed *via* the OHRP website as follows: http://ohrp.cit.nih.gov/search/fwasearch.aspx?styp=bsc (click the button for FWAs [FWA number]).

Investigator's Brochure Acknowledgment Form signed by the site Principal Investigator.

IRB approval for all QLHC-approved protocol versions, Informed Consent versions (Screening, Treatment, and Supplemental), Investigator's Brochure versions (if applicable) and recruitment materials.

14.3 IRB Approval

Prior to initiating the study and receiving the drug agent(s), the investigators at the organizations must obtain written approval to conduct the study from the appropriate IRB. Should changes to the study become necessary, protocol amendments will be submitted to QLHC according to sponsor Amendment Guidelines. QLHC-approved amended protocol must be approved by the IRB prior to implementation.

As investigational agents move in and out of the trial, protocol amendments will be issued; see §6 for more detail.

14.3.1 IRB Approval Timeline Guidelines

Participating institutions will be notified of the allowable timeframe permitted to get each amendment approved by their institutional IRB. For amendments that include new investigational agents in the trial, see §14.3.1.1.

14.3.1.1 Amendment Containing New Investigational Agent(s)

Major Modification (see §6 for definition): Participating institutions have 60 calendar days to submit and obtain IRB approval. If an institution's IRB approval letter is not received by the sponsor or their authorized designee ≤60 calendar days, accrual at that institution will be placed on hold until institutional IRB approval is obtained and approval letters have been received and processed by the sponsor or their authorized designee.

Minor Modification (see §6 for definition): Participating institutions have 30 calendar days to submit and obtain IRB approval. If an institution's IRB approval letters are not received by the sponsor or their authorized designee ≤30 calendar days, accrual at that institution will be placed on hold until institutional IRB approval is obtained and approval letters have been received and processed by the sponsor or their authorized designee.

14.4 Informed Consent

The I-SPY 2 TRIAL will be utilizing a two-step consent process. All potential study participants will be given a copy of the IRB-approved Screening Informed Consent to review. The clinical investigator and study coordinator will explain all aspects of the study in lay language and answer all questions regarding the study. If the participant decides to participate in the screening phase, she will be asked to sign and date the consent form. Participants that complete the screening phase and are eligible for the treatment phase of the study will be given a copy of the IRB-approved Treatment Informed Consent to review. If the participant is randomized to an investigational agent, the appropriate Supplemental Informed Consent Form will be given to the participant at the same time as the Treatment Informed Consent. The investigator and study coordinator will explain all aspects of the study in lay language and answer all

CONFIDENTIAL

questions regarding the study. If the participant decides to participate in the treatment phase of the study, she will be asked to sign and date the Informed Consent document(s). The study agent(s) will not be released to a participant who has not signed the Treatment and appropriate Supplemental Consent Form documents. Participants who refuse to participate or who withdraw from the study will be treated without prejudice.

Participants are provided the option to allow the use of blood samples and tissues obtained during testing, operative procedures, or other standard medical practices for further research purposes.

Prior to study initiation, the Informed Consent documents must be reviewed and approved by QLHC or their authorized designee, the Biomarkers Consortium Project Team, and the IRB at each organization at which the protocol will be implemented. Any subsequent changes to the informed consent must be approved by QLHC or their authorized designee and the Project Team, and then submitted to each organization's IRB for approval prior to initiation.

14.5 Submission of Regulatory Documents

All regulatory documents may be transmitted *via* email (preferred method) or facsimile with the exception of the following documents for which signed originals must be sent via traceable courier:

- Form FDA 1572
- Financial Disclosure Form

Please refer to the I-SPY 2 Manual of Operations for completion and submission guidelines.

14.6 Other

This trial will be conducted in compliance with the protocol, Good Clinical Practice, and the applicable regulatory requirements.

CONFIDENTIAL

15. FINANCING, EXPENSES, AND/OR INSURANCE

Please refer to study Informed Consents.

CONFIDENTIAL

REFERENCES

1. Valero VV, Buzdar AU, Hortobagyi GN. Locally Advanced Breast Cancer.*Oncologist*.1: 8-17, 1996.
2. Esserman LJ, van't Veer LJ, Perou C, Rutgers EJT, Davis SE. Biology of breast cancers that present as interval cancers and at young age should inform how we approach early detection and prevention. *San Antonio Breast Cancer Symp*.abstr 6034, 2008.
3. Lin C, Moore D, DeMichele A, Ollila D, Montgomery L, Lehman CD, Liu M, Gomez R, Krontiras H, Esserman L. The majority of locally advanced breast cancers are interval cancers. *J Clin Oncol*.27: abstr 1503, 2009.
4. Newman LA, Mamounas EP. Review of breast cancer clinical trials conducted by the National Surgical Adjuvant Breast Project. *Surg Clin North Am*. 87: 279–305, vii, 2007.
5. Loi S, Buyse M, Sotiriou C, Cardoso F. Challenges in breast cancer clinical trial design in the postgenomic era. *Curr Opin Oncol*.16: 536–541, 2004.
6. Berry DA, Cirrincione C, Henderson IC, Citron ML, Budman DR, Goldstein LJ, Martino S, Perez EA, Muss HB, Norton L, Hudis C, Winer EP. Estrogen-receptor status and outcomes of modern chemotherapy for patients with node-positive breast cancer.*JAMA*.295: 1658–1667, 2006.
7. Dancey JE, Chen HX. Strategies for optimizing combinations of molecularly targeted anticancer agents.*Nat Rev Drug Discov*.5: 649–659, 2006 .
8. Baselga J, Perez EA, Pienkowski T, Bell R. Adjuvant trastuzumab: a milestone in the treatment of HER-2-positive early breast cancer. *Oncologist*.11 Suppl 1: 4–12, 2006.
9. Gralow JR, Burstein HJ, Wood W, Hortobagyi GN, Gianni L, von Minckwitz G, Buzdar AU, Smith IE, Symmans WF, Singh B, Winer EP. Preoperative therapy in invasive breast cancer: pathologic assessment and systemic therapy issues in operable disease. *J Clin Oncol*.26: 814–819, 2008.
10. Fisher B, Brown A, Mamounas E, Wieand S, Robidoux A, Margolese RG, Cruz AB, Jr., Fisher ER, Wickerham DL, Wolmark N, DeCillis A, Hoehn JL, Lees AW, Dimitrov NV. Effect of preoperative chemotherapy on local-regional disease in women with operable breast cancer: findings from National Surgical Adjuvant Breast and Bowel Project B-18. *J Clin Oncol*.15: 2483–2493, 1997.
11. Bear HD, Anderson S, Smith RE, Geyer CE, Jr., Mamounas EP, Fisher B, Brown AM, Robidoux A, Margolese R, Kahlenberg MS, Paik S, Soran A, Wickerham DL, Wolmark N. Sequential preoperative or postoperative docetaxel added to preoperative doxorubicin plus cyclophosphamide for operable breast cancer: National Surgical Adjuvant Breast and Bowel Project Protocol B-27. *J Clin Oncol*.24: 2019–2027, 2006.
12. Buchholz TA, Lehman CD, Harris JR, Pockaj BA, Khouri N, Hylton NF, Miller MJ, Whelan T, Pierce LJ, Esserman LJ, Newman LA, Smith BL, Bear HD, Mamounas EP. Statement of the science concerning locoregional treatments after preoperative chemotherapy for breast cancer: a National Cancer Institute conference. *J Clin Oncol*.26: 791–797, 2008.
13. Kuerer HM, Newman LA, Buzdar AU, Dhingra K, Hunt KK, Buchholz TA, Binkley SM, Strom EA, Ames FC, Ross MI, Feig BW, McNeese MD, Hortobagyi GN, Singletary SE. Pathologic tumor response in the breast following neoadjuvant chemotherapy predicts axillary lymph node status. *Cancer J Sci Am*. 4: 230–236, 1998.
14. Kuerer HM, Newman LA, Smith TL, Ames FC, Hunt KK, Dhingra K, Theriault RL, Singh G, Binkley SM, Sneige N, Buchholz TA, Ross MI, McNeese MD, Buzdar AU, Hortobagyi GN, Singletary SE. Clinical course of breast cancer patients with complete pathologic primary tumor and axillary lymph node response to doxorubicin-based neoadjuvant chemotherapy. *J Clin Oncol*.17: 460–469, 1999.
15. Kuerer HM, Sahin AA, Hunt KK, Newman LA, Breslin TM, Ames FC, Ross MI, Buzdar AU, Hortobagyi GN, Singletary SE. Incidence and impact of documented eradication of breast cancer axillary lymph node metastases before surgery in patients treated with neoadjuvant chemotherapy. *Ann Surg*. 230: 72–78, 1999.
16. Symmans WF, Peintinger F, Hatzis C, Rajan R, Kuerer H, Valero V, Assad L, Poniecka A, Hennessy B, Green M, Buzdar AU, Singletary SE, Hortobagyi GN, Pusztai L. Measurement of residual breast cancer burden to predict survival after neoadjuvant chemotherapy. *J Clin Oncol*.25: 4414–4422, 2007.
17. Carey LA, Dees EC, Sawyer L, Gatti L, Moore DT, Collichio F, Ollila DW, Sartor CI, Graham ML, Perou CM. The triple negative paradox: primary tumor chemosensitivity of breast cancer subtypes. *Clin Cancer Res*. 13: 2329–2334, 2007.

CONFIDENTIAL

18. Buzdar AU, Ibrahim NK, Francis D, Booser DJ, Thomas ES, Theriault RL, Pusztai L, Green MC, Arun BK, Giordano SH, Cristofanilli M, Frye DK, Smith TL, Hunt KK, Singletary SE, Sahin AA, Ewer MS, Buchholz TA, Berry D, Hortobagyi GN. Significantly higher pathologic complete remission rate after neoadjuvant therapy with trastuzumab, paclitaxel, and epirubicin chemotherapy: results of a randomized trial in human epidermal growth factor receptor 2-positive operable breast cancer. *J Clin Oncol*.23: 3676–3685, 2005.
19. Sledge GW, Neuberg D, Bernardo P, Ingle JN, Martino S, Rowinsky EK, Wood WC. Phase III trial of doxorubicin, paclitaxel, and the combination of doxorubicin and paclitaxel as front-line chemotherapy for metastatic breast cancer: an intergroup trial (E1193). *J Clin Oncol*.21: 588–592, 2003.
20. Davidson N, O'Neill A, Vukov A, Osborne CK, Martino S, White D, Abeloff MD. Effect of chemohormonal therapy in pre-menopausal, node (+), receptor (+) breast cancer: An Eastern Cooperative Oncology Group Phase III Intergroup Trial (E INT-0101). *ASCO Annual Meeting*.abstr 249, 1999.
21. Shi P, Wang MM, Jiang LY, Liu HT, Sun JZ. Paclitaxel-doxorubicin sequence is more effective in breast cancer cells with heat shock protein 27 overexpression. *Chin Med J (Engl)*. 121: 1975–1979, 2008.
22. Esserman L, DeMichele A, Carey A, Hylton N, Leung E, Madhavan S, Broadwater G, I-SPY Clinical R, Pathology, Radiology Investigators at ACRIN and CALGB, NCI SPORE, and NCICB. Preliminary Results from I-SPY (CALGB 150007/150012/ACRIN 6657): Contrast-Enhanced MRI Correlative Science Studies to Characterize Tumor Response to Neoadjuvant Chemotherapy in Locally Advanced Breast Cancer. *NCI 15th SPORE Investigators' Workshop*. Abstr 086, 2007.
23. Esserman L, Perou C, Cheang M, DeMichele A, Carey L, Van't Veer LJ, Gray J, Petricoin E, Conway K, Berry D, I-SPY Investigators. Breast cancer molecular profiles and tumor response of neoadjuvant doxorubicin and paclitaxel: the I-SPY TRIAL (CALGB 150007/150012, ACRIN 6657). *ASCO Annual Meeting*.abstr LBA515, 2009.
24. Hylton N, Carey L, DeMichele A, Blume J, Broadwater G, Madhavan S, Rosen M, George S, Esserman L. Characterizing the biology and response of locally advanced breast cancer in women undergoing neoadjuvant therapy: preliminary results from the I-SPY trial. *San Antonio Breast Cancer Symp*.abstr 80, 2007.
25. Hylton N, Blume J, Bernreuter W, Pisano E, Rosen M, Morris E. MRI assessment of breast cancer response to neoadjuvant chemotherapy: preliminary findings of the American College of Radiology Imaging Network (ACRIN) Trial 6657. *RSNA 2008 (Radiological Society of North America)*.abstr SSA0101, 2008.
26. Hylton NM, Blume JD, Bernreuter WK, Pisano ED, Rosen MA, Morris EA, Weatherall PT, Lehman CD, Polin SM, Newstead G, Marques HS, Schnall MD, Esserman LJ. Comparison of MRI endpoints for assessing breast cancer response to neoadjuvant treatment: preliminary findings of the American College of Radiology Imaging Network (ACRIN) trial 6657. *San Antonio Breast Cancer Symp*.abstr 6043, 2008.
27. Hylton N, Blume J, Gatsonis C, Gomez R, Bernreuter W, Pisano E, Rosen M, Marques H, Esserman LS, Mitch, Team AI-ST. MRI tumor volume for predicting response to neoadjuvant chemotherapy in locally advanced breast cancer: Findings from ACRIN 6657/CALGB 150007. *ASCO Annual Meeting*.abstr 529, 2009.
28. Albertson D, Chin K, Devries S, Feiler H, Pinkel D, Spellman P, Waldman F, Wang N, Hennessy B, Mills G, Barcellos Hoff MH, Bissell M, Guan Y, Hu Z, Kuo W-L, McCormick F, Neve R, Stampfer M, Wooster R, Yaswen P, Das D, Fridlyand J, Correll E, Jin J, Nordmeyer B, Sudar D, Chew K, Dairkee S, Ljung BM, Hwang S, Esserman L, Arbushites M, Benz C, Koehler M, Marks JD, Zhou Y, Park J, Weber B, Gray J. Genomic approaches to breast cancer subset identification and treatment. *San Antonio Breast Cancer Symp*.abstr 31, 2007.
29. Gomez R, Hylton N, Madhavan S, Leung E, Broadwater G, Herman B, Esserman L. Preliminary results from I-SPY trial: tumor patterns on pre-treatment MRI predict breast conservation therapy eligibility. *San Antonio Breast Cancer Symp*.abstr 2024, 2007.
30. Gomez RE, Zakhireh J, Moore D, DeMichele A, Przewoznik J, Ollila D, Frank J, Krontiras H, Montgomery L, Sarode V, Broadwater G, Esserman L. Sentinel node biopsy performed in the neoadjuvant setting for breast cancer: results from the I-SPY TRIAL (CALGB 150007/150012 & ACRIN 6657). *San Antonio Breast Cancer Symp*.abstr 202, 2008.
31. Buxton MB, Esserman LJ. The challenge of integrating information and improving care for breast cancer: the I-SPY trial informatics effort. *42nd ASCO Annual Meeting, Educational Book,* 2005.
32. Zhu J, Sanborn JZ, Wang T, Hsu F, Benz S, Szeto C, Esserman L, Haussler D. UCSC cancer genomics browser. *San Antonio Breast Cancer Symp*.abstr 2022, 2008.

CONFIDENTIAL

33. Grove AS. Efficiency in the health care industries: a view from the outside. *JAMA*.294: 490-492, 2005.
34. Berry DA. Bayesian clinical trials.*Nat Rev Drug Discov*.5: 27–36, 2006.
35. Esserman L, Hylton N, Yassa L, Barclay J, Frankel S, Sickles E. Utility of magnetic resonance imaging in the management of breast cancer: evidence for improved preoperative staging. *J Clin Oncol*.17: 110–119, 1999.
36. Gianni L, Zambetti M, Clark K, Baker J, Cronin M, Wu J, Mariani G, Rodriguez J, Carcangiu M, Watson D, Valagussa P, Rouzier R, Symmans WF, Ross JS, Hortobagyi GN, Pusztai L, Shak S. Gene expression profiles in paraffin-embedded core biopsy tissue predict response. *J Clin Oncol*.23: 7265–7277, 2005.
37. Pusztai L, Ayers M, Stec J, Clark E, Hess K, Stivers D, Damokosh A, Sneige N, Buchholz TA, Esteva FJ, Arun B, Cristofanilli M, Booser D, Rosales M, Valero V, Adams C, Hortobagyi GN, Symmans WF. Gene expression profiles obtained from fine-needle aspirations of breast cancer reliably identify routine prognostic markers and reveal large-scale molecular differences between estrogen-negative and estrogen-positive tumors. *Clin Cancer Res* 9: 2406–2415, 2003.
38. Rouzier R, Rajan R, Wagner P, Hess KR, Gold DL, Stec J, Ayers M, Ross JS, Zhang P, Buchholz TA, Kuerer H, Green M, Arun B, Hortobagyi GN, Symmans WF, Pusztai L. Microtubule-associated protein tau: a marker of paclitaxel sensitivity in breast. *Proc Natl Acad Sci U S A*. 102: 8315–8320, 2005.
39. Tordai A, Wang J, Andre F, Liedtke C, Yan K, Sotiriou C, Hortobagyi GN, Symmans WF, Pusztai L. Evaluation of biological pathways involved in chemotherapy response in breast. *Breast Cancer Res*. 10: 29, 2008.
40. Mirzoeva OK, Das D, Heiser LM, Bhattacharya S, Siwak D, Gendelman R, Bayani N, Wang NJ, Neve RM, Guan Y, Hu Z, Knight Z, Feiler HS, Gascard P, Parvin B, Spellman PT, Shokat KM, Wyrobek AJ, Bissell MJ, McCormick F, Kuo WL, Mills GB, Gray JW, Korn WM. Basal subtype and MAPK/ERK kinase (MEK)-phosphoinositide 3-kinase feedback signaling determine susceptibility of breast cancer cells to MEK inhibition. *Cancer Res*. 69: 565–572, 2009.
41. Wulfkuhle JD, Speer R, Pierobon M, Laird J, Espina V, Deng J, Mammano E, Yang SX, Swain SM, Nitti D, Esserman LJ, Belluco C, Liotta LA, Petricoin EFr. Multiplexed cell signaling analysis of human breast cancer applications for personalized therapy. *J Proteome Res*. 7: 1508-1517, Epub Feb 1508 2008 .
42. Peintinger F, Anderson K, Mazouni C, Kuerer HM, Hatzis C, Lin F, Hortobagyi GN, Symmans WF, Pusztai L. Thirty-gene pharmacogenomic test correlates with residual cancer burden after preoperative chemotherapy for breast cancer. *Clin Cancer Res*.13: 4078–4082, 2007.
43. DeMichele A, Gimotty P, Botbyl J, Aplenc R, Colligon T, Foulkes AS, Rebbeck TR. In response to "Drug metabolizing enzyme polymorphisms predict clinical outcome in a node-positive breast cancer cohort". *J Clin Oncol*.25: 5675–5677, 2007.
44. De Michele A, Dunn L, Gimotty P, Livasy C, Perou C, Carey L, Esserman L, Investigators I-S. Loss of p27 expression and response to neoadjuvant chemotherapy: Results from the I-SPY Trial. *ASCO Annual Meeting*. 2009.
45. Partridge SC, Gibbs JE, Lu Y, Esserman LJ, Tripathy D, Wolverton DS, Rugo HS, Hwang ES, Ewing CA, Hylton NM. MRI measurements of breast tumor volume predict response to neoadjuvant chemotherapy and recurrence-free survival. *AJR Am J Roentgenol*. 184: 1774–1781, 2005.
46. Partridge SC, Heumann EJ, Hylton NM. Semi-automated analysis for MRI of breast tumors. *Stud Health Technol Inform*. 62: 259–260, 1999.
47. Hylton NM. Vascularity assessment of breast lesions with gadolinium-enhanced MR imaging. *Magn Reson Imaging Clin N Am*. 7: 411–420, x, 1999.
48. Hudis CA, Barlow WE, Costantino JP, Gray RJ, Pritchard KI, Chapman JA, Sparano JA, Hunsberger S, Enos RA, Gelber RD, Zujewski JA. Proposal for standardized definitions for efficacy end points in adjuvant breast cancer trials: the STEEP system. *J Clin Oncol*.25: 2127–2132, 2007.
49. Berry DA. The difficult and ubiquitous problems of multiplicities. *Pharm Stat*. 6: 155–160, 2007.

CONFIDENTIAL

<u>I-SPY 2 TRIAL</u>
*(**I**nvestigation of **S**erial Studies to **P**redict **Y**our **T**herapeutic **R**esponse with **I**maging **A**nd mo**L**ecular Analysis **2**)*

<u>SCREENING INFORMED CONSENT</u>

(Fill in names) and associates at the (fill in University) are conducting a research study to better understand how different women respond to standard chemotherapy and standard chemotherapy combined with investigational drugs to treat breast cancer. An investigational drug is a new drug that has not been approved by the Food and Drug Administration (FDA). The investigational drugs in this study have been previously tested in people.

A clinical trial is a research study that carefully tests new ways to prevent, diagnose, or treat diseases like breast cancer. Clinical trials include only participants who choose to take part in them. Please take your time to make your decision. Discuss it with your friends and family. The National Cancer Institute (NCI) booklet, "Taking Part in Cancer Treatment Research Studies", is available from your doctor. You may also want to discuss the pros and cons of participating in this trial with someone who has faced a similar decision; your doctor can probably recommend someone. You should only agree to participate in this study when you are comfortable enough with the information so that you can make an informed decision about joining.

Why is this study being done?

You are being asked to consider taking part in this study because you and your doctor are considering treating your newly diagnosed breast cancer with chemotherapy before your surgery (also known as neoadjuvant therapy).

This consent form is for the "screening" phase of the research study. The screening phase will determine if you are eligible to participate in the treatment phase of the study. The treatment phase of the study will compare how women respond to standard chemotherapy and standard chemotherapy combined with investigational drugs. If you participate in the screening phase of the study, you are not agreeing to participate in the treatment phase of the study. However, by agreeing to participate in this screening phase, you are agreeing to consider participating in the treatment phase if you are determined to be eligible for the study. If you are eligible for the treatment phase, the study team will further discuss the treatment study with you, answer your questions about the study, and obtain a separate written informed consent.

During this screening phase, you will also be asked to complete a series of questionnaires to better understand how the diagnosis of cancer and planning for treatment impact your quality of life.

This study is sponsored by QuantumLeap Healthcare Collaborative (QLHC).

CONFIDENTIAL

How many people will take part in the study?

We expect up to 250 women will be screened for each investigational treatment arm including the standard of care comparison group in this study.

What will happen if I take part in this research study?

You will need to have the following exams, tests, or procedures to find out if you can be in the treatment phase of the study. Some of these exams, tests, or procedures are part of regular cancer care and may be done even if you do not join the study. If you have had some of them recently, they may not have to be repeated. This will be up to your study doctor.

The following tests and procedures are done as standard of care and would be done even if you do not join this study:

- Your medical history will be reviewed with the study doctor or nurse.
- You will have a physical examination, including the measurement of your height, weight, and performance status (activities you do every day).
- You will be given a pregnancy test if you are a woman who can become pregnant.
- You will have blood tests done to check how your body is functioning.
- You will have an echocardiogram (ECHO) or multiple-gated acquisition (MUGA) scan to test how well your heart is functioning.
    - The ECHO scan uses sound waves to make pictures of your heart, which helps determine how well your heart pumps blood. You will be asked to lie on your left side while a technician places a probe with gel on your chest to create images of your heart to determine the function and size. The entire procedure will take about an hour.
    - The MUGA scan is performed by taking a small sample of blood that is then "labeled" with a radioactive substance. This procedure requires the placement of an IV needle into a vein in your arm or port if you have one. The labeled blood sample is then re-injected into a vein in your arm and allowed to circulate in your blood for approximately 60 minutes. Once the labeled blood has circulated around your body, a series of x-ray pictures (similar to a movie) are taken of your heart as it beats. You will be asked to lie flat on a table and remain still for approximately 10-20 minutes while the pictures of your heart are being taken. The entire procedure will take about an hour and a half.
- You may have a Computed Tomography (CT) scan with or without Positron Emission Tomography (PET). These scans are a computer-enhanced X-ray to find out if you might have cancer in another part of your body.
    - You will need to lie still on a table on your chest and stomach inside a large doughnut-shaped machine. The table will move and the machine will make clicking and whirring noises as the pictures are taken. An iodine dye (contrast material) will first be injected into a vein or given to you by mouth. The dye makes tissue and organs more visible in the pictures. The entire procedure will take about an hour.
- You will have magnetic resonance imaging (MRI) of your breasts.
    - You will be placed on your stomach on a padded table in the center of the MRI machine, which is a large cylindrical magnet that is open at each end. The MRI machine produces a strong magnetic field that passes through your body and a computer attached to the MRI machine will process these signals into a picture.

CONFIDENTIAL

You will hear a loud banging noise produced from the magnet during the scan. At some point during the examination, an MRI contrast agent (a liquid dye called gadolinium) will be injected into a vein in your arm through a small catheter. The contrast agent improves the images of your breast by highlighting certain tissue. The MRI scan will take about an hour and you will need to lie still during that time.

- You will have a core biopsy of your breast cancer.
  - A core biopsy is a procedure where your doctor will insert a needle into your breast to extract a piece of tissue about the size of pencil lead. Your doctor will anesthetize (numb) the area prior to inserting the needle to minimize discomfort. You may have bruising and some minor discomfort after this procedure.

The following tests and procedures will be done as part of this study:

- You will have an electrocardiogram (ECG or EKG) that measures that electrical activity of your heart. An ECG involves attaching small wires to your arms, legs, and chest to read the electrical impulses of your heart. An ECG helps identify heart rhythm abnormalities and imbalances of salts in the body. This procedure is done in the cardiology department and takes approximately 15 to 30 minutes.

- You will have magnetic resonance imaging (MRI) of your breasts. If you have already had an MRI of your breasts, you may have to have a second MRI if you join this study.
  - Your MRI will include diffusion-weighted (DW) MRI images to show the different movement of water in tumor and non-tumor tissue. The images collected will be used to learn about the biology of your breast cancer and how it responds to treatment..
- You will have a core biopsy of your breast cancer. If you have already had a core biopsy, you will have to have a second core biopsy if you join this study. The tissue taken from the core biopsy will be used for the following tests:
  - Tests to determine your eligibility and/or treatment, and to have more information about your tumor:
  - MammaPrint: a test to find out if your cancer is at high risk for recurrence if you did not have any treatment, other than surgery, for your cancer.
  - TargetPrint HER2: Additional test to determine your HER2 status (Human Epidermal growth factor Receptor 2) of your breast tumor.
  - BluePrint: A test to measures if your tumor is a particular subtype-Luminal-type (A or B), Basal-type, or HER2-type.
  - TargetPrint ER/PR: Test to measure the ER (Estrogen Receptor) and PR(Progesterone Receptor) status of your breast tumor.

    Your results from the MammaPrint, TargetPrint and BluePrint tests will be made available to your study doctor to share with you.

  - Additional tests will be done to learn more about your breast cancer. These are primarily research tests and their meaning is not yet fully understood. As we learn more about the meaning of these tests, we may approach you to ask if you want to receive the results. After the research has been explained, you would decide whether to receive the results or to decline them.

CONFIDENTIAL

- You will have an additional blood sample taken (about 2-3 teaspoons) before starting the treatment phase for research purposes. The blood sample may be collected at the same time the catheter is placed in your vein for the MRI scan. The blood sample will be used to learn more about your breast cancer.
- Your blood and tissue may be used for genetic research (about diseases that are passed on in families). Reports about research done with your blood and tissue will not be given to you or your doctor, or put into your health record without your prior consent and approval.
- You may have additional tests done if you join this study to determine your eligibility.
- You will complete a questionnaire asking about symptoms like pain or nausea, fatigue, anxiety, sexuality, and other issues that can be affected because of breast cancer and its treatment. The questionnaires will take about 20 minutes to complete.

Your eligibility to continue in the treatment phase of this study will be based upon your ER (estrogen receptor) and HER2 status as well as your MammaPrint risk. **Once your results are back (within approximately two weeks) you will meet with your study doctor to review your results.**

If your test results show your cancer to be HER2+, or ER- or HER2+/ER+/MammaPrint high risk, you will be eligible to continue onto the treatment phase of the study.
If your test results show your cancer to be ER+, HER2– and MammaPrint low risk you will eligible for the I-SPY 2 Registry Study described in more detail below, under "Optional Research Studies".

If you are eligible for the treatment phase, you will be randomized for assignment to a particular treatment.

Being **randomized** means that you will be put into a treatment group by chance. There may be up to 9 treatment groups that you could be randomized to. Which treatment group you are randomized to is done by a computer taking your tumor type into account. Neither you nor the research staff will choose what treatment group you will be in. **If you are HER2-, you will have an 80% chance of receiving standard chemotherapy plus one of the investigational drugs and a 20% chance of receiving standard chemotherapy alone. If you are HER2+, you will have a 100% chance of receiving standard chemotherapy plus an investigational drug.**

If you are HER2-, standard chemotherapy in this trial is paclitaxel followed by doxorubicin and cyclophosphamide, also known as AC.

If you are HER2+, standard chemotherapy in this trial is paclitaxel (Taxol), + trastuzumab (Herceptin) followed by doxorubicin and cyclophosphamide, also known as AC.

The study plan below shows an overview of the Screening and Treatment Phases of the study.

CONFIDENTIAL

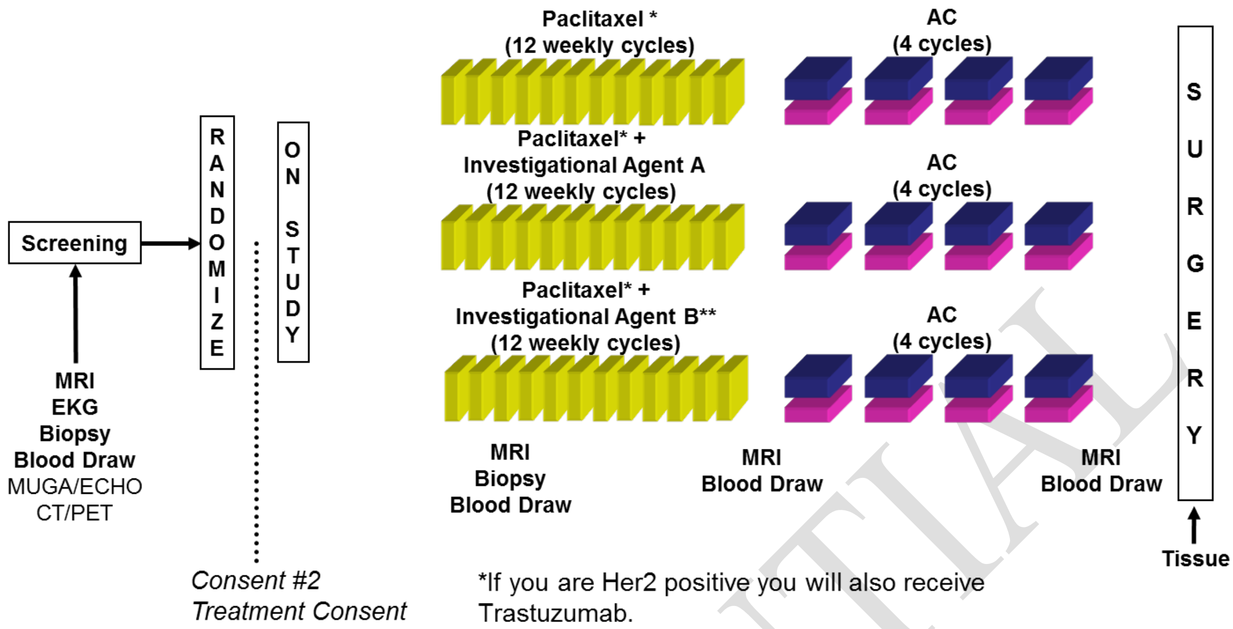

*If you are Her2 positive you will also receive Trastuzumab.

**An investigational drug may be used instead of Trastuzumab or the combination of Paclitaxel and Trastuzumab if you are Her2 Positive.

In bold are additional procedures (MRI, biopsy, blood draw) that are required as part of this study and may not be part of your normal cancer care. AC is also known as Doxorubicin and Cyclophosphamide.

How long will I be in the study?

It will take approximately 2 to 4 weeks to get your test results back and to know if you are eligible for the treatment phase of the study. If you are eligible and decide to join the treatment phase of this study, your treatment will last approximately 6 months.

Can I stop being in the study?

Yes. You can decide to stop at any time. Please talk with your study doctor about which information and/or study tissue or blood, if any, can be used by the study after you stop your participation.

What side effects or risks can I expect from being in the screening study?

You should talk to your study doctor about any side effects you experience while taking part in the study. Since many of the tests and procedures would be done even if you did not join the study, the following are side effects or risks that may occur even if you did not join the study.

Reproductive risks:

- You should not be or become pregnant while on this study. You should not breastfeed a baby while on this study. If you are able to have children, you will have a pregnancy test before you can be in the treatment phase of the study.

CONFIDENTIAL

- If you are eligible and decide to join the treatment phase of this study, you must agree to use an effective double barrier method of birth control to prevent pregnancy while receiving treatment.

Blood Draw () risks:

- The risks of drawing blood include temporary discomfort from the needle stick and bruising.

PET/CT scan risks:

- The scan uses a small amount of radiation as part of the X-ray. The amount of radiation you will be exposed to is relatively small. The dose of radiation may be potentially harmful, but the risks are so small that they are difficult to measure. If you have already had many X-rays, you should discuss this with your study doctor.
- The scan also uses an iodine dye (contrast material) and there is a slight risk of an allergic reaction. This can be mild (itching, rash) to severe (difficulty breathing, shock, or rarely death). The iodine dye may also cause kidney problems, especially if you are dehydrated or have poor kidney function. The study doctors will ask you about any allergies or related conditions before the procedure. If you have any of these problems, you may not be allowed to have a scan with contrast.
- Having a scan may mean some added discomfort for you. In particular, you may be bothered by feelings of claustrophobia when inside the scanner. The iodine dye may cause some discomfort when it is injected. You may feel warm and flushed and get a metallic taste in your mouth. Rarely, the iodine dye may cause nausea, vomiting, or headache.

Electrocardiogram associated risks:

- During an EKG, sticky patches are placed on your chest, arms, and legs. These patches connect to wires that go to a machine that measures the electrical activity of your heart. You may develop a slight rash, or experience redness or skin irrigation in the location where these patches were placed.

Magnetic Resonance Imaging (MRI) associated risks:

- Because the MRI machine acts like a large magnet, you will be screened before you can enter the MRI room. If you have a piece of metal in your body, such as a fragment in your eye, aneurysms clips, ear implants, spinal nerve stimulators, or a pacemaker, you will not be allowed into the MRI room and cannot have an MRI. No serious biological effects have been reported from the magnetic fields used in clinical MRI.
- Having an MRI may mean some added discomfort for you. In particular, you may be bothered by feelings of claustrophobia and by the loud tapping noise during the scan. If you experience a sensation of claustrophobia while in the magnet, the MRI will be immediately stopped. Temporary hearing loss has been reported from this loud noise. This is why you will be asked to wear earplugs.
- Gadolinium contrast:
  - Less likely side effects of the gadolinium injection are mild headaches, nausea, and local pain. A less likely but serious side effect for participants is an allergic reaction to gadolinium. These effects are most commonly hives and itchy eyes, but more severe reactions have been seen which result in shortness of breath or

CONFIDENTIAL

very rarely death. Rarely (about 2% of the time) low blood pressure and feeling lightheaded occur. This can be treated with intravenous fluids. Prior to study entry, you will have a routine blood test in order to check the function of your kidneys. Based on your medical history and results of the test, a doctor will decide whether it is safe for you to undergo the MRI scan.

Core Biopsy risks:

- Your breast will be numbed using a local anesthetic before taking the tissue sample from your tumor. A likely side effect is that you may experience minor discomfort from the procedure. There is a less likely risk of minor pain, bleeding and bruising. Serious bleeding, bruising, infection, or collection of air or gas in the chest cavity (pneumothorax) is possible, although very rare.

Unknown risks

- There may be more side effects that no one knows about yet. The study doctors will let you know if they learn anything that might make you change your mind about participating in the study.

Described below are side effects or risks that may occur from the extra study related procedures while you are in the screening phase of this study.

Screening risk:

- There is a 5% chance that the eligibility tests done from the tissue sample collected will not produce a definite result. If this happens, your eligibility for the treatment phase cannot be completed and you will not be able to join the treatment phase of the study.

Randomization risks:

- You will be assigned to a treatment group by chance. The treatment you receive may prove to be less effective or to have more side effects than the other study treatment(s) or other available treatments.

Treatment risks:

- If you have been found eligible for the treatment phase of this study, the risks from the treatment you have been randomized to will be discussed in detail in the treatment consent form. If you agree to the treatment you have been randomized to, you will sign the treatment consent form and agree to continue in the treatment phase of this study.

Are there benefits to taking part in the study?

Taking part in this study may be better, worse or make no difference in your health. Extra tests will be done as part of this study that may or may not help you and your doctor know more about your specific cancer.

What other choices do I have if I do not take part in this study?

You may choose not to participate in this study. Your option to participate in this study will not affect the care you receive here for your breast cancer in any way.

CONFIDENTIAL

Please talk to your doctor about your choices before deciding if you will take part in this study.

Will my medical information be kept private?

We will do our best to make sure that the personal information in your medical record is kept private. An electronic copy of portions of your medical record will be used in this study through an encrypted secure server. All of your personal information will be removed from these electronic records and you will only be identified by a study number. However, we cannot guarantee total privacy. Your personal information may be given out if required by law. If information from this study is published or presented at a scientific meeting, your name and other personal information will not be used.

Organizations that may look at copies of sections of your medical records used in this trial for research quality assurance and data analysis include:

- QuantumLeap Healthcare Collaborative (QLHC) as the sponsor of this study or their authorized designee
- The National Cancer Institute (NCI) for regulatory oversight of this study
- The Food and Drug Administration (FDA) for regulatory oversight of this study
- The American College of Radiology Imaging Network (ACRIN) for MRI data analysis
- Local Institutional Review Boards (IRB) and government agencies, such as the office for Human Research Protections (OHRP), that are involved in keeping research safe for people.

Health Insurance companies/group health plans may not request your genetic information that we get from your participation in this study. It is against federal law, Genetic Information Nondiscrimination Act (GINA), for a health insurance company/group health plans and most employers to discriminate against you based on your genetic information. By taking part in this study, your genetic information will not be shared according to the GINA federal law.

What are the costs of taking part in the study?

Taking part in the screening phase of this study may lead to added costs to you or your insurance carrier. Please ask about any expected additional costs or insurance problems.

You and/or your health plan/insurance company will need to pay for some or all of the costs of treating your cancer in this study (this includes both the screening and treatment phase, if you are found eligible). Some health plans will not pay for these costs if you are taking part in a research study. Check with your health plan/insurance company to find out what they will pay for. Taking part in this study may or may not cost you or your insurance company more than the cost of getting regular cancer treatment.

The study will cover the cost of the following study related tests and procedures done during the screening phase:

- The MRI scan, if the scan was done only for the purpose of this study.
- The core biopsy, if the core biopsy was done only for the purpose of this study.
- The blood collected for research for this study.
- Any additional blood tests that are done only for the purpose of this study.

CONFIDENTIAL

For more information on clinical trials and insurance coverage, you can visit the National Cancer Institute's Web site at http://www.cancer.gov/about-cancer/treatment/clinical-trials/paying/insurance. You can print a copy of the "Clinical Trials and Insurance Coverage" information from this Web site. Another way to get the information is to call 1-800-4-CANCER (1-800-422-6237) and ask them to send you a free copy.

Will I be paid for taking part in this study?

You will not receive payment for taking part in the study. However, if you need to make additional trips to the clinic for the tests, you may request travel reimbursement.

What happens if I am injured because I took part in this study?

It is important that you tell your study doctor (fill in Investigators name(s)) if you feel that you have been injured because of taking part in this study. You can contact Dr. (fill in) or their associates at xxx-xxx-xxxx.

If you are injured as a result of being in this study, treatment will be available. You and/or your health plan will be charged for this treatment. The study will not pay for medical treatment. The costs of such treatment may be covered by (insert University) and the study sponsor, depending on a number of factors. The University and the Study Sponsor do not normally provide any other form of compensation for injury. For further information about this, you may call the office of the (insert IRB) at xxx-xxx-xxxx.

What are my rights if I take part in this study?

Taking part in this study is your choice. You may choose either to take part or not to take part in the study. If you decide to take part in this study, you may leave the study at any time. No matter what decision you make, there will be no penalty to you and you will not lose any of your regular benefits. Leaving the study will not affect your medical care. You can still get your medical care from our institution.

We will tell you about new information or changes in the study that may affect your health or your willingness to continue in the study.

In the case of injury resulting from this study, you do not lose any of your legal rights to seek payment by signing this form.

Who can answer my questions about the study?

This study has been explained to you by Dr. (fill in) or the person who signed below. If you have any other questions about the study, you may call Dr. (fill in) at xxx-xxx-xxxx**.** Alternatively, you can call the study coordinator,(fill in), at xxx-xxx-xxxx.

CONFIDENTIAL

If you wish to ask questions about the study or your rights as a research participant to someone other than the study doctor or coordinator, or if you wish to voice any problems or concerns you may have about the study, please call (fill in your site) IRB at xxx-xxx-xxxx.

Where can I get more information?
You may call the NCI's Cancer Information Service at **1–800–4–CANCER (1–800–422–6237).** Visit the NCI's Web sites for comprehensive clinical trials information (http://www.cancer.gov/clinicaltrials) or for accurate cancer information (**http://www.cancer.gov)** including PDQ®, an NCI database that contains the latest information about cancer treatment, screening, prevention, genetics, supportive care, and complementary and alternative medicine, plus clinical trials.

For more general information about this trial, you can visit the study's website at: http://www.ispy2.org.

A description of this clinical trial will be available on http://www.ClinicalTrials.gov, as required by US law. This website will not include information that can identify you. At most, the website will include a summary of the study results. You can search this website at any time. The I-SPY 2 TRIAL Identifier is NCT01042379. Enter this identifier into the search engine on ClinicalTrials.gov for information on the I-SPY 2 TRIAL.

Collection of Samples and Use of Research Material
What about the use of my tissue and blood for research?
The researchers think that they will use all of the blood and tissue samples for research as part of this study. However, if any of your tissue or blood is leftover, the researchers would like to keep your extra samples, for up to 10 years, and use them for additional research outside of this study. We would like your permission to use any leftover tissue and blood for future research within or outside of this study.

Your tissue and blood will be used only for research and will not be sold. You will not be paid for allowing your tissue to be used in research even though the research done with your tissue may help to develop new products in the future. You will not receive any payment or financial benefit from any products, tests, or discoveries derived from these samples.

What are the benefits of using your tissue and blood for research?
The research that will be done with your tissue and blood may not help you. It might help people who have cancer and other diseases in the future. The benefits of research using tissue and blood include learning more about what causes cancer and other diseases, how to prevent them, how to treat them, and how to cure them.

What are the risks of using your tissue and blood for research?
Your tissue and blood samples will not be identified using your personal information. Your personal information will not be shared with the researchers. However, the researchers may have access to information about your health. There is a very small chance that your personal information may be released. We will do our best to make sure that your personal information is

CONFIDENTIAL

kept private.

You can still be a part of the study even if you do not want your tissue and blood used for future research within or outside of this study. If you agree to have your tissue and blood used for future research, you can change your mind at any time. Let your study doctor know if you change your mind and your samples will no longer be used for future research outside of this study.

I agree to have my tissue and blood be used for future research.

Yes_________ No_________ Participant Initials_________

For research results that are applicable to my family members, please contact the individual below if I am unavailable.

Name____________________________________ Relationship_____________________
Phone Number_____________________________
Email address______________________________

Optional Research Studies:

"I-SPY-2 Registry Study"(for patients with ER+/HER2-/MammaPrint Low Risk Cancer)

If your tumor is found is found to be ER+, HER2- and MammaPrint Low Risk, study doctors would like to continue to track your treatment and your clinical outcomes. This is NOT a treatment study. Your treatment plan will be decided by you and your doctor.

What will happen if I take part in this study?

The study will collect information about the treatment and how your treatment responds. You will also be asked to continue to complete the questionnaires regarding your quality of life. These will be mailed to you at no cost.

Additional MRI: If you and your doctor choose to do chemotherapy or hormone therapy before surgery, you may get a MRI of your breast prior to surgery to monitor how your breast cancer responds to treatment. You should discuss with your physician whether an MRI will be performed prior to surgery. If you have an MRI scan for study purposes only, the costs will be covered by the study.

CONFIDENTIAL

Tissue Sample at the time of diagnosis and surgery: There are no additional biopsies required for this study. However, a sample of your tissue from your original tumor biopsy, or from your surgery may be used to learn about the biology of your breast cancer.

How long will I be followed for this portion of the study?
You will be followed for the duration of your treatment and up to 15 years after.

If your tumor is found to be ER+, HER2- and MammaPrint Low Risk, do you wish to be followed for the I-SPY 2 Registry Study?

Yes____________________No______________Participant Initials_______________________

Consent
In addition, you will be asked to sign a separate form to authorize access to your health information. You will be given copies of this consent form and the HIPAA form to keep.

PARTICIPATION IN RESEARCH IS VOLUNTARY. You have the right to decline to participate or withdraw at any point in this study without jeopardy to your medical care. Leaving the study will not result in any penalty or loss of benefits to which you are entitled. The study doctor may decide to take you off this study with or without your consent if it is in your best medical interest, funding is stopped, your condition worsens, or new information becomes available.

SIGNATURE
I have read all the above, asked questions, and received answers concerning areas I did not understand.

If you wish to participate in the screening portion of this study, you should sign below.
By signing below you are also acknowledging your decision to either opt in or opt out of the Optional Research Studies described above.

Participant Name: ____

Participant Signature: ____ Date: _____________

Signature of Doctor: ____ Date: _____________

Signature of Person Obtaining Consent: ________________________ Date: _____________

If Necessary
Translator Name: ____

CONFIDENTIAL

Signature of Translator: ___________________________________ Date: ____________

CONFIDENTIAL

I-SPY 2 TRIAL

*(**I**nvestigation of **S**erial Studies to **P**redict **Y**our **T**herapeutic **R**esponse with **I**maging **A**nd mo**L**ecular Analysis **2**)*

STANDARD CHEMOTHERAPY TREATMENT INFORMED CONSENT

(Fill in names) and associates at the (fill in University) are conducting a research study to better understand how different women respond to standard chemotherapy and standard chemotherapy combined with investigational drugs to treat breast cancer. An investigational drug is a new drug that has not been approved by the Food and Drug Administration (FDA). The investigational drugs in this study have been previously tested in people.

A clinical trial is a research study that carefully tests new ways to prevent, diagnose, or treat diseases like breast cancer. Clinical trials include only participants who choose to take part in them. Please take your time to make your decision. Discuss it with your friends and family. The National Cancer Institute (NCI) booklet, "Taking Part in Cancer Treatment Research Studies", is available from your doctor. You may also want to discuss the pros and cons of participating in this trial with someone who has faced a similar decision; your doctor can probably recommend someone. You should only agree to participate in this study when you are comfortable enough with the information so that you can make an informed decision about joining.

You have been asked to take part in this study because you and your doctor have decided to treat your newly diagnosed breast cancer with chemotherapy before your surgery (also known as neoadjuvant therapy). You have also completed the screening phase of this study and have been identified as being eligible to continue on the treatment phase of this study.

Why is this study being done?

At the present time, almost all women with breast cancer receive standard chemotherapy because it is not known who benefits most from such treatment. We hope that this study will determine how women with breast cancer will respond to standard chemotherapy and standard chemotherapy with investigational drugs on the basis of the particular markers in your tumor tissue and blood.

The purpose of this study is to learn:

- If participants with breast cancer benefit from adding an investigational drug to their standard chemotherapy before surgery. An investigational drug is a new drug that has not been approved by the Food and Drug Administration (FDA). The investigational drugs in this study have been previously tested in people.
- What effects, good and bad, chemotherapy has on your cancer.
- What changes this combination of chemotherapy has on your tumor through magnetic resonance imaging (MRI is a procedure used to create detailed pictures of your breasts) and tissue and blood biomarkers. A biomarker is a substance sometimes found in tissue or blood that can be present in normal cells and cancer cells. Researchers will look at differences in biomarkers between normal and cancer cells.

CONFIDENTIAL

- What impact the various treatments you receive for breast cancer have on your quality of life before and up to two years after your surgery

This research study is a phase 2 study. A phase 2 study tests if an investigational drug, or combination of investigational drugs, works in a certain type of cancer and what side effects it has.

This study is also being done to determine whether MRI scans can be routinely used in women receiving neoadjuvant chemotherapy to predict (early in the course of treatment) which treatments will be most effective for women with breast cancer.

This study is sponsored by QuantumLeap Healthcare Collaborative (QLHC).

How many people will take part in the study?

Up to 120 women will take part in the treatment phase of the study for each treatment arm.

What will happen if I take part in the treatment phase of the study?

You will receive the following standard chemotherapy care for treating locally advanced breast cancer: An 8–12 week course of paclitaxel followed by an 8–12 week course of doxorubicin and cyclophosphamide. Depending on your randomization assignment, you may receive paclitaxel following surgery. **Paclitaxel** is a type of drug used to treat cancer by blocking cell growth, sometimes called T. **Doxorubicin** is a type of drug used to treat cancer by killing cancer cells and **cyclophosphamide** is a type of drug used to treat cancer by slowing or stopping cell growth. The combination is sometimes called AC. If you have HER2+ breast cancer, you may receive trastuzumab, also known as Herceptin. Trastuzumab is a type of drug used to treat HER2+ cancer.

Before each chemotherapy treatment, you will have a clinic visit with your study doctor and complete laboratory safety blood work.

You will receive the following standard chemotherapy drugs prior to surgery:

Drug Name	How treatment will be given	How long treatment will take each time	How often treatment is given	When treatment will be given
Paclitaxel*	By vein	1 hour	Weekly	Weeks 1–12
Doxorubicin	By vein	2 hours	Every 2 or 3 weeks	Weeks 13–25
Cyclophosphamide	By vein	1 hour	Every 2 or 3 weeks	Weeks 13–25

*For some investigational drug combinations, paclitaxel may be given after you have surgery either weekly for 12 cycles or every two weeks for four cycles.

A supplemental consent form will be added to the end of this one that will explain how trastuzumab and/or the investigational drugs will be given, the risks related to the investigational drugs that you have been randomized to receive, and any additional tests and procedures required to monitor your health.

CONFIDENTIAL

The following research tests and procedures will be done during the treatment phase of the study:

MRI (Magnetic Resonance Imaging) scans:

You will have three more MRI scans after you begin your chemotherapy treatment and before you have surgery. Your MRI will include diffusion-weighted (DW) MRI images to show the different movement of water in tumor and non-tumor tissue. These MRI scans will help monitor how your breast cancer responds to treatment and learn about the biology of your breast cancer.

- The first MRI scan will be done 3 weeks after you begin your paclitaxel treatment.
- The second MRI scan will be done after completing your paclitaxel treatment but before starting your AC treatment.
- The third MRI scan will be done after the completion of all of your chemotherapy but before you have surgery.

Core Biopsy and Tissue Sample:

You will have one additional core needle biopsies during your chemotherapy treatment.

- The core needle biopsy will be done three weeks after you begin your paclitaxel treatment.

If you have any cancer left at the time of surgery, a small amount of tissue will be taken.

The tissue collected during the course of this study will be used to learn about the biology of your breast cancer and how it responds to treatment.

Your tissue may be used for genetic research (about diseases that are passed on in families). Reports about research done with your tissue will not be given to you or your doctor. These reports will not be put in your health record. The research will not have an effect on your care.

Blood Samples:

You will have three additional blood samples (about 2–3 teaspoons) collected during your chemotherapy treatment. The blood collected during the course of this study will be used to learn about the biology of your breast cancer and how it responds to treatment.

- The first blood draw will be done three weeks after you begin your paclitaxel treatment.
- The second blood draw will be done after you complete your paclitaxel treatment but before starting your AC treatment.
- The third blood draw will be done after you complete all of your chemotherapy treatment but before you have surgery.

Your blood may be used for genetic research (about diseases that are passed on in families). We may approach you to receive the results of research using your tissue and blood samples Reports about research done with your blood or tissue will not be given to you or your doctor, or put in your health record without your prior consent and approval. The research will not have an effect on your care.

CONFIDENTIAL

Quality of Life Questionnaires:
You will complete a series of questionnaires asking about symptoms like pain and nausea, fatigue, anxiety, sexuality, and other issues that can be affected because of breast cancer and its treatment. The questionnaires will take from 5–20 minutes to complete based on which questions are included each time. You will be asked to complete the questionnaires when you are first assigned to a treatment on study, about mid-way through chemotherapy, before surgery, about one month after surgery, and then at 6, 12, and 24 months after your surgery. If you are uncomfortable with any specific questions, you may leave them blank.

A study plan showing when these MRI scans, biopsies, and blood samples will be done is shown below.

CONFIDENTIAL

Study Plan for Treatment

**Before Chemotherapy**<br><br>**(Screening Phase—Completed)**	**Week 1–3**	**End of Week 3**	**Week 4–12**	**End of Week 12**	**Week 13–25**	**Week 23–30**	**Week 24–32**	**Treatment after Surgery**
**MRI Scan**<br><br>**Core Biopsy**<br><br>**Blood Draw**<br><br>MUGA/ECHO<br><br>CT/PET Scan	Weekly paclitaxel chemotherapy* (if you have been assigned), laboratory safety blood work, clinic visit with study doctor<br><br>**Investigational drug (if you have been assigned)**<br><br>Weekly trastuzumab (if you have been assigned)	***MRI scan***<br><br>***Core biopsy***<br><br>***Blood draw***	Weekly paclitaxel chemotherapy* (if you have been assigned), laboratory safety blood work, clinic visit with study doctor<br><br>**Investigational drug (if you have been assigned)**<br><br>Weekly trastuzumab (if you have been assigned)	***MRI Scan***<br><br>***Blood Draw***<br><br>MUGA or ECHO (if you have been assigned to trastuzumab)	Every 2 or 3 weeks: AC chemotherapy, laboratory safety blood work, clinic visit with study doctor<br><br>*the safety blood work and clinic visits will be on the same day as the AC	***MRI Scan***<br><br>***Blood Draw***<br><br>MUGA or ECHO (if you have been assigned to trastuzumab)	Surgery<br><br>***Tissue sample taken at time of surgery***	Treatment after surgery is at the discretion of you and your doctor.

*For some investigational drug combinations, paclitaxel may be given after you have surgery either weekly for 12 cycles or every two weeks for four cycles.

Abbreviations: AC: doxorubicin/cyclophosphamide; CT: computed tomography; ECHO: echocardiogram; MUGA: multigated acquisition scan; PET: positron emission tomography.

Bold: additional procedures that you would have as part of this study which may not be part of your normal cancer care.

CONFIDENTIAL

How long will I be in the study?

You will be in the study during the entire time of your neoadjuvant chemotherapy treatment, approximately 6 months. We will check with your doctor to see how you are doing every 6 months for approximately 5 years after your neoadjuvant chemotherapy treatment. The study staff will monitor any serious side effects you may be experiencing for up to 1 year after your surgery.

Can I stop being in the study?

Yes. You can decide to stop at any time. Tell the study doctor if you are thinking about stopping or decide to stop. They will tell you how to stop your participation safely. Please, also talk with your doctor about which information and/or study tissue or blood, if any, can be used by the study after you stop your participation.

If you are thinking about stopping treatment, it is important to tell your study doctor so any risks from the study treatment can be checked by your study doctor. Another reason to tell your doctor that you are thinking about stopping is to discuss what follow-up care and testing could be most helpful for you.

The study doctor may stop your participation in this study at any time without your consent if:

- The doctor believes it is in the best interest for your health
- You do not follow the study rules
- Your health gets worse or you experience significant side effects from the drug
- The study is stopped by the sponsors

What side effects or risks can I expect from being in the study?

You may have side effects while on the study. Everyone taking part in the study will be watched carefully for any side effects. However, doctors and the study sponsor don't know all the side effects that may happen and there may be unknown side effects that could occur. Side effects may be mild or very serious. Your health team may give you medicines to help lessen side effects. Many side effects go away soon after you stop what is causing them. In some cases, side effects can be serious or long lasting, causing hospitalization, and/or may never go away. There is also a risk of death. You may experience unexpected side effects that may not allow you to complete your standard treatment. You should talk to your study doctor about any side effects you experience while taking part in the study.

CONFIDENTIAL

Potential Side Effects of Standard Chemotherapy Treatments

	Side Effects		
**Drug Treatment**	**Likely**<br>**(seen in more than 20% of participants)**	**Less Likely**<br>**(Seen in 20% or less of participants)**	**Rare but Serious**<br>**(Seen in 2–3% or less of participants but might be serious)**
**Paclitaxel** also known as Taxol (T)	• Temporary lowering of the number of white blood cells (neutropenia) which could result in fever and increased risk of infection<br>• Temporary lowering of red blood cells (anemia)<br>• Temporary lowering of blood platelet cells (thrombocytopenia)<br>• Diarrhea<br>• Loss of hair on scalp and body (alopecia)<br>• Temporary or mild numbness or tingling in the fingers or the toes which may continue after you have stopped treatment (neuropathy)<br>• Temporary pain in the muscles and joints	• Nausea<br>• Vomiting<br>• Flushing in the face<br>• Low blood pressure<br>• Mouth sores (like canker sores)<br>• Abnormal liver function as seen on a blood test	• Allergic reaction, symptoms include:<br>- rash<br>- shortness of breath<br>• Slow or irregular heart beat<br>• Low blood pressure<br>• High blood pressure<br>• Numbness or tingling in the fingers or toes that may cause difficulty in walking or buttoning clothes on a long term basis
**Doxorubicin + cyclophosphamide** also known as AC	• Temporary lowering of the number of white blood cells (neutropenia) which could result in fever and increased risk of infection<br>• Temporary lowering of red blood cells (anemia)<br>• Temporary lowering of blood platelet cells (thrombocytopenia)<br>• Nausea<br>• Vomiting<br>• Diarrhea<br>• Loss of appetite<br>• Skin and nail discoloration<br>• Hair loss (alopecia)<br>• Metallic taste<br>• Mouth sores and throat irritation<br>• Urine may turn red for 1-2 days (due to the color of the doxorubicin)<br>• Irritation of the bladder<br>• Fatigue<br>• Premature menopause (stopping of menstrual	• Sensitivity to sunlight<br>• Heart muscle weakness<br>• Watery, sore eyes<br>• Skin rash and itching<br>• Stuffy nose and sneezing<br>• Dizziness<br>• Confusion<br>• Agitation<br>• Yellowing of the skin and/or eyes<br>• Skin tissue damage if some of the drug leaks from the vein while it is being given to you	• Bleeding from bladder

CONFIDENTIAL

	periods, possibly on a long term basis)		

Epirubicin:

- Due to a potential national shortage of doxorubicin, we may need to substitute doxorubicin with a very similar drug called epirubicin. Epirubicin is in the same class of drugs as doxorubicin. Clinical studies of epirubicin-containing drug regimens in participants with early breast cancer have found these regimens to be equally beneficial as the doxorubicin-containing regimens.
- There is no increased risk in taking epirubicin.
- There are no additional side-effects for epirubicin besides those already listed above for doxorubicin.

Magnetic Resonance Imaging (MRI) associated Risks:

- Because the MRI machine acts like a large magnet, you will be screened before you can enter the MRI room. If you have a piece of metal in your body, such as a fragment in your eye, aneurysms clips, ear implants, spinal nerve stimulators, or a pacemaker, you will not be allowed into the MRI room and cannot have an MRI. No serious biological effects have been reported from the magnetic fields used in clinical MRI.
- Having an MRI may mean some added discomfort for you. In particular, you may be bothered by feelings of claustrophobia and by the loud tapping noise during the scan. If you experience a sensation of claustrophobia while in the magnet, the MRI will be immediately stopped. Temporary hearing loss has been reported from this loud noise. This is why you will be asked to wear earplugs.
- Gadolinium contrast:
  - Less likely side effects of the gadolinium injection are mild headaches, nausea, and local pain. A less likely but serious side effect for participants is an allergic reaction to gadolinium. These effects are most commonly hives and itchy eyes, but more severe reactions have been seen which result in shortness of breath or very rarely death. Rarely (about 2% of the time) low blood pressure and feeling lightheaded occur. This can be treated with intravenous fluids. Prior to study entry, you will have a routine blood test in order to check the function of your kidneys. Based on your medical history and results of the test, a doctor will decide whether it is safe for you to undergo the MRI scan.

Core Biopsy Risks:

- Your breast will be numbed using a local anesthetic before taking the tissue sample from your tumor. A likely side effect is that you may experience minor discomfort from the procedure. There is a less likely risk of minor pain, bleeding and bruising. Serious bleeding, bruising, infection, or collection of air or gas in the chest cavity (pneumothorax) is possible, although very rare.

Blood Draw Risks:

- The risks of drawing blood include temporary discomfort from the needle stick and bruising.

CONFIDENTIAL

Reproductive Risks:

- You should not be or become pregnant while on this study. You should not breastfeed a baby while on this study. If you are pregnant you cannot be in the study. To participate in this study you must agree to use an effective double barrier method of birth control to prevent pregnancy while receiving treatment. Check with your study doctor about the methods of birth control to prevent pregnancy and how long to use them.

Unknown Risks

- The experimental treatments may have side effects that no one knows about yet. The researchers and study doctor will let you know if they learn anything that might make you change your mind about participating in the study.

Are there benefits to taking part in the study?

Taking part in this study may be better, worse or make no difference in your health. We don't know if the combination of these new investigational drugs with standard chemotherapy will be more effective than standard chemotherapy alone. That is why we are doing this study. This research study should help us learn if the investigational drugs given together with standard chemotherapy treatment are likely to benefit a participant more than standard chemotherapy treatment alone.

No matter what, we will learn more about your cancer. We will learn more about what does and does not help in the treatment of your cancer. What we learn may help others with cancer.

What other choices do I have if I do not take part in this study?

You may choose to not participate in this study. Your option to participate in this study will not affect the care you receive here for your breast cancer in any way.
Your choices may include:

- Getting treatment or care for your cancer without being in a study
- You may choose to have surgery first without taking part in this study
- Taking part in another study
- Getting no treatment

Please talk to your doctor about your choices before deciding if you will take part in this study.

Will my medical information be kept private?

We will do our best to make sure that the personal information in your medical record is kept private. An electronic copy of portions of your medical record will be used in this study through an encrypted secure server. All of your personal information will be removed from these electronic records and you will only be identified by a study number. However, we cannot guarantee total privacy. Your personal information may be given out if required by law. If information from this study is published or presented at a scientific meeting, your name and other personal information will not be used.

Organizations that may look at and/or copy your medical records for research quality assurance and data analysis include:

CONFIDENTIAL

- QuantumLeap Healthcare Collaborative (QLHC) as the sponsor of this study or their authorized designee
- The National Cancer Institute (NCI) for regulatory oversight of this study
- The Food and Drug Administration (FDA) for regulatory oversight of this study
- The American College of Radiology Imaging Network (ACRIN) for MRI data analysis
- Local Institutional Review Boards (IRB) and government agencies, such as the office for Human Research Protections (OHRP) and the Data and Safety Monitoring Board (DSMB) that are involved in keeping research safe for people.

Health Insurance companies/group health plans may not request your genetic information that we get from your participation in this study. It is against federal law, Genetic Information Nondiscrimination Act (GINA), for a health insurance company/group health plans and most employers to discriminate against you based on your genetic information. By taking part in this study, your genetic information will not be shared according to the GINA federal law.

What are the costs of taking part in this study?

Taking part in this study may lead to added costs to you or your insurance carrier. Please ask about any expected additional costs or insurance problems.

You and/or your health plan/insurance company will need to pay for some or all of the costs of treating your cancer in this study. Some health plans will not pay for these costs if you are taking part in a research study. Check with your health plan/insurance company to find out what they will pay for. Taking part in this study may or may not cost you or your insurance company more than the cost of getting regular cancer treatment.

The study will cover the following study related tests and procedures done during the treatment phase:

- The MRI scans done 3 weeks after you start chemotherapy, before you start your AC treatment, and before surgery (only if it is done for the purpose of this study)
- The core biopsy done 3 weeks after you start chemotherapy
- The blood collected for research for this study
- If you are assigned to an investigational drug, the study will cover the cost of the investigational drug. Any other medications will be the responsibility of you and/or your health plan/insurance company.

You or your insurance company will be charged for continuing medical care and/or hospitalization.

For more information on clinical trials and insurance coverage, you can visit the National Cancer Institute's Web site at http://cancer.gov/clinicaltrials/insurance. You can print a copy of the "Clinical Trials and Insurance Coverage" information from this Web site. Another way to get the information is to call 1-800-4-CANCER (1-800-422-6237) and ask them to send you a free copy.

Will I be paid for taking part in this study?

CONFIDENTIAL

You will not receive payment for taking part in the study.

Reimbursement of $100 may be provided for those who complete the treatment phase of this study. This is to help reduce the cost related to travel or parking for study visits. Please ask your study doctor or coordinator for more details.

What happens if I am injured because I took part in this study?

It is important that you tell your study doctor(fill in Investigators name(s)), if you feel that you have been injured because of taking part in this study. You can contact Dr. (fill in) or their associates at xxx-xxx-xxxx.

If you are injured as a result of being in this study, treatment will be available. You and/or your health plan will be charged for this treatment. The study will not pay for medical treatment. The costs of such treatment may be covered by (insert University) and the study sponsor, depending on a number of factors. The University does not normally provide any other form of compensation for injury. For further information about this, you may call the office of the (insert IRB) at xxx-xxx-xxxx.

What are my rights if I take part in this study?

Taking part in this study is your choice. You may choose either to take part or not to take part in the study. If you decide to take part in this study, you may leave the study at any time. No matter what decision you make, there will be no penalty to you and you will not lose any of your regular benefits. Leaving the study will not affect your medical care. You can still get your medical care from our institution.

We will tell you about new information or changes in the study that may affect your health or your willingness to continue in the study.

In the case of injury resulting from this study, you do not lose any of your legal rights to seek payment by signing this form.

Who can answer my questions about the study?

This study has been explained to you by Dr. (fill in) or the person who signed below. If you have any other questions about the study, you may call Dr. (fill in) at xxx-xxx-xxxx**.** Alternatively, you can call the study coordinator,(fill in), at xxx-xxx-xxxx.

If you wish to ask questions about the study or your rights as a research participant to someone other than the researchers or if you wish to voice any problems or concerns you may have about the study, please call (fill in your site) IRB at xxx-xxx-xxxx.

Where can I get more information?

You may call the NCI's Cancer Information Service at **1–800–4–CANCER (1–800–422–6237).** Visit the NCI's Web sites for comprehensive clinical trials information (http://www.cancer.gov/clinicaltrials) or for accurate cancer information (http://www.cancer.gov)

CONFIDENTIAL

including PDQ®, an NCI database that contains the latest information about cancer treatment, screening, prevention, genetics, supportive care, and complementary and alternative medicine, plus clinical trials.

For more general information about this trial, you can visit the study's website at: http://www.ispy2.org.

A description of this clinical trial will be available on http://www.ClinicalTrials.gov, as required by US law. This website will not include information that can identify you. At most, the website will include a summary of the study results. You can search this website at any time. The I-SPY 2 TRIAL Identifier is NCT01042379. Enter this identifier into the search engine on ClinicalTrials.gov for information on the I-SPY 2 TRIAL.

Optional Research Studies:
Collection of Samples and Use of Research Material
What about the use of my tissue and blood for research

You will have tissue and blood taken during the course of this study. Your tissue and blood will be used for research as part of this study to learn more about the biology of your breast cancer. The researchers think that they will use all of the blood and tissue samples for this study. However, if any of your tissue or blood is leftover, the researchers would like to keep your extra samples, for up to 10 years, and use them for research. **We would like your permission to use any leftover tissue and blood for future research within or outside this study.**

Your tissue and blood will be used only for research and will not be sold. You will not be paid for allowing your tissue to be used in research even though the research done with your tissue may help to develop new products in the future. You will not receive any payment or financial benefit from any products, tests, or discoveries derived from these samples.

What are the benefits of using your tissue and blood for research?

The research that will be done with your tissue and blood may not help you. It might help people who have cancer and other diseases in the future. The benefits of research using tissue and blood include learning more about what causes cancer and other diseases, how to prevent them, how to treat them, and how to cure them.

What are the risks of using your tissue and blood for research?

Your tissue and blood samples will not be identified using your personal information. Your personal information will not be shared with the researchers. However, the researchers may have access to information about your health. There is a very small chance that your personal information may be released. We will do our best to make sure that your personal information is kept private.

You can still be a part of the study even if you do not want your tissue and blood used for future research. If you agree to have your tissue and blood used for research, you can

CONFIDENTIAL

change your mind at any time. Let your study doctor know if you change your mind and your samples will no longer be used for research.

I agree to have my tissue and blood be used for future research.

Yes_________ No_________ Participant Initials_________

Consent

You will be given copies of this signed consent form to keep. You have already received copies of your signed screening consent form and the HIPAA form.

This is the second consent form for this study. By signing this consent form you are agreeing to participate in the treatment phase of this study. This includes your agreement to receive the treatment you have been randomized to.

PARTICIPATION IN RESEARCH IS VOLUNTARY. You have the right to decline to participate or withdraw at any point in this study without jeopardy to your medical care. Leaving the study will not result in any penalty or loss of benefits to which you are entitled. The study doctor may decide to take you off this study with or without your consent if it is in your best medical interest, funding is stopped, your condition worsens, or new information becomes available.

SIGNATURE

I have read all the above, asked questions, and received answers concerning areas I did not understand.

If you wish to participate in this study, you should sign below.

Participant Name: ____

Participant Signature: ____ Date: _____________

Signature of Doctor: ____ Date: _____________

Signature of Person Obtaining Consent: ________________________ Date: _____________

If Necessary

Translator Name: ____

Signature of Translator: ____________________________________ Date: _____________

CONFIDENTIAL

I-SPY 2 TRIAL
(*Investigation of Serial Studies to Predict Your Therapeutic Response with Imaging And moLecular Analysis 2*)

SUPPLEMENT FOR TRASTUZUMAB TREATMENT

You have already been given the treatment consent form for this study. This section of the consent form will only address the side effects and risks of this specific drug you will receive with paclitaxel and the treatment schedule.

You have been randomized to the treatment group that receives Trastuzumab along with Paclitaxel, the standard of care for participants who are HER2+.

You will receive the following combination of drugs:

Drug Name	How treatment will be given	How long treatment will take each time	How often treatment is given	When treatment will be given
Paclitaxel	By vein	1 hour	Weekly	Weeks 1–12
Trastuzumab	By vein	1 hour	Weekly	Weeks 1–12

This treatment regimen will last for a total of 12 weeks, after which you will receive AC as described in the treatment consent form.

The FDA has recently granted accelerated approval for the use of pertuzumab (Perjeta) for HER2-positive breast cancer in combination with trastuzumab (Herceptin) and docetaxel (Taxotere) in the neoadjuvant (pre-surgical) setting in early breast cancer. Pertuzumab is an antibody that binds to a different part of the HER2 protein than trastuzumab.

The FDA grants accelerated approval to drugs that show early promise against a serious disease. Pertuzumab was approved as neoadjuvant treatment based on a trial of women who took pertuzumab, trastuzumab and docetaxel vs docetaxel and trastuzumab alone. More women who took the pertuzumab regimen had no sign of their cancer present in their breast or lymph nodes at the time of surgery than those women who did not take this regimen. Currently, physicians are allowed to give pertuzumab to patients receiving neoadjuvant treatment while Genentech, the company that makes pertuzumab, continues to collect more data to understand if there is a long-term benefit of pertuzumab for patients who have HER2-positive early breast cancer. Pertuzumab has also been granted full FDA approval for the treatment of HER2-positive metastatic breast cancer, based on studies in which adding pertuzumab to trastuzumab with docetaxel slowed the progression of HER2-positive metastatic disease.

The chemotherapy drugs used in pertuzumab's accelerated approval are different from the drugs we are using in I-SPY 2. We do not know if our treatment is the same, better, or worse.

It is important to discuss this with your doctor. By signing this consent, it means that you have talked with your doctor about the possible role that pertuzumab can have in your treatment.

CONFIDENTIAL

What is Trastuzumab?

Trastuzumab, also known as Herceptin, is an approved drug by the FDA and is the standard treatment for women with Her2 positive breast cancer. Trastuzumab is a specific type of antibody that binds to Her2 proteins on cells. Antibodies are a type of protein made by cells to attack a foreign substance that the cell thinks is harmful. By interacting with Her2 proteins the cancer cells may grow slower.

What additional tests and procedures can I expect from this additional drug while on the study?

To help monitor your health while receiving this treatment, you will have the following additional tests and procedures:

- ECHO or MUGA scan after you complete your paclitaxel and trastuzumab treatment but before you start your AC treatment
- ECHO or MUGA scan after you complete your AC treatment but before you have surgery

You would have both of these scans done as standard of care, even if you did not join the study. The cost of these scans will be covered by you and/or your health plan/insurance company.

What side effects or risks can I expect from these additional drugs while on the study?

You may have side effects while on the study. Everyone taking part in the study will be watched carefully for any side effects. However, doctors and the study sponsor don't know all the side effects that may happen and there may be unknown side effects that could occur. Side effects may be mild or very serious. Your health care team may give you medicines to help lessen the side effects. Many side effects go away soon after you stop what is causing them. In some cases, side effects can be serious or long lasting, causing hospitalization, and/or may never go away. There is also a risk of death. You may experience unexpected side effects that may not allow you to complete your standard treatment.

You should talk to your study doctor about any side effects you experience while taking part in the study.

Reproductive risks:

- If you suspect that you have become pregnant at any time during the study or within 2 months after your last dose of chemotherapy, please notify your study doctor immediately. It is not known whether these drugs can cause harm to the fetus when administered to a pregnant woman or if it affects the ability of a woman to become pregnant; therefore, you should not become pregnant.

CONFIDENTIAL

Potential Side Effects of Standard Treatment

	Side Effects		
**Drug Treatment**	**Likely**<br>**(seen in more than 20% of participants)**	**Less Likely**<br>**(Seen in 20% or less of participants)**	**Rare but Serious**<br>**(Seen in 2–3% or less of participants but might be serious)**
**Paclitaxel + Trastuzumab**	• Diarrhea<br>• Nausea<br>• Vomiting<br>• Fatigue<br>• Decreased appetite<br>• Hair loss (alopecia)<br>• Chills, fever, and low blood pressure during and after Trastuzumab is given<br>• Temporary lowering of the number of white blood cells (neutropenia) which could result in fever and increased risk of infection<br>• Temporary lowering of red blood cells (anemia)<br>• Temporary lowering of blood platelet cells (thrombocytopenia)<br>• Numbness or tingling in the fingers or the toes which may continue after you have stopped treatment (neuropathy)<br>• Temporary pain in the muscles and joints	• Weakening of the heart muscle, which could result in heart failure<br>• Fluid in the lungs and inflammation of the lungs, which can lead to difficulty or the inability to breath<br>• Shortness of breath or cough<br>• Allergic reactions including hives<br>• Fluid around the heart or inflammation of the heart<br>• Sores in the mouth or throat (like canker sores)<br>• Abnormal liver function as seen on a blood test<br>• Flu-like symptoms:<br>- headache<br>- muscle ache<br>- bone ache<br>- loss of appetite<br>• Voice changes	• Severe allergic reaction<br>• Severe lung disease causing great difficulty breathing<br>• Irregular heart beat- this sometimes leads to fainting or heart stopping<br>• Low blood pressure<br>• Liver failure

CONFIDENTIAL

CONSENT

You will be given copies of this consent form to keep along with the standard chemotherapy treatment consent form.

PARTICIPATION IN RESEARCH IS VOLUNTARY. You have the right to decline to participate or withdraw at any point in this study without jeopardy to your medical care. Leaving the study will not result in any penalty or loss of benefits to which you are entitled. The study doctor may decide to take you off this study with or without your consent if it is in your best medical interest, funding is stopped, your condition worsens, or new information becomes available.

If you wish to participate in this study, you should sign below.

Participant Signature: ____ Date: _____________

Signature of Doctor: ______________________________________ Date: _____________

Signature of Person Obtaining Consent: _________________________ Date: _____________

Signature of Translator: _____________________________________ Date: _____________

SUPPLEMENT FOR FIGITUMUMAB TREATMENT

This agent was never activated in the trial (no participants ever received this agent), so the information regarding it has been removed.

I-SPY 2 TRIAL

(*I*nvestigation of *S*erial Studies to *P*redict *Y*our *T*herapeutic *R*esponse with *I*maging *A*nd mo*L*ecular Analysis *2*)

SUPPLEMENT FOR NERATINIB TREATMENT

You have already been given the treatment consent form for this study. This section of the consent form will only address the side effects and risks of this specific drug you will receive with paclitaxel and the treatment schedule.

You have been randomized to the treatment group that receives Neratinib along with Paclitaxel.

You will receive the following combination of drugs:

Drug Name	How treatment will be given	How long treatment will take each time	How often treatment is given	When treatment will be given
Paclitaxel	By vein	1 hour	Weekly	Weeks 1–12
Neratinib	Orally with food	A few minutes	Daily	Weeks 1–12

On days where you receive both paclitaxel and Neratinib, you should take Neratinib after the paclitaxel.

This treatment regimen will last for a total of 12 weeks after which you will receive AC as described in the treatment consent form.

If you are HER2 positive it is standard of care to receive a year of Trastuzumab, also known as Herceptin. You are still eligible to receive the full year of Trastuzumab following your surgery. For more details, talk to your study doctor.

What is Neratinib?

Neratinib is an investigational drug, meaning it has not been approved by the FDA. Neratinib is a small molecule that binds to Her2 proteins in cells. By binding to this protein cancer cells may grow slower.

What additional tests and procedures can I expect from this additional drug while on the study?

To help monitor your health while receiving this treatment, you will have the following additional tests and procedures:

- Laboratory safety blood tests specific for neratinib will be added to your standard of care safety blood tests
- ECHO or MUGA scan after you complete your paclitaxel and neratinib treatment but before you start your AC treatment
- ECHO or MUGA scan after you complete your AC treatment but before you have surgery

The cost of these additional tests and procedures will be covered by the study.

CONFIDENTIAL

What side effects or risks can I expect from these additional drugs while on the study?

You may have side effects while on the study. Everyone taking part in the study will be watched carefully for any side effects. However, doctors and the study sponsor don't know all the side effects that may happen and there may be unknown side effects that could occur. Side effects may be mild or very serious. Your health care team may give you medicines to help lessen the side effects. Many side effects go away soon after you stop what is causing them. In some cases, side effects can be serious or long lasting, causing hospitalization, and/or may never go away. There is also a risk of death. You may experience unexpected side effects that may not allow you to complete your standard treatment. While it is extremely unlikely, these side effects may be long lasting and may possibly prevent you from receiving future standard or experimental therapies.

You should talk to your study doctor about any side effects you experience while taking part in the study.

You should also be aware that some investigational drugs will stop being assigned to new participants entering the trial as the trial continues. Once the study has gathered enough information about a particular investigational drug (good or bad), it will leave the trial and another new investigational drug will enter the trial. If the investigational drug leaves the study while you are still receiving it, you have 3 choices:

1. Continue taking the investigational drug and continue the treatment phase of the trial.
2. Stop taking the investigational drug and continue the treatment phase of the trial.
3. Stop taking the investigational drug and stop participating in the rest of the treatment phase of the trial.

Also, the investigational drug may leave the study because of severe side effects. If the investigational drug is removed for this reason while you are still receiving the drug, you will stop receiving the drug immediately. You will have the choice to continue or stop participating in the study.

The pharmaceutical company that manufactures this drug, Pfizer, will be notified of all side effects and any pregnancy that you may experience while you are part of this study.

Reproductive risks:

- You should not become pregnant while on this study. If you suspect that you have become pregnant at any time during the study or within 2 months after your last dose of chemotherapy, please notify your study doctor immediately. It is not known whether the investigational drugs can cause harm to the fetus when administered to a pregnant woman or if it affects the ability of a woman to become pregnant.

Drug Interactions:

- There are many prescription and over the counter drugs and dietary supplements (sometimes called complementary or alternative medicines) that may interact with neratinib. These drug interactions could lead to increased side effects. Your study doctor will review all of the medications and supplements you are currently taking before starting this treatment. You should also tell your study doctor if you take St. John's Wort or other herbal supplements, or if you eat grapefruit or drink grapefruit juice as this may also interact with neratinib. You should not take any new medications or supplements, including those prescribed by other doctors, without first discussing it with your study

CONFIDENTIAL

doctor or study pharmacist. Drugs that should be avoided while taking neratinib are shown below.

Drugs to Avoid while taking Neratinib

Generic Drug Name	Drug Brand Name®
Clarithromycin	Biaxin
Digoxin	Cardoxin, Digitek, Lanoxin, Lanoxicaps
Erythromycin	Eyrc, E-Mycin
Fluconazole	Diflucan
Fluvoxamine	Luvox
Indinavir	Crixivan
Itraconazole	Sporanox
Ketoconazole	Nizoral, Kuric, Xolegel, Extina
Mibefradil	Posicor
Miconazole	Desenex, Miconazex, Monistat
Nefazodone	Serzone
Nelfinavir	Viracept
Norfluoxetine	----
Quinine	Qualaquin, QM-260
Ritonavir	Norvir
Saquinavir	Invirase, Fortovase
Sertraline	Zoloft, Lustral
Troleandomycin	TAO
Voriconazole	Vfend
Zafirlukast	Accolate

CONFIDENTIAL

Potential Side Effects of Investigational Treatment

	Side Effects		
**Drug Treatment**	**Likely**<br>**(seen in 20% or more of participants)**	**Less Likely**<br>**(Seen in less than 20% of participants)**	**Rare but Serious**<br>**(Seen in 2–3% or less of participants but might be serious)**
**Paclitaxel + Neratinib**	• Diarrhea<br>• Nausea<br>• Fatigue<br>• Decrease in appetite<br>• Weight loss<br>• Temporary lowering of the number of white cells (neutropenia) which could result in fever and increased risk of infection	• Abnormal liver function seen on a blood test<br>• Abdominal pain<br>• Joint pain<br>• Back pain<br>• Headache<br>• Weakness<br>• Dizziness<br>• Dehydration	• Kidney failure seen on a blood test (increase in creatinine)<br>• Bone marrow suppression symptoms:<br>– high fevers<br>– cough<br>– chills<br>– shortness of breath<br>– nose bleeds

	Side Effects		
**Drug Treatment**	**Likely** (seen in 20% or more of participants)	**Less Likely** (Seen in less than 20% of participants)	**Rare but Serious** (Seen in 2–3% or less of participants but might be serious)
	• Temporary lowering of the number red blood cells (anemia)<br>• Vomiting<br>• Rash<br>• Hair loss (alopecia)<br>• Numbness or tingling in the fingers or the toes (neuropathy)		– gum bleeding<br>– bruising<br>• Lowering of blood platelet cells (thrombocytopenia) Pneumonia<br>• Pulmonary embolism<br>• Shock<br>• Intestinal obstruction<br>• Electrolyte abnormalities (as seen on a blood test)<br>• Low blood pressure<br>• Cardiac abnormalities<br>• Urinary tract infection<br>• Herpes eye infection

If you experience multiple loose bowel movements in a day or any worsening of fatigue, nausea, vomiting, abdominal pain or tenderness, fever or rash, notify your study doctor immediately.

One participant with non-small cell lung cancer who was treated with neratinib experienced interstitial lung disease (an inflammation of the lungs that is similar to pneumonia). This lung problem could have been caused by neratinib. The participant's health improved when neratinib was stopped and they began to take steroids and anti-infection medication to treat this side effect. If you feel shortness of breath along with fever or cough, please let your study doctor know immediately.

Although only one case has been reported, bone marrow suppression can be life threatening. If you experience any of the symptoms listed in the table above, notify your doctor immediately.

Unknown Risks:
Drugs similar to neratinib have side effects that included weakening of the heart muscles which could result in heart failure, which has symptoms of shortness of breath and/or fatigue. There have been reports of changes in liver function tests in participants taking drugs similar to neratinib and in some of those cases the liver damage has resulted in death. These side effects have not been seen in participants treated with neratinib, but it is possible that you could experience these side effects. Let your study doctor know if you experience any of these symptoms.

CONSENT

You will be given copies of this consent form to keep along with the standard chemotherapy treatment consent form.

PARTICIPATION IN RESEARCH IS VOLUNTARY. You have the right to decline to participate or withdraw at any point in this study without jeopardy to your medical care. Leaving the study will not result in any penalty or loss of benefits to which you are entitled. The study doctor may decide to take you off this study with or without your consent if it is in your best medical interest, funding is stopped, your condition worsens, or new information becomes available.

CONFIDENTIAL

If you wish to participate in this study, you should sign below.

Participant Signature: ____ Date: _____________

Signature of Doctor: ____ Date: _____________

Signature of Person Obtaining Consent: _________________________ Date: _____________

Signature of Translator: ______________________________________ Date: _____________

CONFIDENTIAL

I-SPY 2 TRIAL
(*Investigation of Serial Studies to Predict Your Therapeutic Response with Imaging And moLecular Analysis 2*)

SUPPLEMENT FOR ABT 888 AND CARBOPLATIN TREATMENT

You have already been given the treatment consent form for this study. This section of the consent form will only address the side effects and risks of these specific drugs you will receive with paclitaxel and the treatment schedule.

You have been randomized to the treatment group that receives ABT 888 and Carboplatin along with Paclitaxel.

You will receive the following combination of drugs:

Drug Name	How treatment will be given	How long treatment will take each time	How often treatment is given	When treatment will be given
Paclitaxel	By vein	1 hour	Weekly	Weeks 1–12
ABT 888	Orally	A few minutes	2 times a day	Weeks 1–12
Carboplatin	By vein	30 minutes	Every 3 weeks	Weeks 1, 4, 7, 10

This treatment regimen will last for a total of 12 weeks after which you will receive AC as described in the treatment consent form.

What are ABT 888 and Carboplatin?

ABT 888 is an investigational drug, meaning it has not been approved by the FDA. ABT 888 is a molecule that prevents proteins poly(ADP-ribose) polymerase [PARP]-1 and -2 from interacting with other proteins in cells. By preventing these interactions the cancer cells may stop growing.

Carboplatin is an approved drug by the FDA. Carboplatin is a molecule that binds to DNA and stops cancer cells from growing. Giving all 3 drugs together may help kill more cancer cells.

What side effects or risks can I expect from these additional drugs while on the study?

You may have side effects while on the study. Everyone taking part in the study will be watched carefully for any side effects. However, doctors and the study sponsor don't know all the side effects that may happen and there may be unknown side effects that could occur. Side effects may be mild or very serious. Your health care team may give you medicines to help lessen the side effects. Many side effects go away soon after you stop what is causing them. In some cases, side effects can be serious or long lasting, causing hospitalization, and/or may never go away. There is also a risk of death. You may experience unexpected side effects that may not allow you to complete your standard treatment. While it is extremely unlikely, these side effects may be long lasting and may possibly prevent you from receiving future standard or experimental therapies.

You should talk to your study doctor about any side effects you experience while taking part in the study.

You should also be aware that some investigational drugs will stop being assigned to new participants entering the trial as the trial continues. Once the study has gathered enough

CONFIDENTIAL

information about a particular investigational drug (good or bad), it will leave the trial and another new investigational drug will enter the trial. If the investigational drug leaves the study while you are still receiving it, you have 3 choices:

1. Continue taking the investigational drug and continue the treatment phase of the trial.
2. Stop taking the investigational drug and continue the treatment phase of the trial.
3. Stop taking the investigational drug and stop participating in the rest of the treatment phase of the trial.

Also, the investigational drug may leave the study because of severe side effects. If the investigational drug is removed for this reason while you are still receiving the drug, you will stop receiving the drug immediately. You will have the choice to continue or stop participating in the study.

The pharmaceutical company that manufactures this drug, Abbott, will be notified of all side effects and any pregnancy that you may experience while you are part of this study.

Unknown Human Risks

In animal studies using doses of ABT 888 that were higher than the dose in this study, the side effects included seizures, loss of weight, decreases in white and red blood cells, and damage to the testis and ovaries. It is not known if these side effects will occur in humans.

Reproductive risks:

- You should not become pregnant while on this study. If you suspect that you have become pregnant at any time during the study or within 3 months after your last dose of chemotherapy, please notify your study doctor immediately. It is not known whether the investigational drugs can cause harm to the fetus when administered to a pregnant woman or if it affects the ability of a woman to become pregnant.

Potential Side Effects of Investigational Treatment

	Side Effects		
**Drug Treatment**	**Likely (seen in 20% or more of participants)**	**Less Likely (Seen in less than 20% of participants)**	**Rare but Serious (Seen in 2–3% or less of participants but might be serious)**
**Paclitaxel + Carboplatin+ ABT 888**	• Fatigue<br>• Decreased appetite<br>• Nausea<br>• Diarrhea<br>• Vomiting<br>• Abdominal pain and swelling<br>• Headache<br>• Constipation<br>• Hair loss<br>• Temporary lowering of the number of white blood cells (neutropenia, lymphopenia) which could result in fever	• Dizziness<br>• Allergic reaction<br>• Weight loss<br>• Changes in heart function as monitored by blood test or ECG (troponin, fast heart beat)<br>• Swelling in legs, hands and feet because of fluid retention<br>• High blood pressure<br>• Kidney toxicity (as measured by a laboratory test)<br>• Urinary tract infection<br>• Blurred vision<br>• Ringing in ears<br>• Flatulence<br>• Mouth sores	• Shortness of breath with cough, respiratory failure<br>• Decreased oxygen in blood<br>• Pneumonia, other serious infections<br>• Fluid around lungs (pleural effusion)<br>• Blood clot in blood vessels (thrombosis, pulmonary embolism)<br>• Blockage of small intestine<br>• Dehydration<br>• Fever<br>• Seizures

CONFIDENTIAL

Drug Treatment	Side Effects		
	**Likely** (seen in 20% or more of participants)	**Less Likely** (Seen in less than 20% of participants)	**Rare but Serious** (Seen in 2–3% or less of participants but might be serious)
	and increased risk of infection<br>• Temporary lowering of the number of red blood cells (anemia)<br>•<br>• Temporary lowering of the number of platelets (help with blood clotting)<br>• Difficulty falling asleep	• Dry mouth, decreased saliva<br>• Feeling anxious or depressed<br>• Indigestion<br>• Hot flushing<br>• Sinus infection or runny nose<br>• Nose bleeds<br>• Chest pain, body aches, joint pain<br>• Neuropathy (tingling, numbness and/or sensitive to touch)<br>• Changes in blood electrolytes (sodium, potassium, calcium, magnesium)<br>• Liver toxicity (as measured by a laboratory test)<br>• Decrease in blood protein (hypoalbuminemia)<br>• Increase in blood glucose (hyperglycemia)	

ABT 888 must only be taken by the person it has been prescribed to. It should be kept out of reach of children and persons who cannot read or are not capable of handling drugs. For safety, people other than the participant in the study should not handle broken tablets.

CONSENT
You will be given copies of this consent form along with the standard chemotherapy treatment consent form.

PARTICIPATION IN RESEARCH IS VOLUNTARY. You have the right to decline to participate or withdraw at any point in this study without jeopardy to your medical care. Leaving the study will not result in any penalty or loss of benefits to which you are entitled. The study doctor may decide to take you off this study with or without your consent if it is in your best medical interest, funding is stopped, your condition worsens, or new information becomes available.

If you wish to participate in this study, you should sign below.

Participant Signature: ____ Date: ____________

Signature of Doctor: ______________________________________ Date: ____________

Signature of Person Obtaining Consent: ______________________ Date: ____________

Signature of Translator: ___________________________________ Date: ____________

CONFIDENTIAL

I-SPY 2 TRIAL
(Investigation of Serial studies to Predict Your Therapeutic Response with Imaging And moLecular analysis 2)

SUPPLEMENT FOR CONATUMUMAB TREATMENT

This agent was never activated in the trial (no participants ever received this agent), so the information regarding it has been removed.

I-SPY 2 TRIAL

(Investigation of Serial studies to Predict Your Therapeutic Response with Imaging And moLecular analysis 2)

SUPPLEMENT FOR AMG 386 TREATMENT

You have already been given the treatment consent form for this study. This section of the consent form will only address the side effects and risks of this specific drug you will receive with paclitaxel and the treatment schedule.

You have been randomized to the treatment group that receives AMG 386 along with Paclitaxel.

You will receive the following combination of drugs:

Drug Name	How treatment will be given	How long treatment will take each time	How often treatment is given	When treatment will be given
Paclitaxel	By vein	1 hour	Weekly	Weeks 1–12
AMG 386	By vein	1 hour or less	Weekly	Weeks 1–12

This treatment regimen will last for a total of 12 weeks after which you will receive AC as described in the treatment consent form.

What is AMG 386?
AMG 386 is an investigational drug, meaning it has not been approved by the FDA. AMG 386 is a small molecule that binds to angiopoietin 1 and 2, proteins that help in the growth of new blood vessels in cancer cells. AMG 386 works by preventing these proteins from finding their target. By blocking this interaction the cancer cells may grow slower.

What additional tests and procedures can I expect from this additional drug while on the study?

To help monitor your health while receiving this treatment, you will have the following additional tests and procedures:

- Laboratory safety tests specific for AMG 386 will be added to your standard of care safety blood tests
- Three additional blood samples will be taken (about 1 teaspoon) for research purposes.
  - The first blood draw will be done before you start your treatment
  - The second blood draw will be done in week 9 of your treatment
  - The third blood draw will be done 30 days after you complete your treatment with AMG 386

The cost of these additional tests will be covered by the study.

What side effects or risks can I expect from these additional drugs while on the study?

CONFIDENTIAL

You may have side effects while on the study. Everyone taking part in the study will be watched carefully for any side effects. However, doctors and the study sponsor don't know all the side effects that may happen and there may be unknown side effects that could occur. Side effects may be mild or very serious. Your health care team may give you medicines to help lessen the side effects. Many side effects go away soon after you stop what is causing them. In some cases, side effects can be serious or long lasting, causing hospitalization, and/or may never go away. There is also a risk of death. You may experience unexpected side effects that may not allow you to complete your standard treatment. While it is extremely unlikely, these side effects may be long lasting and may possibly prevent you from receiving future standard or experimental therapies.

You should talk to your study doctor about any side effects you experience while taking part in the study.

You should also be aware that some investigational drugs will stop being assigned to new participants entering the trial as the trial continues. Once the study has gathered enough information about a particular investigational drug (good or bad), it will leave the trial and another new investigational drug will enter the trial. If the investigational drug leaves the study while you are still receiving it, you have 3 choices:

1. Continue taking the investigational drug and continue the treatment phase of the trial.
2. Stop taking the investigational drug and continue the treatment phase of the trial.
3. Stop taking the investigational drug and stop participating in the rest of the treatment phase of the trial.

Also, the investigational drug may leave the study because of severe side effects. If the investigational drug is removed for this reason while you are still receiving the drug, you will stop receiving the drug immediately. You will have the choice to continue or stop participating in the study.

The pharmaceutical company that manufactures this drug, Amgen, will be notified of all serious side effects that you may experience while you are part of this study.

Reproductive risks:

- You should not become pregnant while on this study. If you suspect that you have become pregnant at any time during the study or within 6 months after your last dose of chemotherapy, please notify your study doctor immediately. It is not known whether the investigational drugs can cause harm to the fetus when administered to a pregnant woman or if it affects the ability of a woman to become pregnant.

Drug Interactions:

- If you are sensitive to the following compounds, let your study doctor know as you might experience increased side effects:
  - Histidine
  - Mannitol
  - Sucrose
  - Arginine
  - Hydrochloride
  - Polysorbate 20

AMG 386 Antibody Formation:

Occasionally, your body may respond to this drug while it is being administered. Your body may make antibodies against this drug that can be found in your blood. If you have these antibodies, it may or may not cause a side effect. It is also possible that these antibodies may decrease the

CONFIDENTIAL

ability of this drug to treat your disease and this may impact your ability to receive this drug anymore. Your blood may be checked for these antibodies.

Potential Side Effects of Investigational Treatment

	Side Effects		
**Drug Treatment**	**Likely (seen in 10% or more of participants)**	**Less Likely (Seen in 1-10% of participants)**	**Rare but Serious (Seen in 1% or less of participants but might be serious)**
**Paclitaxel + AMG 386**	• Swelling of the legs and feet<br>• Increased fluid or swelling in the abdomen or chest cavity<br>• Fatigue<br>• Diarrhea<br>• Nausea<br>• Vomiting<br>• Decrease in appetite<br>• High blood pressure<br>• Muscle weakness or pain<br>• Allergic reaction symptoms:<br>-Rash<br>-Sweating<br>-Swelling or flushing of the skin<br>-Itching<br>-Change in heart rate<br>-Difficulty in breathing<br>-Low blood pressure	• Blurred vision<br>• Weight loss or gain<br>• Chest pain<br>• Headache<br>• Fever<br>• Flushing<br>• Dehydration<br>• Low thyroid function<br>• Abdominal pain, distension<br>• Sore mouth, mucosal inflammation of mouth, gums, throat<br>• Indigestion, gas, constipation<br>• Hair loss (alopecia)<br>• Rash, dry skin, itching, acne<br>• Pain and redness of the skin of the palms and soles of feet<br>• Nail disorders<br>• Abnormal taste<br>• Difficulty speaking<br>• Dizziness<br>• Insomnia<br>• Numbness or tingling in the fingers or toes which may continue after you have stopped treatment (neuropathy)<br>• Lowering number of white blood cells (neutropenia) which could result in fever and increased risk of infection<br>• Lowering number of red blood cells (anemia)<br>• Lowering number of platelets<br>• Abnormal laboratory blood levels including:<br>- low potassium<br>- low sodium<br>- low phosphate<br>- low magnesium<br>- elevated liver enzymes or bilirubin	• Bleeding, that may result in death<br>• Blood clots in a deep vein or artery<br>• Blood clots or blockage (*e.g.*, in lungs) that in some cases may cause death<br>• Fluid in the lungs<br>• Infection in the blood stream (sepsis)<br>• Heart attack or heart failure<br>• Perforations (holes)or abnormal connections (fistula) from one organ to another organ, that may result in death<br>• Blockage of the bowels or intestines<br>• Liver failure<br>• Breathing stopped (heart, respiratory arrest), that may result in death

CONFIDENTIAL

	Side Effects		
**Drug Treatment**	**Likely (seen in 10% or more of participants)**	**Less Likely (Seen in 1-10% of participants)**	**Rare but Serious (Seen in 1% or less of participants but might be serious)**
		• Cough<br>• Shortness of breath<br>• Runny nose, nose bleeds<br>• Blood clot in the veins of the arms, legs, or lungs<br>• Muscle spasms, weakness<br>• Joint pain, back pain<br>• Increased tears, swelling around the eyes	

CONFIDENTIAL

CONSENT

You will be given copies of this consent form along with the standard chemotherapy treatment consent form.

PARTICIPATION IN RESEARCH IS VOLUNTARY. You have the right to decline to participate or withdraw at any point in this study without jeopardy to your medical care. Leaving the study will not result in any penalty or loss of benefits to which you are entitled. The study doctor may decide to take you off this study with or without your consent if it is in your best medical interest, funding is stopped, your condition worsens, or new information becomes available.

If you wish to participate in this study, you should sign below.

Participant Signature: ______________________________ Date: ___________

Signature of Doctor: _______________________________ Date: ___________

Signature of Person Obtaining Consent: ________________ Date: ___________

Signature of Translator: ____________________________ Date: ___________

I-SPY 2 TRIAL

(Investigation of Serial studies to Predict Your Therapeutic Response with Imaging And moLecular analysis 2)

SUPPLEMENT FOR TRASTUZUMAB (HERCEPTIN) PLUS AMG 386 TREATMENT

You have already been given the treatment consent form for this study. This section of the consent form will only address the side effects and risks of this specific drug you will receive with paclitaxel and the treatment schedule.

You have been randomized to the treatment group that receives Trastuzumab (Herceptin) plus AMG 386 along with Paclitaxel.

You will receive the following combination of drugs:

Drug Name	How treatment will be given	How long treatment will take each time	How often treatment is given	When treatment will be given
Paclitaxel	By vein	1 hour	Weekly	Weeks 1–12
Trastuzumab	By vein	1 hour	Weekly	Weeks 1-12
AMG 386	By vein	1 hour or less	Weekly	Weeks 1–12

This treatment regimen will last for a total of 12 weeks, after which you will receive AC as described in the treatment consent form.

You are still eligible to receive the full year of trastuzumab following your surgery. For more details, talk to your study doctor.

The FDA has recently granted accelerated approval for the use of pertuzumab (Perjeta) for HER2-positive breast cancer in combination with trastuzumab (Herceptin) and docetaxel (Taxotere) in the neoadjuvant (pre-surgical) setting in early breast cancer. Pertuzumab is an antibody that binds to a different part of the HER2 protein than trastuzumab.

The FDA grants accelerated approval to drugs that show early promise against a serious disease. Pertuzumab was approved as neoadjuvant treatment based on a trial of women who took pertuzumab, trastuzumab and docetaxel vs docetaxel and trastuzumab alone. More women who took the pertuzumab regimen had no sign of their cancer present in their breast or lymph nodes at the time of surgery than those women who did not take this regimen. Currently, physicians are allowed to give pertuzumab to patients receiving neoadjuvant treatment while Genentech, the company that makes pertuzumab, continues to collect more data to understand if there is a long-term benefit of pertuzumab for patients who have HER2-positive early breast cancer. Pertuzumab has also been granted full FDA approval for the treatment of HER2-positive metastatic breast cancer, based on studies in which adding pertuzumab to trastuzumab with docetaxel slowed the progression of HER2-positive metastatic disease.

The chemotherapy drugs used in pertuzumab's accelerated approval are different from the drugs we are using in I-SPY 2. We do not know if our treatment is the same, better, or worse.

CONFIDENTIAL

It is important to discuss this with your doctor. By signing this consent, it means that you have talked with your doctor about the possible role that pertuzumab can have in your treatment.

What is Trastuzumab?

Trastuzumab, also known as Herceptin, is an approved agent by the FDA and is the standard treatment for women with HER2+ breast cancer. Trastuzumab is a specific type of antibody that binds to HER2 proteins on cells. Antibodies are a type of protein made by cells to attack a foreign substance that the cell thinks is harmful. By interacting with HER2 proteins, the cancer cells may grow more slowly.

What is AMG 386?

AMG 386 is an investigational drug, meaning it has not been approved by the FDA. AMG 386 is a small molecule that binds to angiopoietin 1 and 2, proteins that help in the growth of new blood vessels in cancer cells. AMG 386 works by preventing these proteins from finding their target. By blocking this interaction, the cancer cells may grow more slowly.

What additional tests and procedures can I expect from this additional drug while on the study?

To help monitor your health while receiving this treatment, you will have the following additional tests and procedures:

- Laboratory safety tests specific for AMG 386 will be added to your standard of care safety blood tests
- Three additional blood samples will be taken (about 1 teaspoon) for research purposes.
    - The first blood draw will be done before you start your treatment
    - The second blood draw will be done in week 9 of your treatment
    - The third blood draw will be done 30 days after you complete your treatment with AMG 386
- ECHO or MUGA scan after you complete your paclitaxel plus trastuzumab plus AMG 386 treatment but before you start your AC treatment
- ECHO or MUGA scan after you complete your AC treatment but before you have surgery

The cost of these additional laboratory safety tests and additional blood samples taken for research will be covered by the study. The cost of the ECHO/MUGA's will be covered by you and/or your health plan/insurance company, since you would have both of these scans done as standard of care, even if you did not join the study

What side effects or risks can I expect from these additional drugs while on the study?

You may have side effects while on the study. Everyone taking part in the study will be watched carefully for any side effects. However, doctors and the study sponsor don't know all the side effects that may happen and there may be unknown side effects that could occur. Side effects may be mild or very serious. Your health care team may give you medicines to help lessen the side effects. Many side effects go away soon after you stop what is causing them. In some cases, side effects can be serious or long-lasting, causing hospitalization, and/or may never go away. There is also a risk of death. You may experience unexpected side effects that may not allow you to complete your standard treatment. While it is extremely unlikely, these side effects

CONFIDENTIAL

may be long-lasting and may possibly prevent you from receiving future standard or experimental therapies.

You should talk to your study doctor about any side effects you experience while taking part in the study.

You should also be aware that some investigational drugs will stop being assigned to new participants entering the trial as the trial continues. Once the study has gathered enough information about a particular investigational drug (good or bad), it will leave the trial and another new investigational drug will enter the trial. If the investigational drug leaves the study while you are still receiving it, you have three choices:

1. Continue taking the investigational drug and continue the treatment phase of the trial.
2. Stop taking the investigational drug and continue the treatment phase of the trial.
3. Stop taking the investigational drug and stop participating in the rest of the treatment phase of the trial.

Also, the investigational drug may leave the study because of severe side effects. If the investigational drug is removed for this reason while you are still receiving the drug, you will stop receiving the drug immediately. You will have the choice to continue or stop participating in the study.

The pharmaceutical company that manufactures this drug, Amgen, will be notified of all serious side effects that you may experience while you are part of this study.

Reproductive risks:

- You should not become pregnant while on this study. If you suspect that you have become pregnant at any time during the study or within six months after your last dose of chemotherapy, please notify your study doctor immediately. It is not known whether the investigational drugs can cause harm to the fetus when administered to a pregnant woman or if it affects the ability of a woman to become pregnant.

Drug Interactions:

- If you are sensitive to the following compounds, let your study doctor know as you might experience increased side effects:
    - Histidine
    - Mannitol
    - Sucrose
    - Arginine
    - Hydrochloride
    - Polysorbate 20

AMG 386 Antibody Formation:

Occasionally, your body may respond to this drug while it is being administered. Your body may make antibodies against this drug that can be found in your blood. If you have these antibodies, it may or may not cause a side effect. It is also possible that these antibodies may decrease the ability of this drug to treat your disease and this may impact your ability to receive this drug anymore. Your blood may be checked for these antibodies.

CONFIDENTIAL

Potential Side Effects of Investigational Treatment

Side Effects			
**Drug Treatment**	**Likely (seen in 10% or more of participants)**	**Less Likely (Seen in 1-10% of participants)**	**Rare but Serious (Seen in 1% or less of participants but might be serious)**
**Paclitaxel + Trastuzumab + AMG 386**	• Swelling of the legs and feet<br>• Increased fluid or swelling in the abdomen or chest cavity<br>• Fatigue<br>• Diarrhea<br>• Nausea<br>• Vomiting<br>• Decrease in appetite<br>• High blood pressure<br>• Chills, fever and low blood pressure during and after Trastuzumab is given<br>• Muscle weakness or pain<br>• Allergic reaction symptoms:<br>-Rash<br>-Sweating<br>-Swelling or flushing of the skin<br>-Itching<br>-Change in heart rate<br>-Difficulty in breathing<br>• -Low blood pressure	• Blurred vision<br>• Weight loss or gain<br>• Chest pain<br>• Headache<br>• Fever<br>• Flushing<br>• Dehydration<br>• Low thyroid function<br>• Abdominal pain, distension<br>• Sore mouth, mucosal inflammation of mouth, gums, throat<br>• Indigestion, gas, constipation<br>• Hair loss (alopecia)<br>• Rash, dry skin, itching, acne<br>• Pain and redness of the skin of the palms and soles of feet<br>• Nail disorders<br>• Abnormal taste<br>• Difficulty speaking<br>• Dizziness<br>• Insomnia<br>• Numbness or tingling in the fingers or toes which may continue after you have stopped treatment (neuropathy)<br>• Lowering number of white blood cells (neutropenia) which could result in fever and increased risk of infection<br>• Lowering number of red blood cells (anemia)<br>• Lowering number of platelets<br>• Abnormal laboratory blood levels including:<br>• - low potassium<br>• - low sodium<br>• - low phosphate<br>• - low magnesium<br>• - elevated liver enzymes or bilirubin<br>• Cough<br>• Shortness of breath<br>• Runny nose, nose bleeds<br>• Blood clot in the veins of the arms, legs, or lungs<br>• Muscle spasms, weakness<br>• Joint pain, back pain<br>• Increased tears, swelling around the eyes	• Bleeding, that may result in death<br>• Blood clots in a deep vein or artery<br>• Blood clots or blockage (*e.g.*, in lungs) that in some cases may cause death<br>• Fluid in the lungs<br>• Infection in the blood stream(sepsis)<br>• Heart attack or heart failure<br>• Perforations (holes) or abnormal connections (fistula) from one organ to another organ, that may result in death<br>• Blockage of the bowels or intestines<br>• Liver failure<br>• Breathing stopped (heart, respiratory arrest), that may result in death

CONFIDENTIAL

CONSENT
You will be given copies of this consent form along with the standard chemotherapy treatment consent form.

PARTICIPATION IN RESEARCH IS VOLUNTARY. You have the right to decline to participate or withdraw at any point in this study without jeopardy to your medical care. Leaving the study will not result in any penalty or loss of benefits to which you are entitled. The study doctor may decide to take you off this study with or without your consent if it is in your best medical interest, funding is stopped, your condition worsens, or new information becomes available.

If you wish to participate in this study, you should sign below.

Participant Signature: ____________________________________ Date: _____________

Signature of Doctor: __________________________________ Date: _____________

Signature of Person Obtaining Consent: ___________________ Date: _____________

Signature of Translator: _______________________________ Date: _____________

CONFIDENTIAL

I-SPY 2 TRIAL
*(**I**nvestigation of **S**erial Studies to **P**redict **Y**our **T**herapeutic **R**esponse with **I**maging **A**nd moLecular Analysis **2**)*

SUPPLEMENT FOR GANITUMAB (AMG 479) TREATMENT

You have already been given the treatment consent form for this study. This section of the consent form will only address the side effects and risks of this specific drug you will receive with paclitaxel and the treatment schedule.

You have been randomized to the treatment group that receives Ganitumab along with Paclitaxel.

You will receive the following combination of drugs:

Drug Name	How treatment will be given	How long treatment will take each time	How often treatment is given	When treatment will be given
Paclitaxel	By vein	1 hour	Weekly	Weeks 1–12
Ganitumab	By vein	1 hour	Every 2 weeks	Weeks 1, 3, 5, 7, 9, 11

This treatment regimen will last for a total of 12 weeks, after which you will receive AC as described in the treatment consent form.

For 1 to 2 days before your first treatment with ganitumab, you should drink 8 glasses of fluid per day.

If you are not already on a regimen for diabetes management, you will be prescribed **Metformin**, an FDA-approved drug to control glucose (blood sugar) levels for people with diabetes. Because ganitumab may cause an elevation in blood glucose even in people without diabetes, you will receive metformin at no cost to you to prevent this from occurring unless you are already taking another oral medication to control diabetes. If you are taking another medication to control your diabetes, you will continue taking that medication as directed by your doctor.

What is Ganitumab?
Ganitumab is an investigational drug, meaning it has not been approved by the FDA. Ganitumab is a type of antibody that targets cancer cells. Antibodies are a type of protein made by cells to attack a foreign substance that the cell thinks is harmful. Ganitumab is a specially made antibody designed to block the insulin-like growth factor-1 receptor (IGF-1R), which is found in cancer cells. IGF-1R may help cancer cells survive and grow. By blocking IGF-1R, cancer cells may grow more slowly.

What additional tests and procedures can I expect from this additional drug while on the study?
To help monitor your health while receiving this treatment, you will have the following additional tests and procedures:

- Laboratory safety blood tests specific for ganitumab will be added to your standard of care safety blood tests

CONFIDENTIAL

- An audiology (hearing) test before you start your treatment and after you complete your paclitaxel and ganitumab treatment, but before you start your AC treatment, and for any hearing complaints.

Three additional blood samples will be taken (about 1 teaspoon) for research purposes.
- The first blood draw will be done before you start your treatment
- The second blood draw will be done in week 9 of your treatment
- The third blood draw will be done 30 days after you complete your treatment with ganitumab

The cost of these additional tests will be covered by the study.

What side effects or risks can I expect from these additional drugs while on the study?
You may have side effects while on the study. Everyone taking part in the study will be watched carefully for any side effects. However, doctors and the study sponsor don't know all the side effects that may happen and there may be unknown side effects that could occur. Side effects may be mild or very serious. Your health care team may give you medicines to help lessen the side effects. Many side effects go away soon after you stop what is causing them. In some cases, side effects can be serious or long lasting, causing hospitalization, and/or may never go away. There is also a risk of death. You may experience unexpected side effects that may not allow you to complete your standard treatment. While it is extremely unlikely, these side effects may be long-lasting and may possibly prevent you from receiving future standard or experimental therapies.

You should talk to your study doctor about any side effects you experience while taking part in the study.

You should also be aware that some investigational drugs will stop being assigned to new participants entering the trial as the trial continues. Once the study has gathered enough information about a particular investigational drug (good or bad), it will leave the trial and another new investigational drug will enter the trial. If the investigational drug leaves the study while you are still receiving it, you have three choices:

1. Continue taking the investigational drug and continue the treatment phase of the trial.
2. Stop taking the investigational drug and continue the treatment phase of the trial.
3. Stop taking the investigational drug and stop participating in the rest of the treatment phase of the trial.

Also, the investigational drug may leave the study because of severe side effects. If the investigational drug is removed for this reason while you are still receiving the drug, you will stop receiving the drug immediately. You will have the choice to continue or stop participating in the study.

The pharmaceutical company that manufactures this drug, Amgen, will be notified of all side effects and any pregnancy that you may experience while you are part of this study.

Reproductive risks:
You should not become pregnant while on this study. If you suspect that you have become pregnant at any time during the study or within three months after your last dose of chemotherapy, please notify your study doctor immediately. It is not known whether the investigational drugs can cause harm to the fetus when administered to a pregnant woman or if it affects the ability of a woman to become pregnant.

CONFIDENTIAL

Drug interactions:
Chronic use of drugs that may increase plasma glucose levels (*e.g.,* corticosteroids, furosemide) should be avoided. Currently, there are no other known prescription drugs that interact with ganitumab. However, there may be unknown drug interactions that could lead to increased side effects. Your study doctor will review all of the medications and supplements you are currently taking before starting this treatment. You should not take any new medications or supplements, including those prescribed by other doctors, without first discussing it with your study doctor or study pharmacist. Drugs that should be avoided or you need to be cautious while taking ganitumab are listed below.

Drugs to Avoid While Taking Ganitumab

Generic Drug Name	Drug Brand Name ®
Corticosteroids (chronic)	Many

Drugs to Use with Caution While Taking Ganitumab

Generic Drug Name	Drug Brand Name ®
Furosemide	Lasix*

*Caution should be exercised with initiation of Lasix while being treated with ganitumab; you will also be closely monitored for hyperglycemia while taking this drug along with ganitumab.

Human antihuman antibodies (HAHA):
Occasionally, your body may respond to this drug while it is being administered. Your body may make antibodies, called human anti-human antibody, against this drug that can be found in your blood. There is a risk that you may suffer a rash, allergy, or other unknown side effects. The symptoms of the allergic reaction are described below. It is also possible that these human anti-human antibodies may decrease the ability of this drug to treat your disease.

CONFIDENTIAL

Potential Side Effects of Investigational Treatment

Drug Treatment	Side Effects		
	**Likely** (seen in 10% or more of participants)	**Less Likely** (Seen in 1-10% of participants)	**Rare but Serious** (Seen in less than 1% of participants but might be serious)
**Paclitaxel + ganitumab**	• Fatigue<br>• Lowering number of blood platelet cells<br>• Lowering number of white blood cells (neutropenia) which could result in fever and increased risk of infection<br>• Nausea<br>• Vomiting<br>• Diarrhea<br>• Decrease in appetite<br>• High Blood Sugar (Hyperglycemia)<br>• Rash<br>• Allergic reaction symptoms:<br>-Rash<br>-Sweating<br>-Swelling or flushing of the skin<br>-Itching<br>-Change in heart rate<br>-Difficulty in breathing<br>-Low blood pressure	• Fever or chills<br>• Dry skin<br>• Acne<br>• Weight loss or gain<br>• Constipation<br>• Lowering number of red blood cells (anemia)<br>• Lowering number of white blood cells (leukopenia)<br>• Abnormal liver function as seen on a blood test<br>• Hypersensitivity, infusion site hypersensitivity<br>• Muscle or stomach pain<br>• Inflammation of mucous membranes<br>• Weakness<br>• Headache<br>• Hair loss<br>• Dizziness<br>• Dry mouth<br>• Abnormal taste<br>• Itching<br>• Increased blood pressure<br>• Swelling of the extremities, arms, legs (edema)<br>• Shortness of breath<br>• Dehydration<br>• Nosebleed<br>• Ringing in the ears (tinnitus)<br>• Indigestion	• Heart failure<br>• Kidney failure<br>• Pneumonia<br>• Low white cell count, including cells that fight infection (neutropenia), red cells and platelets (pancytopenia)<br>• Infection of the skin (cellulitis)<br>• Fever, including decreased white cells that fight infection (febrile neutropenia)<br>• Coughing up blood (Hemoptysis)<br>• Acute pancreatitis<br>• Blood clots or blockage (*e.g.*, in lungs) that in some cases may cause death. Coughing up blood<br>• Hearing loss

CONFIDENTIAL

Lactic acidosis is a rare (0.03 per 1000 participant-years) but serious toxicity associated with **metformin** use. When it occurs, it is fatal in approximately 50% of the cases. Risk of lactic acidosis is highest in participants with preexisting kidney or liver failure, severe infections, congestive heart failure, or poor oxygenation, populations that are unlikely to be treated in the I-SPY2 study. Additional risk factors are excessive alcohol consumption, and treatment with intravascular iodine-based contrast agents used in CT (x-ray) scans (not utilized within I-SPY 2 TRIAL).

CONSENT

You will be given copies of this consent form to keep along with the standard chemotherapy treatment consent form.

PARTICIPATION IN RESEARCH IS VOLUNTARY. You have the right to decline to participate or withdraw at any point in this study without jeopardy to your medical care. Leaving the study will not result in any penalty or loss of benefits to which you are entitled. The study doctor may decide to take you off this study with or without your consent if it is in your best medical interest, funding is stopped, your condition worsens, or new information becomes available.

If you wish to participate in this study, you should sign below.

Participant Signature: ____ Date: _____________

Signature of Doctor: ____ Date: _____________

Signature of Person Obtaining Consent: ________________________ Date: _____________

Signature of Translator: ______________________________________ Date: _____________

CONFIDENTIAL

I-SPY 2 TRIAL

(*Investigation of Serial Studies to Predict Your Therapeutic Response with Imaging And moLecular Analysis 2*)

SUPPLEMENT FOR MK-2206 TREATMENT

You have already been given the treatment consent form for this study. This section of the consent form will only address the side effects and risks of this specific drug you will receive with paclitaxel and the treatment schedule.

You have been randomized to the treatment group that receives MK-2206 along with Paclitaxel.

You will receive the following combination of drugs:

Drug Name	How treatment will be given	How long treatment will take each time	How often treatment is given	When treatment will be given
Paclitaxel	By vein	1 hour	Weekly	Weeks 1–12
MK-2206	Orally on an empty stomach (minimum of two hours before or after food)	A few minutes	Weekly	Weeks 1–12

This treatment regimen will last for a total of 12 weeks, after which you will receive AC as described in the treatment consent form.

On days where you receive both paclitaxel and MK-2206, you should take MK-2206 at least one hour but no more than six hours after the completion of paclitaxel.

What is MK-2206?

MK-2206 is an investigational drug, meaning it has not been approved by the FDA. MK-2206 is a small molecule that inhibits a protein known as AKT in cells. By binding to this protein, cancer cells may be killed or grow more slowly.

What additional tests and procedures can I expect from this additional drug while on the study?

To help monitor your health while receiving this treatment, you will have the following additional tests and procedures:

- Laboratory safety blood tests specific for MK-2206 will be added to your standard of care safety blood tests
- ECG (electrocardiogram, measures electrical activity of the heart) two weeks after you begin your paclitaxel and MK-2206 treatment.
    - An ECG involves attaching small wires to your arms, legs, and chest to read the electrical impulses of your heart. An ECG helps identify heart rhythm abnormalities

CONFIDENTIAL

and imbalances of salts in the body. This procedure is done in the cardiology department and takes approximately 15 to 30 minutes.

The cost of these additional tests and procedures will be covered by the study.

What side effects or risks can I expect from these additional drugs while on the study?

You may have side effects while on the study. Everyone taking part in the study will be watched carefully for any side effects. However, doctors and the study sponsor don't know all the side effects that may happen and there may be unknown side effects that could occur. Side effects may be mild or very serious. Your health care team may give you medicines to help lessen the side effects. Many side effects go away soon after you stop what is causing them. In some cases, side effects can be serious or long lasting, causing hospitalization, and/or may never go away. There is also a risk of death. You may experience unexpected side effects that may not allow you to complete your standard treatment. While it is extremely unlikely, these side effects may be long lasting and may possibly prevent you from receiving future standard or experimental therapies.

You should talk to your study doctor about any side effects you experience while taking part in the study.

You should also be aware that some investigational drugs will stop being assigned to new participants entering the trial as the trial continues. Once the study has gathered enough information about a particular investigational drug (good or bad), it will leave the trial and another new investigational drug will enter the trial. If the investigational drug leaves the study while you are still receiving it, you have 3 choices:

1. Continue taking the investigational drug and continue the treatment phase of the trial.
2. Stop taking the investigational drug and continue the treatment phase of the trial.
3. Stop taking the investigational drug and stop participating in the rest of the treatment phase of the trial.

Also, the investigational drug may leave the study because of severe side effects. If the investigational drug is removed for this reason while you are still receiving the drug, you will stop receiving the drug immediately. You will have the choice to continue or stop participating in the study.

The pharmaceutical company that manufactures this drug, Merck, will be notified of all side effects and any pregnancy that you may experience while you are part of this study.

Reproductive risks:

- You should not become pregnant while on this study. If you suspect that you have become pregnant at any time during the study or within two months after your last dose of chemotherapy, please notify your study doctor immediately. It is not known whether the investigational drugs can cause harm to the fetus when administered to a pregnant woman or if it affects the ability of a woman to become pregnant.

Drug Interactions:

- There are many prescription and over the counter drugs and dietary supplements (sometimes called complementary or alternative medicines) that may interact with MK-2206. These drug interactions could lead to increased side effects. Your study doctor will review all of the medications and supplements you are currently taking before starting

CONFIDENTIAL

this treatment. You should not take any new medications or supplements, including those prescribed by other doctors, without first discussing it with your study doctor or study pharmacist. Drugs that should be avoided while taking MK-2206 are shown below.

Drugs to Avoid while taking MK-2206

Generic Drug Name	Drug Brand Name®
Amiodarone	Cordarone®
Amiodarone	Pacerone®
Arsenic trioxide	Trisenox®
Bepridil	Vascor®
Chloroquine	Aralen®
Chlorpromazine	Thorazine®
Cisapride	Propulsid®
Clarithromycin	Biaxin®
Disopyramide	Norpace®
Dofetilide	Tikosyn®
Domperidone*	Motilium®
Droperidol	Inapsine®
Erythromycin	Erythrocin®
Erythromycin	E.E.S.®
Halofantrine	Halfan®
Haloperidol	Haldol®
Ibutilide	Corvert®
Levomethadyl	Orlaam®
Mesoridazine	Serentil®
Methadone	Dolophine®
Methadone	Methadose®
Pentamidine	Pentam®
Pentamidine	NebuPent®
Pimozide	Orap®
Procainamide	Pronestyl®
Procainamide	Procan®
Quinidine	Quinaglute®
Quinidine	Cardioquin®
Sotalol	Betapace®
Sparfloxacin	Zagam®
Thioridazine	Mellaril®

CONFIDENTIAL

**Generic Drug Name**	**Drug Brand Name®**
Cyclosporine A	Sandimmune, Gengraf, Neoral
Ketoconazole	(oral forms only)
Nelfinavir	Viracept
Ritonavir	Norvir
Saquinavir	Invirase, Fortovase
Tacrolimus	Prograf
Verapamil	Calan, Verelan
Reserpine	
Amprenavir	
Atazanavir	
Conivaptan	
Delavirdine	
Barbiturates	
Carbamazepine	
Fosphenytoin	
Nafcillin	
Fosamprenavir	
Grapefruit juice (1)	
Imatinib	
Indinavir	
Isoniazid	
Nevirapine	
Oxcarbazepine	
Phenobarbital	
Itraconazole	
Lopinavir	
Nefazodone	
Phenytoin	
Primidone	
Rifabutin	
Rifampin	
Nicardipine	
Posaconazole	
Telithromycin	

CONFIDENTIAL

Generic Drug Name	Drug Brand Name®
Rifapentine	
St. John's wort (2)	
Troglitazone	

Potential Side Effects of Investigational Treatment

Drug Treatment	Side Effects		
	**Likely** (seen in at least 10% of participants)	**Less Likely** (Seen in 3-10% of participants)	**Rare but Serious** (Seen in less than 3% of participants but might be serious)
**Paclitaxel + MK-2206**	• Rash / dry skin<br>• Itching (pruritus)<br>• Diarrhea<br>• Nausea<br>• Fatigue<br>• Decrease in appetite<br>• Elevated blood glucose (hyperglycemia)<br>• Vomiting<br>• Hair loss (alopecia)<br>• Temporary lowering of the number of white blood cells (neutropenia) which could result in fever and increased risk of infection<br>• Fever<br>• Sores in the mouth or throat (like canker sores)<br>• Inflammation in mucous tissues such as the lining of the gastrointestinal tract (intestine and stomach)<br>• Headache	• Increase in blood enzymes (alkaline phosphatase, lactate dehydrogenase)<br>• Elevated liver function tests<br>• Elevated creatine blood level (measure of kidney function)<br>• Increase in creatinine phosphokinase (CPK) which can be related to injury to muscle tissue around your heart<br>• Decreased blood levels of sodium and potassium<br>• Temporary lowering of red blood cells (anemia)<br>• Temporary lowering of blood platelet cells (thrombocytopenia)<br>• Constipation<br>• Change in taste (dysgeusia)<br>• Heartburn<br>• Common cold<br>• Weight loss<br>• Numbness or tingling in the fingers or toes which may continue after you have stopped treatment(neuropathy)<br>• Muscle spasms<br>• Dehydration<br>• Dry eyes or watery eyes<br>• Skin infection around the nails of the hand or foot<br>• Shortness of breath<br>• Inflammation of the lung (pneumonitis)<br>• Swelling of tissue in your extremities including arms, legs or mouth (edema)<br>• Increased levels of fat in the blood.	• Severe allergic reaction<br>• Inflammation of the outer layer of the eye<br>• Retinal detachment<br>• Heart rhythm abnormality (QTc prolongation)<br>• Heart beat slowing (bradycardia)<br>• Severe lung disease causing fluid, inflammation, or scarring of the lung that can cause trouble breathing.<br>• Partial limitation of eye movements<br>• Lung infection<br>• Urinary tract infection<br>• Ringing in the ears (tinnitus)<br>• Hemorrhoids<br>• Confusion / dizziness<br>• Low blood pressure<br>• Kidney damage / failure<br>• Elevated blood calcium<br>• Decreased protein in the blood<br>• Inflammation of the pancreas<br>• Generalized pain<br>• Bleeding or fluid in the lungs<br>• Blood clots<br>• Stroke<br>• Seizures

CONFIDENTIAL

If you experience multiple loose bowel movements in a day or any worsening of fatigue, nausea, vomiting, fever or rash, notify your study doctor immediately.

Unknown Risks:
Drugs similar to MK-2206 have side effects that included production of abnormal heart beats which could result in heart failure, which has symptoms of palpitations, shortness of breath and/or fatigue. Let your study doctor know if you experience any of these symptoms.

CONSENT
You will be given copies of this consent form to keep along with the standard chemotherapy treatment consent form.

PARTICIPATION IN RESEARCH IS VOLUNTARY. You have the right to decline to participate or withdraw at any point in this study without jeopardy to your medical care. Leaving the study will not result in any penalty or loss of benefits to which you are entitled. The study doctor may decide to take you off this study with or without your consent if it is in your best medical interest, funding is stopped, your condition worsens, or new information becomes available.

If you wish to participate in this study, you should sign below.

Participant Signature: ____ Date: _____________

Signature of Doctor: _______________________________________ Date: _____________

Signature of Person Obtaining Consent: ________________________ Date: _____________

Signature of Translator: ____________________________________ Date: _____________

CONFIDENTIAL

I-SPY 2 TRIAL
(*Investigation of Serial Studies to Predict Your Therapeutic Response with Imaging And moLecular Analysis 2*)

SUPPLEMENT FOR TRASTUZUMAB (HERCEPTIN) PLUS MK-2206 TREATMENT

You have already been given the treatment consent form for this study. This section of the consent form will only address the side effects and risks of this specific drug you will receive with paclitaxel and the treatment schedule.

You have been randomized to the treatment group that receives trastuzumab (Herceptin) and MK-2206 along with paclitaxel (Taxol).

You will receive the following combination of drugs:

Drug Name	How treatment will be given	How long treatment will take each time	How often treatment is given	When treatment will be given
Paclitaxel	By vein	1 hour	Weekly	Weeks 1–12
Trastuzumab	By vein	1 hour	Weekly	Weeks 1–12
MK-2206	Orally on an empty stomach (minimum of 2 hours before or after food)	A few minutes	Weekly	Weeks 1–12

This treatment regimen will last for a total of 12 weeks, after which you will receive AC as described in the treatment consent form.

You are still eligible to receive the full year of trastuzumab following your surgery. For more details, talk to your study doctor.

On days where you receive paclitaxel, trastuzumab and MK-2206, you should take MK-2206 one hour but no more than six hours after the completion of paclitaxel.
On days where you receive only trastuzumab and MK-2206, you should take MK-2206 one hour but no more than six hours after the completion of trastuzumab.

The FDA has recently granted accelerated approval for the use of pertuzumab (Perjeta) for HER2-positive breast cancer in combination with trastuzumab (Herceptin) and docetaxel (Taxotere) in the neoadjuvant (pre-surgical) setting in early breast cancer. Pertuzumab is an antibody that binds to a different part of the HER2 protein than trastuzumab.

The FDA grants accelerated approval to drugs that show early promise against a serious disease. Pertuzumab was approved as neoadjuvant treatment based on a trial of women who took pertuzumab, trastuzumab and docetaxel vs docetaxel and trastuzumab alone. More women who took the pertuzumab regimen had no sign of their cancer present in their breast or lymph nodes at the time of surgery than those women who did not take this regimen. Currently, physicians are allowed to give pertuzumab to patients receiving neoadjuvant treatment while

CONFIDENTIAL

Genentech, the company that makes pertuzumab, continues to collect more data to understand if there is a long-term benefit of pertuzumab for patients who have HER2-positive early breast cancer. Pertuzumab has also been granted full FDA approval for the treatment of HER2-positive metastatic breast cancer, based on studies in which adding pertuzumab to trastuzumab with docetaxel slowed the progression of HER2-positive metastatic disease.

The chemotherapy drugs used in pertuzumab's accelerated approval are different from the drugs we are using in I-SPY 2. We do not know if our treatment is the same, better, or worse.

It is important to discuss this with your doctor. By signing this consent, it means that you have talked with your doctor about the possible role that pertuzumab can have in your treatment.

What is Trastuzumab?

Trastuzumab, also known as Herceptin, is an approved drug by the FDA and is the standard treatment for women with Her2 positive breast cancer. Trastuzumab is a specific type of antibody that binds to Her2 proteins on cells. Antibodies are a type of protein made by cells to attack a foreign substance that the cell thinks is harmful. By interacting with Her2 proteins the cancer cells may grow slower.

What is MK-2206?

MK-2206 is an investigational drug, meaning it has not been approved by the FDA. MK-2206 is a small molecule that inhibits a protein known as AKT in cells. By binding to this protein cancer cells may be killed or grow slower.

What additional tests and procedures can I expect from this additional drug while on the study?

To help monitor your health while receiving this treatment, you will have the following additional tests and procedures:

- Laboratory safety blood tests specific for MK-2206 will be added to your standard of care safety blood tests
- ECG (electrocardiogram, measures electrical activity of the heart) two weeks after you begin your paclitaxel, trastuzumab and MK-2206 treatment.
    - An ECG involves attaching small wires to your arms, legs, and chest to read the electrical impulses of your heart. An ECG helps identify heart rhythm abnormalities and imbalances of salts in the body. This procedure is done in the cardiology department and takes approximately 15 to 30 minutes.
- ECHO or MUGA scan after you complete your paclitaxel and trastuzumab treatment but before you start your AC treatment
- ECHO or MUGA scan after you complete your AC treatment but before you have surgery

The cost of additional safety blood draws and ECGs will be covered by the study. The cost of the ECHO/MUGAs will be covered by you and/or your health plan/insurance company, as you would have both of these scans done as standard of care, even if you did not join the study

What side effects or risks can I expect from these additional drugs while on the study?

You may have side effects while on the study. Everyone taking part in the study will be watched carefully for any side effects. However, doctors and the study sponsor don't know all the side effects that may happen and there may be unknown side effects that could occur. Side effects may be mild or very serious. Your health care team may give you medicines to help lessen the

CONFIDENTIAL

side effects. Many side effects go away soon after you stop what is causing them. In some cases, side effects can be serious or long lasting, causing hospitalization, and/or may never go away. There is also a risk of death. You may experience unexpected side effects that may not allow you to complete your standard treatment.

You should talk to your study doctor about any side effects you experience while taking part in the study.

You should also be aware that some investigational drugs will stop being assigned to new participants entering the trial as the trial continues. Once the study has gathered enough information about a particular investigational drug (good or bad), it will leave the trial and another new investigational drug will enter the trial. If the investigational drug leaves the study while you are still receiving it, you have three choices:

1. Continue taking the investigational drug and continue the treatment phase of the trial.
2. Stop taking the investigational drug and continue the treatment phase of the trial.
3. Stop taking the investigational drug and stop participating in the rest of the treatment phase of the trial.

Also, the investigational drug may leave the study because of severe side effects. If the investigational drug is removed for this reason while you are still receiving the drug, you will stop receiving the drug immediately. You will have the choice to continue or stop participating in the study.

The pharmaceutical company that manufactures this drug, Merck, will be notified of all side effects and any pregnancy that you may experience while you are part of this study.

Reproductive risks:

- If you suspect that you have become pregnant at any time during the study or within 2 months after your last dose of chemotherapy, please notify your study doctor immediately. It is not known whether these drugs can cause harm to the fetus when administered to a pregnant woman or if it affects the ability of a woman to become pregnant; therefore, you should not become pregnant.

Drug Interactions:

- There are many prescription and over the counter drugs and dietary supplements (sometimes called complementary or alternative medicines) that may interact with MK-2206. These drug interactions could lead to increased side effects. Your study doctor will review all of the medications and supplements you are currently taking before starting this treatment. You should not take any new medications or supplements, including those prescribed by other doctors, without first discussing it with your study doctor or study pharmacist. Drugs that should be avoided while taking MK-2206 are shown below.

Drugs to Avoid while taking MK-2206

**Generic Drug Name**	**Drug Brand Name®**
Amiodarone	Cordarone®
Amiodarone	Pacerone®
Arsenic trioxide	Trisenox®

CONFIDENTIAL

Generic Drug Name	Drug Brand Name®
Bepridil	Vascor®
Chloroquine	Aralen®
Chlorpromazine	Thorazine®
Cisapride	Propulsid®
Clarithromycin	Biaxin®
Disopyramide	Norpace®
Dofetilide	Tikosyn®
Domperidone*	Motilium®
Droperidol	Inapsine®
Erythromycin	Erythrocin®
Erythromycin	E.E.S.®
Halofantrine	Halfan®
Haloperidol	Haldol®
Ibutilide	Corvert®
Levomethadyl	Orlaam®
Mesoridazine	Serentil®
Methadone	Dolophine®
Methadone	Methadose®
Pentamidine	Pentam®
Pentamidine	NebuPent®
Pimozide	Orap®
Procainamide	Pronestyl®
Procainamide	Procan®
Quinidine	Quinaglute®
Quinidine	Cardioquin®
Sotalol	Betapace®
Sparfloxacin	Zagam®
Thioridazine	Mellaril®
Cyclosporine A	Sandimmune, Gengraf, Neoral
Ketoconazole	(oral forms only)
Nelfinavir	Viracept
Ritonavir	Norvir
Saquinavir	Invirase, Fortovase
Tacrolimus	Prograf

CONFIDENTIAL

Generic Drug Name	Drug Brand Name®
Verapamil	Calan, Verelan
Reserpine	
Amprenavir	
Atazanavir	
Conivaptan	
Delavirdine	
Barbiturates	
Carbamazepine	
Fosphenytoin	
Nafcillin	
Fosamprenavir	
Grapefruit juice (1)	
Imatinib	
Indinavir	
Isoniazid	
Nevirapine	
Oxcarbazepine	
Phenobarbital	
Itraconazole	
Lopinavir	
Nefazodone	
Phenytoin	
Primidone	
Rifabutin	
Rifampin	
Nicardipine	
Posaconazole	
Telithromycin	
Rifapentine	
St. John's wort (2)	
Troglitazone	

CONFIDENTIAL

Potential Side Effects of Standard Treatment plus MK-2206

	Side Effects		
**Drug Treatment**	**Likely (seen in at least 10% of participants)**	**Less Likely (Seen in 3-10% of participants)**	**Rare but Serious (Seen in less than 3% of participants but might be serious)**
**Paclitaxel + Trastuzumab + MK-2206**	• Rash / dry skin<br>• Itching (pruritus)<br>• Diarrhea<br>• Nausea<br>• Fatigue<br>• Vomiting<br>• Decreased appetite<br>• Elevated blood glucose (hyperglycemia)<br>• Hair loss (alopecia)<br>• Temporary lowering of the number of white blood cells (neutropenia)<br>• Fever<br>• Chills, fever, and low blood pressure during and after Trastuzumab is given<br>• with fever<br>• Sores in the mouth or throat (like canker sores)<br>• Inflammation in mucous tissues such as the lining of the gastrointestinal tract (intestine and stomach)<br>• Headache	• Increase in blood enzymes (alkaline phosphatase, lactate dehydrogenase)<br>• Elevated liver function tests (may be an indication of liver injury or damage)<br>• Elevated creatine blood level (measure of kidney function)<br>• Increase in creatinine phosphokinase (CPK) which can be related to injury to muscle tissue around your heart<br>• Decreased blood levels of sodium and potassium<br>• Temporary lowering of red blood cells (anemia)<br>• Temporary lowering of blood platelet cells (thrombocytopenia)<br>• Constipation<br>• Change in taste (dysgeusia)<br>• Heartburn<br>• Common cold<br>• Weight loss<br>• Numbness or tingling in the fingers or toes which may continue after you have stopped treatment(neuropathy)<br>• Muscle spasms<br>• Dehydration<br>• Dry eyes or watery eyes<br>• Skin infection around the nails of the hand or foot<br>• Shortness of breath<br>• Inflammation of the lung (pneumonitis)<br>• Swelling of tissue in your extremities including arms, legs or mouth (edema)<br>• Increased levels of fat in the blood.<br>• Weakening of the heart muscle	• Severe allergic reaction<br>• Inflammation of the outer most layering of the eye<br>• Partial limitation of eye movements<br>• Retinal detachment<br>• Heart rhythm abnormality (QTc prolongation)<br>• Heart beat slowing (bradycardia)<br>• Severe lung disease causing fluid, inflammation, or scarring of the lung that can cause trouble breathing.<br>• Lung infection<br>• Urinary tract infection<br>• Ringing in the ears (tinnitus)<br>• Hemorrhoids<br>• Confusion / dizziness<br>• Low blood pressure<br>• Kidney damage / failure<br>• Elevated blood calcium<br>• Decreased protein in the blood<br>• Inflammation of the pancreas<br>• Generalized pain<br>• Bleeding in the lungs<br>• Blood clots<br>• Stroke<br>• Seizures<br>• heart failure

If you experience multiple loose bowel movements in a day or any worsening of fatigue, nausea, vomiting, fever or rash, notify your study doctor immediately.

CONFIDENTIAL

Unknown Risks:
Drugs similar to MK-2206 have side effects that included production of abnormal heart beats which could result in heart failure, which has symptoms of palpitations, shortness of breath and/or fatigue. Let your study doctor know if you experience any of these symptoms.

CONSENT
You will be given copies of this consent form to keep along with the standard chemotherapy treatment consent form.

PARTICIPATION IN RESEARCH IS VOLUNTARY. You have the right to decline to participate or withdraw at any point in this study without jeopardy to your medical care. Leaving the study will not result in any penalty or loss of benefits to which you are entitled. The study doctor may decide to take you off this study with or without your consent if it is in your best medical interest, funding is stopped, your condition worsens, or new information becomes available.

If you wish to participate in this study, you should sign below.

Participant Signature: ____ Date: _____________

Signature of Doctor: _____________________________________ Date: _____________

Signature of Person Obtaining Consent: ________________________ Date: _____________

Signature of Translator: _____________________________________ Date: _____________

CONFIDENTIAL

<u>I-SPY 2 TRIAL</u>

*(**I**nvestigation of **S**erial Studies to **P**redict **Y**our **T**herapeutic **R**esponse with **I**maging **A**nd mo**L**ecular Analysis **2**)*

SUPPLEMENT FOR PERTUZUMAB AND TRASTUZUMAB TREATMENT

You have already been given the treatment consent form for this study. This section of the consent form will only address the side effects and risks of this specific drug you will receive with paclitaxel and trastuzumab and the treatment schedule.

***You have been randomized to the treatment group that receives Pertuzumab and Trastuzumab along with Paclitaxel.* You will receive the following combination of drugs:**

Drug Name	How treatment will be given	How long treatment will take each time	How often treatment is given	When treatment will be given
Paclitaxel	By vein	1 hour	Weekly	Weeks 1–12
Trastuzumab	By vein	1 hour	Weekly	Weeks 1–12
Pertuzumab	By vein	1 hour	Every 3 weeks	Weeks 1,4,7,10

This treatment regimen will last for a total of 12 weeks, after which you will receive AC as described in the treatment consent form.

What is Trastuzumab?

Trastuzumab, also known as Herceptin, is an approved drug by the FDA and is the standard treatment for women with HER2+ breast cancer. Trastuzumab is a specific type of antibody that binds to HER2 proteins on cells. Antibodies are a type of protein made by cells to attack a foreign substance that the cell thinks is harmful. By interacting with HER2 proteins, the cancer cells may grow more slowly.

What is Pertuzumab?

Pertuzumab is also a type of antibody that binds to HER2 proteins in breast cancer. However, Pertuzumab binds to a different part of the HER2 molecule than trastuzumab and consequently the combination of the two antibodies has been shown to have increased activity in controlling cancer cell growth. Pertuzumab has been tested in large numbers of women with breast and other types of cancer, and has recently been FDA approved.

What additional tests and procedures can I expect from this additional drug while on the study?

To help monitor your health while receiving this treatment, you will have the following additional tests and procedures:

- ECHO or MUGA scan after you complete your paclitaxel and pertuzumab plus trastuzumab treatment but before you start your AC treatment
- ECHO or MUGA scan after you complete your AC treatment but before you have surgery

CONFIDENTIAL

You would have both of these scans done as standard of care, even if you did not join the study. The cost of these scans will be covered by you and/or your health plan/insurance company.

What side effects or risks can I expect from these additional drugs while on the study?

You may have side effects while on the study. Everyone taking part in the study will be watched carefully for any side effects. However, doctors and the study sponsor don't know all the side effects that may happen and there may be unknown side effects that could occur. Side effects may be mild or very serious. Your health care team may give you medicines to help lessen the side effects. Many side effects go away soon after you stop what is causing them. In some cases, side effects can be serious or long-lasting, causing hospitalization, and/or may never go away. There is also a risk of death. You may experience unexpected side effects that may not allow you to complete your standard treatment. While it is extremely unlikely, these side effects may be long-lasting and may possibly prevent you from receiving future standard or experimental therapies.

You should talk to your study doctor about any side effects you experience while taking part in the study.

You should also be aware that some investigational agents will stop being assigned to new participants entering the trial as the trial continues. Once the study has gathered enough information about a particular investigational agent (good or bad), it will leave the trial and another new investigational agent will enter the trial. If the investigational agent leaves the study while you are still receiving it, you have three choices:

1. Continue taking the investigational agent and continue the treatment phase of the trial.
2. Stop taking the investigational agent and continue the treatment phase of the trial.
3. Stop taking the investigational agent and stop participating in the rest of the treatment phase of the trial.

Also, the investigational agent may leave the study because of severe side effects. If the investigational agent is removed for this reason while you are still receiving the agent, you will stop receiving the agent immediately. You will have the choice to continue or stop participating in the study.

The pharmaceutical company that manufactures this agent, Genentech, will be notified of all side effects and any pregnancy that you may experience while you are part of this study.

Reproductive risks:

- If you suspect that you have become pregnant at any time during the study or within two months after your last dose of chemotherapy, please notify your study doctor immediately. It is not known whether these drugs can cause harm to the fetus when administered to a pregnant woman or if it affects the ability of a woman to become pregnant; therefore, you should not become pregnant.

CONFIDENTIAL

Potential Side Effects of Standard Treatment plus Pertuzumab

	Side Effects		
**Drug Treatment**	**Likely**<br>**(seen in more than 20% of participants)**	**Less Likely**<br>**(Seen in 20% or less of participants)**	**Rare but Serious**<br>**(Seen in 2–3% or less of participants but might be serious)**
**Paclitaxel + Pertuzumab+ Trastuzumab**	• Diarrhea<br>• Nausea<br>• Vomiting<br>• Fatigue<br>• Decreased appetite<br>• Hair loss (alopecia)<br>• Chills, fever, and low blood pressure during and after Trastuzumab or Pertuzumab is given<br>• Temporary lowering of the number of white blood cells (neutropenia) without or with fever (febrile neutropenia)<br>• Temporary lowering of red blood cells (anemia)<br>• Temporary lowering of blood platelet cells (thrombocytopenia)<br>• Numbness or tingling in the fingers or the toes which may continue after you have stopped treatment (neuropathy)<br>• Temporary pain in the muscles, and joints<br>• Fluid in arms and legs<br>• Abdominal pain, or stomach upset<br>• Shortness of breath or cough<br>• Nail inflammation<br>• Mucosal inflammation (inflammation of the lining of the mouth, digestive system, or genital tract)<br>• Abnormal sense of taste<br>• Dizziness<br>• Cold or chest infection<br>• Rash<br>• Dry skin<br>• Itching	• Fluid in the lungs and inflammation of the lungs, which can lead to difficulty or the inability to breath<br>• Pneumonia<br>• Allergic reactions including hives<br>• Fluid around the heart or inflammation of the heart<br>• Infections where the nail and skin meet at the side or the base of a finger or toenail (paronychia)<br>• Sores in the mouth or throat (like canker sores)<br>• Abnormal liver function as seen on a blood test<br>• Flu-like symptoms: headache muscle ache bone ache loss of appetite<br>• Voice changes<br>• Constipation<br>• Mental disturbance (paranoia)<br>• Insomnia<br>• Nose bleeds<br>• Urinary tract infection<br>• High blood pressure<br>• Increased watery eyes	• Severe allergic reaction<br>• Severe lung disease causing great difficulty breathing<br>• Low blood pressure<br>• Liver failure<br>• Small intestine blockage<br>• Weakening of the heart muscle, which could result in heart failure<br>• Blood clot in the veins of the arms, legs, or lungs

CONFIDENTIAL

You will be given copies of this consent form to keep along with the standard chemotherapy treatment consent form.

PARTICIPATION IN RESEARCH IS VOLUNTARY. You have the right to decline to participate or withdraw at any point in this study without jeopardy to your medical care. Leaving the study will not result in any penalty or loss of benefits to which you are entitled. The study doctor may decide to take you off this study with or without your consent if it is in your best medical interest, funding is stopped, your condition worsens, or new information becomes available.

If you wish to participate in this study, you should sign below.

Participant Signature: ____ Date: _____________

Signature of Doctor: ______________________________________ Date: _____________

Signature of Person Obtaining Consent: _________________________ Date: _____________

Signature of Translator: _____________________________________ Date: _____________

CONFIDENTIAL

I-SPY 2 TRIAL
(*Investigation of Serial Studies to Predict Your Therapeutic Response with Imaging And moLecular Analysis 2*)

SUPPLEMENT FOR T-DM1 (TRASTUZUMAB-EMTANSINE) AND PERTUZUMAB TREATMENT

You have already been given the treatment consent form for this study. This section of the consent form will only address the side effects and risks of this specific drug you will receive and the treatment schedule.

You have been randomized to the treatment group that receives T-DM1 and Pertuzumab.

You will receive the following combination of drugs:

Drug Name	How treatment will be given	How long treatment will take each time	How often treatment is given	When treatment will be given
T-DM1	By vein	1 hour	Every 3 weeks	Weeks 1,4,7,10
Pertuzumab	By vein	1 hour	Every 3 weeks	Weeks 1,4,7,10

This treatment regimen will last for a total of 12 weeks, after which you will receive AC as described in the treatment consent form.

You are still eligible to receive paclitaxel and/or a full year of trastuzumab following your surgery. For more details, talk to your study doctor.

What is T-DM1 (Trastuzumab-emtansine)?

T-DM1(Trastuzumab-emtansine) is an investigational drug meaning that is has not yet been approved by the FDA. Trastuzumab, also known as Herceptin, is a specific type of antibody that binds to HER2 proteins on cells. Antibodies are a type of protein made by cells to attack a foreign substance that the cell thinks is harmful. T-DM1 has the same antibody as Trastuzumab but coupled to a cytotoxic compound, emtansine. By interacting with HER2 proteins, T-DM1 may make the cancer cells grow more slowly or kill them directly.

What is Pertuzumab?

Pertuzumab is a type of antibody that binds to HER2 proteins in breast cancer. However, Pertuzumab binds to a different part of the HER2 molecule than Trastuzumab and consequently the combination of the two antibodies has been shown to have an increased activity in controlling cancer cell growth. Pertuzumab has been tested in large numbers of women with breast and other types of cancer and has recently been FDA approved.

What additional tests and procedures can I expect from this additional drug while on the study?

To help monitor your health while receiving this treatment, you will have the following additional tests and procedures:

CONFIDENTIAL

- ECHO or MUGA scan after you complete your T-DM1 and pertuzumab treatment but before you start your AC treatment
- ECHO or MUGA scan after you complete your AC treatment but before you have surgery

The cost of these additional tests will be covered by the study.

What side effects or risks can I expect from these additional drugs while on the study?

You may have side effects while on the study. Everyone taking part in the study will be watched carefully for any side effects. However, doctors and the study sponsor don't know all the side effects that may happen and there may be unknown side effects that could occur. Side effects may be mild or very serious. Your health care team may give you medicines to help lessen the side effects. Many side effects go away soon after you stop what is causing them. In some cases, side effects can be serious or long lasting, causing hospitalization, and/or may never go away. There is also a risk of death. You may experience unexpected side effects that may not allow you to complete your standard treatment. While it is extremely unlikely, these side effects may be long-lasting and may possibly prevent you from receiving future standard or experimental therapies.

You should talk to your study doctor about any side effects you experience while taking part in the study.

You should also be aware that some investigational agents will stop being assigned to new participants entering the trial as the trial continues. Once the study has gathered enough information about a particular investigational agent (good or bad), it will leave the trial and another new investigational agent will enter the trial. If the investigational agent leaves the study while you are still receiving it, you have three choices:

1. Continue taking the investigational agent and continue the treatment phase of the trial.
2. Stop taking the investigational agent and continue the treatment phase of the trial.
3. Stop taking the investigational agent and stop participating in the rest of the treatment phase of the trial.

Also, the investigational agent may leave the study because of severe side effects. If the investigational agent is removed for this reason while you are still receiving the agent, you will stop receiving the agent immediately. You will have the choice to continue or stop participating in the study.

The pharmaceutical company that manufactures this agent, Genentech, will be notified of all side effects and any pregnancy that you may experience while you are part of this study.

Reproductive risks:

- If you suspect that you have become pregnant at any time during the study or within 2 months after your last dose of chemotherapy, please notify your study doctor immediately. It is not known whether these drugs can cause harm to the fetus when administered to a pregnant woman or if it affects the ability of a woman to become pregnant; therefore, you should not become pregnant.

CONFIDENTIAL

Potential Side Effects of T-DM1 plus Pertuzumab

	Side Effects		
**Drug Treatment**	**<u>Likely</u> (seen in more than 10% of participants)**	**<u>Less Likely</u> (Seen in 10% or less of participants)**	**<u>Rare but Serious</u> (Seen 1% or less of participants but might be serious)**
**T-DM1+ Pertuzumab**	• Fatigue<br>• Nausea<br>• Headache<br>• Vomiting<br>• Constipation<br>• Nose bleeds<br>• Diarrhea<br>• Fever<br>• Decreased appetite<br>• Abnormal liver function as seen on a blood test<br>• Temporary lowering of the number of platelets (help with blood clotting)<br>• Low number of red blood cells and hemoglobin (may make you feel tired)<br>• Skin rash<br>• Difficulty breathing while resting (dyspnea)<br>• Difficulty sleeping (insomnia)<br>• Low sodium<br>• Lack or loss of strength<br>• Chills<br>• Cough<br>• Abdominal pain, or stomach upset<br>• Dry mouth<br>• Sores in mouth<br>• Temporary increases in liver enzyme<br>• Numbness or tingling in the fingers or the toes which may continue after you have stopped treatment (neuropathy)<br>• Abnormal laboratory blood levels including:<br>- low potassium<br>• Pain in joints and muscles<br>• Urinary tract infection<br>• Abnormal sense of taste (dysguesia)	• Temporary lowering of the number of white blood cells (neutropenia) without or with fever (febrile neutropenia)<br>• Fluid in arms and legs<br>• Temporary pain in the back, muscles, and/or joints<br>• Chills, fever, and low blood pressure during and after T-DM1or Pertuzumab is given<br>• High blood pressure<br>• Difficulty urinating<br>• Weakening of the heart muscle, which could result in heart failure<br>• Fluid in the lungs and inflammation of the lungs, which can lead to difficulty or the inability to breath<br>• Pneumonia<br>• Shortness of breath or cough<br>• Allergic reactions including hives<br>• Fluid around the heart or inflammation of the heart<br>• Dry skin<br>• Itching<br>• Eye disorders (dry eye, increases tears, blurred vision, pink eye)<br>• Severe hemorrhage (bleeding)<br>• Dizziness<br>• Altered taste sensation<br>• Upset stomach or indigestion<br>• High levels of alkaline phosphatase in blood<br>• Mental disturbance (paranoia)<br>• Confusion<br>• Nail inflammation	• Severe allergic reaction<br>• Severe lung disease causing great difficulty breathing<br>• Irregular heart beat- this sometimes leads to fainting or heart stopping<br>• Low blood pressure<br>• Liver or kidney failure<br>• Small intestine blockage<br>• Skin infection (cellulitis)<br>• Pneumonitis(inflammation of lungs)<br>• Severe liver dysfunction or failure, including localized high blood pressure in liver and development of benign nodules<br>• Blood clot in the veins of the arms, legs or lungs.

CONFIDENTIAL

CONSENT
You will be given copies of this consent form to keep along with the standard chemotherapy treatment consent form.

PARTICIPATION IN RESEARCH IS VOLUNTARY. You have the right to decline to participate or withdraw at any point in this study without jeopardy to your medical care. Leaving the study will not result in any penalty or loss of benefits to which you are entitled. The study doctor may decide to take you off this study with or without your consent if it is in your best medical interest, funding is stopped, your condition worsens, or new information becomes available.

If you wish to participate in this study, you should sign below.

Participant Signature: ____ Date: _____________

Signature of Doctor: ______________________________________ Date: _____________

Signature of Person Obtaining Consent: _________________________ Date: _____________

Signature of Translator: _____________________________________ Date: _____________

CONFIDENTIAL

I-SPY 2 TRIAL
(*Investigation of Serial Studies to Predict Your Therapeutic Response with Imaging And moLecular Analysis 2*)

SUPPLEMENT FOR GANETESPIB TREATMENT

You have already been given the treatment consent form for this study. This section of the consent form will only address the side effects and risks of this specific drug you will receive with paclitaxel and the treatment schedule.

You have been randomized to the treatment group that receives Ganetespib along with Paclitaxel.

You will receive the following combination of drugs:

Drug Name	How treatment will be given	How long treatment will take each time	How often treatment is given	When treatment will be given
Paclitaxel	By vein	1 hour	Weekly	Weeks 1–12
Ganetespib	By vein	1 hour	Weekly	Weeks 1-3, 5-7,9-11

This treatment regimen will last for a total of 12 weeks, after which you will receive AC as described in the treatment consent form.

CONFIDENTIAL

What is Ganetespib?

Ganetespib is an investigational drug, meaning it has not been approved by the FDA. Ganetespib is a small molecule that inhibits a heat shock protein known as HSP90 in cells. By binding to this protein, cancer cells may be killed or grow more slowly.

What additional tests and procedures can I expect from this additional drug while on the study?

To help monitor your health while receiving this treatment, you will have the following additional tests and procedures:

- ECG (electrocardiogram, measures electrical activity of the heart) will be done:
  - Before each treatment with Ganetespib.
  - 24 hours after your first treatment with Ganetespib

    - An ECG involves attaching small wires to your arms, legs, and chest to read the electrical impulses of your heart. An ECG helps identify heart rhythm abnormalities and imbalances of salts in the body. This test will takeapproximately 5-10 minutes.

- Baseline Visual Assessment before first treatment with Ganetespib
  - The study physician will ask questions about your eye health which may include any history of eye problems or eye injuries, symptoms such as discharge, light sensitivity and double-vision, as well as family

history of glaucoma, and diseases such as diabetes which can affect eye health
    - Your visual acuity will be assessed by asking you to read letters on an eye chart.
    - Additional tests may be required based on your eye health history or at your doctors discretion.

The cost of these additional tests and procedures will be covered by the study.

What side effects or risks can I expect from these additional drugs while on the study?

You may have side effects while on the study. Everyone taking part in the study will be watched carefully for any side effects. However, doctors and the study sponsor don't know all the side effects that may happen and there may be unknown side effects that could occur. Side effects may be mild or very serious. Your health care team may give you medicines to help lessen the side effects. Many side effects go away soon after you stop what is causing them. In some cases, side effects can be serious or long lasting, causing hospitalization, and/or may never go away. There is also a risk of death. You may experience unexpected side effects that may not allow you to complete your standard treatment. While it is extremely unlikely, these side effects may be long lasting and may possibly prevent you from receiving future standard or experimental therapies.

You should talk to your study doctor about any side effects you experience while taking part in the study.

You should also be aware that some investigational drugs will stop being assigned to new participants entering the trial as the trial continues. Once the study has gathered enough information about a particular investigational drug (good or bad), it will leave the trial and another new investigational drug will enter the trial. If the investigational drug leaves the study while you are still receiving it, you have 3 choices:

4. Continue taking the investigational drug and continue the treatment phase of the trial.
5. Stop taking the investigational drug and continue the treatment phase of the trial.
6. Stop taking the investigational drug and stop participating in the rest of the treatment phase of the trial.

Also, the investigational drug may leave the study because of severe side effects. If the investigational drug is removed for this reason while you are still receiving the drug, you will stop receiving the drug immediately. You will have the choice to continue or stop participating in the study.

The pharmaceutical company that manufactures this drug, Synta, will be notified of all side effects and any pregnancy that you may experience while you are part of this study.

ECG:

Possible side effects or complications from ECG include rash and itching in the places of electrode/gel placement.

Reproductive risks:

- You should not become pregnant while on this study. If you suspect that you have become pregnant at any time during the study or within two months after your last dose of chemotherapy, please notify your study doctor immediately. It is not known whether the

CONFIDENTIAL

investigational drugs can cause harm to the fetus when administered to a pregnant woman or if it affects the ability of a woman to become pregnant.
- You must continue to use birth control for 30 days after the last dose of study drug.
- In an animal study, pregnant rats given ganetespib daily had side effects such as decreased weight gain and/or weight loss, and an increase in embryonic deaths. In another animal study, rats given a high ganetespib dose daily for 2 days had increased micronuclei in bone marrow polychromatic erythrocytes, which is an indicator of genotoxicity.

Drug Interactions:

- There are many prescription and over the counter drugs and dietary supplements (sometimes called complementary or alternative medicines) that may interact with Ganetespib. These drug interactions could lead to increased side effects. There are also groups of medications, which have to be used with caution or avoided**:** certain types of tranquilizers, blood pressure medications, antibiotics, antiviral medications, and gastric acid medications. Your study doctor will review all of the medications and supplements you are currently taking before starting this treatment.-You should not take any new medications or supplements, including those prescribed by other doctors, without first discussing it with your study doctor or study pharmacist.

Please refer to the standard of care consent form for potential side effects of paclitaxel.

Potential Side Effects of Ganetespib Treatment

**Drug Treatment**	**Side Effects**		
	**Likely (seen in 10% or more of participants)**	**Less Likely (Seen in 1-10% of participants)**	**Rare but Serious (Seen in less than 1% of participants but might be serious)**
**Ganetespib**	• Diarrhea, feeling sick to the stomach (nausea), or throwing up (vomiting). You will receive treatment to help prevent the onset of diarrhea.<br>• Feeling tired (fatigue)<br>• Weakness<br>• Cough<br>• Not feeling hungry (decreased appetite)<br>• Allergic reaction or hypersensitivity reaction to the and/or at the site of infusion that may include a rash, hives, fever, difficulty breathing, chest tightness, low blood pressure or increased blood pressure and heart	• Decrease in the number of of white blood cells that may lead to infection and require treatment with antibiotics. (leukopenia)<br>• Temporary lowering of blood platelet cells (thrombocytopenia)<br>• Blurred vision or other visual impairment (e.g., ability to adapt to changes in brightness,)<br>• Heartburn, abdominal pain or discomfort, swelling, indigestion (dyspepsia)<br>• Hemorrhoids<br>• Dry Mouth<br>• Mouth sores and irritation Dehydration<br>• Changes in blood sugar levels<br>• Feeling dizzy (dizziness)	• abnormal heart rhythm<br>• the heart overall stopping<br>• hole in the stomach, intestine, or colon<br>• Feeling confused, memory loss<br>• Kidney failure<br>• Failure to thrive<br>• Fainting<br>• Pneumonitis<br>• Hepatotoxicity<br>• Increase of a protein called troponin, that could mean damage to the heart muscle<br>• Chest pain<br>• Acute Pancreatitis<br>• Coughing up blood (Hemoptysis)<br>• Flu-like symptoms<br>• Change in blood pressure<br>• Excessive bleeding or delayed clotting

CONFIDENTIAL

Drug Treatment	Side Effects		
	**<u>Likely</u>**<br>**(seen in 10% or more of participants)**	**<u>Less Likely</u>**<br>**(Seen in 1-10% of participants)**	**<u>Rare but Serious</u>**<br>**(Seen in less than 1% of participants but might be serious)**
	rate. Although usually reversible with treatment, it can be severe or life threatening. (infusion related reaction)<br>• Rash, which may be dry and itchy<br>• Decrease in the number of neutrophils- white blood cells that fight infection sometimes with fever and/or flu-like symptoms<br>• Decrease in red blood cells (the cells that carry oxygen through the body) (anemia)<br>• Constipation<br>• Difficulty sleeping (insomnia)<br>• Difficulty breathing (dyspnea)<br>• Dehydration<br>• Weight loss<br>• Abnormal laboratory blood levels including:<br>- low potassium<br>- low sodium<br>- low phosphate<br>- low magnesium<br>• Abnormal liver or kidney functions as seen on a blood test	• Pain, spasms, numbness or weakness in muscles or joints<br>• Tingling or pricking sensation on the skin (paraesthesia)<br>• Headache<br>• Loss of taste (dygeusia)<br>• Brittle or discolored nails<br>• Fever<br>• Peripheral edema	• Depression, agitation, and or other change in mental health<br>• Slowing of the electrical activity of the heart on EKG (QTC interval prolongation)

CONFIDENTIAL

If you experience multiple loose bowel movements in a day or any worsening of fatigue, nausea, vomiting, fever or rash, notify your study doctor immediately.

Results from a recently completed study in healthy volunteers
1-hour intravenous infusion dose of 200 mg/m^2 ganetespib may cause the heart muscle to take longer than usual to make another beat. This is called QT prolongation, and is determined by an electrocardiogram (ECG). This prolongation could predispose patients to develop symptoms such as fainting (syncope) or an arrhythmia (a disturbance in your heart rate) which can potentially be fatal.

While taking part in this study, your doctor may request additional electrocardiograms (ECG, EKG) to monitor your heart rate and rhythm.

IN CASE OF INJURY:

Please refer to the standard of care consent form that you have received.

MEDICAL INFORMATION

Synta Pharmaceuticals may need to look at your medical records for research quality assurance and data analysis.

CONSENT

You will be given copies of this consent form to keep along with the standard chemotherapy treatment consent form.

PARTICIPATION IN RESEARCH IS VOLUNTARY. You have the right to decline to participate or withdraw at any point in this study without jeopardy to your medical care. Leaving the study will not result in any penalty or loss of benefits to which you are entitled. The study doctor may decide to take you off this study with or without your consent if it is in your best medical interest, funding is stopped, your condition worsens, or new information becomes available.

If you wish to participate in this study, you should sign below.

Participant Signature: _______________________________________ Date: _____________

Signature of Doctor: ____ Date: _____________

Signature of Person Obtaining Consent: ________________________ Date: _____________

Signature of Translator: _____________________________________ Date: _____________

CONFIDENTIAL

I-SPY 2 TRIAL

*(**I**nvestigation of **S**erial Studies to **P**redict **Y**our **T**herapeutic **R**esponse with **I**maging **A**nd mo**L**ecular Analysis **2**)*

SUPPLEMENT FOR PLX3397 TREATMENT

You have already been given the treatment consent form for this study. This section of the consent form will only address the side effects and risks of this specific drug you will receive with paclitaxel and the treatment schedule.

You have been randomized to the treatment group that receives PLX3397 along with Paclitaxel.

You will receive the following combination of drugs:

Drug Name	How treatment will be given	How long treatment will take each time	How often treatment is given	When treatment will be given
Paclitaxel	By vein	1 hour	Weekly	Weeks 1–12
PLX3397	Orally with 8oz of water NOTE: Do not eat anything one hour before and after taking PLX3397	A few minutes	Twice a day approximately 12 hours apart plus or minus 2 hours	Weeks 1–12

This treatment regimen will last for a total of 12 weeks after which you will receive AC as described in the treatment consent form.

If you vomit your dose, do NOT make up that dose.

What is PLX3397?

PLX3397 is an investigational drug, meaning it has not been approved by the FDA. PLX3397 targets the activity of certain immune cells, called macrophages, that help cancer cells enter other parts of the body. PLX3397 targets the macrophages to prevent cancer tumors from spreading to other parts of the body.

What additional tests and procedures can I expect from this additional drug while on the study?

There are no additional tests or procedures

What side effects or risks can I expect from these additional drugs while on the study?

You may have side effects while on the study. Everyone taking part in the study will be watched carefully for any side effects. However, doctors and the study sponsor don’t know all the side

CONFIDENTIAL

effects that may happen and there may be unknown side effects that could occur. Side effects may be mild or very serious. Your health care team may give you medicines to help lessen the side effects. Many side effects go away soon after you stop what is causing them. In some cases, side effects can be serious or long lasting, causing hospitalization, and/or may never go away. There is also a risk of death. You may experience unexpected side effects that may not allow you to complete your standard treatment. While it is extremely unlikely, these side effects may be long lasting and may possibly prevent you from receiving future standard or experimental therapies.

You should talk to your study doctor about any side effects you experience while taking part in the study.

You should also be aware that some investigational drugs will stop being assigned to new patients entering the trial as the trial continues. Once the study has gathered enough information about a particular investigational drug (good or bad), it will leave the trial and another new investigational drug will enter the trial. If the investigational drug leaves the study while you are still receiving it, you have 3 choices:

4. Continue taking the investigational drug and continue the treatment phase of the trial.
5. Stop taking the investigational drug and continue the treatment phase of the trial.
6. Stop taking the investigational drug and stop participating in the rest of the treatment phase of the trial.

Also, the investigational drug may leave the study because of severe side effects. If the investigational drug is removed for this reason while you are still receiving the drug, you will stop receiving the drug immediately. You will have the choice to continue or stop participating in the study.

The pharmaceutical company that manufactures this drug, Plexxikon, will be notified of all side effects and any pregnancy that you may experience while you are part of this study.

Reproductive risks:

- You should not become pregnant while on this study. If you suspect that you have become pregnant at any time during the study or within 2 months after your last dose of chemotherapy, please notify your study doctor immediately. It is not known whether the investigational drugs can cause harm to the fetus when administered to a pregnant woman or if it affects the ability of a woman to become pregnant.

Drug Interactions:

- There are many prescription and over the counter drugs and dietary supplements (sometimes called complementary or alternative medicines) that may interact with PLX3397. These drug interactions could lead to increased side effects. Your study doctor will review all of the medications and supplements you are currently taking before starting this treatment. You should not take any new medications or supplements, including those prescribed by other doctors, without first discussing it with your study doctor or study pharmacist. Drugs that should be avoided while taking PLX3397 are shown below.

CONFIDENTIAL

Drugs to Avoid while taking PLX3397

CONFIDENTIAL

**Generic Drug Name**	**Drug Brand Name ®**
Amiodarone	Cordarone®
Amiodarone	Pacerone®
Arsenic trioxide	Trisenox®
Bepridil	Vascor®
Chloroquine	Aralen®
Chlorpromazine	Thorazine®
Cisapride	Propulsid®
Clarithromycin	Biaxin®
Disopyramide	Norpace®
Dofetilide	Tikosyn®
Domperidone*	Motilium®
Droperidol	Inapsine®
Erythromycin	Erythrocin®
Erythromycin	E.E.S.®
Halofantrine	Halfan®
Haloperidol	Haldol®
Ibutilide	Corvert®
Levomethadyl	Orlaam®
Mesoridazine	Serentil®
Methadone	Dolophine®
Methadone	Methadose®
Pentamidine	Pentam®
Pentamidine	NebuPent®
Pimozide	Orap®
Procainamide	Pronestyl®
Procainamide	Procan®
Quinidine	Quinaglute®
Quinidine	Cardioquin®
Sotalol	Betapace®
Sparfloxacin	Zagam®
Thioridazine	Mellaril®
Cyclosporine A	Sandimmune, Gengraf, Neoral

CONFIDENTIAL

Ketoconazole	(oral forms only)
Nelfinavir	Viracept
Ritonavir	Norvir
Saquinavir	Invirase, Fortovase
Tacrolimus	Prograf
Verapamil	Calan, Verelan
Reserpine	
Amprenavir	
Atazanavir	
Conivaptan	
Delavirdine	
Barbiturates	
Carbamazepine	
Fosphenytoin	
Nafcillin	
Fosamprenavir	
Grapefruit juice (1)	
Imatinib	
Indinavir	
Isoniazid	
Nevirapine	
Oxcarbazepine	
Phenobarbital	
Itraconazole	
Lopinavir	
Nefazodone	
Phenytoin	
Primidone	
Rifabutin	
Rifampin	
Nicardipine	
Posaconazole	
Telithromycin	

CONFIDENTIAL

Rifapentine	
St. John's wort (2)	
Troglitazone	

Potential Side Effects of Investigational Treatment

	Side Effects		
**Drug Treatment**	**<u>Likely</u> (seen in 10% or more of patients)**	**<u>Less Likely</u> (Seen in less than 10% of patients)**	**<u>Rare but Serious</u> (Seen in 2–3% or less of patients but might be serious)**
**Paclitaxel + PLX3397**	• Diarrhea<br>• Nausea<br>• Fatigue<br>• Decrease in appetite<br>• Vomiting<br>• Hair loss (alopecia)<br>• Hair color changes (to white or grey; original color may return after stopping study drug)<br>• Decreased white blood cell count (which may lead to increased risk of infection)<br>• Temporary lowering of red blood cells (anemia)<br>• Temporary lowering of blood platelet cells (thrombocytopenia)<br>• Increase in blood enzymes that monitor for and indicate liver damage (transaminases and/or alkaline phosphatase)<br>• Abnormal sense of taste<br>• Tingling and numbness in hands and feet<br>• Bone and muscle aches<br>• Irritated mucous membrane in your mouth	• Edema (swelling of tissues in limbs, face, or around the eyes)<br>• Fever and/or chills<br>• Constipation<br>• Rash<br>• Weight loss<br>• Pruritus (itching of skin)<br>• Dehydration<br>• Headaches<br>• Dizziness<br>• Hypertension (high blood pressure)<br>• Insomnia (in ability to fall asleep or stay asleep)<br>• Allergic reaction<br>• Brittle or yellowed finger/toe nails<br>• Abdominal pain<br>• Cough<br>• Low levels of electrolytes in blood (which could include one or more of the following: potassium, phosphorus, calcium, or sodium)<br>• Mucosal inflammation (inflammation affecting moist tissue lining parts of the inside of your body, such as mouth, nose, lungs, and digestive tract)	• Severe bone marrow suppression with decreased white blood cell count, anemia or reduced platelets<br>• Elevations of blood liver tests indicating potentially severe liver injury<br>• Severe lung disease that can cause trouble breathing<br>• Damage to heart muscle that can lead to heart failure

There is a report of a potential interaction between PLX3397 and warfarin (Coumadin, an oral drug used to thin the blood). Lab results for patients on study taking warfarin have showed an increase in PT/INR, which is a test of blood clotting. If you are taking warfarin, we will monitor

CONFIDENTIAL

your blood carefully to make sure that your blood thinning is well controlled. If necessary, adjustments to your dose of warfarin will be made.

Taking PLX3397 together with other medications that can cause liver function abnormalities may further increase the risk of severe liver injury.

Animals treated with PLX3397 showed decrease in the pumping action (contraction) of the heart. There have been two reports of reduced ejection fraction (the amount of blood pumped by your heart with each beat) in patients receiving PLX3397 at a dose of 3000 mg/day, a much higher dose than you will receive. The role of PLX3397 in these events is undetermined at this time.

In laboratory animal studies, the following side effects were associated with continuous dosing of PLX3397, were believed to be caused by the high doses studied in the animals (well above the doses being tested in this study), and were all reversible upon drug discontinuation:

- Increased bone density in the legs
- Decreased red blood cells in the blood and bone marrow
- Slightly decreased white blood cell counts in the blood and bone marrow (this might be expected to cause increased susceptibility to infection)
- Changes in the appearance of skin and kidney when looked at under the microscope
- Increased size of liver and thyroid gland

These side effects may or may not appear in human subjects, and were not observed with high, single dose administration in animals.

The severity of the side effects listed above could range from mild to severe or even life-threatening.

Unknown Risks:
There may be other risks or side effects from PLX3397 that are not listed above or unknown at this time. Drugs similar to PLX3397 have side effects that included production of abnormal heart beats which could result in heart failure, which has symptoms of palpitations, shortness of breath and/or fatigue. It is also possible to experience a serious allergic reaction, which could become life-threatening or fatal. Symptoms of an allergic reaction include rash, hives, itching, swelling of the mouth, face, lips or tongue, dizziness, tightness in the chest, or trouble breathing. Let your study doctor know if you experience any of these symptoms.

CONSENT
You will be given copies of this consent form to keep along with the standard chemotherapy treatment consent form.

PARTICIPATION IN RESEARCH IS VOLUNTARY. You have the right to decline to participate or withdraw at any point in this study without jeopardy to your medical care. Leaving the study will not result in any penalty or loss of benefits to which you are entitled. The study doctor may decide to take you off this study with or without your consent if it is in your best medical interest, funding is stopped, your condition worsens, or new information becomes available.

If you wish to participate in this study, you should sign below.

Patient Signature: ____ Date: _____________

CONFIDENTIAL

Signature of Doctor: ____ Date: ____________

Signature of Person Obtaining Consent: ________________________ Date: ____________

Signature of Translator: _____________________________________ Date: ____________

CONFIDENTIAL

I-SPY 2 TRIAL
(*Investigation of Serial Studies to Predict Your Therapeutic Response with Imaging And moLecular Analysis 2*)

SUPPLEMENT FOR PEMBROLIZUMAB TREATMENT

You have already been given the treatment consent form for this study. This section of the consent form will only address the side effects and risks of this specific drug you will receive with paclitaxel and the treatment schedule.

You have been randomized to the treatment group that receives pembrolizumab along with Paclitaxel.

You will receive the following combination of drugs:

Drug Name	How treatment will be given	How long treatment will take each time	How often treatment is given	When treatment will be given
Paclitaxel	By vein	1 hour	Weekly	Weeks 1–12
Pembrolizumab	By vein	30 minutes	Every three weeks	Weeks 1,4,7,10

This treatment regimen will last for a total of 12 weeks after which you will receive AC as described in the treatment consent form.

What is Pembrolizumab?

Pembrolizumab is an investigational drug, meaning it has not been approved by the FDA for use in this way. Pembrolizumab is an antibody that blocks the PD-1 (programmed cell death 1) receptor. By blocking this receptor pembrolizumab helps the immune system identify and fight the cancer cells.

Pembrolizumab is also known as KEYTRUDA (approved in *USA and several other countries*) and is available by prescription to treat a type of skin cancer called malignant melanoma.

Pembrolizumab/KEYTRUDA is being studied by the Sponsor to see if it is effective in treating more than 30 types of cancer and to see what side effects are associated with its use.

As of 30-June-2015, pembrolizumab/KEYTRUDA had been given to about 9400 patients with various cancers in clinical trials. Men and women with cancer were treated, some for up to approximately 1.5 years. Safety was studied across several cancers treated with different doses: 2 mg/kg every 3 weeks, and 10 mg/kg every 2 or 3 weeks. The side effects seen were similar.

What additional tests and procedures can I expect from this additional drug while on the study?

To help monitor your health while receiving this treatment, you will have the following additional tests and procedures:

- Laboratory safety blood tests specific for pembrolizumab will be added to your standard of care safety blood tests

CONFIDENTIAL

- Collection of archival (“leftover”) tumor tissue – If available, your doctor will also obtain leftover tumor tissue from your biopsy at diagnosis.

The cost of these additional tests and procedures will be covered by the study.

What side effects or risks can I expect from these additional drugs while on the study?

You may have side effects while on the study. Everyone taking part in the study will be watched carefully for any side effects. However, doctors and the study sponsor don’t know all the side effects that may happen and there may be unknown side effects that could occur. Side effects may be mild or very serious. Your health care team may give you medicines to help lessen the side effects. Many side effects go away soon after you stop what is causing them. In some cases, side effects can be serious or long lasting, causing hospitalization, and/or may never go away. There is also a risk of death. You may experience unexpected side effects that may not allow you to complete your standard treatment. While it is extremely unlikely, these side effects may be long lasting and may possibly prevent you from receiving future standard or experimental therapies.

You should talk to your study doctor about any side effects you experience while taking part in the study.

You should also be aware that some investigational drugs will stop being assigned to new patients entering the trial as the trial continues. Once the study has gathered enough information about a particular investigational drug (good or bad), it will leave the trial and another new investigational drug will enter the trial. If the investigational drug leaves the study while you are still receiving it, you have 3 choices:

7. Continue taking the investigational drug and continue the treatment phase of the trial.
8. Stop taking the investigational drug and continue the treatment phase of the trial.
9. Stop taking the investigational drug and stop participating in the rest of the treatment phase of the trial.

Also, the investigational drug may leave the study because of severe side effects. If the investigational drug is removed for this reason while you are still receiving the drug, you will stop receiving the drug immediately. You will have the choice to continue or stop participating in the study.

The pharmaceutical company that manufactures this drug, Merck, will be notified of all side effects and any pregnancy that you may experience while you are part of this study.

Reproductive risks:

- You should not become pregnant while on this study. If you suspect that you have become pregnant at any time during the study or within 3 months after your last dose of chemotherapy, please notify your study doctor immediately. It is not known whether the investigational drugs can cause harm to the fetus when administered to a pregnant woman or if it affects the ability of a woman to become pregnant

Potential Side Effects of Investigational Treatment

CONFIDENTIAL

	Side Effects		
**Drug Treatment**	**Likely**<br>**(seen in 10% or more of patients)**	**Less Likely**<br>**(Seen in less than 10% of patients)**	**Rare but Serious**<br>**(Seen in 1–4% or less of patients but might be serious)**
**Paclitaxel + pembrolizumab**	• Fatigue<br>• Cough<br>• Shortness of breath<br>• Nausea<br>• Pruritus(itching of skin)<br>• Rash<br>• Decreased appetite<br>• Joint pain<br>• Fever<br>• Swelling of legs and/or feet<br>• Weakness<br>• Diarrhea<br>• Constipation<br>• Back pain<br>• Stomach pain<br>• Vomiting<br>• Hair loss (alopecia)<br>• Low level of salt in the blood that may cause you to feel tired, confused, headache, muscle cramps, or upset stomach<br>• Decreased white blood cell count (which may lead to increased risk of infection)<br>• Temporary lowering of red blood cells (anemia)<br>• Temporary lowering of blood platelet cells (thrombocytopenia)<br>• Increase in blood enzymes that monitor for and indicate liver damage (transaminases and/or alkaline phosphatase)	• Edema (swelling of tissues in limbs, face, or around the eyes)<br>• Fever and/or chills<br>• Weight loss<br>• Dehydration<br>• Headaches<br>• Allergic reaction:<br>- Blood pressure changes (increase or decrease)<br>- Cough<br>- Dizziness<br>- Feeling cold<br>- Feeling that the tongue is swelling or airway is closing and trouble breathing<br>- Fever<br>- Headache<br>- Join pain<br>- Muscle pains<br>- Nausea<br>- Rash, hives, itching<br>- Shortness of breath<br>- Sweating<br>- Tiredeness<br>- vominiting<br>• Mucosal inflammation (inflammation affecting moist tissue lining parts of the inside of your body, such as mouth, nose, lungs, and digestive tract)	• Severe bone marrow suppression with decreased white blood cell count, anemia or reduced platelets<br>• Elevations of blood liver tests indicating potentially severe liver injury<br>• Kidney failure<br>• Severe lung disease that can cause trouble breathing<br>• Damage to heart muscle that can lead to heart failure<br>• Pneumonia,<br>• Infection of the skin (cellulitis).<br>• Inflammation of the skin so you may have widespread peeling of the skin, itching, skin redness<br>• Inflammation of the colon (colitis)<br>• Inflammation of the kidney, which can cause kidney damage<br>• Inflammation of the pituitary gland<br>• Overactive or underactive thyroid<br>• Inflamation of the liver (hepatitis)

CONFIDENTIAL

	Side Effects		
**Drug Treatment**	**<u>Likely</u> (seen in 10% or more of patients)**	**<u>Less Likely</u> (Seen in less than 10% of patients)**	**<u>Rare but Serious</u> (Seen in 1–4% or less of patients but might be serious)**
	• Abnormal sense of taste<br>• Tingling and numbness in hands and feet<br>• Bone and muscle aches<br>• White patches on the skin		• Inflammation of the lungs so you may feel short of breath and cough. Rarely this might lead to death.<br>• Inflammation of the bowels/gut that can cause stomach pain with diarrhea, or stools that are black, tarry, sticky, or have blood or mucus<br>• Inflammation of the muscles so you may feel weak or pain in the muscles<br>• Inflammation of the pancreas (a gland in your abdomen that controls sugar levels) so you may have severe upper abdominal pain that may move to the back, sick to your stomach, and vomiting that gets worse when you eat<br>• Inflammation of the eye so you may have redness of the eye, blurred vision, sensitive to light, have eye pain, see floaters or have headaches<br>• Dizziness or fainting (low blood pressure), flushing, rash,

CONFIDENTIAL

	Side Effects		
**Drug Treatment**	**Likely** (seen in 10% or more of patients)	**Less Likely** (Seen in less than 10% of patients)	**Rare but Serious** (Seen in 1–4% or less of patients but might be serious)
			fever, shortness of breath or upset stomach at the time of receiving your infusion (IV) or just after, or pain at the site of infusion<br>• Inflammation of the pancreas (diabetes) so you may have too much sugar in your blood, may need to urinate more often, lose weight, feel thirsty, and may need regular insulin shots<br>• Inflammation of the nerves that may cause pain, weakness or tingling in the hands and feet, and may spread to the legs, arms and upper body leading to severe muscle weakness

CONFIDENTIAL

Unknown Risks:
There may be other risks or side effects from pembrolizumab that are not listed above or unknown at this time. It is also possible to experience a serious allergic reaction, which could become life-threatening or fatal. Symptoms of an allergic reaction include rash, hives, itching, swelling of the mouth, face, lips or tongue, dizziness, tightness in the chest, or trouble breathing. Let your study doctor know if you experience any of these symptoms.

Additional Information You Need to Know:
You will be told in a timely manner about significant new information that might affect your decision to stay in the study.

CONSENT
You will be given copies of this consent form to keep along with the standard chemotherapy treatment consent form.

PARTICIPATION IN RESEARCH IS VOLUNTARY. You have the right to decline to participate or withdraw at any point in this study without jeopardy to your medical care. Leaving the study will not result in any penalty or loss of benefits to which you are entitled. The study doctor may decide to take you off this study with or without your consent if it is in your best medical interest, funding is stopped, your condition worsens, or new information becomes available.

If you wish to participate in this study, you should sign below.

Patient Signature: ____

Date: ____

Signature of Doctor: ____

Date: ____

Signature of Person Obtaining Consent: _________________________

Date: ____

Signature of Translator: ______________________________________

Date: ____

CONFIDENTIAL

I-SPY 2 TRIAL
*(**I**nvestigation of **S**erial Studies to **P**redict **Y**our **T**herapeutic **R**esponse with **I**maging **A**nd mo**L**ecular Analysis **2**)*

I-SPY 2 SUBSTUDY: SURMOUNT

**Protocol Title:**	**SURMOUNT**<br>**Surveillance Markers of Utility for Recurrence after Neoadjuvant Therapy for Breast Cancer**
**Principal Investigator:**	Angela DeMichele, MD, MSCE<br>Perelman Center for Advanced Medicine, 3 West<br>3400 Civic Center Blvd, Philadelphia, PA 19104<br>(215) 614 -1850
**Emergency Contact:**	During normal office hours:<br>Angela DeMichele, MD, MSCE<br>(215) 614 - 1850<br><br>Danielle Soucier, MPH, MBE<br>*(215)-615-6714*
**UPCC Number:**	**UPCC 16113 - UPENN**
**UPENN IRB Number:**	**818165 - UPENN**

CONFIDENTIAL

Why am I being asked to volunteer?

You are being invited to participate in this research study because you are enrolled in the I-SPY2 TRIAL and, are receiving chemotherapy for breast cancer and are planning to undergo surgery to remove any remaining tumor after the completion of your chemotherapy. Your participation in this research is voluntary. You will be asked to sign this form and will receive a copy if you give your consent to participate. Please take time to read over the consent and ask any questions you may have.

You are being invited to participate in a research study. Your participation is voluntary which means you can choose whether or not you want to participate. If you choose not to participate, there will be no loss of benefits to which you are otherwise entitled. Before you

can make your decision, you will need to know what the study is about, the possible risks and benefits of being in this study, and what you will have to do in this study. The research team is going to talk to you about the research study, and they will give you this consent form to read. You may also decide to discuss it with your family, friends, or family doctor. You may find some of the medical language difficult to understand. Please ask the study doctor and/or the research team about this form and anything you do not understand. If you decide to participate, you will be asked to sign this form.

What is the purpose of this research study?
The purpose of this research is to study markers in the blood and bone marrow that could help us in the future predict which patients are at increased risk of their cancer returning. We will use the results of this research to improve the treatment and prevention of breast cancer in the future.

How long will I be in the study? How many other people will be in the study?

You will be in the study for up to ten years. There will be a total of 350 patients enrolled into this study.

What am I being asked to do?

If you choose to take part, then you will be asked to provide both blood and bone marrow samples. The procedures are listed below. They are common medical procedures, but are for research purposes only (they are not considered part of your routine care).

Blood Samples You will have up to 13 blood samples (total of about a pint of blood over 10 years) collected over the course of the study. You will have 1 additional blood sample (about 3 tablespoons) collected if your cancer recurs.

- **Enrollment:** A blood sample (about 1.5 tablespoons) will be collected around the time you enroll into the study.
- **Surgery:** A blood sample (about 2 tablespoons) will be collected at your scheduled surgery for the removal of any residual breast tumor.
- **Post-operative:** A blood sample (about 1.5 tablespoons) will be collected at your appointment after surgery.
- **Annual follow-up visits:** A blood sample (about 3 tablespoons) will be collected annually during the follow-up period (up to 10 years) when you return for your routine oncologic check-ups, for a maximum of 10 blood samples.
- **Recurrence:** If your cancer recurs, a blood sample (about 3 tablespoons) will be collected during a visit around the time of your recurrence.

Bone Marrow Samples
You will have 1 bone marrow sample (about 1 tablespoon) collected over the course of the study in addition to the blood samples described above. Bone marrow collection is a commonly performed medical procedure in which a needle is inserted into the back of the pelvic bone and a syringe is used to remove a liquid sample of bone marrow. Your study doctor will discuss with you whether to perform the bone marrow collection in the clinic or while you are under general anesthesia for your breast surgery. If done in the clinic, we will use local anesthesia (numbing medicine) to numb the skin, the tissue underneath it, and the surface of the bone. If, during the

CONFIDENTIAL

course of the follow-up period (10 years), your cancer returns, the study team will also ask you to provide 1 additional bone marrow sample (about 1 tablespoon).

- **Surgery:** A bone marrow sample (about 1 tablespoon) will be collected at your scheduled surgery or at a separate time in the clinic around the time of your surgery. This will take about 10 minutes if performed during surgery, or about 30 minutes if performed at a separate time in the clinic.
- **Recurrence:** If your cancer recurs, a bone marrow sample (about 1 tablespoon) will be collected around the time of your recurrence. This will take about 10 minutes if performed during surgery, or about 30 minutes if performed at a separate time in the clinic.

We would like to keep track of your medical condition for up to ten years. We would like to do this by speaking with you during your annual follow-up appointment and by reviewing your medical records. Your medical information will be collected for the first 5 years of follow-up as part of your participation in the I-SPY 2 Trial. The SURMOUNT study will add-on an additional five years of follow-up data collection. Keeping in touch with you and checking on your condition every year helps us look at the long-term effects being examined in this study. As in I-SPY 2, we are requesting your permission to collect medical information from your chart and link this information to your specimens (blood samples, bone marrow) so that we may better understand how the research findings might be related to patient outcomes. This information will include general information about you (e.g., age, race/ethnicity), information about your cancer and its treatment, and information about your other medical conditions, your condition following treatment, and any new tests or treatments you may undergo.

Finally, we are requesting your permission to re-contact you in the future for new studies or questions that may develop as we perform this research. It is important to note that when research is performed on these specimens, the investigators conducting this research will protect the access to any identifying information such as your name or medical record number.

<u>To summarize, you will be asked to provide the following</u>:

- Blood sample after the completion of your treatment
- Blood sample, and bone marrow sample during your scheduled surgery. The bone marrow sample may alternatively be collected during a clinic appointment. The decisions regarding the collection time of the bone marrow sample will be made with your clinical team.
- Blood sample at a post operative appointment
- A blood sample annually for the 10 year follow-up period
- Blood sample and bone marrow sample if your cancer recurs
- Medical information monitored and updated for changes in your disease status

While you will not benefit personally from participating in this study, we hope that the information we gain will help us improve treatments and prevention methods for breast cancer in the future. New tests or discoveries from this research may have potential commercial value but subjects will not share in any financial benefits.

Because this study is a collaborative effort with the I-SPY 2 TRIAL, we will be asking the I-SPY 2 study team for access to study data currently being collected in the I SPY 2 TRIAL. This includes data from your treatment outcomes and early follow-up period as well as data that are obtained during the analysis of the tissue and blood specimens collected for the I-SPY 2 TRIAL. We will also be sharing information about your participation in this study with I-SPY 2 investigators, within the limits set by the I-SPY 2 informed consent form.

CONFIDENTIAL

What are the possible risks or discomforts?

This study might involve the following risks and/or discomforts to you. In addition, there may be uncommon or previously unrecognized risks that might occur. If you become injured, inform the treating physician that you are enrolled in this research study.

Risks Associated with Study Procedures

Blood Samples: You will be asked to provide several blood samples (each research blood draw being about 3 tablespoons) at different points in this study. Occasionally there are minor complications to a blood draw including pain, bleeding, bruising, swelling, fainting and/or infections at the need insertion site.

Bone Marrow Samples: You will also be asked to provide bone marrow (about 1 tablespoon). Although this procedure is not considered part of your routine care, it is generally considered a safe procedure, but it is important for you to be aware of the following risks:

- Discomfort or bruising at the collection site, which often lasts for 1-4 days after the procedure.
- Bleeding from the biopsy site, which is usually only a small amount that has stopped by the end of the procedure. More serious bleeding is possible but happens rarely.
- Pain, redness or swelling at the collection site.
- Infection at the collection site, which is very rare

Your study doctor will discuss with you whether to perform the bone marrow collection in the office or while you are under anesthesia for your breast surgery. If done in the office, we will use local anesthesia (numbing medicine) to numb the skin, the tissue underneath it, and the surface of the bone. Despite the numbing medicine, many patients experience discomfort or pain for a few seconds while the syringe is being used to remove the bone marrow sample. If the procedure is done while you are under general anesthesia for the breast surgery, you will not likely feel any pain or discomfort at the time of the bone marrow aspiration.

Privacy: Finally, there is a small risk of release of medical records that is not intended. In order to minimize this risk, your I-SPY 2 patient ID will be used for this study. This is not the same number as your medical record number. Your specimens (blood samples, bone marrow) will also be assigned a unique code. Any material that we share with others will be supplied with the code numbers only, and without your name or medical record number. All study information will be stored in locked file cabinets and in password-protected computer files. Only authorized study personnel will have access to these files.

You should talk to your study doctor about any side effects that you have while taking part in the study.

Risks of Germline Genetic Testing

In the course of this research, your blood and tissue may be used for genetic tests to understand abnormal genes that are passed on in families. This testing would be performed on "germline DNA", the DNA that is present in all of your cells. In some cases, this type of testing can predict your risk of future illness. In this study, we will be looking at genes in your tumor research samples to examine the genetic changes that make your cancer unique. Germline testing on your sample

CONFIDENTIAL

may be performed only to help us focus specifically on which genetic changes are specific to your tumor. We will not be performing specific germline testing that could be informative about your risk of future illness. Reports about research done on germline DNA not be given to you or your doctor, or put into your health record without your prior consent and approval.

What if new information becomes available about the study?

During the course of the study, we may find more information that could be important to you. This includes information that, once learned, might cause you to change your mind about being in the study. If we discover new information about the study that could affect your decision to stay in the study, you will be notified as soon as possible. You will be able to ask questions about this information and can discuss it with your family, friends, or doctor. It is always your decision to continue in the study or leave the study.

What are the possible benefits of the study?

The study of your blood and bone marrow samples obtained for research purposes may result in the discovery of better ways to diagnose, predict outcomes, or treat patients with breast cancer. Should this occur, you will not receive financial compensation related to those medical or scientific discoveries or products. You are not expected to get any direct benefit from being in this research study other than the anticipated benefits to society.

Will I get my test results?

The tests being done on your specimens are primarily research tests and their meaning is not yet fully understood. They cannot and should not be used to make medical decisions about your treatment. For this reason, these reports will not be placed into your health record and the research findings will not have an effect on your care. As we learn more about the meaning of these tests, we may approach you to ask if you want to receive the results. After the research has been explained, you would decide whether to receive the results or to decline them. Tell us, by checking the correct box below, if you would like to have these research results returned to you at the end of the study. If you consent to this process, we will contact you or your designate when these research results are available.

☐ I would like to be contacted in the future to have my research results returned to me

☐ I do not wish to be contacted in the future to have my research results returned to me

What other choices do I have if I do not participate?

You do not have to participate in this research study in order to receive treatment. Your treatment will not be affected in any way, whether or not you participate in this study. You should discuss this with your physician.

Will I be paid for being in this study?

You will not be paid for any part of your participation in this study, nor will you be paid for the use of your blood samples or bone marrow now or in the future. You will also not be paid for any idea

CONFIDENTIAL

or product that may be developed as a result of this study.

Will I have to pay for anything?

You and your insurance company will pay for the costs of the routine blood tests, X-rays, scans, and other routine laboratory tests that are part of your standard medical care. The cost of the bone marrow aspirate and blood samples will be covered by the study. Neither you nor your insurance company will be asked to pay for any research tests. You also will not be asked to pay for the cost of research testing on your blood samples or bone marrow. Please ask about any unexpected added costs or insurance problems.

What happens if I am injured or hurt during the study?

If you have a medical emergency during the study you should go to the nearest emergency room. You may contact the Principal Investigator or Emergency contact listed on page one of this form. You may also contact your doctor, or seek treatment outside of the University of Pennsylvania. Be sure to tell the doctor or his/her staff that you are in a research study being conducted at the University of Pennsylvania. Ask them to call the telephone numbers on the first page of this consent form for further instructions or information about your care.

In the event that you are hurt or injured as a result of participation in this research study, please contact the investigator listed on page one of this form.

We will offer you the care needed to treat injuries directly resulting from taking part in this research. We may bill your insurance company or other third parties, if appropriate, for the costs of the care you get for the injury, but you may also be responsible for some of them.

There are no plans for the University of Pennsylvania to pay you or give you other compensation for the injury. You do not give up your legal rights by signing this form.

If you think you have been injured as a result of taking part in this research study, tell the person in charge of the research as soon as possible. The researcher's name and phone number are listed on page one of this form

When is the study over? Can I leave the study before it ends?

If you decide to participate, you are free to leave the study at any time. Withdrawal will not interfere with your current or future care. The investigators also have the right to stop your participation at any time. This could be because you have had an unexpected reaction to a study procedure, or have failed to follow instructions, or because the entire study has been stopped.

If in the future you decide that you no longer wish to participate in the study, it is your right to do so. If you decide to withdraw from study participation, we will use information obtained from the samples previously provided unless you ask us not to. To do this, you must request in writing that your specimens be disposed of according to standard medical research procedures. Please note that all specimens or data distributed prior to withdrawal will not be retrieved. If you do not make such a request in writing, your participation in the study will continue until the study completion and the specimens will be used and stored indefinitely.

CONFIDENTIAL

The study is expected to end after all participants have completed all visits and all information has been collected. This study may also be stopped at any time by your physician or the study Sponsor without your consent because:

- The primary investigator feels it is necessary for your health or safety. Such an action would not require your consent, but you will be informed if such a decision is made and the reason for this decision.
- You have not followed study instructions
- The sponsor of or the study principal investigator has decided to stop the study

What information about me may be collected, used or shared with others?

Your protected health information (name, date of birth, zip code, phone number, medical record number) will be collected at your site as part of the I-SPY2 TRIAL and will not be shared data for this study.
Your initials, study site, and your unique code number will be used and shared as part of this study to identify your data, blood samples, and bone marrow.

Why is my information being used?

Your protected health information is used by your doctor to contact you during the study. Your initials, study site, and unique code number, along with the results of the tests and procedures as part of this study will be used to:

- do the research
- oversee the research
- to see that the research was done right.

Who may use and share information about me?

The following individuals may use or share your information for this research study:

- **Dr. Angela DeMichele, MD, MSCE and her team**
- **The University of Pennsylvania Institutional Review Board**
- **Other authorized personnel at the University of Pennsylvania**
- **The Office of Human Research Protection**
- **Other Regulatory Bodies**
- **Other collaborating researchers including but not limited to:**
    - **Laura Esserman, MD, MBA and her research team – University of California San Francisco**
    - **John Park, MD, PhD and his research team – University of California San Francisco**
    - **Approved I-SPY 2 TRIAL Study Team Researchers**
- **Janssen Diagnostics, LLC – a company that is providing support for some of the analysis that will be conducted on your blood samples. The company will receive only de-identified data from this study, which would list you only by study code number. We will not be sharing with the company any of your personal and private information.**

CONFIDENTIAL

Once your personal health information is disclosed to others outside the School of Medicine, it may no longer be covered by federal privacy protection regulations.

The Principal Investigator or study staff will inform you if there are any additions to the list above during your active participation in the trial. Any additions will be subject to University of Pennsylvania procedures developed to protect your privacy.

Who can see or use my information? How will my personal information be protected?

Donating specimens may involve a loss of privacy, but information about you will be handled as confidentially as possible. Study participants will not be identified in any report or publication about this study. If information from this study is published or presented at scientific meetings, your name and other personal information will not be used. Although every effort will be made to keep research records private, there may be times when federal or state law requires the disclosure of such records, including personal information.

We will do our best to make sure that the personal information obtained during the course of this research study in your medical record will be kept private. However, we cannot guarantee total privacy. Your personal information may be given out if required by law. If information from this study is published or presented at scientific meetings, your name and other personal information will not be used.

Organizations that may inspect and/or copy your research records for quality assurance and data analysis include groups such as study personnel, representatives from the Food and Drug Administration (FDA), and the Institutional Review Board for the University of Pennsylvania. By signing this consent form, you authorize such access to your medical records.

How long may the School of Medicine use or disclose my information?

Your permission to use of your initials, study site, and unique code number for this specific study does not expire. There is no plan to destroy your data or specimens after the conclusion of this study. A unique identifier will be assigned to your specimens and data. The link between your unique identifier and data/specimens will only be accessible to key study personnel and will be locked in a secure database. For this reason your data and specimens will used and retained indefinitely.

Your information may be held in a research database. However, the School of Medicine may not re-use or re-disclose information collected in this study for a purpose other than this study unless:

- **You have given written authorization**
- **The University of Pennsylvania's Institutional Review Board grants permission**
- **As permitted by law**

Can I change my mind about giving permission for use of my information?

Yes. You may withdraw or take away your permission to use and disclose your health information – blood samples, and bone marrow to study investigators at any time. You do this by sending written notice to the investigator for the study. If you withdraw your permission, you will not be able to stay in this study.

Electronic Medical Records and Research Results

CONFIDENTIAL

Reports about research done with your blood samples, and bone marrow will not be given to you or your doctor. These reports will not be placed into your health record. The research will not have an effect on your care. We are not returning results of this research to you or your doctor because we do not yet know if these results will have any importance to your health or treatment.

What are my rights if I take part in this study? Who can I call about my rights?

Taking part in this study is your choice. You may choose either to take part or not to take part in the study. If you decide to take part in this study, you may leave the study at any time. No matter what decision you make, there will be no penalty to you and you will not lose any of your regular benefits. Leaving the study will not affect your medical care. You can still get your medical care from our institution.

We will tell you about new information or changes in the study that may affect your health or your willingness to continue in the study.

In the case of injury resulting from this study, you do not lose any of your legal rights to seek payment by signing this form.

If you have questions regarding your participation in this research study or if you have any questions about your rights as a research subject don't hesitate to speak with the Principal Investigator listed on page one of this form. Concerning your rights as a research subject, you may also contact the Office of Regulatory Affairs at the University of Pennsylvania by calling (215) 898-2614.

When you sign this form, you are agreeing to take part in this research study. This means that you have read the consent form, your questions have been answered, and you have decided to volunteer. Your signature also means that you are permitting the University of Pennsylvania Health System and the School of Medicine to use your personal health information collected about you for research purposes within our institution. You are also allowing the University of Pennsylvania Health System and the School of Medicine to disclose that personal health information to outside organizations or people involved with the operations of this study.

A copy of this consent form will be given to you.

________________________ ____________________________________ ___________

Name of Subject (Please Print) Signature of Subject Date

CONFIDENTIAL

Name of Person Obtaining Consent (Please Print)	Signature	Date

CONFIDENTIAL

Consent Form for Use of Leftover Biospecimens for Research

About Using Blood Samples and Bone Marrow for Research

We would like to obtain your consent for the use of leftover specimens for research. If you agree, these samples will be kept and may be used in research to learn more about cancer and other diseases. This includes leftover blood samples, serum, plasma, and buffy coat cells and bone marrow from the samples we collect. We would like to keep this for future research.

The research that may be done with your blood samples and bone marrow is not designed specifically to help you. It might help people who have cancer and other diseases in the future.

Reports about research done with your blood samples and bone marrow will not be given to you or your doctor. These reports will not be put in your health record. The research will not have an effect on your care.

Things to Think About

The choice to let us keep the left over specimens (blood samples and bone marrow) for future research is up to you. No matter what you decide to do, it will not affect your care. If you decide now that your specimens can be kept for research, you can change your mind at any time. Just contact us and let us know that you do not want us to use your specimens. Then any specimen that remains will no longer be used for research.

In the future, people who do research may need to know more about your health. While the investigator(s) may give them reports about your health, we will not give them your name, address, phone number, or any other information that will let the researchers know who you are.

Sometimes specimens are used for genetic research (about diseases that are passed on in families). Even if your specimens are used for this kind of research, the results will not be put in your health records.

Your specimens will be used only for research and will not be sold. The research done with your specimens may help to develop new products in the future.

Benefits

The benefits of research using specimens include learning more about what causes cancer and other diseases, how to prevent them, and how to treat them.

Risks

The greatest risk to you is the release of information from your health records. We will do our best to make sure that your personal information will be kept private. The chance that this information will be given to someone else is very small.

CONFIDENTIAL

Making Your Choice

Please read each sentence below and think about your choice. Please make your choice by selecting "yes" or "no" after each statement. If you have any questions, please talk to your doctor or nurse, or call our institutional review board at 215-898-2614. **No matter what you decide to do, it will not affect your care**

My blood samples (serum, plasma, and buffy coat cells) and bone marrow may be kept for use in future research to learn about, prevent, or treat cancer.	☐ Yes ☐ No
My blood samples (serum, plasma, and buffy coat cells) and bone marrow may be kept for use in future research to learn about, prevent or treat other health problems (for example: diabetes, Alzheimer's disease, or heart disease).	☐ Yes ☐ No
Someone may contact me in the future to ask me to take part in more research	☐ Yes ☐ No

Where can I get more information?
You may call the National Cancer Institute's Cancer Information Service at:

1-800-4-CANCER (1-800-422-6237) or TTY: 1-800-332-8615

- You may also visit the NCI Web site at http://cancer.gov/
- For NCI's clinical trials information, go to: http://cancer.gov/clinicaltrials/
- For NCI's general information about cancer, go to http://cancer.gov/cancerinfo/

You will get a copy of this form. If you want more information about this study, ask your study doctor.

Signature

_________________________ _______________________________ __________
Name of Subject (Please Print) Signature of Subject Date

CONFIDENTIAL

Appendix A
I-SPY 2 Statistical Considerations

Notation

Let $y_i \in \{0,1\}$ be the indicator for the pCR response of patient i $(i = 1, \ldots, N)$.

Let $x_{1i}, x_{2i}, x_{3i} \in \{0,1\}$ represent the HR, HER2 and MP values respectively for patient i (with 1 denoting positive, and 0 denoting negative status for HR and HER2; and 1 and 0 denoting 'High' and 'Low' for MP). We index the eight subtypes defined by the values of (HR, HER2, MP) as {0,1,2,3,4,5,6,7} corresponding to {0,0,0), (0,0,1), (0,1,0), (0,1,1) ,(1,0,0), (1,0,1), (1,1,0), (1,1,1)}.

If T_0, T_1, T_2, T_3 are the tumor sizes at baseline, one month, three months and just prior to surgery, then we define the tumor size response as $r_t = \frac{(T_t - T_0)}{T_0}$ for $t = 1,2,3$. Negative values of r_t correspond to a decrease in tumor size, with $r_t = -1.0$ being the lowest value and greatest improvement (indicating the tumor size is not detectable by MRI). In order to formulate a multiple imputation model for the pCR response in cases where the pCR value is not available, we discretize the range of r_t values which we denote as m_t. The mapping between r_t values and the corresponding m_t is given in Table 1.

Table 1 : Categories for tumor size reduction

Category (m_t)	Fraction Increased Tumor Size (r_t)
1	≥ 0
2	$(-0.1,0]$
3	$(-0.2, -0.1]$
4	$(-0.3, -0.2]$
5	$(-0.4, -0.3]$
6	$(-0.5, -0.4]$
7	$(-0.6, -0.5]$
8	$(-0.7, -0.6]$
9	$(-0.8, -0.7]$
10	$(-0.9, -0.8]$
11	$(-0.95, -0.9]$
12	$(-0.99, -0.95]$
13	< -0.99

Let m_{1i}, m_{2i}, m_{3i} respectively be the MRI tumor size responses categories for each post-baseline MRI for patient i.
We represent the treatment arms generically as $a \in \{0,1,2, \ldots, A\}$, with $a = 0$ being the control arm. Let patient i's treatment arm be denoted by a_i.
At any interim analysis, for a given patient i, it is possible that the pCR value or one or more of the tumor size data may not be available.

CONFIDENTIAL

The pCR Response Model

The pCR response is modeled using a Bayesian logistic regression model

$$y_i \sim \text{Bernoulli}(p_i)$$
$$\text{logit}(p_i) = \beta_0 + \beta_1 x_{1i} + \beta_2 x_{2i} + \beta_3 x_{3i} + \theta_{t_i} + \gamma_{1,t_i} x_{1i} + \gamma_{2,t_i} x_{2i} + \gamma_{3,t_i} x_{3i}$$

(with $\theta_0 = \gamma_{1,0} = \gamma_{2,0} = \gamma_{3,0} = 0$ to ensure parameter identifiability).

For each of the coefficients Θ in the regression model, we assume independent normal prior distributions: $\Theta \sim N(\mu_\Theta, \sigma_\Theta^2) = N(0,1)$.

We also include the data from the Control arm of the I-SPY 1 trial as historical prior information. However, the likelihood function evaluation corresponding to the I-SPY1 data is raised to the power of 0.2 to reflect a discounting or weak borrowing.

If all the pCR data y_i were available, then this is a standard Bayesian logistic regression model that may be fit using Markov Chain Monte Carlo (MCMC) methods. However, since the pCR response data for some patients might not be available, we use an imputation model (described below) based on the patients with pCR data to impute the missing pCR values. We then use the imputed values in the logistic regression model. The imputation model is also a closely related Bayesian logistic regression model that is fit using a MCMC algorithm. The two MCMC algorithms for the pCR response and the imputation model are interleaved: For every MCMC sample of the imputation model parameters, we impute the missing pCR values and use them to draw the next posterior sample from the above model. Thus, this procedure constitutes a multiple imputation scheme for the missing pCR values. Consequently, the uncertainty due to the missing values is appropriately reflected in the posterior estimates of the above model parameters.

Imputation Model

Although the final pCR response might not be available for every patient, useful information about the likely pCR reponse for these subjects may be available in the form of MRI assessments of the tumor size at one or more times prior to surgery. For predicting missing pCR, we employ simple imputation models that exploit the tumor size MRI data. We fit a set of logistic regression models to predict the pCR value based on the MRI response at each of the three time points when the MRI data are available.

Specifically, for each time point $t = 1,2,3$, we use data from all patients for whom we have both the pCR response and MRI assessment r_t available, and we fit the model:

$$\text{logit}(\pi_{t,i}) = \text{logit}(p_{x_i}) + \alpha_{t,j_i}$$

Where $\alpha_{t,j_i}$is the parameter corresponding to the category j_i (see Table 1) for subject i at time t and $p_{x_i}$is the parameter from the logistic regression for pCR corresponding to $x_i = (x_{1i}, x_{2i}, x_{3i})$, the genetic type of subject i. We impose a monotonicity constraint of $\alpha_{t,l} \leq \alpha_{t,m}$for $l < m$ to reflect the condition that a 'higher' category (i.e., larger tumor reduction) is more likely to lead to a pCR than a 'lower' category. The informal intuitive notion behind this model is that it acts as a simple categorical prediction model to the 'residual' from the main logistic regression model for pCR. For each of the coefficients α in the regression model, we assume independent normal prior distributions: $\alpha \sim N(\mu_\alpha, \sigma_\alpha^2) = N(0,1)$.
At each MCMC iteration, if the pCR response for subject j is missing, then it is imputed as

CONFIDENTIAL

$$\hat{y}_j \sim \text{Bernoulli}(\pi_{T,j})$$

Where T is the latest time point for which we have a tumor response measurement for subject j.

The Prevalence Model

The population prevalence rate across the 8 subtypes defined by the (HR,HER2, MP) values is modeled as a categorical distribution (i.e., multinomial distribution with n=1) over the subtypes indexed by {0,1,..7}, with $Pr(C = c) = \phi_c$. We use a Dirichlet prior

$$(\phi_0, \phi_1, \ldots, \phi_7) \sim Dirichlet(0.1, 0.1, \ldots, 0.1)$$

Which results in a posterior distribution

$$(\phi_0, \phi_1, \ldots, \phi_7) | Data \sim Dirichlet(n_0 + 0.1, n_1 + 0.1, \ldots, n_7 + 0.1)$$

pCR Response Probabilities for Subtypes and Signatures

The response-adaptive prescriptions that govern the trial conduct are specified in terms of the probability of pCR response (by treatment arm) for each of the eight subtypes and each of the ten signatures. These probabilies are derived from the parameters of the dose response and prevalence models as follows.

Let $\pi_C(a)$ be the probability of pCR for treatment arm 'a' in genetic subtype 'C'. If C represents (x_1, x_2, x_3) where $x_i \in \{0,1\}$ for the (HR,HER2,MP) value, then

$$\text{logit}(\pi_C(a)) = \beta_0 + \beta_1 x_1 + \beta_2 x_2 + \beta_3 x_3 + \theta_a + \gamma_{1,a} x_1 + \gamma_{2,a} x_2 + \gamma_{3,a} x_{3a}$$

Let $\text{P}_S(a)$ be the probability of pCR for treatment arm 'a' for signature 'S'. Then

$$\text{P}_S(\text{a}) = \frac{\sum_{C \in S} \pi_C(a) \cdot \phi_C}{\sum_{C \in S} \phi_C}$$

(The notation $C \in S$means subtype 'C' is included in signature 'S'.)

Adaptive Treatment Allocation

For response-adaptive treatment allocation for a new patient, we update the posterior distribution based on the latest available data. For any subtype, 'C', and any experimental treatment arm, 'A', we compute the probability that the pCR response for 'A' is higher than the Control arm [PrCtl(C,A)].
If the new patient is of subtype 'c', then she is assigned to Control with a 20% probability, and the assignment probability to any experimental arm, 'a', eligible for subtype 'c' with probability proportional to PrCtl(c,a) (with the probabilities being appropriately normalized over the eligible experimental arms to add up to 80%).

CONFIDENTIAL

Predictive Probabilities Used for Determine Graduation and Futility Stopping

The criteria for graduation and early stopping for futility are based on the predictive probability of success for an experimental arm in the 10 specified signatures. The 'predicitive probability of success' is the predictive probability that an arm will be found to be significantly better than the control treatment in an equally randomized 2-arm Phase III trial with 150 patients/arm that uses a 1-sided test of the hypothesis that the experimental arm's pCR is larger than that of the control arm at the 0.025 significance level.

A sufficiently high predictive probability of success for a signature qualifies an experimental arm to be eligible to graduate for that particular signature. If an experimental arm has a sufficiently low predictive probability of success for all signatures, it is stopped for futility.

Calculation of Posterior Probabilities

All posterior probabilities are based on all currently available information. The parameters of the pCR-Response Imputation models use Markov Chain Monte Carlo samples; and the prevalence probabilities are sampled directly from its posterior distribution.

Model for Simulating MRI values

To simulate MRI outcomes, we first generate a simulated patients' pCR from the assumed true Bernoulli distribution, and then conditional on the pCR outcome we simulate the tomor size responses from normal distributions with means and variances as observed in I-SPY1.

CONFIDENTIAL

Appendix B
Modified National Comprehensive Cancer Network (NCCN) Guidelines, Dose Modifications, and Management of Standard Therapy Toxicity

Dose Modifications for Standard Therapy: General Considerations

Standard dose reduction for paclitaxel is 25%, which refers to a decrease of 20 mg/m^2; dose reduction for paclitaxel may differ when treatment is given in combination with an investigational agent.
Standard dose reduction for doxorubicin/cyclophosphamide is 20%.

If paclitaxel is held for 3 weeks in a row OR a paclitaxel dose reduction below 60 mg/m^2 is required, stop all protocol therapy. Participant should proceed with additional chemotherapy or surgery at the discretion of the treating physician. Participant will remain on study for outcome assessment.

Missed doses of standard chemotherapy can be made up to complete the full regimen (*e.g.*, 12 cycles of paclitaxel and trastuzumab, if the participant is HER2+, and four cycles of AC). Whenever possible, standard therapy dose and schedule should be maintained.

Please refer to the tables below for standard therapy dose modifications (paclitaxel alone, paclitaxel + trastuzumab, and AC).

For dose modifications of the investigational agent, please see guidelines in each investigational agent's appendix.

Table 1. Paclitaxel Dose Modification

**Event**	**Paclitaxel Dose Modification**
**Neutropenia**	
≥1000/mm^3	No change to paclitaxel.<br>• For ANC ≤1500/mm^3 consider the use of prophylactic myeloid growth factors (filgrastim),<br>o Start on day 2 or 3 and use for 2–6 days according to participant need, at physician discretion, and to avoid dose reduction.<br>o Growth factor should not be given on the same day as chemotherapy.<br>o Pegfilgrastim may not be used with paclitaxel due to the weekly dosing in this study.
<1000/mm^3	Hold paclitaxel until ANC >1000/mm^3. Resume paclitaxel based on timing of recovery:<br>• ≤1 week—no change to paclitaxel<br>• >1 but <3 weeks—reduce paclitaxel dose by 25% for all subsequent cycles<br>• ≥3 weeks—stop paclitaxel. Participant should proceed with additional chemotherapy or surgery at the discretion of the treating physician. Participant remains on study for outcome assessment.
**Neutropenic Fever**	
ANC ≤1000/mm^3, fever ≥38.5°C	Hold paclitaxel until resolved (ANC >1000/mm^3, fever <38.5°C). Resume paclitaxel according to number of episodes:

CONFIDENTIAL

Event	Paclitaxel Dose Modification
	• First episode: no change in paclitaxel<br>• Second episode: 25% dose reduction of paclitaxel for all subsequent cycles<br>• Third episode: stop paclitaxel. Participant should proceed with additional chemotherapy or surgery at the discretion of the treating physician. Participant remains on study for outcome assessment.<br><br>If paclitaxel is held for 3 weeks in a row, stop paclitaxel. Participants should proceed with additional chemotherapy or surgery at the discretion of the treating physician. Participant remains on study for outcome assessment.<br><br>GCSF may be used between days 2–6 according to participant need, at physician discretion, and to avoid dose reduction. Pegfilgrastim may not be used with paclitaxel due to the weekly dosing in this study.
**Thrombocytopenia**	
≥100,000/mm^3	No change to paclitaxel.
75–99,999/mm^3	Hold paclitaxel until ≥100,000/mm^3, resume paclitaxel based on timing of recovery:<br>• ≤1 week—no change to paclitaxel.<br>• >1 but <3 weeks—reduce paclitaxel dose by 25% for all subsequent cycles.<br>• ≥3 weeks—stop paclitaxel. Participant should proceed with additional chemotherapy or surgery at the discretion of the treating physician. Participant remains on study for outcome assessment.
<75,000/mm^3	Hold paclitaxel until ≥100,000/mm^3. Resume paclitaxel with a 25% dose reduction for all subsequent cycles.<br><br>If paclitaxel is held for 3 weeks in a row, stop paclitaxel. Participant should proceed with additional chemotherapy or surgery at the discretion of the treating physician. Participant remains on study for outcome assessment.
**Anemia**	
All grades	No change in paclitaxel.<br><br>For all anemia events related to paclitaxel regardless of grade, iron studies should be checked and iron should be replaced as indicated.<br>• Red blood cell transfusions can be given at the investigators discretion as needed for symptom control.
**Hepatic**	
Grade 0 or 1	No change in paclitaxel.
≤ Grade 2	Grade 2 bilirubin:<br>Hold paclitaxel until bilirubin resolves to ≤ grade 1. Resume paclitaxel based on time of recovery.<br>• If bilirubin resolves to ≤ grade 1 in <2 weeks, resume paclitaxel at previous dose.<br>• If bilirubin remains at grade 2 after holding two consecutive doses of paclitaxel (2 weeks), resume paclitaxel with a 25% reduction in dose for all subsequent doses.<br>• If paclitaxel is held for 3 weeks in a row, stop paclitaxel. Participant should proceed with additional chemotherapy or surgery at the

CONFIDENTIAL

Event	Paclitaxel Dose Modification
	discretion of the treating physician. Participant remains on study for outcome assessment.<br><br>A rise in indirect bilirubin with a normal direct bilirubin believed to be attributable to Gilbert's disease does not require change in dose or agent hold. A note to file should be created.<br><br>Grade 2 AST or ALT:<br>Hold paclitaxel until AST/ALT resolve to ≤ grade 1.<br>• If AST/ALT resolve to ≤ grade 1 in <3 weeks, resume paclitaxel at previous dose.<br>• If paclitaxel is held for 3 weeks in a row, stop paclitaxel. Participant should proceed with additional chemotherapy or surgery at the discretion of the treating physician. Participant remains on study for outcome assessment.
Grade 3	Grade 3 bilirubin (not due to Gilbert's disease):<br>Stop paclitaxel. Participant should proceed with additional chemotherapy or surgery at the discretion of the treating physician. Participant remains on study for outcome assessment.<br><br>Grade 3 AST or ALT:<br>Hold paclitaxel until AST/ALT resolve to ≤ grade 1. Resume paclitaxel at the previous dose.<br>• If AST/ALT remains at grade 3 after holding two consecutive doses of paclitaxel, resume paclitaxel with a 25% dose reduction for all subsequent doses.<br>• If paclitaxel is held for 3 weeks in a row, stop paclitaxel. Participant should proceed with additional chemotherapy or surgery at the discretion of the treating physician. Participant remains on study for outcome assessment.
Grade 4	Grade 4 bilirubin, AST or ALT:<br>Stop paclitaxel. Participant should proceed with additional chemotherapy or surgery at the discretion of the treating physician. Participant remains on study for outcome assessment.
**Nausea/Vomiting**	
Grade 0–2	No change to paclitaxel.
≥Grade 3	Hold paclitaxel until resolved to ≤grade 1.<br>• Resume paclitaxel at previous dose with modification of pre-medications.<br>• For second episode ≥ grade 3 despite maximal supportive care:<br>o Resume paclitaxel with a 25% dose reduction for all subsequent doses.
**Mucositis**	
Grade 0–2	No change to paclitaxel.
≥Grade 3	Hold paclitaxel until resolved to ≤grade 1.<br>• Resume paclitaxel at the previous dose, with modification of premedications.

CONFIDENTIAL

Event	Paclitaxel Dose Modification
	• For second episode ≥ grade 3 despite maximal supportive care:<br>o Resume paclitaxel with a 25% dose reduction for all subsequent cycles.
**Neurotoxicity**	
Grade 0–2	No change to paclitaxel.
Grade 3	Hold paclitaxel until neuropathy improves to ≤ grade 2.<br>• Resume paclitaxel with a 25% dose reduction for all subsequent cycles.<br>If paclitaxel is held for 3 weeks in a row for neuropathy, stop paclitaxel. Participant should proceed with additional chemotherapy or surgery at the discretion of the treating physician. Participant remains on study for outcome assessment.
Grade 4	Stop paclitaxel. Participant should proceed with additional chemotherapy or surgery at the discretion of the treating physician. Participant remains on study for outcome assessment
**Anaphylaxis/Hypersensitivity**	
Mild (*e.g.*, mild flushing, rash, pruritus)	Complete paclitaxel infusion.<br>• No treatment required, but observe participant at least until symptoms have resolved.
Moderate (*e.g.*, moderate flushing, rash, mild dyspnea, chest discomfort)	Stop paclitaxel infusion.<br>• Give intravenous diphenhydramine 20–25 mg and intravenous dexamethasone 10 mg.<br>If symptoms resolve:<br>• Resume paclitaxel infusion after recovery of symptoms at half the previous rate for 15 minutes. If no recurrence of symptoms, the planned rate may be resumed.<br>If symptoms recur after paclitaxel re-challenge:<br>• Stop paclitaxel infusion and stop all subsequent paclitaxel therapy. Participant should proceed with additional chemotherapy or surgery at the discretion of the treating physician. Participant remains on study for outcome assessment.
Severe (*e.g.*, hypotension requiring pressors, angioedema, respiratory distress requiring bronchodilators)	Stop paclitaxel infusion.<br>• Administer diphenhydramine 25 mg and dexamethasone 10 mg IV. Add epinephrine or bronchodilators as needed per institutional guidelines.<br>• Stop all subsequent paclitaxel therapy. Participant should proceed with additional chemotherapy or surgery at the discretion of the treating physician. Participant remains on study for outcome assessment.
**Other Clinically Significant Toxicity Excluding Fatigue, Alopecia, and Leukopenia at Physician Discretion**	
Grade 0 or 1	No change to paclitaxel.
Grade 2	Hold paclitaxel until resolved to ≤ grade 1. Resume paclitaxel at previous dose.<br>• Increase supportive care measures if possible.
≥Grade 3	Hold paclitaxel and contact the DCC for further instruction (1-855-889-5170).<br>If ≥grade 3toxicity recurs,<br>• Stop paclitaxel and contact the DCC for further instruction (1-855-889-5170).

CONFIDENTIAL

Table 2. Paclitaxel and Trastuzumab Dose Modification

Event	Paclitaxel + Trastuzumab Dose Modification
**Neutropenia**	
≥1000/mm³	No change to paclitaxel and trastuzumab.<br><br>• For ANC ≤1500/mm³ consider the use of prophylactic myeloid growth factors (filgrastim),<br>o Start on day 2 or 3 and use for 2–6 days according to participant need, at physician discretion, and to avoid dose reduction.<br>o Growth factor should not be given on the same day as chemotherapy.<br>o Pegfilgrastim may not be used with paclitaxel due to the weekly dosing in this study.
<1000/mm³	Hold paclitaxel until ANC >1000/mm³ but continue trastuzumab. Resume paclitaxel based on timing of recovery:<br>• ≤1 week—no change to paclitaxel and trastuzumab.<br>• >1 but <3 weeks—reduce paclitaxel dose by 25% for all subsequent cycles. No change to trastuzumab<br>• ≥3 weeks—stop paclitaxel and trastuzumab. Participant should proceed with additional chemotherapy or surgery at the discretion of the treating physician. Participant remains on study for outcome assessment.
**Neutropenic Fever**	
ANC ≤1000/mm³, fever ≥38.5°C	Hold paclitaxel until resolved (ANC >1000/mm³, fever <38.5°C) but continue trastuzumab. Resume paclitaxel according to number of episodes:<br>• First episode: no change in paclitaxel and trastuzumab<br>• Second episode: 25% dose reduction of paclitaxel for all subsequent cycles. No change to trastuzumab.<br>• Third episode: stop paclitaxel and trastuzumab. Participant should proceed with additional chemotherapy or surgery at the discretion of the treating physician.<br><br>**If paclitaxel is held for 3 weeks in a row,** stop paclitaxel and trastuzumab. Participants should proceed with additional chemotherapy or surgery at the discretion of the treating physician. Participant remains on study for outcome assessment.<br><br>GCSF may be used between days 2–6 according to participant need, at physician discretion, and to avoid dose reduction. Pegfilgrastim may not be used with paclitaxel due to the weekly dosing in this study.
**Thrombocytopenia**	
≥100,000/mm³	No change to paclitaxel and trastuzumab.
75–99,999/mm³	Hold paclitaxel until ≥100,000/mm³ but continue trastuzumab. Resume paclitaxel based on timing of recovery:<br>• ≤1 week—no change to paclitaxel and trastuzumab.<br>• >1 but <3 weeks—reduce paclitaxel dose by 25% for all subsequent cycles. No change to trastuzumab.

CONFIDENTIAL

Event	Paclitaxel + Trastuzumab Dose Modification
	• ≥3 weeks—stop paclitaxel and trastuzumab. Participant should proceed with additional chemotherapy or surgery at the discretion of the treating physician. Participant remains on study for outcome assessment.
<75,000/mm^3	Hold paclitaxel until ≥100,000/mm^3 but continue trastuzumab.<br>• Resume paclitaxel with a 25% dose reduction for all subsequent cycles. No change to trastuzumab<br><br>If paclitaxel is held for 3 weeks in a row, stop paclitaxel and trastuzumab. Participant should proceed with additional chemotherapy or surgery at the discretion of the treating physician. Participant remains on study for outcome assessment.
**Anemia**	
All grades	No change in paclitaxel and trastuzumab.<br><br>For all anemia events related to paclitaxel regardless of grade, iron studies should be checked and iron should be replaced as indicated.<br><br>• Red blood cell transfusions can be given at the investigators discretion as needed for symptom control.
**Hepatic**	
Grade 0 or 1	No change in paclitaxel and trastuzumab.
≤ Grade 2	Grade 2 bilirubin:<br>Hold paclitaxel until bilirubin resolves to ≤ grade 1 but continue trastuzumab. Resume paclitaxel based on timing of recovery:<br>• If bilirubin resolves to ≤ grade 1 in <2 weeks, resume paclitaxel at previous dose.<br>• If bilirubin remains at grade 2 after holding two consecutive doses of paclitaxel (2 weeks), resume paclitaxel with a 25% reduction in dose for all subsequent doses.<br>• If paclitaxel is held for 3 weeks in a row, stop paclitaxel and trastuzumab. Participant should proceed with additional chemotherapy or surgery at the discretion of the treating physician. Participant remains on study for outcome assessment.<br><br>A rise in indirect bilirubin with a normal direct bilirubin believed to be attributable to Gilbert's disease does not require change in dose or an agent hold. A note to file should be created.<br><br>Grade 2 AST or ALT:<br>Hold paclitaxel until AST/ALT resolve to ≤ grade 1, but continue trastuzumab.<br>• If AST/ALT resolve to ≤ grade 1 in <3 weeks, resume paclitaxel at previous dose. No change to trastuzumab.<br>• If paclitaxel is held for 3 weeks in a row, stop paclitaxel and trastuzumab. Participant should proceed with additional chemotherapy or surgery at the discretion of the treating physician. Participant remains on study for outcome assessment.
Grade 3	Grade 3 bilirubin (not due to Gilbert's disease):<br>Stop paclitaxel and trastuzumab. Participant should proceed with additional chemotherapy or surgery at the discretion of the treating physician. Participant remains on study for outcome assessment.<br><br>Grade 3 AST or ALT:

CONFIDENTIAL

Event	Paclitaxel + Trastuzumab Dose Modification
	Hold paclitaxel until AST/ALT resolve to ≤ grade 1but continue trastuzumab. Resume paclitaxel at the previous dose.<br>• If AST/ALT remains at grade 3 after holding two consecutive doses (2 weeks) of paclitaxel, resume paclitaxel with a 25% dose reduction for all subsequent doses. No change to trastuzumab<br>• If paclitaxel is held for 3 weeks in a row, stop paclitaxel and trastuzumab. Participant should proceed with additional chemotherapy or surgery at the discretion of the treating physician. Participant remains on study for outcome assessment.
Grade 4	Grade 4 bilirubin, AST or ALT:<br>Stop paclitaxel and trastuzumab. Participant should proceed with additional chemotherapy or surgery at the discretion of the treating physician. Participant remains on study for outcome assessment.
**Nausea/Vomiting**	
Grade 0–2	No change to paclitaxel and trastuzumab.
≥ Grade 3	Hold paclitaxel until resolved to ≤grade 1 but continue trastuzumab.<br>• For first episode, resume paclitaxel at previous dose with modification of premedications. No change to trastuzumab.<br>• For second episode of ≥grade 3 despite maximal supportive care:<br>o Resume paclitaxel with a 25% dose reduction for all subsequent doses. No change to trastuzumab.
**Mucositis**	
Grade 0–2	No change to paclitaxel and trastuzumab.
≥ Grade 3	Hold paclitaxel until resolved to ≤grade 1 but continue trastuzumab.<br>• For first episode, resume paclitaxel at the previous dose, with modification of pre-medications. No change to trastuzumab.<br>• For second episode ≥grade 3 despite maximal supportive care,<br>o Resume paclitaxel with a 25% dose reduction for all subsequent cycles. No change to trastuzumab.
**Neurotoxicity**	
Grade 0–2	No change in paclitaxel and trastuzumab.
Grade 3	Hold paclitaxel until neuropathy improves to ≤grade 2 but continue trastuzumab.<br>• Resume paclitaxel with a 25% dose reduction for all subsequent cycles. No change to trastuzumab.<br>If paclitaxel is held for 3 weeks in a row for neuropathy, stop paclitaxel and trastuzumab. Participant should proceed with additional chemotherapy or surgery at the discretion of the treating physician. Participant remains on study for outcome assessment.
Grade 4	Stop paclitaxel and trastuzumab. Participant should proceed with additional chemotherapy or surgery at the discretion of the treating physician. Participant remains on study for outcome assessment
**Cardiac**	
Asymptomatic decline in LVEF<br>LVEF has declined 16 or more percentage points from baseline	Stop trastuzumab for 4 weeks but continue paclitaxel.<br>• Reassess LVEF at 4 weeks:

CONFIDENTIAL

Event	Paclitaxel + Trastuzumab Dose Modification
OR<br>LVEF has declined 10 to 15 percentage points from baseline AND is below the lower limit of normal	o If LVEF has recovered to baseline or the absolute decrease from baseline is <15 percentage points, resume trastuzumab at previous dose.<br>o If LVEF has not either recovered to baseline or the absolute decrease from baseline is >15 percentage points, stop trastuzumab and paclitaxel therapy. Participant should proceed with additional chemotherapy or surgery at the discretion of the treating physician. Participant remains on study for outcome assessment of the treating physician. Participant remains on study for outcome assessment.
Symptomatic cardiac dysfunction	Stop trastuzumab and paclitaxel therapy. Participant should proceed with additional chemotherapy or surgery at the discretion of the treating physician. Participant remains on study for outcome assessment.
**Anaphylaxis/Hypersensitivity**	
Mild (*e.g.*, mild flushing, rash, pruritus)	Complete paclitaxel infusion.<br>• No treatment required, but observe participant at least until symptoms have resolved.
Moderate (*e.g.*, moderate flushing, rash, mild dyspnea, chest discomfort)	For paclitaxel:<br>• Stop paclitaxel infusion.<br>• Give intravenous diphenhydramine 20–25 mg and intravenous dexamethasone 10 mg.<br>• Resume paclitaxel infusion after recovery of symptoms at half the previous rate for 15 minutes. If no recurrence of symptoms, the planned rate may be resumed.<br><br>For trastuzumab:<br>• Stop trastuzumab infusion.<br>• Treat symptomatically according to institutional guidelines.<br>• Resume trastuzumab infusion at a slower rate according to institutional guidelines after reaction has resolved. If no recurrence of symptoms, the planned rate may be resumed.<br><br>If symptoms recur after paclitaxel or trastuzumab re-challenge:<br>• Stop paclitaxel or trastuzumab infusion and stop all subsequent paclitaxel and trastuzumab therapy. Participant should proceed with additional chemotherapy or surgery at the discretion of the treating physician. Participant remains on study for outcome assessment.
Severe (*e.g.*, hypotension requiring pressors, angioedema, respiratory distress requiring bronchodilators)	Stop paclitaxel or trastuzumab.<br>• Administer diphenhydramine 25 mg and dexamethasone 10 mg IV. Add epinephrine or bronchodilators as needed per institutional guidelines.<br>• Stop all subsequent paclitaxel and trastuzumab therapy. Participant should proceed with additional chemotherapy or surgery at the discretion of the treating physician. Participant remains on study for outcome assessment.
**Other Clinically Significant Toxicity Excluding Fatigue, Alopecia, and Leukopenia at Physician Discretion**	
Grade 0 or 1	No change to paclitaxel and trastuzumab.
Grade 2	Hold paclitaxel until resolved to ≤ grade 1 but continue trastuzumab. Resume paclitaxel at previous dose.

CONFIDENTIAL

Event	Paclitaxel + Trastuzumab Dose Modification
	• Increase supportive care measures if possible.
≥ Grade 3	Hold paclitaxel and contact the DCC for further instruction (1-855-889-5170).<br><br>If toxicity deemed related to trastuzumab, at physician discretion:<br>• Hold trastuzumab.<br>• Contact the DCC for further instructions (1-855-889-5170).

Table 3. Doxorubicin/Cyclophosphamide (AC) Dose Modification

Event	Doxorubicin/Cyclophosphamide (AC) Dose Modification
**Neutropenia**	
≥1000/mm^3	No change to AC.
<1000/mm^3	Hold until ANC >1000/mm^3, resume AC based on timing of recovery:<br>• ≤1 week—no change to AC.<br>• >1 but <3 weeks—reduce AC dose by 20% for subsequent cycles.<br>• ≥3 weeks—stop AC. Participant should proceed with additional chemotherapy or surgery at the discretion of the treating physician. Participant remains on study for outcome assessment.
**Neutropenic Fever**	
ANC ≤1000/mm^3, fever ≥ 38.5°C	Hold until resolved (ANC >1000/mm^3, fever <38.5°C), resume according to number of episodes:<br>• First episode: no change to AC.<br>• Second episode: reduce AC dose by 20% for all subsequent cycles.<br>• Third episode: stop AC. Participant should proceed with additional chemotherapy or surgery at the discretion of the treating physician. Participant remains on study for outcome assessment.
**Thrombocytopenia**	
≥ 100,000/mm^3	No change to AC.
75–99,999/mm^3	Hold until ≥ 100,000/mm^3, resume based on timing of recovery:<br>• ≤1 week—no change to AC.<br>• >1 week but <3 weeks—reduce AC dose by 20% for all subsequent cycles.<br>• ≥3 weeks —stop AC. Participant should proceed with additional chemotherapy or surgery at the discretion of the treating physician. Participant remains on study for outcome assessment
<75,000/mm^3	Hold until ≥ 100,000/mm^3.<br>• Resume AC a with 20% dose reduction for all subsequent cycles.<br><br>If AC is held for 3 weeks in a row, stop AC. Participant should proceed with additional chemotherapy or surgery at the discretion of the treating physician. Participant remains on study for outcome assessment.
**Anemia**	
All grades	No change to AC.
**Hepatic**	
Grade 0 or 1	No change to AC.

CONFIDENTIAL

Event	Doxorubicin/Cyclophosphamide (AC) Dose Modification
≥ Grade 2	Hold AC until ≤ grade 1.<br>• Resume AC at previous dose.<br><br>If AC is held for 3 weeks in a row, <u>stop AC</u>. Participant should proceed with additional chemotherapy or surgery at the discretion of the treating physician. Participant remains on study for outcome assessment.
**Nausea/Vomiting**	
Grade 0–2	No change to AC.
≥ Grade 3	Hold AC until resolved to ≤ grade 1.<br>• Resume AC with a 20% dose reduction for all subsequent cycles.
**Mucositis**	
Grade 0–2	No change to AC.
≥ Grade 3	Hold AC until resolved to ≤ grade 1.<br>• Resume AC with a 20% dose reduction for all subsequent cycles.
**Cardiac**	
Grade 0–2	No change to AC.
≥ Grade 3	Discontinue AC if:<br>• A participant has symptoms of CHF and a diagnosis of CHF is confirmed;<br>• A participant has a myocardial infarction;<br>• 15% absolute decline in LVEF from baseline or >10% decline in LVEF from baseline to below LLN.<br>For any other cardiac toxicity > grade 3, hold AC and contact DCC for instructions (1-855-889-5170).<br><br>**NOTE:** PACs or PVCs without cardiac dysfunction (*e.g.,* acute dysrhythmias) during and shortly after doxorubicin infusion are NOT an indication to permanently stop doxorubicin.<br><br>Participant should proceed with additional chemotherapy or surgery at the discretion of the treating physician. Participant remains on study for outcome assessment.
**Other Clinically Significant Toxicity Excluding Fatigue, Alopecia, and Leukopenia at Physician Discretion**	
Grade 0 or 1	No change to AC.
Grade 2	Hold AC until resolved to ≤ grade 1. Resume AC at previous dose.<br>• Increase supportive care measures, if possible.
≥ Grade 3	Hold AC and contact DCC for instructions (1-855-889-5170).<br><br>If ≥ grade 3 toxicity recurs, <u>stop AC</u>. Participant should proceed with additional chemotherapy or surgery at the discretion of the treating physician. Participant remains on study for outcome assessment.

Abbreviations: AC: anthracycline (doxorubicin + cyclophosphamide); ANC: absolute neutrophil count; CHF: congestive heart failure; LLN: lower limit of normal; LVEF: left ventricular ejection fraction; PAC: premature atrial complex; PVC: premature ventricular complex.

<u>Toxicity Management of Paclitaxel, Paclitaxel + Trastuzumab, Doxorubicin/Cyclophosphamide</u>

See attached NCCN guidelines and Sparano *et al.,* N Engl J Med. 358: 1663–1671, 2008.

CONFIDENTIAL

National Comprehensive Cancer Network®

BRS5a

Chemotherapy Order Template™

Breast Cancer

AC (DOXOrubicin/Cyclophosphamide) Every 21 Days → PACLItaxel Every 21 Days

AC (DOXOrubicin/Cyclophosphamide) Every 21 Days Course

INDICATION:	REFERENCES:	NCCN SUPPORTIVE CARE:
Adjuvant	1. NCCN Clinical Practice Guidelines in Oncology™ Breast Cancer. V.2.2008.<br>2. Mamounas EP, et al. *J Clin Oncol.* 2005;23(16):3686-96.[a]	1. *Emetic Risk:* Day 1 High<br>2. *Fever Neutropenia Risk:* Intermediate

CHEMOTHERAPY REGIMEN

21-day cycle for 4 cycles

- **DOXOrubicin** 60 mg/m^2 IV Push on Day 1
- **Cyclophosphamide** 600 mg/m^2 IV over 30 minutes on Day 1
- Oral hydration is strongly encouraged with cyclophosphamide; poorly hydrated patients may need supplemental IV hydration. Patients should attain combined oral and IV hydration of 2 – 3 L/day on day of chemotherapy.
  See example of recommended supplemental IV hydration below.

This course is 4 cycles of AC (DOXOrubicin/cyclophosphamide) Every 21 Days.
PACLItaxel Every 21 Days is initiated following completion of this course.
Please see Order Template BRS5b for PACLItaxel Every 21 Days course.

SUPPORTIVE CARE

Antiemetic therapy (See www.nccn.org/professionals/physician_gls/PDF/antiemesis.pdf)

- Aprepitant 125 mg PO or fosaprepitant 115 IV Day 1, aprepitant 80 mg PO Days 2 – 3
  AND
- Dexamethasone 12 mg PO/IV Day 1, then 8 mg PO/IV Days 2 – 4
  AND
- 5-HT3 antagonist:
  Ondansetron 16 – 24 mg PO or 8 – 12 mg (maximum 32 mg/day) IV Day 1
  OR
  Granisetron 2 mg PO daily or 1 mg PO BID or 0.01 mg/kg (maximum 1 mg) IV daily Day 1
  OR
  Dolasetron 100 mg PO or 1.8 mg/kg IV or 100 mg IV Day 1
  OR
  Palonosetron 0.25 mg IV Day 1
  AND
- ± Lorazepam 0.5 – 2 mg PO/IV or sublingual every 4 or every 6 hours Days 1 – 4

PRN for breakthrough: Patients should be given at least one medication in a different category than that given above to have as needed for breakthrough. Please consult the NCCN Clinical Practice Guidelines in Oncology™ Antiemesis for appropriate antiemetic therapy.

Template continued on page 2

This template is a peer-reviewed statement of the consensus of its authors derived from the NCCN Clinical Practice Guidelines in Oncology™ regarding their views of currently accepted approaches to treatment. This template does not constitute an order. Any clinician seeking to treat a patient using this template is expected to use independent medical judgment in the context of individual clinical circumstances of a specific patient's care or treatment. NCCN disclaims all warranties, express or implied including, without limitation, the implied warranties of merchantability and fitness for a particular purpose. NCCN does not warrant the accuracy, currency, or completeness of the template or make any representation regarding the use or the results of the use of the template in treatment. In no event shall NCCN or its members be liable for any damages including, without limitation, incidental, indirect, special, punitive, or consequential damages arising out of or in connection with the use of this template including, without limitation, loss of life, loss of data, loss of income or profit, losses sustained as a result of any injury to any person, or loss or damage to property or claims of third parties.

© 2008 National Comprehensive Cancer Network

Chemotherapy Order Template™

Breast Cancer

AC (DOXOrubicin/Cyclophosphamide) Every 21 Days → PACLItaxel Every 21 Days

AC (DOXOrubicin/Cyclophosphamide) Every 21 Days Course

Myeloid growth factor therapy (see www.nccn.org/professionals/physician_gls/PDF/myeloid_growth.pdf)

CSFs not generally recommended as primary prophylaxis based on FN risk of chemotherapy regimen. For more information on prophylaxis of FN, refer to NCCN Clinical Practice Guidelines in Oncology™ Myeloid Growth Factors and Appendix C to the NCCN Chemotherapy Order Templates

Other Supportive Therapy

- For cyclophosphamide: *Example of recommended supplemental IV hydration*: Sodium chloride 0.9% infused IV at a rate of 1.5 – 3 mL/kg/hour for a total of 500 mL on day of chemotherapy.

MONITORING AND HOLD PARAMETERS

- CBC with differential should be assessed routinely for potential dose evaluation.
- For DOXOrubicin:
  - DOXOrubicin is an anthracycline. Cumulative anthracycline dosage should be monitored.
  - Ejection fraction should be assessed prior to initiation of anthracycline treatment and as clinically indicated.
  - Liver function should be assessed prior to each cycle for potential dose evaluation.
- For cyclophosphamide: Renal function should be assessed prior to each cycle for potential dose evaluation.

SAFETY PARAMETERS AND SPECIAL INSTRUCTIONS

- For DOXOrubicin: **DOXOrubicin is a vesicant.**
- For aprepitant and fosaprepitant: Refer to Appendix D for specific information regarding associated drug interactions.

This template is a peer-reviewed statement of the consensus of its authors derived from the NCCN Clinical Practice Guidelines in Oncology™ regarding their views of currently accepted approaches to treatment. This template does not constitute an order. Any clinician seeking to treat a patient using this template is expected to use independent medical judgment in the context of individual clinical circumstances of a specific patient's care or treatment. NCCN disclaims all warranties, express or implied including, without limitation, the implied warranties of merchantability and fitness for a particular purpose. NCCN does not warrant the accuracy, currency, or completeness of the template or make any representation regarding the use or the results of the use of the template in treatment. In no event shall NCCN or its members be liable for any damages including, without limitation, incidental, indirect, special, punitive, or consequential damages arising out of or in connection with the use of this template including, without limitation, loss of life, loss of data, loss of income or profit, losses sustained as a result of any injury to any person, or loss or damage to property or claims of third parties.

© 2008 National Comprehensive Cancer Network

National Comprehensive Cancer Network®

BRS13a

Chemotherapy Order Template™

Breast Cancer

Dose-Dense AC (DOXOrubicin/Cyclophosphamide) →Dose-Dense PACLItaxel

Dose-Dense AC (DOXOrubicin/ Cyclophosphamide) Course

INDICATION:	REFERENCES:	NCCN SUPPORTIVE CARE:
Adjuvant	1. NCCN Clinical Practice Guidelines in Oncology™ Breast Cancer. V.1.2009.<br>2. Citron ML, et al. *J Clin Oncol.* 2003; 21(8):1431-9.[a]	1. *Emetic Risk:* Day 1 High<br>2. *Fever Neutropenia Risk:* High

CHEMOTHERAPY REGIMEN

14-day cycle for 4 cycles

- **DOXOrubicin** 60 mg/m^2 IV Push on Day 1
- See *Safety Parameters and Special Instructions* for information on slow IV Push administration.
- **Cyclophosphamide** 600 mg/m^2 IV over 30 minutes on Day 1
- Oral hydration is strongly encouraged with cyclophosphamide; poorly hydrated patients may need supplemental IV hydration. Patients should attain combined oral and IV hydration of 2 – 3 L/day on day of chemotherapy. See *Other Supportive Therapy* for example of recommended hydration.

This course is 4 cycles of dose-dense AC (DOXOrubicin/cyclophosphamide). Dose-dense PACLItaxel is initiated following completion of this course. Please see Order Template BRS13b for dose-dense PACLItaxel course.

SUPPORTIVE CARE

Antiemetic therapy (See www.nccn.org/professionals/physician_gls/PDF/antiemesis.pdf)

Days 1 – 4

- Aprepitant 125 mg PO or fosaprepitant 115 mg IV Day 1, aprepitant 80 mg PO Days 2 – 3
  AND
- Dexamethasone 12 mg PO/IV Days 1 – 4
  AND
- 5-HT3 antagonist (recommended on days of highly emetogenic chemotherapy administration) :
  Palonosetron 0.25 mg IV Day 1
  OR
  Dolasetron 100 mg PO or 1.8 mg/kg IV or 100 mg IV Day 1
  OR
  Granisetron 2 mg PO daily or 1 mg PO BID or 0.01 mg/kg (maximum 1 mg) IV daily Day 1 or transdermal patch containing 34.3 mg granisetron applied 24 – 48 hours prior to first dose of chemotherapy (patch supplies 5 days of therapeutic drug starting 24 hours after application)
  OR
  Ondansetron 16 – 24 mg PO or 8 – 12 mg (maximum 32 mg/day) IV Day 1
  AND
- ± Lorazepam 0.5 – 2 mg PO/IV or sublingual every 4 or every 6 hours as needed Days 1 – 4
  AND
- ± H_2 blocker or proton pump inhibitor

Template continued on page 2

This template is a peer-reviewed statement of the consensus of its authors derived from the NCCN Clinical Practice Guidelines in Oncology™ regarding their views of currently accepted approaches to treatment. This template does not constitute an order. Any clinician seeking to treat a patient using this template is expected to use independent medical judgment in the context of individual clinical circumstances of a specific patient's care or treatment. NCCN disclaims all warranties, express or implied including, without limitation, the implied warranties of merchantability and fitness for a particular purpose. NCCN does not warrant the accuracy, currency, or completeness of the template or make any representation regarding the use or the results of the use of the template in treatment. In no event shall NCCN or its members be liable for any damages including, without limitation, incidental, indirect, special, punitive, or consequential damages arising out of or in connection with the use of this template including, without limitation, loss of life, loss of data, loss of income or profit, losses sustained as a result of any injury to any person, or loss or damage to property or claims of third parties.

© 2009 National Comprehensive Cancer Network 07/09/2009

Breast Cancer

Dose-Dense AC (DOXOrubicin/Cyclophosphamide) →Dose-Dense PACLItaxel

Dose-Dense AC (DOXOrubicin/ Cyclophosphamide) Course

PRN for breakthrough: Patients should be given at least one medication in a different category than that given above to have as needed for breakthrough. Please consult the NCCN Clinical Practice Guidelines in Oncology™ Antiemesis for appropriate antiemetic therapy.

Myeloid growth factor therapy (See www.nccn.org/professionals/physician_gls/PDF/myeloid_growth.pdf)

- **Filgrastim (Category 1*)**
  5 mcg/kg/day subcutaneously daily recommended to start 24 – 72 hours after completion of chemotherapy and to continue until post-nadir ANC recovery to normal or near-normal levels by laboratory standards. Dose is rounded to the nearest vial size by institution-defined weight limits. Same-day administration is not recommended.
  OR
- **Pegfilgrastim (Category 1*)**
  6 mg subcutaneously recommended to be given 24 – 72 hours after completion of chemotherapy for one dose only. There are insufficient data to support dose and schedule of weekly regimens or schedules less than 2 weeks and these cannot be recommended. Same-day administration is not recommended.
  OR
- **Sargramostim (Category 2B*)**
  250 mcg/m^2/day subcutaneously daily recommended to start 24– 72 hours after completion of chemotherapy and to continue until post-nadir ANC recovery to normal or near-normal levels by laboratory standards. Dose is rounded to the nearest vial size by institution-defined weight limits. Same-day administration is not recommended.

Other Supportive Therapy

- For cyclophosphamide: *Example of recommended hydration*: Sodium chloride 0.9% infused IV at a rate of 1.5 – 3 mL/kg/hour for a total of 500 mL on day of chemotherapy.

MONITORING AND HOLD PARAMETERS

- CBC with differential should be assessed routinely for potential dose evaluation.
- For DOXOrubicin:
  - DOXOrubicin is an anthracycline. Cumulative anthracycline dosage should be monitored.
  - Ejection fraction should be assessed prior to initiation of treatment and as clinically indicated.
  - Liver function should be assessed routinely for potential dose evaluation.
- For cyclophosphamide: Renal function should be assessed prior to each cycle for potential dose evaluation.

Template continued on page 3

This template is a peer-reviewed statement of the consensus of its authors derived from the NCCN Clinical Practice Guidelines in Oncology™ regarding their views of currently accepted approaches to treatment. This template does not constitute an order. Any clinician seeking to treat a patient using this template is expected to use independent medical judgment in the context of individual clinical circumstances of a specific patient's care or treatment. NCCN disclaims all warranties, express or implied including, without limitation, the implied warranties of merchantability and fitness for a particular purpose. NCCN does not warrant the accuracy, currency, or completeness of the template or make any representation regarding the use or the results of the use of the template in treatment. In no event shall NCCN or its members be liable for any damages including, without limitation, incidental, indirect, special, punitive, or consequential damages arising out of or in connection with the use of this template including, without limitation, loss of life, loss of data, loss of income or profit, losses sustained as a result of any injury to any person, or loss or damage to property or claims of third parties.

© 2009 National Comprehensive Cancer Network 07/09/2009

Chemotherapy Order Template™

Breast Cancer

Dose-Dense AC (DOXOrubicin/Cyclophosphamide) →Dose-Dense PACLItaxel

Dose-Dense AC (DOXOrubicin/ Cyclophosphamide) Course

SAFETY PARAMETERS AND SPECIAL INSTRUCTIONS

- For DOXOrubicin:
  - **DOXOrubicin is a vesicant.**
  - This agent is administered IV Push. The preferred IV Push method for a vesicant is administration through the side port of a freely flowing IV; alternatively, the drug can be administered via direct IV push.
- For aprepitant and fosaprepitant: Refer to Appendix D for specific information regarding associated drug interactions.

*The NCCN Guidelines Steering Committee has devised a set of Categories of Consensus. These annotations contain two dimensions: the strength of the evidence behind the recommendation and the degree of consensus about its inclusion.

- **Category 1:** There is uniform NCCN consensus, based on high-level evidence, that the recommendation is appropriate.
- **Category 2A:** There is uniform NCCN consensus, based on lower-level evidence including clinical experience, that the recommendation is appropriate.
- **Category 2B**: There is nonuniform NCCN consensus (but no major disagreement), based on lower-level evidence including clinical experience, that the recommendation is appropriate.
- **Category 3:** There is major NCCN disagreement, regardless of the level of evidence, that the recommendation is appropriate.

This template is a peer-reviewed statement of the consensus of its authors derived from the NCCN Clinical Practice Guidelines in Oncology™ regarding their views of currently accepted approaches to treatment. This template does not constitute an order. Any clinician seeking to treat a patient using this template is expected to use independent medical judgment in the context of individual clinical circumstances of a specific patient's care or treatment. NCCN disclaims all warranties, express or implied including, without limitation, the implied warranties of merchantability and fitness for a particular purpose. NCCN does not warrant the accuracy, currency, or completeness of the template or make any representation regarding the use or the results of the use of the template in treatment. In no event shall NCCN or its members be liable for any damages including, without limitation, incidental, indirect, special, punitive, or consequential damages arising out of or in connection with the use of this template including, without limitation, loss of life, loss of data, loss of income or profit, losses sustained as a result of any injury to any person, or loss or damage to property or claims of third parties.

© 2009 National Comprehensive Cancer Network

07/09/2009

National Comprehensive Cancer Network®

NCCN

BRS16a

Chemotherapy Order Template™

Breast Cancer

AC (DOXOrubicin/Cyclophosphamide) Every 21 Days → T (PACLItaxel) Every 21 Days + Trastuzumab

AC (DOXOrubicin/Cyclophosphamide) Every 21 Days Course

INDICATION:	REFERENCES:	NCCN SUPPORTIVE CARE:
Adjuvant	1. NCCN Clinical Practice Guidelines in Oncology™ Breast Cancer. V.2.2008.<br>2. Romond EH, et al. *N Engl J Med.* 2005;353(16):1673-84.[a]	1. *Emetic Risk:* Day 1 High<br>2. *Fever Neutropenia Risk:* Intermediate

CHEMOTHERAPY REGIMEN

21-day cycle for 4 cycles

- **DOXOrubicin** 60 mg/m^2 IV Push on Day 1
- **Cyclophosphamide** 600 mg/m^2 IV over 30 minutes on Day 1
- Oral hydration is strongly encouraged with cyclophosphamide; poorly hydrated patients may need supplemental IV hydration. Patients should attain combined oral and IV hydration of 2 – 3 L/day on day of chemotherapy.
  See example of recommended supplemental IV hydration below.

This course is 4 cycles of AC (DOXOrubicin/cyclophosphamide) Every 21 Days.
T (PACLItaxel) Every 21 Days and trastuzumab course is initiated following completion of this course.
Please see Order Template BRS16b for T (PACLItaxel) Every 21 Days + trastuzumab course.

SUPPORTIVE CARE

Antiemetic therapy (See www.nccn.org/professionals/physician_gls/PDF/antiemesis.pdf)

- Aprepitant 125 mg PO or fosaprepitant 115 mg IV Day 1, aprepitant 80 mg PO Days 2 – 3
  AND
- Dexamethasone 12 mg PO/IV Day 1, then 8 mg PO/IV Days 2 – 4
  AND
- 5HT3 antagonist:
  Ondansetron 16 - 24 mg PO or 8 – 12 mg (maximum 32 mg/day) IV Day 1
  OR
  Granisetron 2 mg PO daily or 1 mg PO BID or 0.01 mg/kg (maximum 1 mg) IV daily Day 1
  OR
  Dolasetron 100 mg PO or 1.8 mg/kg IV or 100 mg IV Day 1
  OR
  Palonosetron 0.25 mg IV Day 1
  AND
- ± Lorazepam 0.5 - 2 mg PO/IV or sublingual every 4 or every 6 hours Days 1 – 4

PRN for breakthrough: Patients should be given at least one medication in a different category than that given above to have as needed for breakthrough. Please consult the NCCN Clinical Practice Guidelines in Oncology™ Antiemesis for appropriate antiemetic therapy.

Template continued on page 2

This template is a peer-reviewed statement of the consensus of its authors derived from the NCCN Clinical Practice Guidelines in Oncology™ regarding their views of currently accepted approaches to treatment. This template does not constitute an order. Any clinician seeking to treat a patient using this template is expected to use independent medical judgment in the context of individual clinical circumstances of a specific patient's care or treatment. NCCN disclaims all warranties, express or implied including, without limitation, the implied warranties of merchantability and fitness for a particular purpose. NCCN does not warrant the accuracy, currency, or completeness of the template or make any representation regarding the use or the results of the use of the template in treatment. In no event shall NCCN or its members be liable for any damages including, without limitation, incidental, indirect, special, punitive, or consequential damages arising out of or in connection with the use of this template including, without limitation, loss of life, loss of data, loss of income or profit, losses sustained as a result of any injury to any person, or loss or damage to property or claims of third parties.

© 2008 National Comprehensive Cancer Network

Chemotherapy Order Template™

Breast Cancer

AC (DOXOrubicin/Cyclophosphamide) Every 21 Days → T (PACLItaxel) Every 21 Days + Trastuzumab

AC (DOXOrubicin/Cyclophosphamide) Every 21 Days Course

Myeloid growth factor therapy (See www.nccn.org/professionals/physician_gls/PDF/myeloid_growth.pdf)

CSFs not generally recommended for primary prophylaxis based on FN risk of chemotherapy regimen. For more information on prophylaxis of FN, refer to NCCN Clinical Practice Guidelines in Oncology™ Myeloid Growth Factors and Appendix C to the NCCN Chemotherapy Order Templates.

Other Supportive Therapy

- For cyclophosphamide: *Example of recommended supplemental IV hydration*: Sodium chloride 0.9% infused IV at a rate of 1.5 – 3 mL/kg/hour for a total of 500 mL on day of chemotherapy.

MONITORING AND HOLD PARAMETERS

- CBC with differential should be assessed routinely for potential dose evaluation.
- For DOXOrubicin:
  - o DOXOrubicin is an anthracycline. Cumulative anthracycline dosage should be monitored.
  - o Ejection fraction should be assessed prior to initiation of treatment and as clinically indicated.
  - o Liver function should be assessed prior to each cycle for potential dose evaluation.
- For cyclophosphamide: Renal function should be assessed prior to each cycle for potential dose modification.

SAFETY PARAMETERS AND SPECIAL INSTRUCTIONS

- For DOXOrubicin: **DOXOrubicin is a vesicant.**
- For aprepitant and fosaprepitant: Refer to Appendix D for specific information regarding associated drug interactions.

This template is a peer-reviewed statement of the consensus of its authors derived from the NCCN Clinical Practice Guidelines in Oncology™ regarding their views of currently accepted approaches to treatment. This template does not constitute an order. Any clinician seeking to treat a patient using this template is expected to use independent medical judgment in the context of individual clinical circumstances of a specific patient's care or treatment. NCCN disclaims all warranties, express or implied including, without limitation, the implied warranties of merchantability and fitness for a particular purpose. NCCN does not warrant the accuracy, currency, or completeness of the template or make any representation regarding the use or the results of the use of the template in treatment. In no event shall NCCN or its members be liable for any damages including, without limitation, incidental, indirect, special, punitive, or consequential damages arising out of or in connection with the use of this template including, without limitation, loss of life, loss of data, loss of income or profit, losses sustained as a result of any injury to any person, or loss or damage to property or claims of third parties.

© 2008 National Comprehensive Cancer Network

National Comprehensive Cancer Network® NCCN

Chemotherapy Order Template™
Breast Cancer
AC (DOXOrubicin/Cyclophosphamide) Every 21 Days → PACLItaxel Every 21 Days

PACLItaxel Every 21 Days Course

INDICATION:	REFERENCES:	NCCN SUPPORTIVE CARE:
Adjuvant	1. NCCN Clinical Practice Guidelines in Oncology™ Breast Cancer. V.2.2008.<br>2. Mamounas EP, et al. *J Clin Oncol.* 2005;23(16):3686-96.[d]	1. *Emetic Risk:* Day 1 Low<br>2. *Fever Neutropenia Risk:* Refer to NCCN Clinical Practice Guidelines in Oncology™ Myeloid Growth Factors. V.1.2008.

CHEMOTHERAPY REGIMEN
21-day cycle for 4 cycles

- **PACLItaxel** 175 – 225 mg/m^2 IV over 3 hours on Day 1

This course is 4 cycles of PACLItaxel Every 21 Days.
This course is initiated following completion of the AC (DOXOrubicin/ cyclophosphamide) Every 21 Days course.
Please see Order Template BRS5a for the AC (DOXOrubicin/cyclophosphamide) Every 21 Days course.

SUPPORTIVE CARE

Premedications

PACLItaxel requires premedication for hypersensitivity:

- **H2 antagonist:**
  Famotidine 20 mg IV/PO 30 – 60 minutes pre-PACLItaxel
  OR
  Ranitidine 50 mg IV or 150 mg PO 30 – 60 minutes pre-PACLItaxel
  OR
  Cimetidine 300 mg IV/PO 30 – 60 minutes pre-PACLItaxel
  AND
- **H1 antagonist:**
  Diphenhydramine 12.5 – 50 mg IV/PO 30 – 60 minutes pre-PACLItaxel
  AND
- **Dexamethasone:**
  Dexamethasone 20 mg PO approximately 12 and 6 hours pre-PACLItaxel
  OR
  Dexamethasone 20 mg IV 30 minutes pre-PACLItaxel

Template continued on page 2

This template is a peer-reviewed statement of the consensus of its authors derived from the NCCN Clinical Practice Guidelines in Oncology™ regarding their views of currently accepted approaches to treatment. This template does not constitute an order. Any clinician seeking to treat a patient using this template is expected to use independent medical judgment in the context of individual clinical circumstances of a specific patient's care or treatment. NCCN disclaims all warranties, express or implied including, without limitation, the implied warranties of merchantability and fitness for a particular purpose. NCCN does not warrant the accuracy, currency, or completeness of the template or make any representation regarding the use or the results of the use of the template in treatment. In no event shall NCCN or its members be liable for any damages including, without limitation, incidental, indirect, special, punitive, or consequential damages arising out of or in connection with the use of this template including, without limitation, loss of life, loss of data, loss of income or profit, losses sustained as a result of any injury to any person, or loss or damage to property or claims of third parties.

© 2008 National Comprehensive Cancer Network

Chemotherapy Order Template™

Breast Cancer

AC (DOXOrubicin/Cyclophosphamide) Every 21 Days → PACLItaxel Every 21 Days

PACLItaxel Every 21 Days Course

Antiemetic therapy (See www.nccn.org/professionals/physician_gls/PDF/antiemesis.pdf)

Day 1
No additional dexamethasone needed on Day 1 if dexamethasone already given for hypersensitivity.

- Dexamethasone 12 mg PO/IV Day 1
  OR
- Prochlorperazine 10 mg PO/IV every 4 or every 6 hours Day 1
  OR
- Metoclopramide 10 – 40 mg PO/IV every 4 or every 6 hours ± Diphenhydramine 25 – 50 mg PO/IV every 4 or every 6 hours Day 1
  AND
- ± Lorazepam 0.5 – 2 mg PO/IV every 4 or every 6 hours Day 1

PRN for breakthrough: Patients should be given at least one medication in a different category than that given above to have as needed for breakthrough. Please consult the NCCN Clinical Practice Guidelines in Oncology™ Antiemesis for appropriate antiemetic therapy.

MONITORING AND HOLD PARAMETERS

- CBC with differential should be assessed routinely for potential dose evaluation.
- For PACLItaxel:
  - Liver function should be assessed prior to each cycle for dose potential evaluation.
  - Hypersensitivity reaction may occur with infusion. Monitor for and treat hypersensitivity reactions as per institutional standard.
  - Signs and symptoms of neurotoxicity should be assessed prior to each cycle. Modifications of chemotherapy may be warranted.

SAFETY PARAMETERS AND SPECIAL INSTRUCTIONS

- For PACLItaxel:
  - PACLItaxel is an irritant.
  - PACLItaxel should be prepared either in glass or non-PVC containers and administered through non-PVC tubing and an in-line filter of not greater than 0.22 microns.

This template is a peer-reviewed statement of the consensus of its authors derived from the NCCN Clinical Practice Guidelines in Oncology™ regarding their views of currently accepted approaches to treatment. This template does not constitute an order. Any clinician seeking to treat a patient using this template is expected to use independent medical judgment in the context of individual clinical circumstances of a specific patient's care or treatment. NCCN disclaims all warranties, express or implied including, without limitation, the implied warranties of merchantability and fitness for a particular purpose. NCCN does not warrant the accuracy, currency, or completeness of the template or make any representation regarding the use or the results of the use of the template in treatment. In no event shall NCCN or its members be liable for any damages including, without limitation, incidental, indirect, special, punitive, or consequential damages arising out of or in connection with the use of this template including, without limitation, loss of life, loss of data, loss of income or profit, losses sustained as a result of any injury to any person, or loss or damage to property or claims of third parties.

© 2008 National Comprehensive Cancer Network

National Comprehensive Cancer Network®

Chemotherapy Order Template™
Breast Cancer
Dose-Dense AC (DOXOrubicin/Cyclophosphamide) → Dose-Dense PACLItaxel

Dose-Dense PACLItaxel Course

INDICATION:
Adjuvant

REFERENCES:
1. NCCN Clinical Practice Guidelines in Oncology™ Breast Cancer. V.1.2009.
2. Citron ML, et al. *J Clin Oncol.* 2003; 21(8):1431-9.[a]

NCCN SUPPORTIVE CARE:
1. *Emetic Risk:* Day 1 Low
2. *Fever Neutropenia Risk:* High

CHEMOTHERAPY REGIMEN
14-day cycle for 4 cycles

- **PACLItaxel** 175 mg/m^2 IV over 3 hours on Day 1

This course is 4 cycles of dose-dense PACLItaxel.
This course is initiated following completion of the dose-dense AC (DOXOrubicin/cyclophosphamide) course.
Please see Order Template BRS13a for the dose-dense AC (DOXOrubicin/ cyclophosphamide) course.

SUPPORTIVE CARE

Premedications

PACLItaxel requires premedication for hypersensitivity:

- **H_2 antagonist:**
  Famotidine 20 mg IV/PO 30 – 60 minutes pre-PACLItaxel
  OR
  Ranitidine 50 mg IV or 150 mg PO 30 – 60 minutes pre-PACLItaxel
  OR
  Cimetidine 300 mg IV/PO 30 – 60 minutes pre-PACLItaxel
  AND
- **H_1 antagonist**:
  Diphenhydramine 12.5 – 50 mg IV/PO 30 – 60 minutes pre-PACLItaxel
  AND
- **Dexamethasone:**
  Dexamethasone 20 mg PO approximately 12 and 6 hours pre-PACLItaxel
  OR
  Dexamethasone 20 mg IV 30 minutes pre-PACLItaxel

Template continued on page 2

This template is a peer-reviewed statement of the consensus of its authors derived from the NCCN Clinical Practice Guidelines in Oncology™ regarding their views of currently accepted approaches to treatment. This template does not constitute an order. Any clinician seeking to treat a patient using this template is expected to use independent medical judgment in the context of individual clinical circumstances of a specific patient's care or treatment. NCCN disclaims all warranties, express or implied including, without limitation, the implied warranties of merchantability and fitness for a particular purpose. NCCN does not warrant the accuracy, currency, or completeness of the template or make any representation regarding the use or the results of the use of the template in treatment. In no event shall NCCN or its members be liable for any damages including, without limitation, incidental, indirect, special, punitive, or consequential damages arising out of or in connection with the use of this template including, without limitation, loss of life, loss of data, loss of income or profit, losses sustained as a result of any injury to any person, or loss or damage to property or claims of third parties.

07/05/2009

© 2009 National Comprehensive Cancer Network

NCCN National Comprehensive Cancer Network®

Chemotherapy Order Template™
Breast Cancer
Dose-Dense AC (DOXOrubicin/Cyclophosphamide) → Dose-Dense PACLItaxel

Dose-Dense PACLItaxel Course

Antiemetic therapy (See www.nccn.org/professionals/physician_gls/PDF/antiemesis.pdf)

Day 1
No additional dexamethasone needed on Day 1 if dexamethasone already given for hypersensitivity.

- Dexamethasone 12 mg PO/IV Day 1
  OR
- Prochlorperazine 10 mg PO/IV every 4 or every 6 hours Day 1
  OR
- Metoclopramide 10 – 40 mg PO/IV every 4 or every 6 hours Day 1
  AND
- ± Lorazepam 0.5 – 2 mg PO/IV every 4 or every 6 hours as needed Day 1
  AND
- ± H_2 blocker or proton pump inhibitor

PRN for breakthrough: Patients should be given at least one medication in a different category than that given above to have as needed for breakthrough. Please consult the NCCN Clinical Practice Guidelines in Oncology™ Antiemesis for appropriate antiemetic therapy.

Myeloid growth factor therapy (See www.nccn.org/professionals/physician_gls/PDF/myeloid_growth.pdf)

- **Filgrastim (Category 1*)**
  5 mcg/kg/day subcutaneously daily recommended to start 24 – 72 hours after completion of chemotherapy and to continue until post-nadir ANC recovery to normal or near-normal levels by laboratory standards. Dose is rounded to the nearest vial size by institution-defined weight limits. Same-day administration is not recommended.
  OR
- **Pegfilgrastim (Category 1*)**
  6 mg subcutaneously recommended to be given 24 – 72 hours after completion of chemotherapy for one dose only. There are insufficient data to support dose and schedule of weekly regimens or schedules less than 2 weeks and these cannot be recommended. Same-day administration is not recommended.
  OR
- **Sargramostim (Category 2B*)**
  250 mcg/m^2/day subcutaneously daily recommended to start 24 – 72 hours after completion of chemotherapy and to continue until post-nadir ANC recovery to normal or near-normal levels by laboratory standards. Dose is rounded to the nearest vial size by institution-defined weight limits. Same-day administration is not recommended.

MONITORING AND HOLD PARAMETERS

- CBC with differential should be assessed routinely for potential dose evaluation.
- For PACLItaxel:
  - Liver function should be assessed routinely for potential dose evaluation.
  - Hypersensitivity reaction may occur with infusion. Monitor for and treat hypersensitivity reactions per institutional standard.
  - Signs and symptoms of neurotoxicity should be assessed prior to each dose. Modifications of chemotherapy may be warranted.

Template continued on page 3

This template is a peer-reviewed statement of the consensus of its authors derived from the NCCN Clinical Practice Guidelines in Oncology™ regarding their views of currently accepted approaches to treatment. This template does not constitute an order. Any clinician seeking to treat a patient using this template is expected to use independent medical judgment in the context of individual clinical circumstances of a specific patient's care or treatment. NCCN disclaims all warranties, express or implied including, without limitation, the implied warranties of merchantability and fitness for a particular purpose. NCCN does not warrant the accuracy, currency, or completeness of the template or make any representation regarding the use or the results of the use of the template in treatment. In no event shall NCCN or its members be liable for any damages including, without limitation, incidental, indirect, special, punitive, or consequential damages arising out of or in connection with the use of this template including, without limitation, loss of life, loss of data, loss of income or profit, losses sustained as a result of any injury to any person, or loss or damage to property or claims of third parties.

07/05/2009

© 2009 National Comprehensive Cancer Network

National Comprehensive Cancer Network®

NCCN

Chemotherapy Order Template™

Breast Cancer

Dose-Dense AC (DOXOrubicin/Cyclophosphamide) → Dose-Dense PACLItaxel

Dose-Dense PACLItaxel Course

SAFETY PARAMETERS AND SPECIAL INSTRUCTIONS

- For PACLItaxel:
  - PACLItaxel is an irritant.
  - PACLItaxel should be prepared either in glass or non-PVC containers and administered through non-PVC tubing and an in-line filter of not greater than 0.22 microns.

*The NCCN Guidelines Steering Committee has devised a set of Categories of Consensus. These annotations contain two dimensions: the strength of the evidence behind the recommendation and the degree of consensus about its inclusion.

- **Category 1:** There is uniform NCCN consensus, based on high-level evidence, that the recommendation is appropriate.
- **Category 2A:** There is uniform NCCN consensus, based on lower-level evidence including clinical experience, that the recommendation is appropriate.
- **Category 2B**: There is nonuniform NCCN consensus (but no major disagreement), based on lower-level evidence including clinical experience, that the recommendation is appropriate.
- **Category 3:** There is major NCCN disagreement, regardless of the level of evidence, that the recommendation is appropriate.

This template is a peer-reviewed statement of the consensus of its authors derived from the NCCN Clinical Practice Guidelines in Oncology™ regarding their views of currently accepted approaches to treatment. This template does not constitute an order. Any clinician seeking to treat a patient using this template is expected to use independent medical judgment in the context of individual clinical circumstances of a specific patient's care or treatment. NCCN disclaims all warranties, express or implied including, without limitation, the implied warranties of merchantability and fitness for a particular purpose. NCCN does not warrant the accuracy, currency, or completeness of the template or make any representation regarding the use or the results of the use of the template in treatment. In no event shall NCCN or its members be liable for any damages including, without limitation, incidental, indirect, special, punitive, or consequential damages arising out of or in connection with the use of this template including, without limitation, loss of life, loss of data, loss of income or profit, losses sustained as a result of any injury to any person, or loss or damage to property or claims of third parties.

© 2009 National Comprehensive Cancer Network 07/05/2009

NCCN National Comprehensive Cancer Network®

BRS16b

Chemotherapy Order Template™

Breast Cancer

AC (DOXOrubicin/Cyclophosphamide) Every 21 Days → T (PACLItaxel) Every 21 Days + Trastuzumab

T (PACLItaxel) Every 21 Days + Trastuzumab Course

INDICATION:	REFERENCES:	NCCN SUPPORTIVE CARE:
Adjuvant	1. NCCN Clinical Practice Guidelines in Oncology™ Breast Cancer. V.1.2009.<br>2. Romond EH, et al. *N Engl J Med.* 2005;353(16):1673-84.[a]	1. *Emetic Risk:* Day 1 Low; Trastuzumab Minimal<br>2. *Fever Neutropenia Risk:* Refer to NCCN Clinical Practice Guidelines in Oncology™ Myeloid Growth Factors. V.1.2009.

CHEMOTHERAPY REGIMEN

21-day cycle for 4 cycles

- **PACLItaxel** 175 mg/m^2 IV over 3 hours on Day 1

Weekly to complete 52 weeks total of trastuzumab

- **Trastuzumab**
  - 4 mg/kg IV over 90 minutes on Day 1 of Week 1 followed by
  - 2 mg/kg IV over 30 minutes weekly beginning with Week 2

OR

Weekly to complete 12 weeks total of trastuzumab

- **Trastuzumab**
  - 4 mg/kg IV over 90 minutes on Day 1 of Week 1 followed by
  - 2 mg/kg IV over 30 minutes weekly beginning with Week 2

Followed by

21-day cycle to complete 52 weeks total of trastuzumab

- **Trastuzumab**
  - 6 mg/kg IV over 30 – 90 minutes every 21 days beginning Week 13

This course is 4 cycles of T (PACLItaxel) Every 21 Days and 52 weeks of trastuzumab.
This course is initiated following completion of the AC (DOXOrubicin/cyclophosphamide) Every 21 Days course.
Please see Order Template BRS16a for the AC (DOXOrubicin/cyclophosphamide) Every 21 Days course.

Template continued on page 2

This template is a peer-reviewed statement of the consensus of its authors derived from the NCCN Clinical Practice Guidelines in Oncology™ regarding their views of currently accepted approaches to treatment. This template does not constitute an order. Any clinician seeking to treat a patient using this template is expected to use independent medical judgment in the context of individual clinical circumstances of a specific patient's care or treatment. NCCN disclaims all warranties, express or implied including, without limitation, the implied warranties of merchantability and fitness for a particular purpose. NCCN does not warrant the accuracy, currency, or completeness of the template or make any representation regarding the use or the results of the use of the template in treatment. In no event shall NCCN or its members be liable for any damages including, without limitation, incidental, indirect, special, punitive, or consequential damages arising out of or in connection with the use of this template including, without limitation, loss of life, loss of data, loss of income or profit, losses sustained as a result of any injury to any person, or loss or damage to property or claims of third parties.

© 2009 National Comprehensive Cancer Network 07/02/2009

Chemotherapy Order Template™

Breast Cancer

AC (DOXOrubicin/Cyclophosphamide) Every 21 Days → T (PACLItaxel) Every 21 Days + Trastuzumab

T (PACLItaxel) Every 21 Days + Trastuzumab Course

SUPPORTIVE CARE

Premedications

PACLItaxel requires premedication for hypersensitivity:

- **H_2 antagonist**:
  Famotidine 20 mg IV/PO 30 – 60 minutes pre-PACLItaxel
  OR
  Ranitidine 50 mg IV or 150 mg PO 30 – 60 minutes pre-PACLItaxel
  OR
  Cimetidine 300 mg IV/PO 30 – 60 minutes pre-PACLItaxel
  AND
- **H_1 antagonist**:
  Diphenhydramine 12.5 – 50 mg IV/PO 30 – 60 minutes pre- PACLItaxel
  AND
- **Dexamethasone**:
  Dexamethasone 20 mg PO approximately 12 and 6 hours pre-PACLItaxel
  OR
  Dexamethasone 20 mg IV 30 minutes pre-PACLItaxel

Antiemetic therapy (See www.nccn.org/professionals/physician_gls/PDF/antiemesis.pdf)df

Day 1
No additional dexamethasone needed on Day 1 if dexamethasone already given for hypersensitivity.

- Dexamethasone 12 mg PO/IV Day 1
  OR
- Prochlorperazine 10 mg PO/IV every 4 or every 6 hours Day 1
  OR
- Metoclopramide 10 – 40 mg PO/IV every 4 or every 6 hours Day 1
  AND
- ± Lorazepam 0.5 – 2 mg PO/IV every 4 or every 6 hours as needed Day 1
  AND
- ± H_2 blocker or proton pump inhibitor

PRN for breakthrough: Patients should be given at least one medication in a different category than what given above to have as needed for breakthrough. Please consult the NCCN Clinical Practice Guidelines in Oncology™ Antiemesis for appropriate antiemetic therapy.

Days of trastuzumab:

PRN for breakthrough: Although this is a minimally emetic chemotherapy regimen, all patients should be provided with antiemetic therapy for breakthrough emesis. Please consult the NCCN Clinical Practice Guidelines in Oncology™ Antiemesis for appropriate antiemetic therapy.

Template continued on page 3

This template is a peer-reviewed statement of the consensus of its authors derived from the NCCN Clinical Practice Guidelines in Oncology™ regarding their views of currently accepted approaches to treatment. This template does not constitute an order. Any clinician seeking to treat a patient using this template is expected to use independent medical judgment in the context of individual clinical circumstances of a specific patient's care or treatment. NCCN disclaims all warranties, express or implied including, without limitation, the implied warranties of merchantability and fitness for a particular purpose. NCCN does not warrant the accuracy, currency, or completeness of the template or make any representation regarding the use or the results of the use of the template in treatment. In no event shall NCCN or its members be liable for any damages including, without limitation, incidental, indirect, special, punitive, or consequential damages arising out of or in connection with the use of this template including, without limitation, loss of life, loss of data, loss of income or profit, losses sustained as a result of any injury to any person, or loss or damage to property or claims of third parties.

© 2009 National Comprehensive Cancer Network 07/02/2009

National Comprehensive Cancer Network®

NCCN

BRS16b

Chemotherapy Order Template™

Breast Cancer

AC (DOXOrubicin/Cyclophosphamide) Every 21 Days → T (PACLItaxel) Every 21 Days + Trastuzumab

T (PACLItaxel) Every 21 Days + Trastuzumab Course

MONITORING AND HOLD PARAMETERS

- CBC with differential should be assessed routinely for potential dose evaluation.
- For PACLItaxel:
  - o Liver function should be assessed prior to each cycle for potential dose evaluation.
  - o Hypersensitivity reaction may occur with infusion. Monitor for and treat hypersensitivity reactions per institutional standard.
  - o Signs and symptoms of neurotoxicity should be assessed prior to each dose. Modifications of chemotherapy may be warranted.
- For trastuzumab:
  - o Hypersensitivity reaction may occur with infusion. Monitor for and treat hypersensitivity reactions per institutional standard.
  - o Ejection fraction should be assessed prior to initiation of treatment and as clinically indicated.

SAFETY PARAMETERS AND SPECIAL INSTRUCTIONS

- For PACLItaxel:
  - o PACLItaxel is an irritant.
  - o PACLItaxel should be prepared either in glass or non-PVC containers and administered through non-PVC tubing and an in-line filter of not greater than 0.22 microns.

This template is a peer-reviewed statement of the consensus of its authors derived from the NCCN Clinical Practice Guidelines in Oncology™ regarding their views of currently accepted approaches to treatment. This template does not constitute an order. Any clinician seeking to treat a patient using this template is expected to use independent medical judgment in the context of individual clinical circumstances of a specific patient's care or treatment. NCCN disclaims all warranties, express or implied including, without limitation, the implied warranties of merchantability and fitness for a particular purpose. NCCN does not warrant the accuracy, currency, or completeness of the template or make any representation regarding the use or the results of the use of the template in treatment. In no event shall NCCN or its members be liable for any damages including, without limitation, incidental, indirect, special, punitive, or consequential damages arising out of or in connection with the use of this template including, without limitation, loss of life, loss of data, loss of income or profit, losses sustained as a result of any injury to any person, or loss or damage to property or claims of third parties.

© 2009 National Comprehensive Cancer Network 07/02/2009

Appendix C
Overview of Investigational Agents and Biomarkers

1. Overview of Investigational Agents Study Status

Table 1.1 Investigational Agents Approved, Pending Activation for Randomization

There are no Investigational Agents approved, pending activation for randomization at this time.

Table 1.2 Investigational Agents Approved, Activated for Randomization

Agent	Target	HER2+ / HR+	HER2+ / HR–	HER2– / HR+	HER2– / HR–
**T-DM1 plus pertuzumab**	**ErbB2/EGFR /ErbB3 dimerization inhibitor**	**Yes*** **	**Yes*** **	**No**	**No**

****T-DM1 plus pertuzumab is delivered in place of paclitaxel and trastuzumab in HER2+ participants*

Table 1.3 Investigational Agents Graduated or Dropped, No Longer Activated for Randomization

2. Summary of All Additional Eligibility Criteria Required for All Investigational Agents

Table 2 Additional Investigational Agents Eligibility Criteria

Investigational Agents Inclusion Criteria

Inclusion criteria:

- Creatinine clearance >**40** mL/min per 24-hour urine collection or calculated according to the Cockcroft-Gault formula (either formula can be used dependent on the reported units):

**CrCl (mL/min) =**	$\frac{(140\text{–age}) \times \text{actual body weight (kg)}}{72 \times \text{serum creatinine (mg/dL)}}$	(× 0.85 for females)
	$\frac{(140\text{–age}) \times \text{actual body weight (kg)}}{0.8136 \times \text{serum creatinine } (\mu\text{mol/L})}$	(× 0.85 for females)

- Urinary protein quantitative value of ≤30 mg/dL in urinalysis or ≤1+ on dipstick. (If criteria cannot be met, 24-hour urine collection can be done to calculate total protein excretion. If a 24 hour total urinary protein excretion is <1000 mg, the participant may be included.)
- Diastolic blood pressure (DBP) <100 mm Hg and systolic blood pressure (SBP) <160 mm Hg on at least one of three sequential blood pressure determinations performed during their clinic visit, or within the week during the screening process. (At the discretion of the investigator, a participant with DBP ≥90 mm Hg but <100 mm Hg or with SBP ≥140 but <160 mm Hg may be excluded based on prior history of poorly controlled hypertension or lack of compliance with management.)
- PTT or APTT ≤1.5 × ULN per institutional laboratory range and INR ≤1.5.
- HgbA1C ≤8%. Participants with diabetes who meet the HbgA1C criteria are eligible. Participants currently on metformin OR another oral hypoglycemic agent as their medication to manage their insulin resistance are eligible and should stay on their current treatment. Insulin-dependent diabetics are eligible for study participation.
- QTcF ≤470 msec on EKG (test results can be used if done within 30 days of entry to screening phase)

CONFIDENTIAL

- No evidence of clinically significant bradycardia (HR <50 bpm), or history of clinically significant bradyarrhythmias such as sick sinus syndrome, 2nd degree AV block (Mobitz Type 2), or participants taking digoxin
- No history of venous (DVT), arterial, thromboembolism or pulmonary thromboembolism within 12 months prior to screening.
- No history of stroke (cerebrovascular accident) or TIA within 12 months prior to screening
- No history of hypertensive crisis or hypertensive encephalopathy within six months prior to screening.
- No history of clinically significant bleeding within six months prior to screening
- No treatment with immune modulators such as systemic cyclosporine or tacrolimus within 30 days prior to treatment.
- No major surgery within **28 days** prior to enrollment in the screening phase or with a persistent open wound.
- No minor surgical procedures, within **three days** prior to randomization. (Placement of tunneled central venous access device acceptable).
- No serious non-healing wound, ulcer (including gastrointestinal) or bone fracture.
- No therapeutic anti-coagulation (*i.e*., warfarin or LMWH).
- No history of abdominal fistula, GI perforation, or intra-abdominal abscess within 6 months prior to screening.
- No active chronic gastrointestinal disorder with diarrhea as a major symptom in the past two years (*e.g*., Crohn's disease, malabsorption, or grade >2 diarrhea of any etiology at baseline).
- No history of uncontrolled seizures
- Serum potassium, magnesium, and calcium levels within the laboratory's reference range
- No ventricular tachycardia or a supraventricular tachycardia that requires treatment with a Class Ia antiarrhythmic drug (eg, quinidine, procainamide, disopyramide) or Class III antiarrhythmic drug (eg, sotalol, amiodarone, dofetilide). Use of other antiarrhythmic drugs is permitted.
- No use of medications that have been linked to the occurrence of torsades de pointes (see Table 8 for the list of such medications at the end of the appendix M)
- No second- or third-degree atrioventricular (AV) block unless treated with a permanent pacemaker
- No complete left bundle branch block (LBBB)
- No history of long QT Syndrome or a family member with this condition

NOTE: In addition to the eligibility criteria in section 4.1.2 of the main protocol, participants must also meet all additional eligibility criteria described above (Table 2) in order to be eligible for the treatment phase of I-SPY 2. Each investigational agent-specific eligibility criteria can also be found in §2.2 of each investigational agent-specific appendix.

3. Dose and Administration Schedules for Investigational Agents

3.1 Dose and Administration Schedules for Investigational Agents Approved, Activated for Randomization

Table 3.1.3 Paclitaxel (q1w × 12 weeks), and Pertuzumab (q3w × 12 weeks) with Trastuzumab (q1w × 12 weeks); Followed by AC (q2w or q3w)

Agent	Dose	Route	Cycle[b]
Paclitaxel	80 mg/m^2	iv	1–12
**Pertuzumab[a]**	**840 mg (loading dose)**<br>**420 mg (thereafter)**	iv	**1,**<br>**4, 7, 10**

CONFIDENTIAL

Agent	Dose	Route	Cycle[b]
**Trastuzumab**	**4 mg/kg (loading dose)**<br>**2 mg/kg (thereafter)**	iv	**1**<br><br>**2–12**
Doxorubicin	60 mg/m^2	iv	13–16
Cyclophosphamide	600 mg/m^2	iv	13–16

[a] Pertuzumab is administered 1 hour before the delivery of weekly paclitaxel. On days that all three drugs are given, trastuzumab infusion is given first followed by pertuzumab followed by paclitaxel infusion
[b]Note that each cycle for paclitaxel combinations = one week, each cycle for AC = two or three weeks.

Table 3.1.4 T-DM1 (q3w × 12 weeks), and Pertuzumab (q3w × 12 weeks); Followed by AC (q2w or q3w)

Agent	Dose	Route	Cycle[b]
**T-DM1[a]**	**3.6 mg/kg**	**IV**	**1–4**<br>(weeks 1,4,7,10)
**Pertuzumab [a]**	**840 mg (loading dose)**<br>**420 mg (thereafter)**	**IV**	1<br>2–4<br>**(weeks 4, 7, 10)**
Doxorubicin	60 mg/m^2	IV	5–8
Cyclophosphamide	600 mg/m^2	IV	5–8

[a] T-DM1 is administered one hour after completion of pertuzumab
[b]Note that each cycle for T-DM1 and pertuzumab combinations = three weeks, each cycle for AC = two or three weeks.

3.2 Dose and Administration Schedules for Approved Investigational Agents Pending Activation for Randomization

There are no Investigational Agents approved, pending activation for randomization at this time.

3.3 Dose and Administration Schedules for Investigational Agents Graduated or Dropped, No Longer Active for Randomization

4. Qualifying and Exploratory Biomarkers

Table 4 I-SPY 2 List of Biomarkers

Specimen	Aliquot	Assay	Where done	Proposed by
Core, Frozen 1	Five 8 µm sections	RPMA	CAPMM at GMU, Wulfkuhle	Investigator–Chip Petricoin
	One 8 am section	H&E	UCSF I-SPY Lab	I-SPY 2 Protocol
	Half of a core, sectioned	RNA–Agilent 44K Microarray	Agendia	I-SPY 2 Protocol
	400 ng DNA	DNA- SNP Array	Giacomini Lab at UCSF	Investigators–Kathy Giacomini, Matt Goetz, Liewei Wang

CONFIDENTIAL

Specimen	Aliquot	Assay	Where done	Proposed by
	TBD	DNA–Methylation	TBD	General Investigator Discussion
	TBD	DNA–p53 Implicit	TBD	Roche Diagnostics
	TBD	DNA–PIK3CA mut by TaqMan	TBD	Roche Diagnostics
Core, Frozen 2	1 µg isolated tumor RNA	RNA- Affymetrix U133A Microarray and TFAC/RCB predictor	MDACC	Investigator–Fraser Symmans
	TBD	PIK3CA mut, PIK3CA amp, PTEN loss	TBD	Pfizer–Pan ErbB Agent
	TBD	Expression of ErbB members (EGFR, HER2, HER3, HER4)	TBD	Pfizer–Pan ErbB Agent
Core, Frozen 3	FFPE, 2 sections	mRNA array assay to predict drug sensitivity	UCSF- I-SPY Lab	Investigator–Joe Gray
	FFPE, 1 section	IHC: CHFR	Kaufmann lab at Mayo	Investigator–Scott Kaufmann & Matt Goetz
	FFPE, sections	IHC: AKT-S308, PTEN, Stathmin	TBD	Pfizer–Pan ErbB Agent
Core, Frozen 4				
Serum	100 µl	IGF pathway related proteins (IGF-1R, IGF-2)	Hixon Lab at Brown Univ.	Pfizer– IGFR Agent
	TBD	Her2	TBD	Pfizer–Pan ErbB Agent
Plasma				
Buffy Coat	400 ng DNA	GWAS	Giacomini Lab at UCSF	Investigator–Kathy Giacomini, Matt Goetz, Liewei Wang
Bone Marrow and/or Blood	TBD	CTC	TBD	Investigator–John Park (companion study)

CONFIDENTIAL

Appendix K
Pertuzumab with Trastuzumab

I-SPY 2 Investigational Agent Information

INVESTIGATIONAL AGENT INFORMATION SUMMARY

Agent Class:	ErbB2/EGFR/ErbB3 dimerization inhibitor
Structural Class:	Fully human monoclonal antibody (IgG_1)
Manufacturer:	Genentech/Roche
Drug Chaperones:	Dr. Stephen Chia, University of British Columbia

Pharmaceutical Information:

Dosage Form:	Intravenous infusion
Physical Description:	Sterile, preservative-free liquid concentrate supplied at 30 mg/ml pertuzumab in 20 mM L histidine acetate (pH 6.0), 120 mM sucrose and 0.02% polysorbate 20
Strength:	Each 20-mL drug product vial contains 420 mg of pertuzumab (14.0 mL/vial)
Storage Conditions:	Single use vials are to be refrigerated at 2°C–8°C (36°F–46°F).
Special Storage Instructions:	Vial contents should be protected from light, and should not be frozen.

Administration Information:

Route:	Intravenous
Standard Regimen:	840 mg loading dose, then 420 mg q3wk
Agent Preparation:	The solution of pertuzumab for infusion, diluted in polyvinylchloride (PVC) or non-PVC polyolefin bags containing 0.9% Sodium Chloride Injection, USP, may be stored for up to 24 hours prior to use. The diluted solution should be stored refrigerated (2°C–8°C). Because the formulation does not contain a preservative, the vial seal may only be punctured once. Any remaining solution should be discarded.
Pre-medication:	Specific pre-medication is not required for routine treatment. Participants who experience pertuzumab infusion-associated symptoms may be pre-medicated for subsequent infusions with acetaminophen and anti-histamines.
Administration:	Treatment will be administered on an outpatient basis. Pertuzumab will be administered on Day 1 of the first taxane-containing cycle at the required loading dose of 840 mg as an iv infusion. Three weeks (21 days) after the first dose of pertuzumab and every three weeks thereafter, pertuzumab will be administered at a dose of 420 mg as an iv infusion. The initial dose of pertuzumab will be given after the infusion of trastuzumab (following a 60 minutes observation period) and administered over 60 (± 10) minutes with participants to be observed for a further 60 minutes. The infusion should be slowed or interrupted if the participant experiences infusion-related symptoms. If the infusion is well

CONFIDENTIAL

tolerated, subsequent doses may be administered over 30(±10) minutes and participants will be observed for a further 60 minutes for infusion-related symptoms such as fever, chills.

All infusion-related symptoms must have resolved before any paclitaxel is given or the participant is discharged. Participants who experience infusion-related symptoms may be pre-medicated with acetaminophen and anti-histamines for subsequent infusions.

Dose reduction for toxicity is not permitted. Missed doses of pertuzumab can be made up in combination with missed doses of paclitaxel and trastuzumab unless otherwise specified in §2.6 Table 3.

Concomitant Medications: There are no known agents known to interact adversely with concomitantly administered pertuzumab.

Other:

Refer to §2.6 for side effect management and dose reduction plans.

The above is intended as a summary only; please see the complete appendix for additional investigational agent information.

CONFIDENTIAL

1. RATIONALE FOR TESTING

Pertuzumab (rhuMAb 2C4), is a fully humanized monoclonal antibody, that acts by blocking the association of HER2 with other HER family members, including EGFR, HER3, and HER4, to form HER2 heterodimers [1]. Dimerization is crucial for activation of EGFR/HER receptors that contribute to tumor growth and progression. Although both pertuzumab and trastuzumab bind to the extracellular domain of HER2, they bind to distinct epitopes, and consequently, ligand-activated downstream signaling is blocked by pertuzumab but not by trastuzumab. This results in intracellular inhibition of two major signaling pathways, MAPK and PI3K. Inhibition of these signaling pathways can result in cell growth arrest and apoptosis, respectively [2].

Like trastuzumab, pertuzumab also stimulates antibody-dependent, cell-mediated cytotoxicity [3] but due to their complementary modes of action, the combination of pertuzumab with trastuzumab exerts greater antitumor activity than with either agent alone in HER2 overexpressing disease. In phase 2 studies the pertuzumab-trastuzumab regimen has shown activity in participants with HER-2+ metastatic and early breast cancer (EBC) [4, 5].

Furthermore, pertuzumab may not require HER2 overexpression to exert activity as an antitumor agent. For example, EGFR is frequently overexpressed or activated in a number of human tumors [6]. In contrast to transformation of normal cells by HER2, transformation by EGFR requires not only EGFR overexpression but also EGFR ligand expression. Because sustained intensive EGFR signaling frequently involves coordinate activation of HER2 by heterodimerization, inhibition of the HER2 component of this complex may arrest the growth of tumors driven by ligand-activated EGFR.

The role of HER3 in breast cancer is becoming increasingly recognized [7]. Studies in cell culture systems have shown that heregulin (HRG)-activated HER3–HER2 heterodimers elicit the strongest proliferative and transformation responses of any possible receptor combination [8]. Trastuzumab is thought to be effective in disrupting ligand independent HER2-HER3-PI3K complexes, while pertuzumab prevents ligand-induced HER2-HER3 dimerization [9]. In *in vivo* breast cancer xenograft models with nude mice, HER2/HER3 heterodimers were detected in 100% of xenografts inhibited by pertuzumab, where as pertuzumab-resistant lines showed a much lower level of heterodimerization [3].

Inhibition of these critical cell pathways by pertuzumab is expected to synergize with chemotherapy (and trastuzumab for HER2+ breast cancer) and this has been borne out in both preclinical experiments and in clinical studies. Augmentation by pertuzumab of the antitumor effects of the paclitaxel, irinotecan, cisplatin, gemcitabine and capecitabine, has been shown in various human lung, breast [3], gastric and ovarian tumor xenograft models [10]. In the clinic, pertuzumab combination therapy with taxanes (docetaxel or paclitaxel) has been examined in five separate completed or ongoing studies [5, 10, 11]. Most recently the results of the phase 3 CLEOPATRA study were published [12] showing the combination of pertuzumab, trastuzumab (Herceptin), and docetaxel compared to placebo + HT in first-line treatment of metastatic breast cancer (MBC) and showed significantly improved response rates and prolonged progression-free survival with no significant additional toxicity.

Overall, pertuzumab has been generally well tolerated when administered as monotherapy as well as in combination with other anticancer therapies [10]. Gastrointestinal toxicities (diarrhea, nausea, vomiting, decreased appetite) and fatigue are the most frequently reported AEs with single-agent therapy. The majority of these AEs were grade 1 or 2 in severity. Diarrhea, and rash are common events increased with pertuzumab in combination with chemotherapy compared with chemotherapy alone. The most common AEs during dual therapy with pertuzumab and trastuzumab in metastatic breast cancer were diarrhea, fatigue, nausea, and rash. The most frequently occurring AEs during neoadjuvant treatment of participants with EBC with pertuzumab with/without trastuzumab in combination with docetaxel were alopecia,

CONFIDENTIAL

neutropenia, diarrhea, nausea, fatigue, rash, and mucosal inflammation. Infusion related/hypersensitivity/anaphylactic reactions have been rarely identified in participants (<1%) receiving pertuzumab. A low level of cardiac toxicities, predominantly asymptomatic declines in left ventricular ejection fraction (LVEF), have been reported.

1.1 Biological Actions

1.1.1 *In Vitro* and Mechanistic Studies

In vitro studies show that pertuzumab blocks ligand-activated HER2 signaling whereas trastuzumab does not. In the breast carcinoma cell line MCF-7, pertuzumab, but not trastuzumab, blocked HRG-induced activation of the PI3K cell survival pathway—as indicated by a lack of phosphorylation of a key enzyme (Akt) in this pathway [2]. Furthermore, pertuzumab blocks HRG-dependent *in vitro* growth of a number of breast cancer cell lines as well as cell lines derived from other solid tumors [10]. For example, in the HRG-secreting MDA-MB-175VII breast carcinoma cell line, which expresses low/moderate levels of HER2 protein (1+ by immunohistochemistry), cell proliferation was inhibited in a dose-dependent fashion by both pertuzumab and trastuzumab but the magnitude of the inhibition was far greater with pertuzumab [10]. The combination of trastuzumab and pertuzumab was shown to have a synergistic growth inhibiting effect on BT474 breast tumors, which express high levels of HER2, underscoring the complementary mechanism of action of the two drugs [13].

The calculated pertuzumab concentration at which half-maximal growth inhibition (IC_{50}) occurred was 120 ng/mL or 0.8 nM, and is consistent with biochemical measurements of pertuzumab inhibition of HRG binding or receptor activation [14].

1.1.2 Animal Studies

Pertuzumab alone or in combination with paclitaxel, irinotecan, cisplatin, gemcitabine capecitabine, and erlotinib exhibited antitumor activity in various human tumor xenograft models.

Pertuzumab showed single agent activity in xenograft models of lung adenocarcinoma, breast carcinomas, and non-small cell lung cancer tumors [10]. For example, growth inhibition ranging from 50%–70% compared with control was observed with pertuzumab in five out of 18 NSCLC xenografts [14]. Several of the pertuzumab growth-inhibited NSCLC lines displayed strong HER2 activation. Similar studies using six different human mammary tumor explants revealed one clear responder with more than 90% growth inhibition to single agent pertuzumab [14], while one out of four ovarian tumor explants was inhibited by more than 70% [14].

The combination of pertuzumab and trastuzumab synergistically inhibited the growth of xenografts derived from HER2-overexpressing KPL-4 breast cancer cells [3] The synergistic action of pertuzumab and trastuzumab may be explained by their complementary modes of action: while pertuzumab prevents the ligand-activated formation of HER2 heterodimers, trastuzumab can block the shedding of HER2 extracellular domain that would result in constitutively activated truncated receptors.

1.1.3 Human Studies

Twenty-seven phase 1, 2 and 3 clinical trials are completed or ongoing to evaluate the PK, pharmacodynamics, safety, and efficacy of pertuzumab in participants with advanced solid tumors, including breast cancer, NSCLC, prostate, and ovarian cancer. As of the most current IB (with a safety data cut-off date of December 7, 2014), 6886 patients have received at least one dose of pertuzumab as

CONFIDENTIAL

the primary investigational medicinal product in company-sponsored (i.e., Roche or Chugai-sponsored) completed and ongoing clinical studies. An additional 1549 patients have received pertuzumab in combination with investigational compounds in company-sponsored trials.

A phase 1 monotherapy dose-escalation trial administered doses between 0.5 and 15 mg/kg pertuzumab iv every three weeks to 21 participants with incurable, locally advanced solid tumors (three with breast cancer) to investigate safety and PKs [2]. Pertuzumab was generally well tolerated and the maximum tolerated dose was not reached. Nineteen of 21 participants completed at least two cycles of pertuzumab therapy. Two participants, one with ovarian cancer and one with pancreatic islet cancer, had PRs of 10 and 11 months respectively, and six achieved stable disease for more than 2.5 months [2].

In single-agent pertuzumab studies, partial responses or stable disease lasting ≥six months has been observed in 15% of participants with ovarian cancer (study TOC2689g) and in 8% of participants with HER2 low-expressing breast cancer (BO16934). No responses were observed in clinical studies of single agent pertuzumab in participants with hormone-refractory prostate cancer (BO17004, TOC2682g) or NSCLC (TOC2752g) [10].

The phase 2 BO16934 study evaluated the efficacy of pertuzumab when administered in either 420 mg or 1050 mg doses to participants with previously treated, MBC with low HER2 expression [15]. During the study, participants received a median of two cycles of treatment (range 1–24). Two participants in the low dose group had a partial response, with a duration of 18 weeks in one participant and 31 weeks in the other. No participants in the higher dose group had an objective response. In total, 32 participants (41.0%) had stable disease as best response, of which two participants in the low-dose arm and two in the high-dose arm had stable disease for ≥six months, to yield an overall clinical benefit response rate of 7.7%.

The recent publication of a multicenter, open-label, single-arm, two-stage study (BO17929) reported on the response of participants with advanced HER2-positive breast cancer. The disease progression in these participants had occurred during prior trastuzumab-based therapy who received trastuzumab weekly (4 mg/kg loading dose, then 2 mg/kg every week) or every three weeks (8 mg/kg loading dose, then 6 mg/kg every three weeks) and pertuzumab every three weeks (840 mg loading dose, then 420 mg every three weeks). Treatment continued until disease progression or excessive toxicity [4]. All 66 participants were assessable for efficacy and safety. The objective response rate was 24.2%, and the clinical benefit rate was 50%. Five participants (7.6%) experienced a CR, 11 participants (16.7%) experienced a PR, and 17 participants (25.8%) experienced SD of ≥ six months. Median PFS was 5.5 months. In light of the efficacy of dual-agent pertuzumab and trastuzumab, the protocol was amended to include a third cohort of participants (n=29) who received pertuzumab monotherapy. If the participant’s disease failed to respond to pertuzumab monotherapy, or responded and then relapsed, participants could, at the investigators’ discretion, continue pertuzumab and have trastuzumab re-introduced. The primary analysis of the third cohort of Study BO17929 adds support to the view that the two antibodies, pertuzumab and trastuzumab, are more active than either antibody alone. Currently, four patients are in survival follow up, and one patient remains on treatment, remaining on treatment for over 7.5 years and has received 130 cycles of pertuzumab and trastuzumab (Cycle 85 was omitted due to pneumococcal pneumonia).

In a phase 1b combination trial, 19 participants with advanced solid tumors (one with breast cancer) were administered iv doses of 1050 mg pertuzumab combined with 60 or 75 mg/m^2 docetaxel or 75 or 100 mg/m^2 docetaxel following 420 mg pertuzumab with a loading dose of 840 mg [11]. Both drugs were administered iv every three weeks. SD was observed in more than half of treated participants; >50% decline in prostate-specific antigen was reported for one participant whose hormone-resistant prostate cancer was treated with 75 mg/m^{-2} docetaxel and 420 mg pertuzumab with a loading dose of 840 mg; however, there was no suggestion of a response in the breast cancer participant. Doses of 75 mg/m^{-}

CONFIDENTIAL

[2]docetaxel and 420 mg pertuzumab with a loading dose of 840 mg were recommended for a phase 2 trial. There were no drug-drug interactions [11].

In the phase 3 pivotal study WO20698/TOC4129g (CLEOPATRA) of 808 participants with previously untreated HER2+ MBC, a statistically significant and clinically meaningful improvement in PFS was observed in participants treated with pertuzumab, trastuzumab and docetaxel (n=402) compared to those receiving placebo, trastuzumab and docetaxel (n=406). PFS was prolonged at the median by 6.1 months and the risk of disease progression or death was reduced by 38% (hazard ratio = 0.62; 95% CI = 0.51, 0.75; p<0.0001) with an improvement in median PFS from 12.4 months to 18.5 months [12]. The second interim analysis for OS performed on May 14, 2012, crossed the predefined stopping boundary for statistical significance (p ≤ 0.0138), demonstrating that treatment with pertuzumab, trastuzumab and docetaxel significantly improved OS compared with placebo, trastuzumab and docetaxel (HR = 0.66; 95% CI: 0.52, 0.84; p = 0.0008. Following the statistically significant improvement in OS observed at the second interim analysis, patients still receiving study treatment in the placebo arm and whose disease had not progressed were offered crossover from placebo to pertuzumab. Forty eight patients crossed over to receive pertuzumab. The final analysis of OS took place after 389 deaths had occurred, with a median follow-up of 50 months. Despite crossover, the statistically significant OS benefit in favor of the pertuzumab-treated group was maintained (HR = 0.68; 95% CI: 0.56, 0.84; p=0.0002). The median OS was longer by 15.7 months in the pertuzumab, trastuzumab and docetaxel compared to the placebo, trastuzumab and docetaxel (median 56.5 months and 40.7 months, respectively). The median PFS for pertuzumab, trastuzumab and docetaxel and placebo, trastuzumab and docetaxel were 18.7 months and 12.4 months, respectively (HR=0.68; 95% CI, 0.58, 0.80) [10].

In the phase 2 study WO20697 (NEOSPHERE), 417 participants with HER2-positive EBC receiving combination neoadjuvant therapy with pertuzumab, trastuzumab and docetaxel (n=107) had a pCR rate of 46%, compared with 29% in participants receiving trastuzumab plus docetaxel (n=107) (p=0.0141, 95% CI 21–39) [5].

In the phase 2 study BO22280 (TRYPHAENA) 223 participants with HER2+ EBC received neoadjuvant pertuzumab and trastuzumab a) concomitantly with anthracycline-based treatment (n=72); b) following anthracycline-based treatment (n=75) or c) concomitantly with a carboplatin-based regimen (N=76). All three treatment regimens were efficacious, with 57%–66% of participants achieving a pCR [16].

A phase 2 study BP27836 (JOSHUA) evaluated the responses of two different doses of pertuzumab in combination with trastuzumab, capecitabine, and cisplatin in 30 patients with HER2-positive locally advanced or metastatic GEJ or GC. Arm A received a loading dose of 840 mg followed by 420 mg of pertuzumab once every three weeks, while Arm B received 840 mg every three weeks. Both combinations appear to be active in HER2-positive GEJ or GC with a clinical response rate at an explorartory endpoint of 86% in Arm A and 55% in Arm B. However, given the small sample, 15 patients in each arm, no conclusions regarding the relationship between the dose and response can be made [10]

In other combination treatment studies with pertuzumab in tumors not selected for HER-2 positivity, participants with platinum-resistant ovarian cancer (TOC3258g) treated with pertuzumab and gemcitabine (n=65) exhibited prolonged PFS over gemcitabine alone (n=65) (hazard ratio = 0.66; 95% CI: 0.43, 1.03). However, in platinum-sensitive ovarian cancer participants (BO17931; n=149), addition of pertuzumab to a carboplatin-based doublet of chemotherapy did not improve PFS [10].

Partial responses were observed in three participants (20%) who received pertuzumab in combination with erlotinib (n=15) in a phase 1 study in NSCLC (WO20024). In addition, in study TOC4603g (PENGUIN) five of 41 with NSCLC who received pertuzumab with erlotinib showed investigator assessed CT response (one CR and four PRs) at day 56 [10].

CONFIDENTIAL

1.2 Ongoing/Planned NCI or Industry Testing

The effects of pertuzumab in participants with solid tumors are being evaluated in 27 ongoing or completed studies, summarized in Table 1 below. The table reflects information provided in the most recent IB, dated February 2015, with a safety data cut-off date of December 7, 2014.

Table 1. Pertuzumab Ongoing and Completed Clinical Trials

Phase*	Indications	Study Size	Doses	Study ID
1a, Completed	Advanced solid Tumors	21	0.5, 2.0, 5.0, 10.0, and 15 mg/kg	TOC2297g
1, Completed	Advanced solid Tumors	18	5, 10, 15 and 25 mg/kg	JO17076[c]
2, Completed	Advanced ovarian Cancer	61<br>62	Cohort 1: 420 mg<br>Cohort 2: 1050 mg	TOC2689g[a]
2, Completed	MBC with low expression of HER2	41<br>37	Arm A: 420 mg<br>Arm B: 1050 mg	BO16934
2, Completed	Chemotherapy naïve, hormone refractory prostate cancer (HRPC)	35<br>33	Cohort 1: 420 mg<br>Cohort 2: 1050 mg	BO17004
2, Completed	Castration resistant PC pretreated with docetaxel	41	420 mg	TOC2682g[a]
2, Completed	Advanced, recurrent non-small cell lung cancer (NSCLC)	43	420 mg	TOC2572g[a]
2, Completed	Prescribed therapy in TOC2689g, TOC2572g or TOC2682g	3	420 mg	TOC2664g
1b, Completed	Advanced solid tumors	18	Pertuzumab 1050 mg q3w<br>Capecitabine (825, 1000, 1250 mg/m2)	BO17003
1b, Completed	Advanced solid tumors	19	Pertuzumab 1050 mg q3w<br>Docetaxel (60, 75 mg/m^2)<br>Pertuzumab 420 mg q3w<br>Docetaxel (75, 100 mg/m^2)	BO17021
1b, Completed	Advanced NSCLC	15	Pertuzumab: 420 mg q3w<br>Erlotinib (100,150 mg/day)	WO20024
2, Ongoing	HER2 positive MBC	66 in cohort 1& 2; 29 co-hort 3	Pertuzumab 420mg q3w<br>Trastuzumab (either 2mg/kg qw or 6mg/kg q3w)	BO17929
2, Closed to enrollment	NSCLC	41	Pertuzumab 420mg q3w<br>Erlotinib 150 mg/day	TOC4603g (PENGUIN)
2, Completed	Platinum-resistant ovarian, peritoneal or fallopian tube cancer	Gem+ Per: 65<br>Gem: 65	Gemcitabine 800 mg/m^2 ± Pertuzumab/ 420mg q3w	TOC3258g

CONFIDENTIAL

Phase*	Indications	Study Size	Doses	Study ID
2, Completed	Platinum-sensitive ovarian cancer	Per+ chemo: 74<br>chemo: 75	Carboplatin-based chemo[c] ± Pertuzumab 420mg q3w	BO17931
2, Completed	HER2 positive EBC and LABC	A: 107<br>B: 107<br>C: 107<br>D: 96	Arm A. trastuzumab, docetaxel[d]<br>Arm B. trastuzumab, docetaxel, pertuzumab[d]<br>Arm C. trastuzumab, pertuzumab[d]<br>Arm D. pertuzumab, docetaxel[f]<br>pertuzumab: 420 mg q3w, trastuzumab: 6mg/kg, docetaxel: 75mg/m2 to 100mg/m^2	WO20697 (NEOSPHERE)
3, Ongoing	HER2 positive MBC	452	Pertuzumab: 420 mg q3w<br>Trastuzumab 6 mg/kg q3w<br>Capecitabine:<br>Arm A (no Pertuzumab): 1250 mg/m²<br>twice-daily for 14 days<br>followed by 7 days rest q3w.<br>Arm B (with Pertuzumab):<br>1000 mg/m²<br>twice-daily for 14 days<br>followed by 7 days rest q3w	MO22324 (PHEREXA)
2, Ongoing	HER2 positive EBC, LABC<br>Neoadjuvant: All q3w	Arm A: 72<br>Arm B: 75<br>Arm C: 76	Pertuzumab: 420 mg<br>Trastuzumab 6 mg/kg<br>FEC: 5-FU 500 mg/m^2, epirubicin 100 mg/m^2 and cyclophosphamide<br>600 mg/m^2)<br>Carboplatin AUC 6<br>Docetaxel: 75 mg/m^2 initially, 100 mg/m2 if no DLT (NB participants on carboplatin and docetaxel with trastuzumab and pertuzumab remain on 75 mg/m^2)	BO22280 (TRYPHENA)
2, Ongoing	HER2 positive locally advanced or MBC	Cohort 1: 106<br>Cohort 2: 107	Pertuzumab: 420 mg q3w<br>Trastuzumab: 6 mg/kg q3w<br>Vinorelbine: 25 mg/m² Day 2 + Day 9 of Cycle 1, followed by 30-35 mg/m² on Day 1 and 8 (or Day 2 and 9) of each subsequent cycle, q3w<br>Cohort 1: Pertuzumab then trastuzumab sequentially in separate infusion bags<br>Cohort 2: Receive trastuzumab + Pertuzumab in one infusion bag	MO27782 (VELVET)

CONFIDENTIAL

Phase*	Indications	Study Size	Doses	Study ID
2, Ongoing	HER2 positive and hormone receptor-positive locally advanced or MBC	211	Pertuzumab: 420 mg q3w<br>Trastuzumab: 6 mg/kg q3w<br>AI: Anastrozole: 1 mg once daily; OR letrozole: 2.5 mg once daily.<br>AI given after optional induction chemotherapy (18 weeks of either docetaxel or paclitaxel)	MO27775 (PERTAIN)
2a, ongoing	Advanced, HER2-positive gastric cancer	Arm A: 15<br><br>Arm B: 15	Trastuzumab, cisplatin + capecitabine<br>Arm A: Pertuzumab: 840 mg (loading for Cycle 1); 420 mg (for Cycles 2-6).<br>Arm B: Pertuzumab 840 mg (all cycles).	BP27836 (JOSHUA)
3, Ongoing	HER2 positive MBC	808	Pertuzumab or placebo + trastuzumab, docetaxel<br>pertuzumab: 420 mg q3w<br>trastuzumab: 6 mg/kg q3w<br>docetaxel: 75 mg/m^2, escalating to100mg/m^2 q3w	WO20698/ TOC4129g (CLEOPATRA)
3, Ongoing	HER2 positive and hormone receptor-positive locally advanced or MBC	1436	Pertuzumab: 420 mg q3w<br>Trastuzumab and taxanes administered in line with approved local Product Information	MO28047 (PERUSE)
3, Ongoing	Platinum-resistant epithelial ovarian cancer	Part 1: 30 patients planned<br>Part 2 154 patients planned	Pertuzumab: 420 mg q3w (840mg/kg loading dose).<br>Topotecan, paclitaxel, and gemcitabine: administered according to the summary of product characteristics, and as per investigator discretion or local practice	MO28113 (PENELOPE)
3, Ongoing	HER2-positive metastatic gastroesophageal junction and gastric cancer	435	Patients in both arms receive:<br>Trastuzumab: 6 mg/kg, q3w<br>Cisplatin: 80 mg/m2 IV, q3w<br>Capecitabine: 1000 mg/m2, PO, b.i.d., 28<br>Doses total, q3w<br>OR<br>5-FU: 800 mg/m2/24 h IV for 120 hours, q3w.<br>Arm A - Pertuzumab: 840 mg IV, q3w<br>Arm B - Placebo: IV, q3w	BO25114 (JACOB)

CONFIDENTIAL

Phase*	Indications	Study Size	Doses	Study ID
2, Ongoing	HER2 positive MBC	69	Paclitxel(80 mg/m2) qw + trastuzumabq3w (8 mg/kg loading dose → 6 mg/kg q3w) + pertuzumab q3w (840 mg as a loading dose → 420 mg q3w)	NCT01276041
3, Ongoing	HER2 positive EBC	4805	Pertuzumab: 420 mg q3w or placebo and<br>trastuzumab: 6 mg/kg q3w to be started with taxane in either an anthracycline or non-anthracycline chemotherapy regimen<br>Anthracycline regimen FEC (or FAC) OR AC (or EC) followed by taxane:<br>docetaxel: 75 mg/m^2 -100mg/m^2 q3w, paclitaxel 80mh/m2 q1w<br>FEC: 5-FU 500 to 600 mg/m^2, epirubicin 90-120 mg/m^2 and cyclophosphamide<br>500 to 600 mg/m^2) q3w<br><br>Non -Anthracycline Regimen<br>Carboplatin AUC 6 (max dose 900mg) q3w<br>Docetaxel: 75 mg/m^2 q3w	BO25126/<br>TOC4939G<br>(APHINITY)

Abbreviations: qod, every other day; qw, once weekly; NA, dose information not available
*Some trials listed may have more recently been completed, closed, or cancelled
[a] "Completed" indicates end of trial (as defined in protocol) has been reached
[b]Japanese studies sponsored by Chugai Pharmaceutical Co Ltd
[c]either paclitaxel (175 mg/m^2 q3w)/carboplatin (AUC 5/q3w) or gemcitabine (1000 mg/m^2 day 1 and day 8 q3w)/carboplatin (AUC 4/q3w);
[d] Arms A–D all receive 5-fluorouracil (600 mg/m^2), epirubicin (90 mg/m^2), cyclophosphamide (600 mg/m^2), and Arm C receives docetaxel 75mg/m^2 to 100mg/m^2 in the adjuvant setting.

1.3 Toxicity and Safety

1.3.1 Animal Studies

In iv toxicity studies in cynomolgus monkeys, pertuzumab treatment at doses up to 150 mg/kg was generally well-tolerated [10]. There was a treatment-related increase in the incidence of diarrhea in all groups of animals administered pertuzumab iv in both seven-and 26-week toxicity studies. In the seven-week study, diarrhea was not severe and did not result in dehydration or adversely affected body weights or clinical pathology parameters. In the 26-week study, diarrhea was more frequent in the treatment groups compared with the control group but did not appear to be dose-dependent. One monkey was euthanized after 18 weeks of treatment, with dehydration related to severe diarrhea, and two monkeys required supportive care for recurrent diarrhea. Diarrhea appeared to be only partially reversible on recovery for one month in the seven-week and on recovery for two months in the 26-week study.

Pertuzumab was administered by iv injection in the repeat-dose toxicity studies in monkeys. No treatment-related clinical observations or histopathologic findings were noted at the injection sites. At

CONFIDENTIAL

concentrations of up to 21.6 mg/ml, pertuzumab did not cause hemolysis of cynomolgus monkey or human erythrocytes [10].

A segment II teratology study was conducted in monkeys to evaluate effects on embryotoxicity and teratogenicity by dosing from gestation day (GD) 19 through 50, the period of organogenesis in this species. Dose levels in the pertuzumab groups consisted of a loading dose on GD 19 (30, 100, or 150 mg/kg in the low-, mid-, or high-dose groups, respectively) followed by a twice a week maintenance dose (10, 33.3, or 100 mg/kg in the low-, mid-, or high-dose groups, respectively). Embryo/fetal losses were noted in all pertuzumab-treated groups. The majority of these losses occurred on approximately GD 30–40 (vehicle control 0/12; low-dose 4/12; mid-dose 6/12; high-dose 10/12) [14]. In addition, low amniotic fluid volume and microscopic evidence of delayed renal development (renal hypoplasia) were observed in all pertuzumab-treated groups [10].

1.3.2 Human Studies

As of the most current IB, with a safety data cut-off of December 7, 2014, an estimated 6886 patients have received pertuzumab as the primary investigational medicinal product and an additional 1549 patients have received pertuzumab in combination with investigational compounds in company-sponsored trials. Overall, pertuzumab has been generally well tolerated when administered as monotherapy and can be given in combination with trastuzumab and a range of other anticancer therapies with manageable additional toxicity. Grade 1 to 2 diarrhea, fatigue, nausea, vomiting, and decreased appetite have been reported as a common AEs in participants receiving single agent pertuzumab (n=386). In combination therapy regimens additional AEs included rash, neutropenia, mucosal inflammation, dry skin, anemia, dyspepsia, and thrombocytopenia depending on the concomitant chemotherapy agent in use (mostly grade 1–2). Details of specific and more uncommon safety findings obtained from ongoing or completed clinical trials with pertuzumab follow [10].

Five dose levels of pertuzumab ranging from 0.5 mg/kg to 25 mg/kg were evaluated in two single agent phase 1 studies (TOC2297g , JO17076), while five phase 2 single agent studies (BO17004, BO16934, TOC2689g, TOC2572g and TOC2682g) treated participants at a 840 mg loading dose followed by 420 mg or 1050 q3wk. Safety data from all of these studies are pooled for this summary. The most commonly reported AEs were fatigue, vomiting, nausea, diarrhea, rash, decrease appetite, and lymphopenia. The majority were CTCAE grade 1 or 2. Forty percent (40%) of participants receiving single-agent pertuzumab experienced higher grade AEs (>grade 3). Diarrhea was the most commonly reported grade 3–4 AE (6.5%) with other higher grade AEs occurring in more than 2% of participants including, vomiting, nausea, small intestine obstruction, dyspnea, pneumonia, pleural effusion, abdominal pain, constipation, and fatigue. A total of 27% of participants receiving single-agent pertuzumab reported at least one SAE. The most common SAE was small intestinal obstruction (3.6%, all reported in Study TOC2689g, in participants with advanced ovarian cancer) followed by pneumonia (2.6%). In the pertuzumab single-agent (fixed, therapeutic dose) studies, 37% of participants died of which the cause in the vast majority (95%) was progressive disease.

There is safety data available (cut-off date February 11, 2014) from the ongoing study of pertuzumab in combination with trastuzumab and docetaxel compared with placebo, trastuzumab and docetaxel in MBC participants (WO20698/TOC4129g (CLEOPATRA)). Overall the safety profile, including the cardiac toxicity of the pertuzumab combination regimen was generally comparable with that of the placebo-controlled arm apart from higher incidences (≥ 5% difference) of grade 1–2 diarrhea, rash, mucosal inflammation, dry skin, headache, upper respiratory tract infection, pruritus, muscle spasms, and grade 3–4 febrile neutropenia. Grade ≥ 3 AEs of neutropenia (46% *vs.* 49%), febrile neutropenia (8% *vs.* 14%) and diarrhea (5% *vs.* 9%) were all more frequent (>2% difference) in participants receiving pertuzumab. SAE incidence was higher in the pertuzumab arm than in the placebo-controlled arm, primarily due to the

CONFIDENTIAL

greater number of reports of febrile neutropenia (11% of participants receiving the pertuzumab combination, compared to 5% of participants in the placebo-controlled arm). At the time of the most recent clinical cut-off, a similar proportion of patients in each of the arms experienced AEs, with varying causalities, that led to discontinuation of all study treatments (8.6% of participants receiving pertuzumab vs. 6.1% of participants who did not). Excluding events that resulted in the discontinuation of docetaxel only, the most common reasons for discontinuation were left ventricular dysfunction, followed by diarrhea. By the clinical cut-off date, 386 deaths had been reported in the study (based on the safety population). There were more deaths in the placebo-controlled arm (217 participants [55%] of which 196 were due to PD) than in the pertuzumab arm (169 participants [41%], of which 150 were due to PD).

An ongoing phase 3b study (MO28047 [PERUSE]) is evaluating pertuzumab in combination with trastuzumab and a taxane in patients with first-line HER2-positive metastatic breast cancer, with an interim analysis performed on a population of 704 patients (cut-off date: September 13, 2013). Median exposure to taxanes, trastuzumab, and pertuzumab was 53 months, with a median number of 6, 8, and 9 cycles, respectively. Initial taxanes selected by the investigator included paclitaxel (47% of patients), docetaxel (45%), and nab-paclitaxel (6%). A majority of patients (97%) experienced at least one adverse event (any grade), with the most frequent including diarrhea, alopecia, nausea, fatigue, asthenia, peripheral neuropathy, mucosal inflammation, rash, epistaxis, vomiting and dysgeusia. Grade ≥3 AEs were reported for 46% of patients; among taxane subgroups, the frequencies of Grade 3 and 4 AEs were 53% (docetaxel), 41% (paclitaxel), and 27% (nab-paclitaxel). The most frequent Grade 3 and 4 AEs included neutropenia, diarrhea, febrile neutropenia, fatigue, asthenia, anemia, mucosal inflammation, dyspnea, and peripheral neuropathy. Five patients died due to AEs: one in the docetaxel subgroup (septicemia) and four in the paclitaxel subgroup (single events of pancreatitis, pneumonitis, sepsis, and an unexplained death).

There is preliminary safety data available (clinical cutoff date: July 10, 2014) from an interim safety analysis of both cohorts in the phase 2 study of pertuzumab in combination with trastuzumab and vinorelbine in patients with HER2-positive advanced (metastatic or locally advanced) breast cancer (MO27782 [VELVET]). Cohort 1 received pertuzumab and trastuzumab sequentially from separate infusion bags, followed by vinorelbine. Cohort 2 received pertuzumab and trastuzumab from a single infusion bag, followed by vinorelbine. At the time of the cutoff, the most common AEs (≥ 20% of patients in Cohort 1) were diarrhea, neutropenia, nausea, asthenia, fatigue, pyrexia, chills, vomiting, constipation, alopecia, anemia, rash, and decreased appetite. The most common AEs (> 20% of patients) were diarrhea, neutropenia, nausea, asthenia, fatigue, pyrexia, vomiting, constipation, hypertension, alopecia, pain in extremity, decreased appetite, muscle spasms, mucosal inflammation, stomatitis. dyspnea, and epistaxis.The most common grade ≥3 AEs reported by three or more patients in Cohort 1 were neutropenia, leucopenia, febrile neutropenia, diarrhea, asthenia, constipation, anemia, bone pain, fatigue, hypersensitivity, vomiting, and stomatitis. The most commonly reported grade ≥3 AEs in Cohort 2 patients included neutropenia, hypertension, leukopenia, febrile neutropenia, diarrhea, asthenia, fatigue, anemia, mucosal inflammation, pneumonia, nausea, hypokalemiea, general deterioration, and gamma-GT increased In Cohort 1, SAEs were experienced by 31 patients (29%), which the most common events (experienced by 2 or more patients) were febrile neutropenia, pyrexia, hypersensitivity, pneumonia, drug hypersensitivity, and abdominal pain. Thirty-nine patients (36%) experienced at least one SAE, with the most common events (occurring in at least two patients) included febrile neutropenia, pyrexia, pneumonia, neutropenia, device-related infection, pulmonary embolism, pleural effusion, and nausea. In Cohort 1, nine patients died due to AEs (7 due to disease progression, 2 due to an AE), whereas 11 patients died in Cohort 2 ((5 due to disease progression, 6 due to an AE). As of the most recent clinical cut-off, an interim efficacy analysis was performed for Cohort 1. In Cohort 1, the primary endpoint ORR was 62.9% (95% CI: 52.0%, 72.9%), and the study secondary endpoint, interim median PFS, was 14.3 months (95% CI: 11.0, 17.3) in the ITT population [10].

CONFIDENTIAL

In the ongoing study of pertuzumab combined with trastuzumab in HER2+ MBC (BO17929) safety data from all three cohorts is available with cut-off dates as of February 2008 for Cohorts 1 and 2, and November 2010 for Cohort 3. Diarrhea, fatigue, nausea, and rash were reported in cohort 1 and 2 with diarrhea being the most frequent event (64%) which was grade 1 in severity in the majority of cases. Ten participants reported 17 grade ≥3 AEs, all of which occurred as single cases, with the exception of diarrhea (in two participants). A total of 13 SAEs (in 10 participants) were reported: these occurred in single participants with the exception of back pain (two participants).Two Grade 3 SAEs (dizziness and paranoia) were also reported. The most frequent AEs in Cohort 3 were diarrhea, nausea, vomiting, fatigue and asthenia. Seven of the 29 participants experienced a total of 13 grade 3 AEs. All grade 3 AEs occurred in single participants with the exception of diarrhea and fatigue (both two participants). One participant experienced two SAEs (bone fracture and osmotic demyelination syndrome). This participant had cerebral and cerebellar metastases and recent radiotherapy to the brain. At the time of the updated analysis (November 2010) 19 participants in Cohorts 1 and 2, and 11 participants in Cohort 3 had died, of which 18 were PD-related (one participant had died as a result of hepatic coma, secondary to progression of hepatic metastases; later determined to be a progressive disease-related death). Another participant died due to a cerebrovascular accident in survival follow up, over 18 months after withdrawal from treatment.

In an additional NCI-sponsored study of pertuzumab and trastuzumab conducted in MBC participants after multiple lines of treatment failure (TOC3487s/06-C-0035), two grade ≥3 events were reported in the first 11 participants (one event of allergic reaction related to the infusion and one of symptomatic CHF and enrollment was terminated per trial protocol. In addition to the CHF, two participants had grade 2 left ventricular systolic dysfunction (LVSD) and three participants had grade 1 LVSD declines to below 50% of baseline. The reasons for the apparent difference in cardiac toxicity between participants on this study and those in a comparable population in Study BO17929 are not entirely clear but may include differences in eligibility criteria with regards to baseline LVEF and LVEF declines during prior trastuzumab therapy.

A completed trial of neoadjuvant treatment in participants with operable, locally advanced, inflammatory, or early HER2+ breast cancer, comparedfour treatment regimens (WO20697 [NEOSPHERE**]).** The most recent clinical cutoff date (July 2013) includes information from the post-treatment follow-up period. The addition of pertuzumab to trastuzumab plus docetaxel (Arm B) did not notably affect the safety profile compared to neoadjuvant treatment with trastuzumab plus docetaxel (Arm A). The combination of pertuzumab plus docetaxel (Arm D) gave a broadly comparable safety profile to trastuzumab and docetaxel (Arm A) and pertuzumab, trastuzumab and docetaxel (Arm B). However, in the overall treatment period, the proportion of patients with AEs reported in SOC “Cardiac Disorders”, was highest in Arm B (20.6%) and lowest in Arm A (7.5%). The most frequently occurring AEs (≥25% of participants in any arm) in the neoadjuvant period in Arms A, B and D (the docetaxel-containing arms of the study) were alopecia, neutropenia, diarrhea, nausea, fatigue, rash and mucosal inflammation. The majority of AEs were grade 1 or 2 in severity. Participants receiving pertuzumab plus trastuzumab without docetaxel (Arm C) in the neoadjuvant period experienced notably fewer AEs across most body systems than participants in the docetaxel-containing arms of the study. In the adjuvant period the most frequest AEs (≥ 25% of participants in any arm) included nausea, neutropenia, vomiting, fatigue, radiation skin injury, diarrhea and alopecia. In general the incidence of AEs was highest in Arm C, most likely due to the administration of docetaxel during the adjuvant period. In the post-treatment follow-up period, of the 378 patients who entered the post-treatment follow-up period, 7 patients (6.5%) in Arm A, 9 patients (8.4%) in Arm B, 7 patients (6.5%) in Arm C, and 7 patients (7.4%) in Arm D experienced AEs. The most common AEs (in 2 or more patients, in any arm) were: left ventricular dysfunction (LVD), back pain, myalgia, pain in extremity, and anxiety. The most frequently reported grade ≥3 AEs in participants in the neoadjuvant and adjuvant period were were neutropenia, leukopenia, febrile neutropenia, and granulocytopenia. In the post-treatment follow-up period, there were three incidents of Grade ≥ 3 AEs: one event in Arm A (abdominal distension), one in Arm C (breast prosthesis removal), both considered

unrelated to study treatment, and an event in Arm D (myeloproliferative disorder), considered possibly related to study treatment, by the Investigator. The incidence of SAEs was broadly comparable in Arms A, B, and D (10 – 17% of patients). The most frequently reported SAEs in Arms A, B, and D were neutropenia and febrile neutropenia. The only incident of SAE post-treatment was the myeloproliferative disorder in Arm D. Twenty-six participants had died at the time of the third clinical cut-off date, with one occurring in the neoadjuvant period and twenty-five deaths (6 in Arm A, 3 in Arm B, 8 in Arm C, and 8 in Arm D) occurred during the post-treatment follow-up period. Nineteen of the 25 deaths were due to disease progression/breast cancer, four had no cause of death reported, and two were due to colon/colorectal cancer (as additional primary cancer).

An ongoing study (BO22280 [TRYPHAENA]) to evaluate pertuzumab plus trastuzumab given either concomitantly (Arm A) or sequentially (Arm B) with standard anthracycline-based neoadjuvant chemotherapy, or concomitantly with a carboplatin-based neoadjuvant chemotherapy regimen (Arm C) has as its primary endpoint cardiac tolerability, as assessed by LVEF declines and symptomatic LVSD. Incidence of cardiac dysfunction was low, and was consistent with experience in other trials. Following the post-treatment follow-up period (up to the third clinical cutoff, July 2013) results showed that there were no clinically relevant, long term toxicities in any of the three treatment arms. The majority of the LVEF declines were asymptomatic (91.4%) and had recovered to ≥ 50%, at the time of the third clinical cutoff. At the third clinical cutoff, two ongoing cases (LVEF < 50%), one in Arm A and B (previously symptomatic) were considered asymptomatic by the investigator. In the neoadjuvant period the incidence of AEs (all grades) was similar across all three treatment arms, with the majority of AEs Grade 1-2, and the most common AE was diarrhea (61%-72% of patients). The majority of AEs were grade 1-2 in the adjuvant period, with an increased incidence (>10% more) in Arm C compared with at least one of the two other arms: diarrhea, anemia, dysgeusia, insomnia and thrombocytopenia. As of the third clinical cutoff date, of the 219 patients in the post-treatment follow-up period, 20 patients (6 patients [8.3%] in Arm A, 9 patients [12.0%] in Arm B and 5 patients [6.6%] in Arm C) experienced AEs, the most common of which was LVD and all other events occurred in single patients. Two SAEs were reported in the post-treatment followup period in Arm B; one Grade 3 symptomatic LVSD, which was considered possibly related to adjuvant trastuzumab and one Grade 4 neutropenic infection considered possibly related to studytreatment by the investigator.

A randomized multicenter, double-blind, placebo-controlled comparison of chemotherapy plus trastuzumab plus placebo versus chemotherapy plus trastuzumab plus pertuzumab as adjuvant therapy in patients with operable HER2-positive primary breast cancer (Study BO25126/TOC4939 [APHINITY]) was reviewed by the iDMC. The iDMC noted a significant number of cases of Grade 3-4 diarrhea, dehydration and hypokalemia. The iDMC recommended that study sites and treating physicians be alerted to these possible toxicities, and encouraged managing diarrhea, dehydration and hypokalemia aggressively. However it was not deemed necessary to update the protocol and the ICF since information regarding risk of diarrhea is already described in the protocol and diarrhea and dehydration are listed as side effects in the ICF and there was no objection to the continuation of the study. As of November 30, 2013, study recruitment (4.805 patients) is complete.

Additional safety data on the results of studies of pertuzumab treatment in combination with other chemotherapy regimens in ovarian cancer (TOC3258g, BO17931, MO28113), advanced solid tumors (BO17003, BO17021), NSCLC (WO20024, TOC4603g), colorectal cancer (TOC4163s), and gastric cancer (BP27836) are available in the pertuzumab Investigators' Brochure (v 14, February 2015) [10].

1.4 Pharmacokinetics/Pharmacodynamics

1.4.1 Animal Studies

CONFIDENTIAL

PK parameters of pertuzumab have been tested in the mice, rats, and monkeys. Pertuzumab showed a biphasic disposition with an initial distribution phase of <1 day, a terminal elimination half-life of ~10 days, and a volume of distribution which approximates serum volume. Pertuzumab disposition in mice and monkeys is similar to that observed with other humanized monoclonal antibodies sharing the same IgG1 framework.

In dose-response studies, a weekly dose (0.4–60 mg/kg) of pertuzumab was administered to nude mice implanted with NSCLC tumors and breast cancer tumors. Greater than 80% suppression of tumor growth was achieved at steady-state trough concentrations of 5-25 µg/mL in these models.

1.4.2 Human Studies

Similar PKs have been observed across all trials with no change in clearance at and above a dose of 2.0 mg/kg resulting in a systemic serum clearance of approximately 0.24 L/day and a terminal half-life of approximately 18 days for a typical participant[17]. Based on these data a dosing interval of three weeks is recommended in clinical studies. In the phase 2 studies, a loading dose of 840 mg (followed by 420 mg q3w), was capable of attaining steady-state trough and peak concentrations by the second cycle (38–136 µg/ml; 137.5–237.6 µg/ml, respectively) [14]. Population PK modeling of data from phase 1a and phase 2 studies using both fixed, body surface area, and weight-based dosing comparisons support the continued use of fixed, non-weight-based dosing in female participants with breast or ovarian cancer. There was no evidence of an impact of pertuzumab on the PK of co-administered gemcitabine, docetaxel, capecitabine, or erlotinib in phase 1b and 2 studies.

2. INVESTIGATIONAL STUDY AGENT ADMINISTRATION IN THE I-SPY 2 TRIAL

Intervention will be administered on an outpatient basis. Reported clinical AEs and potential risks are described in §3.2.

2.1 Dose Regimen and Dose Groups

The dose schedule for pertuzumab is:

Table 2. Paclitaxel (q1w x 12 weeks), and Pertuzumab (q3wk x 12 weeks) with Trastuzumab (q1w x 12 weeks)[a]; Followed by AC (q2w or q3w)

Agent	Dose	Route	Cycle[b]
Paclitaxel	80 mg/m^2	iv	1–12
**Pertuzumab**	**840 mg (loading dose)<br>420 mg (thereafter)**	iv	**1,<br>4, 7, 10**
**Trastuzumab**	**4 mg/kg (loading dose)<br>2 mg/kg (thereafter)**	iv	**1<br>2–12**
Doxorubicin	60 mg/m^2	iv	13–16
Cyclophosphamide	600 mg/m^2	iv	13–16

[a] Pertuzumab is administered 1 hour before the delivery of weekly paclitaxel. On days that all three drugs are given, trastuzumab infusion is given first followed by pertuzumab followed by paclitaxel infusion. Pertuzumab may be administered after a 60 minute observation period following the trastuzumab infusion. If the infusion is well tolerated subsequent infusions may be administered after a 30 minute waiting period.
Paclitaxel can be delivered after a 60 minute observation period following the pertuzumab infusion. If there were no infusion related symptoms from pertuzumab, subsequent infusions may be administered after a 30 minute

CONFIDENTIAL

observation period. If an infusion related symptom occurs it is recommended a 60 minute observation period be undertaken prior to initiating either the pertuzumab and/or the paclitaxel.
On days that only trastuzumab and paclitaxel are administered, trastuzumab is infused first with no observation period required.

[b]Note that each cycle for paclitaxel and trastuzumab combinations = 1 week, each cycle for pertuzumab =3 weeks; each cycle for AC = 2 or 3 weeks.

Treatment with pertuzumab will be an 840 mg (loading dose), followed three weeks later by 420 mg (regardless of body weight) given every three weeks thereafter combined with paclitaxel at 80 mg/m^2 per week (for 12 weeks continuously) and trastuzumab. Trastuzumab will be administered weekly at a 2mg/kg dose after an initial loading dose of 4 mg/kg body weight. This dose of pertuzumab was chosen based on the demonstrated safety of weekly taxanes combined with q3wk pertuzumab as well as q3wk pertuzumab monotherapy and pertuzumab plus trastuzumab combination therapy in the phase 1–3 trials described above.

2.2.1 Additional Eligibility Criteria

There are no additional eligibility requirements for pertuzumab.

NOTE: Participants must meet all other investigational agent-specific criteria as described in Appendix C §2 and the main protocol §4.1.2 in order to be eligible for the treatment phase of I-SPY 2.

2.3 Contraindications

None available at this time.

2.4 Concomitant Medications

There are no agents known to interact adversely with concomitantly administered pertuzumab

2.5 Clinical Evaluation and Procedures

Laboratory evaluations for general safety monitoring during treatment are described in protocol §8.1–8.3.

- Cardiac safety (LVEF) will be monitored by ECHO or MUGA at the following timepoints (the same method should be used at every time point in each participant):
    - After completion of the taxane regimen and before initiating AC (*i.e.*,12 weeks of the paclitaxel and trastuzumab and pertuzumab regimen)
    - After completing the AC regimen and before surgery

If the results are abnormal, ECHO or MUGA will be repeated one month later

2.6 Dose Modification and Management of Toxicity

Pertuzumab will be continued every third week throughout the paclitaxel chemotherapy course for a total of 4 doses, unless specified otherwise in Table 3 below. There will be no dose reductions for pertuzumab. Missed doses of pertuzumab can be made up in combination with missed doses of paclitaxel and trastuzumab unless otherwise specified in Table 3 below. There will be no dose reductions for trastuzumab, but treatment may be delayed. If treatment is held for more than three consecutive weeks the participant must be removed from the study. If removal is due to cardiac toxicity, the participant should be followed with echocardiograms every three–four months for one year after discontinuation of therapy.

Dose adjustments are to be made according to the organ system showing the greatest degree of toxicity. Toxicity will be graded for severity using the NCI CTCAE version 4.0. Initiation of the next cycle of therapy may be delayed no more than two weeks to allow recovery from toxicity. Treatment delay of ≥ three weeks due to toxicity will lead to stopping all protocol therapy. Participant should proceed with additional chemotherapy or surgery at the discretion of the treating physician. Participant will remain on study for outcome assessment.

Table3. Paclitaxel + Pertuzumab+ Trastuzumab Dose Modification

**Event**	**Paclitaxel + Pertuzumab+ Trastuzumab Dose Modification**
**Neutropenia**	
≥1000/mm^3	No change to paclitaxel, trastuzumab and pertuzumab<br>• For ANC ≤ 1500/mm^3, consider the use of prophylactic myeloid growth factors (filgrastim),<br>o Start on day 2 or 3 and use according to participant need, at physician discretion, and to avoid dose reduction.<br>o Growth factor should not be given on the same day as chemotherapy.<br>o Pegfilgrastim may not be used with paclitaxel due to the weekly dosing in this study.
<1000/mm^3	Hold paclitaxel, trastuzumab and pertuzumab, if combined treatment cycle(1,4,7,10) until ANC ≥ 1000/mm^3. Resume treatment based on timing of recovery:<br>• ≤1 week: No change to paclitaxel ,trastuzumab and pertuzumab.<br>• >1 but <3 weeks: Dose-reduce paclitaxel by 25% for all subsequent cycles. No change to pertuzumab and trastuzumab<br>• ≥3 weeks: Stop paclitaxel ,trastuzumab and pertuzumab. Participant should proceed with additional chemotherapy, trastuzumab or surgery at the discretion of the treating physician. Participant remains on study for outcome assessment.<br><br>G-CSF may be used between days 2–6 according to participant need, at physician discretion, and to avoid dose reduction. Pegfilgrastim may not be used with paclitaxel due to the weekly dosing used in this study.
**Neutropenic Fever**	
ANC ≤1000/mm^3, fever ≥38.5°C	Hold paclitaxel, trastuzumab and pertuzumab if combined therapy cycle (1,4,7,10) until resolved (ANC > 1000/mm^3, fever < 38.5°C). Resume treatment according to number of episodes:<br>• First episode: No change to paclitaxel, trastuzumab and pertuzumab.<br>• Second episode: Reduce paclitaxel by 25% for all subsequent doses. No change to trastuzumab and pertuzumab.<br>• Third episode: STOP paclitaxel, trastuzumab and pertuzumab. Participant should proceed with additional chemotherapy or surgery at the discretion of the treating physician. Participant will remain on study for outcome assessment.<br>• If paclitaxel is held for 3 weeks in a row, permanently discontinue paclitaxel and pertuzumab and trastuzumab. Participant should proceed with additional chemotherapy, trastuzumab or surgery at the discretion of the treating physician. Participant remains on study for outcome assessment.

CONFIDENTIAL

Event	Paclitaxel + Pertuzumab+ Trastuzumab Dose Modification
	G-CSF may be used between days 2–6 according to participant need, at physician discretion, and to avoid dose reduction. Pegfilgrastim may not be used with paclitaxel due to the weekly dosing in this study.
**Thrombocytopenia**	
≥100,000/mm^3	No change to paclitaxel, trastuzumab and pertuzumab.
75–99,999/mm^3	Hold paclitaxel, trastuzumab and pertuzumab, if combined treatment cycle(1,4,7,10) until ≥ 100,000/mm3. Resume treatment based on timing of recovery:<br>• ≤1 week: No change to paclitaxel, trastuzumab and pertuzumab.<br>• ≥1 but <3 weeks: Dose-reduce paclitaxel by 25% for all subsequent cycles. No change to pertuzumab and trastuzumab<br>▪ ≥ 3 weeks: Stop paclitaxel, trastuzumab and pertuzumab. Participant should proceed with additional chemotherapy, trastuzumab or surgery at the discretion of the treating physician. Participant will remain on study for outcome assessment.
<75,000/mm^3	Hold paclitaxel, trastuzumab and pertuzumab, if combination therapy week(1,4,7,10) until ≥ 100,000/mm^3. Resume treatment with a dose-reduction of 25% for all subsequent cycles. No change to trastuzumab and pertuzumab.<br><br>≥ 3 weeks: Stop paclitaxel ,trastuzumab and pertuzumab. Participant should proceed with additional chemotherapy, trastuzumab or surgery at the discretion of the treating physician. Participant remains on study for outcome assessment.
**Anemia**	
All grades	No change to paclitaxel, trastuzumab and pertuzumab.<br>For all anemia events related to paclitaxel regardless of grade, iron studies should be checked and iron should be replaced as indicated.<br>• Red blood cell transfusions can be given at the investigators discretion as needed for symptom control.
**Hepatic**	
Grade 1	No change to paclitaxel, trastuzumab and pertuzumab.
Grade 2	Grade 2 bilirubin<br>Hold paclitaxel, trastuzumab and pertuzumab if combined therapy cycle (1,4,7,10) until bilirubin resolves to ≤ grade 1.. Resume treatment based on timing of recovery.<br>• < 2 weeks- No change to paclitaxel, trastuzumab and pertuzumab.<br>• ≥ 2 weeks- Resume paclitaxel with a 25% reduction in dose for all subsequent doses. No change to trastuzumab and pertuzumab.<br>• If treatment is held for 3 weeks in a row, stop paclitaxel, trastuzumab and pertuzumab. Participant should proceed with additional chemotherapy, trastuzumab or surgery at the discretion of the treating physician. Participant remains on study for outcome assessment.<br><br>A rise in indirect bilirubin with a normal direct bilirubin believed to be attributable to Gilbert's disease does not require change in dose or a drug hold. A note to file should be created.<br><br>Grade 2 AST or ALT:

CONFIDENTIAL

Event	Paclitaxel + Pertuzumab+ Trastuzumab Dose Modification
	Hold paclitaxel, trastuzumab and pertuzumab if combined therapy cycle(1,4,7,10) until AST/ALT resolve to ≤ grade 1. Resume treatment based on timing of recovery:<br>• < 3 weeks- resume paclitaxel at previous dose(s). No change to trastuzumab and pertuzumab.<br>• If paclitaxel is held for 3 weeks in a row, stop paclitaxel, trastuzumab and pertuzumab. Participant should proceed with additional chemotherapy, trastuzumab or surgery at the discretion of the treating physician. Participant remains on study for outcome assessment.
Grade 3	Grade ≥ 3 bilirubin (not due to Gilbert's disease):<br>Stop paclitaxel, trastuzumab and pertuzumab..Participant should proceed with additional chemotherapy or surgery at the discretion of the treating physician. Participant remains on study for outcome assessment.<br><br>Grade 3 AST or ALT:<br>Hold paclitaxel, trastuzumab and pertuzumab if combined treatment cycle (1,4,7,10) until AST/ALT resolve to ≤ grade 1.<br>Resume treatment based on timing to recovery:<br>• < 2 weeks- No change to paclitaxel, trastuzumab and pertuzumab.<br>• ≥ 2 weeks- Resume paclitaxel with a 25% reduction in dose for all subsequent doses. No change to trastuzumab and pertuzumab.<br>• If paclitaxel is held for 3 weeks in a row, stop trastuzumab and pertuzumab. Participant should proceed with additional chemotherapy, trastuzumab or surgery at the discretion of the treating physician. Participant remains on study for outcome assessment.
Grade 4	Grade 4 AST or ALT:<br>Stop paclitaxel, trastuzumab and pertuzumab. Participant should proceed with additional chemotherapy, trastuzumab or surgery at the discretion of the treating physician. Participant remains on study for outcome assessment.
**Hyperglycemia (random glucose)**	
≤Grade 2	No change to paclitaxel, trastuzumab and pertuzumab.
≥ Grade 3	Hold paclitaxel, trastuzumab and pertuzumab if combined treatment cycle (1,4,7,10) until hyperglycemia resolve to ≤ grade 2. Resume treatment based on timing to recovery:<br>• < 2 weeks- No change to paclitaxel, trastuzumab and pertuzumab.<br>• ≥ 2 weeks-Stop paclitaxel, trastuzumab and pertuzumab..<br><br>If ≥ Grade 3 hyperglycemia recurs:<br>Stop paclitaxel trastuzumab and pertuzumab. Participant should proceed with additional chemotherapy, trastuzumab or surgery at the discretion of the treating physician. Participant remains on study for outcome assessment.
**Nausea/Vomiting/Anorexia**	
Grade 0–2	No change to paclitaxel, trastuzumab and pertuzumab.<br>• Nausea and/or vomiting should be controlled with adequate anti-emetic therapy. Prophylactic anti-emetic therapy (*e.g.,* aprepitant, ondansetron, palonosetron, dexamethasone) should be administered to all

CONFIDENTIAL

Event	Paclitaxel + Pertuzumab+ Trastuzumab Dose Modification
	participants; specific agents are at the discretion of the treating physician.<br>• Participants are encouraged to take plenty of oral fluids.
≥ Grade 3	Hold paclitaxel, trastuzumab and pertuzumab if combined treatment cycle(1,4,7,10) until resolved to ≤ grade 1. Resume treatment based on number of episodes:<br>• First episode: No change to paclitaxel, trastuzumab and pertuzumab. Consider modification of premedications.<br>• Second episode despite maximal supportive care: Resume paclitaxel with a 25% dose reduction for all subsequent doses. No change to trastuzumab and pertuzumab<br>• Third episode: Stop paclitaxel, trastuzumab and pertuzumab. Participant should proceed with additional chemotherapy, trastuzumab or surgery at the discretion of the treating physician. Participant remains on study for outcome assessment.
**Diarrhea**	
≥Grade 3	Hold paclitaxel, trastuzumab and pertuzumab until diarrhea resolved to ≤ grade 1. Resume treatment based on time to recovery:<br>• ≤ 1 week—no change to paclitaxel, trastuzumab and pertuzumab.<br>• > 1 week and < 3 weeks—,at physician discretion:<br>o Dose reduce paclitaxel by 25% for all subsequent doses. No change to trastuzumab and pertuzumab<br>• ≥ 3 weeks—stop paclitaxel and PERTUZUMAB and Trastuzumab. Participant should proceed with additional chemotherapy, trastuzumab or surgery at the discretion of the treating physician. Participant remains on study for outcome assessment.
**Mucositis/Stomatitis**	
Grade 0–2	No change to paclitaxel, trastuzumab and pertuzumab.
≥Grade 3	Hold paclitaxel, trastuzumab and pertuzumab until symptoms have resolved to ≤ grade 1. Resume treatment based on number of episodes:,.<br>• First episode: No change to paclitaxel, trastuzumab and pertuzumab. Consider modifications to premedications and the addition of G-CSF..<br><br>For persistent toxicity despite maximal supportive care:<br>• Dose reduce paclitaxel by 25% for all subsequent doses. No change to trastuzumab and pertuzumab.
**Cardiac**	
Asymptomatic decline in LVEF that warrants intervention<br><br>LVEF <lower limit of normal AND absolute decrease in LVEF of ≥10% compared with pretreatment LVEF<br><br>OR	Stop trastuzumab and pertuzumab for 4 weeks but continue paclitaxel Reassess LVEF at 4 weeks:<br>o If LVEF has recovered to baseline or the absolute decrease from baseline is < 15 percentage points, resume trastuzumab and pertuzumab at previous dose.<br>If LVEF has not either recovered to baseline or the absolute decrease from baseline is ≥15 percentage points, stop trastuzumab plus paclitaxel and pertuzumab 4therapy. Participant should proceed with additional chemotherapy or surgery at the discretion of the treating physician. Participant remains on study for outcome assessment of the treating physician. Participant remains on study for outcome assessment.

CONFIDENTIAL

Event	Paclitaxel + Pertuzumab+ Trastuzumab Dose Modification
LVEF had an absolute decrease of ≥15% compared with pretreatment LVEF.	
Symptomatic cardiac heart failure	Stop paclitaxel and trastuzumab and pertuzumab. Refer for cardiologic evaluation and appropriate management for heart failure. Participant should proceed with additional chemotherapy or surgery at the discretion of the treating physician. Participant remains on study for outcome assessment.
**Neurotoxicity**	
Grade 0–2	No change to paclitaxel, trastuzumab and pertuzumab.
Grade 3	Hold paclitaxel, trastuzumab and pertuzumab until neuropathy improves to ≤ grade 2.<br>• Resume paclitaxel with a 25% dose reduction for all subsequent cycles. No change to trastuzumab and pertuzumab.<br>• If paclitaxel is held for 3 weeks in a row for neuropathy, stop paclitaxel, trastuzumab and pertuzumab.. Participant should proceed with additional chemotherapy, trastuzumab or surgery at the discretion of the treating physician. Participant remains on study for outcome assessment.
Grade 4	Stop paclitaxel, trastuzumab and pertuzumab. Participant should proceed with additional chemotherapy, trastuzumab or surgery at the discretion of the treating physician. Participant remains on study for outcome assessment
**Anaphylaxis/Hypersensitivity**	
Mild (*e.g.*, mild flushing, rash, pruritis)	Mild symptoms (grade 1: *e.g.*, transient flushing, rash or fever):<br>• Complete infusion (paclitaxel, trastuzumab and/or pertuzumab).<br>• No treatment required, but observe participant at least until symptoms have resolved.
Moderate (*e.g.*, moderate flushing, rash, mild dyspnea, chest discomfort)	Moderate symptoms (grade 2: *e.g.*, rash, flushing, urticaria, dyspnea, chest discomfort):<br>For paclitaxel:<br>• Hold paclitaxel infusion. No change to pertuzumab and trastuzumab.<br>• Give intravenous diphenhydramine 20–25 mg and intravenous dexamethasone 10 mg.<br>• Resume paclitaxel infusion after recovery of symptoms at half the previous rate for 15 minutes. If no recurrence of symptoms, the planned rate may be resumed.<br>For trastuzumab:<br>• Stop trastuzumab infusion. No change to pertuzumab and paclitaxel<br>• Treat symptomatically according to institutional guidelines.<br>• Resume trastuzumab infusion at a slower rate according to institutional guidelines after reaction has resolved. If no recurrence of symptoms, the planned rate may be resumed.<br><br>For pertuzumab:<br>• Stop pertuzumab infusion. No change to paclitaxel and trastuzumab<br>• Treat symptomatically according to institutional guidelines.<br>• Resume pertuzumab infusion at a slower rate according to institutional guidelines after reaction has resolved. If no recurrence of symptoms, the planned rate may be resumed.<br><br>If symptoms recur after paclitaxel or trastuzumab or pertuzumab re-challenge:

CONFIDENTIAL

Event	Paclitaxel + Pertuzumab+ Trastuzumab Dose Modification
	• Stop paclitaxel or trastuzumab or pertuzumab infusion and stop all subsequent paclitaxel and trastuzumab and pertuzumab treatment. Participant should proceed with additional chemotherapy or surgery at the discretion of the treating physician. Participant remains on study for outcome assessment.<br><br>Any moderate hypersensitivity reaction should be discussed with the protocol chair if drug is to be continued. Contact the DCC for further instructions (1-855-889-5170).
Severe (*e.g*., hypotension requiring pressers, angioedema, respiratory distress requiring bronchodilators)	Severe or life-threatening symptoms (grade 3 or 4: *e.g.,* hypotension, angioedema, respiratory distress or anaphylaxis):<br>• Stop paclitaxel or trastuzumab or pertuzumab infusion.<br>• Administer diphenhydramine 25 mg and dexamethasone 10 mg iv. Add epinephrine or bronchodilators as needed per institutional guidelines.<br>• Stop all subsequent paclitaxel and trastuzumab and pertuzumab. Participant should proceed with additional chemotherapy or surgery at the discretion of the treating physician. Participant remains on study for outcome assessment.
**Other Clinically Significant Toxicity Excluding Fatigue, Alopecia, and Leukopenia at Physician Discretion**	
Grade 0 or 1	No change to any agent.
Grade 2	Hold paclitaxel, trastuzumab and pertuzumab if combined treatment cycle(1,4,7,10), until resolved to ≤ grade 1.<br>Resume paclitaxel, trastuzumab and pertuzumab at previous dose.<br>• Increase supportive care measures if possible
≥ Grade 3	Hold paclitaxel, trastuzumab and pertuzumab. Contact the DCC for further instruction (1-855-889-5170).

Abbreviations: ANC: absolute neutrophil count; DCC: Data Coordinating Center; iv: intravenous injection
Grades refer to CTCAE version 4
*All dose reductions will be based on blood counts obtained on a planned day of chemotherapy. Nadir counts will not be measured routinely.

The following dose levels will be utilized for the purpose of dose modifications for toxicity:

Table 5. Dose Adjustments for Paclitaxel

Dose Adjustment	Paclitaxel Dose, mg/m^2
Standard dose	80
25% reduction	60

[a]Dose to be given only if a dose reduction is required.

There will be no dose reductions for trastuzumab OR pertuzumab used to treat participants with HER2+ disease.

3. INVESTIGATIONAL AGENT PHARMACEUTICAL INFORMATION

3.1 Investigational Study Agent (IND # 105,139, IND Sponsor: QLHC)

CONFIDENTIAL

Confidential pharmaceutical information for investigational study agents supplied by the pharmaceutical partner is available through an FDA IND cross-reference letter.

3.2 Reported Clinical Adverse Events and Potential Risks

As of the most current IB (clinical cut-off date: December 7, 2014), 6886 participants have been exposed to pertuzumab as monotherapy and 1549 patients have received pertuzumab in combination with various chemotherapeutic drugs. Safety data is available from 27 separate studies, representing the majority of these participants completed or proposed for enrollment at this time. The most common and frequent AEs (> 20% of patients) reported for participants receiving pertuzumab monotherapy are diarrhea, fatigue, nausea, vomiting, and decreased appetite, with the majority being grade 1 or 2. Pertuzumab was well tolerated when given in combination with trastuzumab with an increase in the incidence but not the severity of the AEs seen with pertuzumab alone (notably diarrhea, rash and fatigue). When compared to the AE profile seen with the combination of trastuzumab and docetaxel, the addition of pertuzumab added little additional toxicity (predominantly diarrhea, mucosal inflammation, rash, and febrile neutropenia) when all three drugs were used concurrently.

The most frequently occurring AEs during neoadjuvant treatment with pertuzumab, trastuzumab and docetaxel were alopecia, neutropenia, diarrhea, nausea, fatigue, rash, and mucosal inflammation, The tolerability of this regimen was broadly comparable to pertuzumab plus docetaxel alone. Where pertuzumab and trastuzumab were given in addition to commonly used anthracycline-based and carboplatin-based neoadjuvant regimens, the most common AEs were diarrhea, alopecia, nausea, neutropenia, vomiting, fatigue, anemia, dyspepsia, and thrombocytopenia.

Serious or severe infusion-related symptoms have rarely been observed in participants receiving pertuzumab. A low level of cardiac toxicities, predominantly associated with asymptomatic declines in LVEF, have been reported. Importantly, despite targeting the same HER2 pathway, pertuzumab adds no significant cardiac toxicity when given with trastuzumab (with or without chemotherapy).

CONFIDENTIAL

Infusion-related Symptoms

In participants receiving single-agent pertuzumab, 2% of participants experienced AEs during pertuzumab infusions including symptoms such as fever, chills, diarrhea, fatigue, hypotension, shortness of breath, skin rash, headache, nausea, vomiting, and/or hypersensitivity reactions, including anaphylaxis. If a significant infusion-associated reaction occurs, the infusion should be slowed down or interrupted and appropriate medical therapies should be administered.

Left Ventricular Dysfunction

In completed studies, the incidence of left ventricular dysfunction associated with pertuzumab does not appear to be greater than that associated with trastuzumab. In participants selected for having good cardiac function at baseline and with minimal cardiac risks, the combination of trastuzumab and pertuzumab is well tolerated, even when chemotherapy is also administered. Overall, 16 symptomatic cardiac failure events (as reported in individual studies as symptomatic CHF or LVSD) have been reported in 1326 participants with advanced malignant disease or early stage breast cancer treated with pertuzumab (data from completed primary analyses). Six of these events were reported in participants with MBC, two in participants with ovarian cancer, and five in participants with EBC.

Five of the participants (all with MBC) had received prior anthracyclines. Of the participants with breast cancer, all but one received pertuzumab in combination with trastuzumab. In the pivotal phase 3 study WO20698/TOC4129g, a total of 24 participants (6.1%) receiving pertuzumab plus trastuzumab and docetaxel experienced a decline in LVEF, compared to 28 participants (7.4%) in the placebo-controlled arm. Participants with significant cardiac disease or a baseline LVEF below the institution's lower limit of normal should not commence treatment with pertuzumab. Monitoring of LVEF is advised while participants are receiving pertuzumab.

Diarrhea and Rash

Diarrhea was observed in approximately 57% of participants treated with single-agent pertuzumab in phase 2 studies, and between 43%–100% of participants who received pertuzumab in phase 2 and phase 3 combination therapy studies. Diarrhea was Grade 1 or 2 in the vast majority of events.

Rash was observed in approximately 24% of participants receiving single-agent pertuzumab, with the majority (20%) deemed treatment related. Rash was observed in 11%–73% of participants in pertuzumab combination studies. The rash was generally of Grade 1 or 2 severity.

Neutropenia

In the phase 3 study WO20698/TOC4129g incidence of Grade ≥ 3 febrile neutropenia was increased in participants with MBC receiving pertuzumab, trastuzumab and docetaxel compared to those in the placebo-controlled arm (13.0% *vs.* 7.6%, respectively). No febrile neutropenia AEs started after docetaxel was discontinued.

Respiratory Symptoms

There is a potential risk of interstitial lung disease (ILD) because of pertuzumab's role in inhibiting heterodimerization with other members of the HER family, including EGFR. However, few reports of ILD have been received from participants receiving pertuzumab and these indicated alternative causes for the events (*e.g.*, concomitant medication, preceding/concurrent neutropenia with potential infection) or relevant medical history. Three out of 386 participants who received single-agent pertuzumab reported an

CONFIDENTIAL

ILD adverse event, will all requiring treatment. However, only one was reported as Grade ≥3, and one was attributed to the pertuzumab. In the phase 3 study WO20698/TOC4129g, the percentage of participants receiving pertuzumab, trastuzumab and docetaxel compared to those in the placebo-controlled arm and experienced an ILD adverse event were 2.5% and 1.5%, respectively.

Venous Thromboembolic Events

In study WO20698/TOC4129g, 15 participants (3.7%) receiving pertuzumab, trastuzumab, and docetaxel compared to 6 participants (1.5%) experienced venous thromboembolic events. Nine participants receiving pertuzumab, trastuzumab, and docetaxel experienced grade ≥3 venous thromboembolic events, with two considered to be related to study treatment (both were pulmonary embolism events, with one considered by the investigator to be related to docetaxel). In study BO22280, in the neoadjuvant period, 2 patients receiving pertuzumab and trastuzumab concomitantly with FEC and then with docetaxel (Arm A), and 1 patient receiving concomitant carboplatin, pertuzumab, trastuzumab and docetaxel (Arm C) experienced venous thromboembolic events (Arm A: Grade 2 thrombophlebitis and Grade 4 pulmonary embolism; Arm C: Grade 2 thrombophlebitis), which were all considered possibly related to the treatment study. In the adjuvant period, one participant who received sequential FEC then pertuzumab, trastuzumab and docetaxel in the neoadjuvant period (Arm B) experienced a Grade 3 thrombosis, but was considered unrelated to the study treatment.

Immunogenicity

In the phase 1/2 trials, two of the 366 pertuzumab-treated participants (0.5%) who had at least one post-dose sample available for anti-therapeuticantibody analysis, tested positive for antibodies to pertuzumab. Both participants experienced grade 3 hypersensitivity reactions that precluded further administration of pertuzumab. In the phase 3 WO20698/TOC4129g study, antibodies to pertuzumab were detected in 6.7% of participants in the placebo, trastuzumab and docetaxel arm and 3.3% of participants in the pertuzumab, trastuzumab and docetaxel arm. Although exploratory analyses of efficacy based on anti-therapeutic antibody status suggests that efficacy in both treatment arms might have been reduced in patients with positive anti-therapeutic antibodies, the analyses should be viewed in the context of a limited participant sample with positive anti-therapeutic antibodies, None of these participants experienced anaphylactic/hypersensitivity reactions that were clearly related to anti-drug antibody development.

3.3 Investigational Agent Availability

Pertuzumab is manufactured by Genentech. The drug product is provided in a 20-mL vial which contains 420 mg of pertuzumab (14.0 mL/vial). The sterile, preservative-free liquid concentrate is supplied at 30 mg/ml pertuzumab in 20 mM L-histidine acetate (pH 6.0), 120 mM sucrose and 0.02% polysorbate 20. The investigator or designee will record the lot number, expiration date and the amount of study medication dispensed. Pertuzumab is provided under a CTA between Genentech and the QLHC.

3.4 Investigational Agent Distribution

Shipment of investigational agents to a participating site will not be approved until documentation of IRB approval of the sponsor-approved protocol and consent is available, and the collection of all Essential Documents is complete.

Investigational agents may be requested by the investigator (or their authorized designees) at each organization. Investigational agents will be shipped directly to the institution or site where the agent will be prepared and administered. The transfer of agents between institutions in not permitted (unless prior approval from the sponsor is obtained). Agents are requested by completing the Investigational Agent

CONFIDENTIAL

Request Form (to include complete shipping contact information). Please refer to the I-SPY 2 Manual of Operations for completion and submission guidelines.

Once DCC establishes that the requesting site is authorized to receive investigational agents, the order will be forwarded by DCC to the manufacturer, who will ship the investigational agent directly to the study site. Instructions for ordering investigational agents are available in the I-SPY 2 Manual of Operations and Procedures.

.

3.5 Investigational Agent Preparation and Handling

The solution of pertuzumab for infusion, diluted in PVC or non-PVC polyolefin bags containing 0.9% Sodium Chloride Injection, USP, may be stored for up to 24 hours prior to use. The diluted solution should be stored refrigerated (2°C–8°C). Because the formulation does not contain a preservative, the vial seal may only be punctured once. Any remaining solution should be discarded. The shelf-life of the clinical and commercial drug product is 24 months.

Pertuzumab should be prepared using aseptic technique and administered intravenously by infusion. 14 ml pertuzumab liquid concentrate should be withdrawn and diluted into a 250-mL PVC or non-PVC polyolefin 0.9% sodium chloride infusion bag. After dilution, 1 mL of solution should contain approximately 3.36mg of pertuzumab (840mg/250mL) for the initial dose, where two vials are required, and approximately 1.68 mg of pertuzumab (420mg/250mL) for the subsequent dose where one vial is required. The bag should be gently inverted to mix in order to avoid foaming. Parenteral drug products should be inspected for particulates and discolouration prior to administration. Once the infusion is prepared it should be administered immediately

No incompatibilities between pertuzumab and polyvinylchloride, polyethylene or non- PVC polyolefin bags have been observed. D5W solution should not be used to dilute pertuzumab since it is chemically and physically unstable in such solutions.

3.6 Investigational Agent Accountability

The investigator, or a responsible party designated by the investigator, must maintain a careful record of the inventory and disposition of all investigational agents. The investigator is required to maintain adequate records of receipt, dispensing, and final disposition of study agent. Include on the receipt record from whom the agent was received and to whom study agent was shipped, date, quantity and batch or lot number. On the dispensing record, note quantities and dates study agent was dispensed to and returned by each participant.

3.7 Investigational Agent Packaging and Labeling

Pertuzumab is packaged and labeled by Genentech according to their established procedures. Labels are printed and attached to the study drug vial or other packaging container prior to shipping to the site. Each will be labeled with a single panel label that will include, but is not limited to, the following information:

- Blank spaces to write the study number and investigator name
- IND caution statement
- Drug identification
- Lot number
- Storage conditions
- Dosing instructions
- Blank spaces to write the participant's identification number, initials and date dispensed

CONFIDENTIAL

Each label must remain affixed to the bottle or vial.

Samples of labels:

3.8 Investigational Agent Storage

Single use vials are to be refrigerated at 2°C–8°C (36°F–46°F). Vial contents should be protected from light, and should not be frozen.

3.9 Investigational Agent Destruction/Disposal

Once drug accountability is performed, the participating sites should use local/institutional procedures for disposal of returned/unused study drug and bottles/containers. Copies of all certificates of destruction of any unused study drug must be provided to DCC. **Prior to destruction,** the pharmacist should contact the assigned study monitor.

Study sites may also return study drugs to the manufacturer for destruction if they are unable to destroy locally. Please contact DCC for instructions.

CONFIDENTIAL

REFERENCES

1. Franklin MC, Carey KD, Vajdos FF, Leahy DJ, de Vos AM, Sliwkowski MX. Insights into ErbB signaling from the structure of the ErbB2-pertuzumab complex. Cancer Cell 2004;5:317–28.
2. Agus DB, Gordon MS, Taylor C, Natale RB, Karlan B, Mendelson DS, Press MF, Allison DE, Sliwkowski MX, Lieberman G, Kelsey SM, Fyfe G. Phase I clinical study of pertuzumab, a novel HER dimerization inhibitor, in patients with advanced cancer. J Clin Oncol 2005;23:2534–43.
3. Scheuer W, Friess T, Burtscher H, Bossenmaier B, Endl J, Hasmann M. Strongly enhanced antitumor activity of trastuzumab and pertuzumab combination treatment on HER2-positive human xenograft tumor models. Cancer Res 2009;69:9330–36.
4. Baselga J, Gelmon KA, Verma S, Wardley A, Conte P, Miles D, Bianchi G, Cortes J, McNally VA, Ross GA, Fumoleau P, Gianni L. Phase II trial of pertuzumab and trastuzumab in patients with human epidermal growth factor receptor 2-positive metastatic breast cancer that progressed during prior trastuzumab therapy. J Clin Oncol 2010;28:1138–44.
5. Gianni L, Pienkowski T, Im Y-H, Roman L, Tseng L-M, Liu M-C, Lluch A, Staroslawska E, de la Haba-Rodriguez J, Im S-A, Pedrini JL, Poirier B, Morandi P, Semiglazov V, Srimuninnimit V, Bianchi G, Szado T, Ratnayake J. Efficacy and safety of neoadjuvant pertuzumab and trastuzumab in women with locally advanced, inflammatory, or early HER2-positive breast cancer (NeoSphere): A randomised multicentre, open-label, phase 2 trial. Lancet Oncol 2012;13:25–32.
6. Fan Z, Mendelsohn J. Therapeutic application of anti-growth factor receptor antibodies. Curr Opin Oncol 1998;10:67–73.
7. Sithanandam G, Anderson LM. The ERBB3 receptor in cancer and cancer gene therapy. Cancer Gene Ther 2008;15:413–48.
8. Riese DJ n, van Raaij TM, Plowman GD, Andrews GC, Stern DF. The cellular response to neuregulins is governed by complex interactions of the erbB receptor family. Mol Cell Biol 1995;15:5770–76.
9. Junttila TT, Akita RW, Parsons K, Fields C, Lewis Phillips GD, Friedman LS, Sampath D, Sliwkowski MX. Ligand-independent HER2/HER3/PI3K complex is disrupted by trastuzumab and is effectively inhibited by the PI3K inhibitor GDC-0941. Cancer Cell 2009;15:429–40.
10. F. Hoffmann-La Roche Ltd. Investigator's brochure. RO4368451. Pertuzumab (rhuMAb 2C4). (IND 9900). Fourteenth, February 2015.
11. Attard G, Kitzen J, Blagden SP, Fong PC, Pronk LC, Zhi J, Zugmaier G, Verweij J, de Bono JS, de Jonge M. A phase Ib study of pertuzumab, a recombinant humanised antibody to HER2, and docetaxel in patients with advanced solid tumours. Br J Cancer 2007;97:1338–43.
12. Baselga J, Cortes J, Kim S-B, Im S-A, Hegg R, Im Y-H, Roman L, Pedrini JL, Pienkowski T, Knott A, Clark E, Benyunes MC, Ross G, Swain SM. Pertuzumab plus trastuzumab plus docetaxel for metastatic breast cancer. N Engl J Med 2012;366:109–19.
13. Nahta R, Hung M-C, Esteva FJ. The HER-2-targeting antibodies trastuzumab and pertuzumab synergistically inhibit the survival of breast cancer cells. Cancer Res 2004;64:2343–46.
14. F. Hoffmann-La Roche Ltd. Investigator's brochure. RO4368451. Pertuzumab (rhuMAb 2C4). (IND 9900). Eleventh version, February 2012.
15. Cortes J, Baselga J, Kellokumpu-Lehtinen P, Bianchi G, Cameron D, Miles D, Salvagni S, Wardley A, Goeminne J-C, Gianni L. Open label, randomized, phase II study of pertuzumab (P) in patients (pts) with metastatic breast cancer (MBC) with low expression of HER2. J Clin Oncol 2005;23 (Suppl):abst. No. 3068.
16. Schneeweiss A, Chia S, Hickish T, Harvey V, Eniu A, Hegg R, Tausch C, Seo J-H, Tsai Y-F, Ackrill A, Ross G, Cortés J. Neoadjuvant pertuzumab and trastuzumab concurrent or sequential with an anthracycline-containing or concurrent with an anthracycline-free standard regimen: A randomized phase II study (TRYPHAENA). CTRC-AACR San Antonio Breast Cancer Symposium 2011;34th:abst. No. S5-6.
17. Allison DE, Ng, C., Derynck, M.K., Lutzker, S.G., Jimenez, V. and Kelsey, S.M. Pharmacokinetics (PK) of pertuzumab (rhuMAb 2C4) in phase II studies of ovarian, breast, prostate, and lung cancers. J Clin Oncol 2005;23 (suppl):abst. No. 2532.

CONFIDENTIAL

Appendix L
Pertuzumab + Trastuzumab Emtansine
(T-DM1, Trastuzumab-MCC-DM1)

I-SPY 2 Investigational Agent Information

INVESTIGATIONAL AGENT INFORMATION SUMMARY

General Information:

Pertuzumab:

Agent Class:	ErbB2/EGFR/ErbB3 dimerization inhibitor
Structural Class:	Fully human monoclonal antibody (IgG_1)
Manufacturer:	Genentech/Roche

T-DM1:

Agent Class:	Anti-HER2 antibody, Antimitotic, Antimicrotubule
Structural Class:	Antibody-Drug Conjugate (MW 148.5 kDa)
Manufacturer:	Genentech/Roche
Drug Chaperones:	Dr. Angela DeMichele, University of Pennsylvania<br>Dr. Stacy Moulder, MD Anderson

Pharmaceutical Information:

Pertuzumab:

Dosage Form:	Intravenous infusion
Physical Description:	Sterile, preservative-free liquid concentrate supplied at 30 mg/ml pertuzumab in 20 mM L histidine acetate (pH 6.0), 120 mM sucrose and 0.02% polysorbate 20
Strength:	Each 20-mL drug product vial contains 420 mg of pertuzumab (14.0 mL/vial)
Storage Conditions:	Single use vials are to be refrigerated at 2°C–8°C (36°F–46°F).
Special Storage Instructions:	Vial contents should be protected from light, and should not be frozen.

T-DM1:

Dosage Form:	Supplied as a lyophilized product for reconstitution with sterile water for injection (SWFI)
Strength:	Trastuzumab emtansine (T-DM1) is available for clinical trials in 100 mg and 160 mg vials.
Storage Conditions:	Store intact bottle at temperature 2–8ºC; protect from light.
Packaging Unit:	15 mL single-use vial containing 100 mg T-DM1 *or* 20 mL vial containing 160 mg T-DM1
Special Storage Instructions:	Protect from light. Do not freeze.

Administration Information:

Pertuzumab:

Route:	Intravenous
Standard Regimen:	840 mg loading dose, then 420 mg q3wk

CONFIDENTIAL

Agent Preparation: The solution of pertuzumab for infusion, diluted in PVC or non-PVC polyolefin bags containing 0.9% sodium chloride injection, USP, may be stored for up to 24 hours prior to use. The diluted solution should be stored refrigerated (2°C–8°C). Because the formulation does not contain a preservative, the vial seal may only be punctured once. Any remaining solution should be discarded.

Pre-medication: Specific pre-medication is not required for routine treatment. Participants who experience pertuzumab infusion-associated symptoms may be pre-medicated for subsequent infusions with acetaminophen and anti-histamines.

T-DM1:

Route: Intravenous infusion is performed over 60 min

Standard Regimen: 3.6 mg/kg iv every three weeks (q3w)

Agent Preparation: Based on participant weight, the total dose is added to the iv bag. Gently invert the bag to mix the solution. Do not shake vigorously. Use within 1 hour of reconstitution with sWFI.

Pre-medication: Specific premedication is not required for routine treatment.

Administration of the Combination Pertuzumab + T-DM1:

Treatment will be administered on an outpatient basis. Pertuzumab will be administered over 60 (± 10) minutes with participants to be observed for a further 60 minutes. The infusion should be slowed or interrupted if the participant experiences infusion-related symptoms. If the infusion is well tolerated, subsequent doses may be administered over 30 (±10) minutes and participants will be observed for a further 60 minutes for infusion-related symptoms such as fever, chills. Participants should be administered T-DM1 a minimum of one hour after pertuzumab. The initial dose of T-DM1 will be administered over 60 (±10) minutes with participants to be observed for a further 30 minutes. The infusion should be slowed or interrupted if the participant experiences infusion-related symptoms. If the infusion is well tolerated, subsequent doses may be administered over 30 (±10) minutes and participants will be observed for a further 30 minutes for infusion-related symptoms such as fever, chills. The goal is to administer the agents on the same day whenever possible. Missed doses of T-DM1 and /or pertuzumab can be made up to complete 12 weeks of treatment unless otherwise specified in §2.6 Table 3.

Concomitant Medications:

No formal drug-drug interaction studies have been conducted with trastuzumab emtansine pertuzumab. In vitro metabolism studies in human liver microsomes suggest that DM1, a component of trastuzumab emtansine, is metabolized mainly by CYP3A4 and, to a lesser extent, by CYP3A5. DM1 does not induce or inhibit P450-mediated metabolism in vitro. Caution should be taken when trastuzumab emtansine is co-administered with potent CYP3A inhibitors.

Other:

CONFIDENTIAL

T-DM1 is contraindicated in participants with known hypersensitivity to any component of the product. No contraindications for pertuzumab. Also, refer to §2.6 for side effect management and dose reduction plans.

The above is intended as a summary only; please see the complete appendix for additional investigational agent information.

CONFIDENTIAL

1 RATIONALE FOR TESTING

Pertuzumab (rhuMAb 2C4), is a fully humanized monoclonal antibody, that acts by blocking the association of HER2 with other HER family members, including EGFR, HER3, and HER4, to form HER2 heterodimers [1]. Dimerization is crucial for activation of EGFR/HER receptors that contribute to tumor growth and progression. Although both pertuzumab and trastuzumab bind to the extracellular domain of HER2, they bind to distinct epitopes, and consequently, ligand-activated downstream signaling is blocked by pertuzumab but not by trastuzumab. This results in intracellular inhibition of two major signaling pathways, MAP kinase and PI3K. Inhibition of these signaling pathways can result in cell growth arrest and apoptosis, respectively [2].

Like trastuzumab, pertuzumab also stimulates antibody-dependent, cell-mediated cytotoxicity [3] but due to their complementary modes of action, the combination of pertuzumab with trastuzumab exerts greater antitumor activity than with either agent alone in HER2 overexpressing disease. In phase 2 studies the pertuzumab-trastuzumab regimen has shown activity in participants with HER2+ MBC and EBC [4, 5].

Furthermore, pertuzumab may not require HER2 overexpression to exert activity as an antitumor agent. For example, EGFR is frequently overexpressed or activated in a number of human tumors [6]. In contrast to transformation of normal cells by HER2, transformation by EGFR requires not only EGFR overexpression but also EGFR ligand expression. Because sustained intensive EGFR signaling frequently involves coordinate activation of HER2 by heterodimerization, inhibition of the HER2 component of this complex may arrest the growth of tumors driven by ligand-activated EGFR.

The role of HER3 in breast cancer is becoming increasingly recognized [7]. Studies in cell culture systems have shown that HRG-activated HER3–HER2 heterodimers elicit the strongest proliferative and transformation responses of any possible receptor combination [8]. Trastuzumab is thought to be effective in disrupting ligand independent HER2-HER3-PI3K complexes, while pertuzumab prevents ligand-induced HER2-HER3 dimerization [9]. In *in vivo* breast cancer xenograft models with nude mice, HER2/HER3 heterodimers were detected in 100% of xenografts inhibited by pertuzumab, whereas pertuzumab-resistant lines showed a much lower level of heterodimerization

T-DM1 is a novel antibody-drug conjugate (ADC) that is specifically designed for the treatment of human epidermal growth factor receptor 2-positive (HER2+) malignancies. T-DM1 is composed of trastuzumab, a humanized monoclonal antibody directed against the extracellular region to HER2; and DM1, an anti-microtubule agent derived from maytansine; combined through 4-[*N*-maleimidomethyl]cyclohexane-1-carboxylate (MCC), derived from succinimidyl MCC (SMCC), a thioether linker that conjugates DM1 to trastuzumab. On average, one molecule of trastuzumab is conjugated with 3.5 molecules of DM1 to create T-DM1, which binds to HER2 with affinity similar to that of trastuzumab. It is hypothesized that after binding to HER2, T-DM1 undergoes receptor-mediated internalization, resulting in intracellular release of DM1 and subsequent cell death [10].

DM1 is an inhibitor of tubulin polymerization; it binds to tubulin (20–100 times more potently than vincristine) to suppress microtubule dynamics, thereby blocking mitotic progression, resulting in cell death presumably from direct mitotic death or via apoptosis [11, 12]. Its parent molecule, maytansine, was studied in approximately 800 participants who were administered maytansine q3w either as a single dose or for three consecutive days. Responses were seen in participants with breast and lung cancer; however, because of its narrow therapeutic index, clinical development was not continued [13].

Overexpression of HER2 is observed in approximately 15%–20% of human breast cancers. Several lines of scientific and clinical evidence support a direct role for HER2 overexpression in the aggressive growth and poor clinical outcomes associated with these tumors [14]. The development of trastuzumab in the

CONFIDENTIAL

1990s provided women with HER2-overexpressing tumors a markedly better outcome than was possible with chemotherapy alone. Increases in response rate, response duration, and PFS were associated with a five-month survival advantage when given in the first-line metastatic setting as demonstrated in the initial phase 3 trials [15]. The use of adjuvant trastuzumab in HER2+ EBC improves participant outcomes as demonstrated in several randomized trials, reducing the risk of relapse by about 50% and the risk of death by about 30%. Trastuzumab is also used pre-operatively in combination with chemotherapy for participants with HER2+ LABC (stage IIb–IIIc)and/or in cases where participants wish to minimize the extent of breast cancer surgery [10].

Despite advances in therapy for HER2+ early breast cancer, a significant proportion of participants will develop recurrence of disease and new treatment options are needed. As an antibody-drug conjugate, upon binding to HER2 on cancer cells, T-DM1 is internalized through receptor-mediated endocytosis [16] and active DM1 is subsequently released inside the cells [17]. Directing DM1, a potently cytotoxic agent, via a highly specific antibody, trastuzumab, to cancer cells is expected to minimize its effect on normal cells, thereby overcoming systemic toxicity associated with DM1 and reducing dose-limiting toxicity that hinders its clinical use [18–21]. Furthermore, given the complementary actions of Pertuzumab and Trastuzumab, the development of a combination regimen of Pertuzumab + T-DM1 has the potential to optimize therapy for HER2+ disease by enhancing intracellular inhibition of multiple pathways, enhancing ADCC and targeting an effective cytotoxic, DM1, directly to tumor cells overexpressing Her2/neu. The favorable toxicity profiles of both Pertuzumab and T-DM1 further support the combination of these agents for clinical investigation.

1.1 Efficacy

1.1.1 *In Vitro* Studies and Mechanistic Studies

The antiproliferative activity of T-DM1 has been evaluated in human breast cancer cells with high and low levels of HER2 expression. These studies demonstrate that the antiproliferative activity of T-DM1 is most pronounced against cells with high HER2 expression. In addition, T-DM1 shows greater anti-proliferative activity than trastuzumab against trastuzumab-sensitive breast cancer cells, and is highly potent on trastuzumab-insensitive cell lines. In comparison to trastuzumab, which shows cytostatic activity, T-DM1 induces cell death in HER2-overexpressing breast cancer cells [10].

Similar to trastuzumab, T-DM1 inhibits shedding of the HER2 extracellular domain, suppresses phosphorylation of Akt, and mediates antibody-dependent cellular cytotoxicity in HER2-overexpressing breast cancer cell lines. T-DM1 also exhibits activity against lapatinib-resistant cell lines and tumors [22].

1.1.2 Animal Studies

Antitumor activity of T-DM1 was evaluated infour independent models of HER2+ human cancers, including three xenograft models using human tumor cell lines. BT-474 EEI is a trastuzumab-resistant, estrogen-independent subline of the HER2+ breast cancer cell line BT-474, while KPL-4 is a HER2+ breast cancer cell. The third model (fo5) is a transgenic mouse model in which the human HER2 gene, under transcriptional regulation of the murine mammary tumor virus promoter, is overexpressed in mammary epithelium, causing spontaneous development of mammary tumors that overexpress the human HER2 receptor. This tumor model is insensitive to trastuzumab. All in *vivo* antitumor efficacy studies were designed to test the activity of T-DM1 against established tumors (*i.e.,* tumors were allowed to grow to a size of 150–300 mm^3 before treatment was initiated). T-DM1 activity was observed in all four models with an efficacious dose range of 3–15 mg/kg, depending on the sensitivity of the particular model [10].

CONFIDENTIAL

A study in the fo5 HER2-transgenic trastuzumab-insensitive model compared the efficacy of T-DM1 to free DM1, trastuzumab alone, or trastuzumab and DM1 used as individual agents. Antitumor activity was observed only with trastuzumab emtansine, confirming the specific activity of T-DM1, compared with the individual components. Additional *in vivo* studies showed that no antitumor activity was observed using T-DM1 against a HER2-negative (HER2−) tumor, or using a control ADC (anti-IL8-MCC-DM1), demonstrating that antigen-specific recognition of tumor cells is required for antitumor efficacy. In the fo5 model, significant tumor growth inhibition was observed with 10 mg/kg T-DM1, and tumor regression with 15 and 30 mg/kg dosed q3w for a total of three doses [10].

In the BT-474 EEI breast cancer model, T-DM1 given every three weeks for a total of three doses showed significant inhibition of tumor growth at the 3 mg/kg dose; tumor stasis was observed at 10 mg/kg and tumor regression at 15 mg/kg. In comparison, trastuzumab given at 15 mg/kg showed minimal tumor growth inhibition. In the MCF7-neo/HER2 model, significant tumor growth inhibition was observed with a single injection of trastuzumab emtansine at doses between 3-10 mg/kg [10].

The efficacy of T-DM1 compared with trastuzumab was tested in the KPL-4 trastuzumab-sensitive mammary tumor model. KPL-4 cells display a phosphatidylinositol-3-kinase (PI3K) mutation (H1047R) which renders them insensitive to the anti-signaling effects of trastuzumab in cell culture studies. However, KPL-4 xenograft tumors respond to trastuzumab due to trastuzumab-mediated antibody-dependent cellular cytotoxicity. Weekly doses of 15 mg/kg trastuzumab caused regression of KPL-4 mammary tumors; however, tumors showed rapid re-growth upon cessation of treatment. In contrast, a single administration of 15 mg/kg T-DM1 resulted in sustained tumor regressions throughout the course of the study (125 days) [10].

In cell culture and mouse xenograft models, the combination of T-DM1 with pertuzumab was more efficacious than single-agent T-DM1 [10].

1.1.3 Human Studies

As of September 30, 2014, fifteen studies with T-DM1 had been completed in breast cancer, and a further 13 studies in participants with breast cancer were ongoing [10].

Completed studies assessing the safety and efficacy of single agent T-DM1 in participants with MBC include the dose-finding phase 1 study TDM3569g [23], two single-arm phase 2 studies TDM4258g [24] and TDM4374g, study TDM4688g, which evaluated effects of T-DM1 on the QT interval, the randomized Phase 2 study TDM4450g comparing single agent T-DM1 with trastuzumab plus docetaxel and the open-label, expanded access study TDM4884g. Also completed are three studies assessing T-DM1 in combination- a phase 1b/2 study TDM4373g/BO22495 which evaluated combination of T-DM1 plus pertuzumab in MBC/LABC, a single-arm Phase Ib/IIa dose-finding study TDM4652g/GO01355, evaluating T-DM1 combined with paclitaxel with or without pertuzumab and a single-arm Phase Ib/IIa dose-finding study BP22572, evaluating T-DM1 combined with docetaxel with or without pertuzumab in patients with LABC or MBC. Among Japanese participants with MBC, a phase 1 study (JO22591) and a phase 2 study (JO22997) of T-DM1 monotherapy as well as a phase 1b study of T-DM1 and pertuzumab (JO22992) have been completed.

Efficacy and safety data are also available from Study TDM4997g/BO25734 (TH3RESA) whichis evaluating trastuzumab emtansine versus Treatment of Physician's Choice (TPC; standard treatment with chemotherapy, hormone therapy and/or biologic agents a and an ongoing Phase III study evaluating single-agent trastuzumab emtansine versus capecitabine plus lapatinib (TDM4370g/BO21977; EMILIA [37]) [25].

Finally, a recent report suggests that treatment with T-DM1 does not exhaust the potential benefit of continuing second line or salvage anti-HER2 therapies [26]. Twenty MBC participants, all heavily pretreated with trastuzumab-based therapies, first initiated and then discontinued T-DM1 therapy for progression or toxicity. Upon reinitiation of further therapy, 33% of those that underwent trastuzumab or lapatinib-based retreatments experienced partial responses with durations ranging from 5.5–6.4 months.

1.1.3.1 Efficacy of T-DM1 Monotherapy

The efficacy of single-agent T-DM1 (3.6 mg/kg q3w) has been evaluated in phase 2 and 3 clinical trials. Study TDM4258g enrolled participants with HER2+ MBC who had progressed on previous HER2-directed therapy, whereas participants in study TDM4374g had disease progression after at least two HER2-directed therapies (*i.e.,* trastuzumab and lapatinib) in the metastatic or locally advanced setting. In both of these studies, the primary efficacy endpoint was objective response as assessed by independent review of tumor assessments. The clinical activity of T-DM1 was similar in the two studies, with an ORR of 26% in TDM4258g and 32% in TDM4374g. No participant in either study achieved a CR.

Efficacy was also assessed in study TDM4688g, a single-arm phase 2 study designed to evaluate the effect of T-DM1 (3.6 mg/kg q3w) on the duration of the QTc interval in participants with recurrent HER2+ LABC or MBC who had progressed on trastuzumab. Objective response was assessed by the investigator. Thirteen participants (25.5%) achieved a response with T-DM1 monotherapy; five had a CR and eight had a PR.

Study TDM4450g was a randomized, open-label, phase 2 study comparing T-DM1 (3.6 mg/kg q3w) with trastuzumab plus docetaxel combination therapy as first-line treatment for participants with HER2+ MBC. Analyses of PFS, the primary efficacy parameter, were based on investigator assessments; other secondary efficacy parameters, were performed based on a data cut-off date of 15 November 2010, with the exception of OS which was based on a cut-off date of 31 August 2011 . A significant improvement in PFS was observed for participants treated with T-DM1 compared with participants receiving trastuzumab plus taxane (median PFS 14.2 *vs*. 9.2 months; hazard ratio =0.594; 95% CI: 0.364–0.968; log-rank test p=0.0353). The ORR was similar for the two treatment arms, but a longer duration of response was observed in participants treated with T-DM1 (median duration not reached *vs*. 9.5 months for trastuzumab plus taxane). OS rates were similar between treatment arms (HR = 1.06; 95% CI: 0.477, 2.352), however the data are limited by the overall sample size and the small number of deaths in each arm.

Study BO21977/TDM4370g (EMILIA) is a randomized, open-label, phase 3 study comparing T-DM1(3.6 mg/kg q3w) with lapatinib plus capecitabine in participants with unresectable HER2+ LABC or MBC previously treated with trastuzumab and a taxane. A total of 991 participants were enrolled. PFS was performed based on data cut-off date of January 14, 2012 (approximately 13 month median follow-up). Significant improvement in PFS, as assessed by independent review, was observed in participants treated with T-DM1 compared with participants treated with lapatinib plus capecitabine (median PFS 9.6 *vs*. 6.4 month; hazard ratio =0.65; 95% CI:0.55–0.77). OS was performed based on data-cutoff date of July 31, 2012 (approximately 19 month median follow-up). T-DM1 significantly increased OS compared with lapatinib plus capecitabine (median OS 30.9 *vs.* 25.1 month; hazard ratio 0.68; 95% CI=0.55-0.85). Estimated 1-year survival rates were 85.2% *vs.* 78.4 for participants treated with T-DM1 and lapatinib plus capecitabine respectively [25].

1.1.3.2 Efficacy of T-DM1 Combined with Pertuzumab in LABC/MBC

CONFIDENTIAL

The efficacy of T-DM1 when combined with pertuzumab has been evaluated in study TDM4373g and a subgroup of participants from study TDM4688g. In both studies, pertuzumab was administered at a loading dose of 840 mg (in Cycle 1) and at a maintenance dose of 420 mg in subsequent cycles.

Study TDM4373g was a phase 1b/2 study of T-DM1 plus pertuzumab in participants with HER2+ LABC (unresectable local or regional) or MBC. Of the 67 participants enrolled, 46 participants had received prior treatment for recurrent LABC or MBC and had relapsed or progressed on a prior HER2-directed therapy; 21 had not received any prior treatment for recurrent locally advanced or metastatic disease (first-line participants). The safety and tolerability of T-DM1 in combination with pertuzumab was first established in three participants at a T-DM1 dose of 3.0 mg/kg. Subsequent participants were treated with 3.6 mg/kg T-DM1 plus pertuzumab. The overall ORR was 41%; however, the ORR was higher among participants who had not received prior treatment for MBC (57%) than in those who had progressed after receiving prior therapy for advanced disease (33%).

Participants in study TDM4688g who developed early disease progression while receiving single agent T-DM1 (*i.e.*, within six cycles or 18 weeks from study start) were eligible to receive combination treatment with T-DM1 (3.6 mg/kg q3w) plus pertuzumab in subsequent cycles. Twenty of the 51 participants in the study were treated with combination therapy; two participants achieved a PR.

1.1.3.3 Efficacy of Trastuzumab Emtansine combined with Taxanes in LABC/MBC

The Phase Ib (dose finding)/IIa study, TDM4652g/GO01355, evaluated trastuzumab emtansine in combination with paclitaxel with or without pertuzumab, in patients with LABC or MBC. In the Phase IIa portion of the study the ORR was 47.6% in patients treated with trastuzumab emtansine plus paclitaxel (n = 22) and was 52.4% in patients treated with trastuzumab emtansine plus paclitaxel and pertuzumab (n = 22). The median duration of response was not estimable in either patient group. Study BP22572 was a non-randomized, non-stratified Phase Ib/IIa study assessing trastuzumab emtansine in combination with docetaxel (doublet regimen), in patients with LABC (previously untreated) or MBC. For patients with LABC, pertuzumab could be added to trastuzumab emtansine and docetaxel (triplet regimen). In the MBC population (n = 25), the ORR was 80.0% (95% CI: 59.3; 93.2). In the overall LABC population (n = 73), the pathological complete response (pCR) rate was 60.3% (95%CI: 48.1, 71.5). In LABC patients receiving the doublet regimen (n = 40), the pCR rate was 60.0%, and in those receiving the triplet regimen (n = 33), it was 60.6 %.

Table 1.1 Completed Clinical Trials with T-DM1 [10, 27]

Phase/Status	Cohort	Study Size	Dose/Other Treatments	No. of Sites	Protocol ID/Reference
**Studies in Metastatic Breast Cancer**					
1/Complete, CSR available	HER2+ MBC with progression on/within 60 days of trastuzumab; prior chemotherapy for MBC required	52	Three-weekly dosing: 0.3, 0.6, 1.2, 2.4, 3.6, 4.8 mg/kg iv; weekly dosing: 1.2, 1.6, 2.0. 2.4, 2.9 mg/kg iv	NA	TDM3569g US only/ NCT00932373
2/Complete, CSR available	HER2+ MBC with progression on trastuzumab; prior chemotherapy for MBC required	112	3.6 mg/kg iv q3w until PD or unacceptable toxicity	40	TDM4258g US only/ NCT00509769

CONFIDENTIAL

2/Complete, CSR available	HER2+ MBC with progression on at least 2 lines of HER2-directed therapy (trastuzumab and lapatinib)	110	3.6 mg/kg iv q3w until PD or unacceptable toxicity	NA	TDM4374g/ US only/ NCT00679211
2/Complete, CSR available	HER2+ LABC or MBC with recurrence or progression on trastuzumab	51	T-DM1 (as single agent or combined with pertuzumab): 3.6 mg/kg iv q3w; pertuzumab: 840 mg/kg iv (loading dose) then 420 mg/kg iv q3w	NA	TDM4688g US only/ NCT00943670
1b/2, Complete, Final CSR available	HER2+ LABC or MBC; prior treatment with HER2-directed therapy permitted but not required	67	T-DM1: 3.6 mg/kg iv q3w; pertuzumab: 840 mg/kg iv (loading dose), followed by 420 mg/kg iv q3w	NA	TDM4373g/ BO22495 Global/ NCT00875979
2/Complete, CSR Available	HER2+ MBC; no prior chemotherapy or HER2-directed therapy for MBC	137	Arm A: T-DM1 3.6 mg/kg iv q3w; Arm B: trastuzumab 8 mg/kg iv (loading dose) then 6 mg/kg iv + docetaxel 75 or 100 mg/m^2 IV q3w	65	TDM4450g/ BO21976 Global/ NCT00679341
1b/Completed. Final CSR available	Previously-treated HER2+ LABC or MBC; prior treatment with trastuzumab	Phase Ib: 60 Phase IIa: 44	T-DM1: 2.4, 3.6 mg/kg q3w, 2.0, 2.4mg/kg qw; paclitaxel: 65, 80 mg/m^2 qw; pertuzumab: 840 mg/kg iv (loading dose)	5	TDM4652g/GO01355 US only/ NCT00951665
Phase 4, expanded access/Completed CSR available	HER2+ LABC or MBC; prior treatment with at least two prior regimens of HER2-directed therapy	335	3.6 mg/kg iv q3w	15	TDM4884g/ML01356 US only/ ML01356 (T-PAS)/ NCT01120561
2/Completed. Final CSR available	Early stage HER2+ breast cancer	153	T-DM1:3.6 mg/kg iv q3w	44	TDM4874g/ BO22857 Global/ NCT01196052
1/Complete, CSR in preparation	Previously-treated HER2+ recurrent breast cancer; prior treatment with trastuzumab required	56	T-DM1: 3.6 mg/kg iv q3w; GDC-0941:80 mg qd, days 1–14	3	GDC4627g US only/ NCT00928330

CONFIDENTIAL

1b/2, Complete Final CSR available	HER2-positive, operable or inoperable LABC or MBC; prior HER2- directed therapy not required	98	T-DM1: 2.4 or 3.6 mg/kg iv q3w; docetaxel: 60, 75, or 100 mg/m² q3w; pertuzumab: 840 mg/kg iv (loading dose), then 420 mg/kg iv q3w	8	BP22572 France, Spain, US, UK/ NCT00934856

Table 1.1 Completed Clinical Trials with T-DM1 [10, 27]

Phase/Status	Cohort	Study Size	Dose/Other Treatments	No. of Sites	Protocol ID/Reference
1/Complete, CSR available	Previously-treated HER2+ advanced or recurrent breast cancer	10	T-DM1, cycle 1: 1.8, 2.4 or 3.6 mg/kg q3w	NA	JO22591 Japan only (Chugai study)
1b/Complete, CSR available	Previously-treated HER2+ advanced or recurrent breast cancer	6	T-DM1: 3.6 mg/kg iv q3w; pertuzumab: 840 mg/kg iv (loading dose) then 420 mg/kg iv q3w	NA	JO22992 Japan only (Chugai study)
2/Complete, CSR available	HER2+ LABC/recurrent or MBC	76	T-DM1: 3.6 mg/kg iv q3w	NA	JO22997 Japan only (Chugai study)
2/Complete, CSR in preparation	HER2-positive locally advanced/recurrent or metastatic breast cancer	234	T-DM1: 3.6 mg/kg IV q3w	234	JO29317 Japan Only (Chugai study)

CSR: clinical study report; HER2+: human epidermal growth factor receptor 2-positive; iv: intravenous; LABC: locally advanced breast cancer; MBC: metastatic breast cancer; NA: not available; PD: progressive disease; qw: once weekly; q3w: once every three weeks; T-DM1: trastuzumab emtansine
* T-DM1 parent studies: TDM3569g, TDM4258g, TDM4374g, TDM4450g, TDM4373g/BO22495, TDM4688g, BP22572, TDM4884g, BO25499

CONFIDENTIAL

1.2 Ongoing/Planned Studies

In addition to the completed studies discussed above, 9 studies in participants with MBC/LABC and 3 studies in EBC are ongoing; a study in participants with advanced gastric cancer is open [10].

Phase 3 studies are ongoing to evaluate single agent T-DM1 *vs.* capecitabine plus lapatinib (BO21977/TDM4370g), and *vs.* standard treatment with chemotherapy, hormone therapy, and/or biologic agents (TDM4997g/BO25734).

The combination of T-DM1 plus pertuzumab is currently being assessed in three ongoing trials: In the metastatic setting, T-DM1 treatment with and without pertuzumab is being evaluated *vs.* trastuzumab plus taxane (docetaxel or paclitaxel) in an ongoing phase 3 study (“MARIANNE”BO22589/TDM4788g). [28]. A two-arm Phase 3 trial assessing T-DM1 treatment plus pertuzumab (KAITLIN, BO28407) following anthracyclines compared to trastuzumab plus pertuzumab plus a taxane in HER2+ operable breast cancer is ongoing. Additionally, a two arm study of T-DM1 plus pertuzumab compared to trastuzumab, pertuzumab plus chemotherapy (docetaxel/carboplatin) in HER2+ operable breast cancer is ongoing (KRISTINE, BO28408)

A phase 3 randomized study of T-DM1 compared to trastuzumab plus docetaxel as first line treatment for HER2+ progressive breast cancer is ongoing.(YP28405, KAILEE).
The safety of T-DM1 in EBC is being investigated in three phase III studies: BO20738 (KATHERINE), BO28408 (KRISTINE) and BO28407 (KAITLIN). All are in recruitment phase.
A Phase 3 study to determine the efficacy and safety of T-DM1 monotherapy in locally advanced or metastatic breast cancer is ongoing (MO28231, KAMILLA), and a study of T-DM1 monotherapy in advanced gastric cancer (BO27952, GATSBY) is ongoing.

Table 1.2. Ongoing/Planned Clinical Trials with T-DM1 [10, 27]

Phase/Status	Cohort	Study Size	Dose/Other Treatments	No. of Sites	Protocol ID/NCT No.
**Studies in Metastatic Breast Cancer**					
3/Ongoing	HER2+ LABC or MBC; prior taxane and trastuzumab required	991	Arm A: T-DM1 3.6 mg/kg iv q3w Arm B: lapatinib 1250 mg/day po qd and capecitabine 1000 mg/m^2 po bid. on days 1–14 of a 21-day cycle	324	TDM4370g/ BO21977 Global/ NCT00829166 (EMILIA)
Extension study of parent studies*/Ongoing	HER2+ LABC or MBC; T-DM1 in parent study, or control arm therapy (H+D) in TDM4450g (without PD)	Planned: 700	3.6 mg/kg iv q3w	NA	TDM4529g/ BO25430 Global/ NCT00781612
3/Ongoing	HER2+ progressive or recurrent LABC or previously untreated HER2+ MBC	1095	T-DM1: 3.6 mg/kg iv q3w; pertuzumab: 840 mg/kg iv (loading dose), then 420 mg/kg iv q3w; trastuzumab: 8 mg/kg iv (loading dose) then 6 mg/kg iv q3w + docetaxel 75 or 100 mg/m^2 q3w, or trastuzumab: 4 mg/kg iv (loading dose) then 2 mg/kg iv qw + paclitaxel 80 mg/m^2 qw	294	BO22589/ TDM4788g Global/ NCT01120184 (MARIANNE)
3/Ongoing	HER2+ MBC; prior treatment with at least two regimens of HER2-directed therapy for MBC	602	Arm A: T-DM1 3.6 mg/kg iv q3w; Arm B: physician's choice (includes chemotherapy, hormone therapy, HER2-directed therapy)	184	TDM4997g Global/ BO25734/ NCT01419197 (TH3RESA)

CONFIDENTIAL

Table 1.2. Ongoing/Planned Clinical Trials with T-DM1 *(continued)*					
**Phase/Status**	**Cohort**	**Study Size**	**Dose/Other Treatments**	**No. of Sites**	**Protocol ID/NCT No.**
PK study/Ongoing	HER2+ MBC; prior treatment allowed but not required; mild or moderate hepatic impairment or normal liver function	Planned: 28	T-DM1:<br>3.6 mg/kg iv q3w	13	BO25499 Global/ NCT01513083
1/2; Ongoing	HER2-positive mBC and LABC<br>MGC (untreated in metastatic setting)	Planned: Up to 128	T-DM1: MBC - 3.6 mg/kg IV q3w; MGC - 2.4 mg/kg qw.<br>Capecitabine: escalated 750- 1000 mg bid	3	MO28230 (TRAX-HER2)/ NCT01702558
3b/Ongoing	HER2-positive mBC and LABC prior treatment with an anti-HER2 agent and chemotherapy; All lines	Planned: 2000	T-DM1:<br>3.6 mg/kg IV q3w	286	MO28231 (KAMILLA)/ NCT01702571
4/Ongoing	Third-line HER2-positive, MBC	Planned: 335	T-DM1: 3.6 mg/kg IV q3w until PD	15	TDM4884g/ ML01356 (T-PAS)/ NCT01120561
3/Ongoing	HER2- positive, unresectable, locally advanced BC (recurrent disease or PD despite primary multimodality therapy) and/or MBC (no prior chemotherapy for MBC)	Planned: 561	T-DM1:<br>3.6 mg/kg IV q3w<br>Trastuzumab: loading dose of 8 mg/kg, then 6 mg/kg IV q3w.<br>Docetaxel: 75-100 mg/m2 q3w		YP28405 (KAILEE) Global
**Studies in Early Breast Cancer**					
3/Ongoing	Residual HER2+ primary breast cancer after pre-operative therapy	Planned: 1484	Arm A: T-DM1 3.6 mg/kg iv q3w for 14 cycles; Arm B: trastuzumab 8 mg/kg iv (loading dose) followed by 6 mg/kg iv q3w for 14 cycles	NA	BO27938 Global/ NCT01772472 (KATHERINE)
3/Ongoing	Treatment naïve, operable, locally advanced or inflammatory HER2 positive breast cancer	Planned: 432	T-DM1: 3.6 mg/kg IV q3w + Pertuzumab: Loading dose of 820 mg then 420 mg IV q3w.<br>Trastuzumab: Loading dose of 8 mg/kg, then 6 mg/kg IV q3w.<br>Carboplatin: AUC 6	NA	B028408 (KRISTINE) Global NCT02131064

CONFIDENTIAL

			Docetaxel: 75mg/m2		
2/Ongoing	Newly diagnosed HER2+ breast cancer that will be treated with adjuvant systemic chemotherapy following definitive surgery.	Planned: 2500	T-DM1:<br>3.6 mg/kg IV q3w<br>Pertuzumab: Loading dose of<br>820 mg then 420 mg IV q3w.<br>Trastuzumab: Loading dose of 8<br>mg/kg, then 6 mg/kg IV q3w.<br>Docetaxel: 75-100 mg/m2 q3w<br>OR paclitaxel: 80 mg/m2 qw	NA	BO28407 (KAITLIN) Global NCT01966471
**Studies in Gastric Cancer**					
2/3; Ongoing	HER2+ locally advanced or metastatic gastric cancer, or adenocarcinoma of the GEJ; prior systemic therapy required	Planned: 412 global study, 225 China,	Arm A: T-DM1 3.6 mg/kg iv q3w Arm B: T-DM1 2.4 mg/kg iv q3w Arm C: Docetaxel 75 mg/m2 q3w or Paclitaxel 80 mg/m2 qw	130	BO27952 (GATSBY) Global/ NCT01641939

GEJ: gastroesophageal junction; HER2+: human epidermal growth factor receptor 2-positive; iv: intravenous; LABC: locally advanced breast cancer; MBC: metastatic breast cancer; NA: not available; PD: progressive disease; PK: pharmacokinetic; po: oral; qd: daily; qw: once weekly; q3w: once every three weeks; T-DM1: trastuzumab emtansine
* T-DM1 parent studies: TDM3569g, TDM4258g, TDM4374g, TDM4450g, TDM4373g/BO22495, TDM4688g, BP22572, TDM4884g, BO25499

1.3 Toxicity

1.3.1 Human Studies

The safety profile of T-DM1 in MBC is based on completed studies with single-agent treatment in 884 participants (from studies TDM4258g, TDM4374g, TDM4688g,TDM4529g, TDM4450g, and TDM4370g) and combined treatment with pertuzumab in 87 participants (from studies TDM4373g/BO22495 and TDM4688g) with a clinical cutoff date of 31 July 2012. There are no major safety concerns for the currently ongoing clinical trials of T-DM1 as monotherapy, or in combination with pertuzumab or taxanes [10].

1.3.1.1 Safety in Completed Studies

T-DM1 Monotherapy

The safety profile of T-DM1 monotherapy is derived from a pooled analysis of 884 participants treated at 3.6 mg/kg q3w in seven completed studies (TDM4258g, n=112; TDM4374g, n=110; TDM4688g, n=51; TDM 4370g/BO21977 [EMILIA], n=490; TDM4450g/BO21976g, n=106; TDM3569g, n=15), including follow-up data for participants from these studies who continued to receive treatment in the extension study (TDM4529g, n=43)[29].

The most common AEs associated with T-DM1 monotherapy were fatigue (46.4%), nausea (43.0%), thrombocytopenia (29.6%), headache (29.4%), and constipation (26.5%). The vast majority of these events were grade 1 or 2 in intensity. Thrombocytopenia, which was identified as the primary DLT in the

CONFIDENTIAL

phase 1 study, was recorded in approximately 30% of participants. The most common grade ≥3 AEs (occurring in more than 2% of participants) were thrombocytopenia (10.7%), increased AST (4.3%), fatigue (3.2%), increased ALT (3.1%), hypokalemia (2.9%), and anemia (2.7%). 62 participants (6.6%) experienced AEs resulting in discontinuation of their T-DM1 treatment, most commonly due to laboratory abnormalities with thrombocytopenia (1.5%) and increased hepatic transaminase (AST, 0.8%; ALT, 0.5%). Cases of nodular regenerative hyperplasia (NRH) of the liver have been identified from liver biopsies in patients presenting with signs and symptoms of portal hypertension. There has been one case of fatal liver failure in a participant with NRH.

As of data cut-off date July 31, 2012, 171 patients (19.3%) experienced an SAE during treatment with single-agent T-DM1, with no single predominant SAE. SAEs recorded by more than 5 patients were pneumonia (1.7%), pyrexia (1.4%), cellulitis (1.1%), vomiting and thrombocytopenia (each 0.9%); convulsion, dyspnea (each occurring in 0.8%); abdominal pain, sepsis, back pain (each occurring in 0.7%); and pleural effusion (0.6%).

Study TDM4450g/BO21976 is a randomized controlled study comparing T-DM1 monotherapy with trastuzumab plus docetaxel as a first-line treatment in participants with HER2+ unresectable LABC or MBC. Preliminary safety analyses were performed based on a data cut-off date of May 24, 2012. Of the 70 participants assigned to treatment with trastuzumab plus docetaxel, two received no treatment, and a further two participants mistakenly received a dose of T-DM1 and so were included in the T-DM1 group for safety analyses.

The incidence of grade ≥3 AEs was lower in participants treated with T-DM1 than in those receiving trastuzumab plus docetaxel (46% *vs*. 91%). The most common grade ≥3 AEs in the T-DM1 group (occurring in >5% of participants) were increased ALT (10.1%), increased AST (8.7%), thrombocytopenia (7.2%), and pneumonia (7.2%).

One participant in the T-DM1 group died as a result of an AE (sudden death); the relationship of this event with study treatment in inconclusive. One participant in the trastuzumab plus docetaxel group died due to cardiopulmonary failure.

SAEs were recorded in 20.3% of participants in the T-DM1 group and 27.3% of participants in the trastuzumab plus docetaxel group. The most frequent SAEs during T-DM1 treatment were pneumonia (five participants, 7.2%); all other events occurred only once.

Five participants (7.2%) had their T-DM1 treatment discontinued due to an AE: increases in both AST and ALT (three participants), thrombocytopenia (one participant), and eye pain and ocular hyperemia (one participant). In comparison, 23 participants (34.8%) in the trastuzumab plus docetaxel group had a component of their study treatment discontinued as a result of an AE (most commonly peripheral neuropathy/peripheral sensory neuropathy, recorded in seven participants, 10.6%).

Sixteen participants treated with single agent T-DM1 developed asymptomatic decreases from baseline LVEF of at least 15% concomitant with LVEF <50% and all but two continued treatment without delay or dose reduction.

The safety profile of T-DM1 was different from that of trastuzumab plus docetaxel, as shown by the incidences of some common AEs. Thrombocytopenia (19 participants, 27.5%) and increases in AST (30 participants, 43.5%) occurred more frequently in the T-DM1 group, while alopecia (44 participants, 66.7%), peripheral edema (29 participants, 43.9%), and neutropenia (43 participants, 65.2%) were much more prevalent in the control group.

CONFIDENTIAL

T-DM1 Plus Pertuzumab

The safety profile of T-DM1 in combination with pertuzumab is derived from 87 participants from study TDM4373g (n=67) and a subset of participants from study TDM4688g (n=20), including follow-up data for any participants continuing to receive treatment in the extension study (TDM4529g). The most common AEs were fatigue (52.9%), nausea (42.5%), diarrhea (35.6%), and cough (33.3%), with most of these events being grade 1 or 2. The most common grade ≥3 AEs (occurring in more than two participants, 2.5%) were fatigue (11.5%), thrombocytopenia (10.3%), increased AST (9.2%), dyspnea (9.2%), increased ALT (6.9%), anemia (5.7%), cellulitis (4.6%), peripheral sensory neuropathy (4.6%), hypokalemia (4.6%), pleural effusion (3.4%) and pneumonia (3.4%). Eighteen participants (20.7%) experienced AEs which resulted in discontinuation of their T-DM1 treatment; apart from fatigue (four participants, 4.6%) and increased AST (two participants, 2.3%), all AEs leading to treatment discontinuation were single occurrences.

SAEs were recorded for 28 participants (32.2%) during treatment with T-DM1 plus pertuzumab. The majority of these events were singular occurrences. SAEs recorded by more than one participant (*i.e.*, >1%) included dyspnea (4.6%); pleural effusion, cellulitis, pneumonia (each occurring in 3.4% of participants); and anemia, abdominal pain, nausea, vomiting, and pyrexia (each occurring in 2.3% of participants). One participant died as a result of an AE (pneumonia) following treatment with T-DM1 plus pertuzumab.

These data show that the addition of pertuzumab did not significantly alter the safety profile of T-DM1. There were no additional safety concerns with the combination of T-DM1 plus pertuzumab; however, the incidences of grade ≥3 AEs and SAEs were higher in participants on combination therapy (grade ≥3 AEs, 59.8% *vs*. 44.2% and SAEs, 32.2% *vs.* 19.3% for monotherapy *vs.* combination therapy, respectively) [30].

T-DM1 plus Taxane
In study BP22572, TDM1 was evaluated in combination with docetaxel with or without pertuzumab. In patients with MBC (n = 25), the most common AEs (in at least 50% of patients) were:neutropenia (76%), asthenia (72%), thrombocytopenia (64%), epistaxis (56%), and leukopenia (52%). The most common Grade ≥ 3 AEs were neutropenia (72%) and leukopenia (44%). Overall 80% (i.e., 20/25) of patients had Grade ≥ 3 AEs and 40% (10/25) of patients had SAEs. Five patients (20%) discontinued treatment, most commonly as a result of thrombocytopenia. There were no deaths in this patient group. In the overall LABC population (n = 73), the most common AEs experienced in LABC patients were: asthenia (62%), epistaxis (55%), mucosal inflammation (49%), alopecia (44%), dysgeusia (44%), nausea (44%), and increased lacrimation (42%). The majority of Grade ≥ 3 AEs were related to laboratory parameters (hematological and hepatotoxicity-related), with the most common AEs being neutropenia (29%), increased ALT (15%), and thrombocytopenia (12%). 64% of patients had Grade ≥ 3 AEs and 25% of patients had SAEs. A total of 14% discontinued treatment due to an AE (the most common event was increased ALT). One patient, died as a result of respiratory failure due to pneumonitis, as noted below.

T-DM1 in Early Breast Cancer

Safety data are available for T-DM1 monotherapy after anthracycline treatment in participants with EBC in study BO22857/TDM4874g, based on a clinical cut-off date of 12 June 2013. Co-primary endpoints were safety and the rate of prespecified cardiac events (defined as death from a cardiac cause or severe CHF [NYHA Class III or IV] with a decrease in LVEF of ≥ 10 percentage points from baseline to an LVEF of < 50%) occurring within the first 12 weeks of trastuzumab emtansine treatment. There were no pre-specified cardiac events and no AEs of left ventricular dysfunction or heart failure. Four patients

CONFIDENTIAL

(2.7%) had asymptomatic LVEF declines to < 50% and maximum decrease of at least 10 percentage points from baseline; one of these patients discontinued trastuzumab emtansine. The LVEF declines for the other three patients were considered by the investigators to be unrelated to trastuzumab emtansine, and in two patients the LVEF decline occurred following trastuzumab treatment.
The most common AEs (in at least 20% of patients) while receiving trastuzumab emtansine treatment were: nausea (37.8%), headache (37.2%), epistaxis (32.4%),asthenia (30.4%), and pyrexia (26.4%), fatigue (23.0%), arthralgia (22.3%), thrombocytopenia (21.6%) and myalgia (20.9%). A total of 61 patients (41.2%) had Grade ≥ 3 AEs while receiving trastuzumab emtansine: the most common events (≥ 2%) reported were thrombocytopenia (8.1%), ALT increase (7.4%), AST increase (7.4%), neutropenia (5.4%), and hypertension (2.7%), fatigue (2.0%) and hypokalemia (2.0%). Fifteen patients (10.1%) experienced an SAE; events reported in more than one patient were atrial fibrillation, pyrexia, and device related infection (each in 2 patients). No patients died in this study. A total of 20 patients (13.5%) experienced an AE that led to the discontinuation of trastuzumab emtansine. The most commonly affected system organ class was blood and lymphatic system disorders (primarily thrombocytopenia). ; [10, 31].

AEs of Special Interest

Events considered to be of special interest due to their observed frequency and/or clinical relevance include thrombocytopenia, hepatotoxicity, peripheral neuropathy, infusion reactions, hypokalaemia, cardiac dysfunction, peripheral neuropathy, infusion-related reactions/hypersensitivity, cardiac toxicity (left ventricular dysfunction), and pulmonary toxicity (cases of interstitial lung disease such as pneumonitis)[10]. These are described in further detail in §3.2. Neutropenia and anemia have been observed in clinical trials of trastuzumab in combination with chemotherapy, and extravasation was observed in clinical trials with maytansine; these cannot be excluded as potential risks with T-DM1. Fetal harm is considered a potential risk with T-DM1 exposure; there have been two reports of pregnancy during T-DM1 use to date, although no studies of T-DM1 in pregnant women have been conducted. [10].

AE's Leading to Death

In the pooled analysis of single agent T_DM1, twelve participants receiving single-agent T-DM1 have died for reasons other than disease progression.
In six cases (hepatic failure in study TDM4374g and bacterial sepsis in study TDM4529g, hepatic function abnormality, and metabolic encephalopathy), the event was considered by the investigator to be related to T-DM1; the remaining six events were considered to be unrelated to study treatment. Seven additional deaths, assessed by the investigator to be related to T-DM1 treatment, in ongoing metastatic breast cancer studies have been reported since data cutoff date of 30 September 2013: hepatic encephalopathy in Study DM4997g/BO25734, neutropenic sepsis in Study TDM4370g/BO21977, subarachnoid hemorrhage in Study TDM4997g/BO25734, pneumonitis in Study TDM4788g/BO22589, and deaths of unknown cause in Study TDM4652g, MO28231, and TDM4884g/ML01356.

AEs leading to death which occurred either after the data cut off for analyses, or in studies not included in either pooled population, were not captured in the pooled analyses (n=884). The following additional AEs leading to death, assessed by the investigator to be related to trastuzumab emtansine treatment, have been reported into the safety database in studies in MBC, up to a clinical cut-off date of 30 September 2014 (each occurred once except where noted): pneumonitis (n = 3), hepatic encephalopathy (n = 2), neutropenic sepsis, subarachnoid hemorrhage, death of unknown cause, pneumonia, hepatotoxicity, multi-organ failure, acute renal failure, respiratory failure (n = 2), acute myeloid leukemia, interstitial pneumonitis, bilateral pneumonitis, sepsis, and upper gastrointestinal bleeding.

CONFIDENTIAL

In addition to the two fatal events of subarachnoid hemorrhage described [10] (one in MBC in Study TDM4997g/BO25734, as noted above and one in MGC in Study BO27952, there have been seven further fatal hemorrhagic events reported in patients with MBC receiving trastuzumab emtansine in clinical trials (Studies TDM4997g/BO25734, TDM4788g/BO22589, MO28231, TDM4652g/GO01355, and TDM4884g/ML01356). None of the additional seven cases in the MBC trials were considered by the investigator to have a relationship to trastuzumab emtansine treatment, all had significant contributing medical conditions, such as underlying malignancy (eg, brain metastasis) and in some cases the patients were also receiving anticoagulation therapy. However a contributory role for trastuzumab emtansine cannot be excluded, as a decrease in platelet count was reported at the time of the event in five cases (in 2 cases, platelet counts were not reported). The majority (78%) of the fatalities were due to hemorrhagic events in the CNS.
In Early Breast Cancer, one case of intracranial hemorrhage occurred in a patient in Study BO27938 which resulted in death. In study BO27952, a phase 2/3 study evaluating T-DM1 versus taxane in locally advanced or metastatic gastric cancer, the following AEs leading to death, assessed by the investigator to be related to trastuzumab emtansine treatment, have been reported into the safety database in studies in MGC, up to the clinical cut-off date of 30 September 2014 (each occurred once except where noted): cardiac failure, aspiration pneumonia, subarachnoid hemorrhage, alveolar hemorrhage, gastric hemorrhage (n=2). ,

1.3.1.2 Safety in Ongoing Studies

Study TDM4370g/BO21977(EMILIA) is a randomized phase 3 study comparing T-DM1 monotherapy with lapatinib plus capecitabine in participants with HER2+ unresectable LABC or MBC previously treated with trastuzumab and a taxane. Preliminary safety analyses were performed based on a data cut-off date of Januaray 31, 2014.

The incidence of grade ≥3 AEs was lower in participants treated with T-DM1 than in those receiving lapatinib plus capecitabine (47.3% *vs*. 59.6%). The most common grade ≥3 AEs in the T-DM1 group were thrombocytopenia (30.4%) and elevated serum concentrations of aspartate aminotransferase , alanine aminotransferase, anemia, fatigue, hypokalemia, and neutropenia (each occurring in more than 2% of patients). First occurrence of grade ≥3 thrombocytopenia was reported in the first two cycles of T-DM1 treatment. With dose modifications, majority of the participants who experienced thrombocytopenia or elevated serum aminotransferase levels were able to continue treatment. A total of 45 participants discontinued T-DM1 (9.2%), compared with patients who discontinued lapatinib (8.6%) or capecitabine (10.7%).

Three deaths in the T-DM1 group, metabolic encephalopathy after CNS progression and and neutropenic sepsis events , were attributed to T-DM1.

Study TDM4997g/BO25734 (TH3RESA) is evaluating trastuzumab emtansine compared with TPC in patients with HER2-positive MBC, who had received at least two HER2-directed regimens in the metastatic setting. There were 404 patients who received trastuzumab emtansine as their planned treatment, and 198 patients who received TPC. Fewer patients receiving trastuzumab emtansine than those receiving TPC had Grade ≥ 3 AEs (39.0% vs. 46.2%) or AEs leading to dose reduction (12.4% vs. 20.7%). The incidence of SAEs (24.1% vs. 22.3%) and of AEs leading to treatment discontinuation of any component of therapy (11.7% vs. 10.9%) similar between treatment arms. The most frequently reported AEs in the trastuzumab emtansine arm (in at least 20% of patients) were: nausea (35.5%), fatigue (30.3%), headache (24.3%) and constipation (22.3%).

CONFIDENTIAL

1.3.2 Animal Studies

Nonclinical toxicity studies have been conducted in rats and monkeys, including single-dose toxicity studies in both species, repeat-dose toxicity studies in rats and in monkeys up to eight iv injections given q3w, a cardiovascular safety pharmacology study in monkeys, and genotoxicity studies in monkeys [10].

A single-dose GLP study with a three-week recovery period was conducted in rats at doses up to 60 mg/kg. The highest dose was not tolerated. Animals that received 6 or 20 mg/kg T-DM1 showed no clinical signs of toxicity. Hepatocellular degeneration and liver necrosis (only at 20 mg/kg), renal tubular degeneration and necrosis, increased mitoses in liver, kidney, pituitary, and spleen, and a transient elevation of liver enzymes and mild thrombocytopenia were observed in animals treated with 6 or 20 mg/kg doses; all with complete resolution three weeks after dosing. As a result, 20 mg/kg was determined to be the highest non-severely toxic dose (HNSTD) following a single iv injection to male and female rats.

In cynomolgus monkeys, the HNSTD for single-dose administration of T-DM1 was determined to be 30 mg/kg based on two single-dose studies with a three-week recovery period after iv administration of T-DM1 and T-DM1 containing 5%–7% unconjugated DM1, respectively. All clinical pathology findings were reversible during a three-week recovery period, including elevation of serum AST (30 mg/kg), thrombocytopenia (30 mg/kg and 10 mg/kg), and alkaline phosphatase (30 mg/kg), mild increases in leukocyte counts (10 and 30 mg/kg), test-article-related microscopic findings in liver, spleen, kidney, skin, choroid plexus, and minimal axonal degeneration of the sciatic nerve (only in 30 mg/kg).

The toxicity of free DM1 was evaluated in single-dose GLP, and non-GLP studies in rats. A dose range-finding study identified a single iv dose of 0.2 mg/kg free DM1 as the MTD. A 0.2 mg/kg dose of free DM1 corresponds to a T-DM1 dose of 11.7 mg/kg.

Two repeat-dose GLP toxicity studies were conducted in cynomolgus monkeys with iv administration of T-DM1 at four dose levels (0, 1, 3, 10, and 30 mg/kg) every three weeks for four to eight doses followed by a three- or six-week recovery period. The 10 and 30 mg/kg doses were associated with reversible hepatotoxicity, and a mild decrease in platelet counts was also observed. The 10 and 30 mg/kg dose levels were associated with histological evidence of axonal degeneration in the sciatic nerve and in the dorsal funiculus of the spinal cord; these changes were seen at all necropsy time points and were more pronounced in animals at 30 mg/kg. Based on overall tolerability and reversibility of findings, the HNSTD of T-DM1 in cynomolgus monkeys following four injections given q3w was determined to be 10 mg/kg. Based on body surface area extrapolations of the HNSTD from repeat-dose studies in cynomolgus monkeys and a safety factor of 12, the proposed human dose was 0.3 mg/kg (10 mg/m^2).

A GLP cardiovascular safety pharmacology study was conducted in conscious cynomolgus monkeys using radiotelemetry. Animals were given single iv doses of 0, 3, 10, or 30 mg T-DM1/kg of body weight and followed for a three-week observation period. No cardiovascular abnormalities, including changes in troponins I or T or creatine kinase levels, and no effects on respiratory rate and respiratory depth, were detected.

A GLP human Ether-à-go-go Related Gene (hERG) assay was conducted to examine the in vitro effects of DM1 on the hERG channel current (a surrogate for IKr, the rapidly activating, delayed rectifier cardiac potassium current). The half maximal inhibitory concentration (IC_{50}) for the inhibitory effect of DM1 on hERG potassium current was determined to be > 29.5 μM. Based on an IC_{50} of ≥ 29.5 μM, average plasma DM1 concentrations detected in trastuzumab emtansine –treated patients (6 ng/mL), and 93%

CONFIDENTIAL

plasma protein binding (Study 05-1047), a safety margin of at least 30-fold can be calculated for DM1 effects on the hERG channel current.

Trastuzumab emtansine was compatible with cynomolgus monkey and human serum and plasma and did not cause hemolysis of cynomolgus monkey or human blood.

As thrombocytopenia was identified in clinical studies as a frequent AE of trastuzumab emtansine, in vitro and ex vivo studies were performed to assess the potential mechanism. In the in vitro studies of human megakaryocytes (MKs) derived fromhematopoietic stem cells (HSCs), treatment with trastuzumab emtansine inhibited MK cell maturation and decreased MK numbers. Additional studies suggested that the decrease in MK cell numbers occurred via internalization of trastuzumab emtansine in a target-independent, partially Fc-dependent manner with intracellular release of DM1 resulting in a reduction of the stem cell population. These observations support the hypothesis that impaired platelet production from MKs in the bone marrow may contribute to the thrombocytopenia observed in clinical trials. Ex vivo studies of human platelet function showed that at clinically relevant concentrations, neither trastuzumab emtansine nor DM1 had any observable effect on platelet aggregation or a direct impact on platelet activation. Neither trastuzumab emtansine nor DM1 inhibited platelet aggregation or activation induced by platelet agonists.

In vitro experiments were conducted to assess the potential cytotoxic effect, uptake, and catabolism of trastuzumab emtansine in primary human hepatocytes. Exposure to trastuzumab emtansine at clinically relevant concentrations (25–400 mg/mL) resulted in < 30% reduction in cellular ATP levels and a 40%-80% decrease in albumin secretion, with no observed changes in reactive oxygen species generation, glutathione levels, mitochondrial stress or alpha-1-antitrypsin levels. Similar changes were also seen in hepatocytes incubated with the same concentrations of anti-gD-DM1 (anti-glycoprotein D; a target irrelevant control) or DM1, but not with trastuzumab alone, suggesting the effects of trastuzumab emtansine on human hepatocytes are non-specific, HER2 independent, and related to the DM1 moiety. Following exposure to [125I]- or [111In]-labeled trastuzumab emtansine tracer alone, a small fraction (< 4%) of the added radioactivity was detected in the hepatocytes. Co-incubation with an excess of unlabeled trastuzumab emtansine further reduced the radioactivity (< 1%) in the cells. Exposure to [^{125}I] or [^{111}In] anti-gD-DM1 resulted in similarly lower levels (< 2%) of radioactivity in the hepatocytes, suggesting that trastuzumab emtansine uptake by hepatocytes is non-specific (non-target-mediated). Incubation with trastuzumab-[3H]
DM1 also revealed a low level of uptake (< 3% of the added radioactivity). Trastuzumab emtansine appeared to be catabolized in the hepatocytes: precipitation of cell lysate with acetonitrile (ACN) resulted in an increase in ACN-soluble radioactivity over time. RP-HPLC fractionation combined with LSC detected several species in the ACN-soluble fraction. Direct measurement of known trastuzumab emtansine catabolites using LC-MS/MS showed similar levels of catabolites, mainly as MCC-DM1 and a negligible amount of DM1 with no detectable Lys-MCC-DM1, in hepatocytes exposed to either trastuzumab emtansine or anti-gD-DM1, further supporting the non-specific uptake and catabolism of trastuzumab emtansine in human hepatocytes.

Genotoxicity of T-DM1 and DM1 was evaluated in a GLP cynomolgus or rat bone marrow micronucleus assays. Bone marrow was collected from monkeys that were given 1, 3, or 10 mg/kg iv T-DM1 q3w for eight doses. T-DM1 did not induce micronuclei in the bone marrow of cynomolgus monkeys under the conditions of the assay.

DM1 was evaluated at single iv doses of 0.01, 0.05, 0.1, or 0.2 mg/kg DM1 administered to male Sprague-Dawley rats. DM1 induced a dose-dependent increase in micronucleus frequency at 0.05, 0.1, and 0.2 mg/kg, demonstrating evidence of aneugenicity and/or clastogenicity. DM1 and/or its metabolites

CONFIDENTIAL

in the presence or absence of an exogenous mammalian metabolic activation system (S9) were negative in the *Salmonella-Escherichia coli*/Mammalian-Microsome Reverse Mutation Assay.

1.4 Pharmacokinetics

1.4.1 Human Studies

Phase 1 and 2 studies (qw and q3w regimens)

In the phase 1 trial in breast cancer participants (TDM3569g), T-DM1 was administered once every three weeks (q3w) or weekly (qw) as a 30- to 90-minute iv infusion [32, 33]. PK was evaluated on days 2, 3, 4/5, 8, 11, 15, and 18/19.Six dose levels (0.3 mg/kg, three participants; 0.6 mg/kg, one participant; 1.2 mg/kg, one participant; 2.4 mg/kg, one participant; 3.6 mg/kg, 15 participants; 4.8 mg/kg, three participants) were evaluated for the q3w schedule, and five dose levels (1.2 mg/kg, three participants; 1.6 mg/kg, three participants; 2.0 mg/kg, three participants; 2.4 mg/kg, seven participants; 2.9 mg/kg, three participants) were evaluated for the qw schedule [10]. Across all dose levels under both q3w and qw regimens, the T-DM1 has a faster CL and longer $t_{1/2}$ than total trastuzumab, consistent with preclinical studies; the mean values of post-infusion serum C_{max} and AUC_{inf} were lower for T-DM1 compared with total trastuzumab. Exposure to free DM1 relative to conjugated T-DM1 was very low, with maximum free DM1 levels of <7 ng/mL; on the basis of the ratios of AUC_{inf} and C_{max} values for T-DM1 to DM1, the DM1 exposure was approximately 10,000-fold less by mass and 50-fold less by molar equivalents compared with exposure to T-DM1. Little to no accumulation of DM1 was detected in later cycles using both q3w and qw regimens. Mean C_{max} and AUC_{inf} generally increased proportionally with dose, with C_{max} ranged from 9.76 to 130.3 µg/mL across the dose levels tested (0.3–4.8 mg/kg) for the q3w regimen and 22.6–78.1 µg/mL (dose range 1.2–2.9 mg/kg) for the qw regimen; the AUC_{inf} was 14.5 to 673.0 day•µg/mL across the dose levels tested (0.3–4.8 mg/kg) for the q3w regimen, and AUC_{inf} was 76.2 to 212.0 day•µg/mL for the qw regimen. The volume of distribution of T-DM1 approximates physiological serum volume, with mean values ranging from 30.7 to 58.4 mL/kg for the q3w regimen and 47. 5 to 59.8 mL/kg for the qw regimen; those values do not appear to change with dose [10].

For the q3w regimen, dose-dependent CL and $t_{1/2}$ values were observed for T-DM1. CL values tended to decrease and $t_{1/2}$ values tended to increase with dose across the 0.3–4.8 mg/kg dose range. For the qw regimen, mean CL and $t_{1/2}$ values were similar across the doses ranging from 1.2–2.9 mg/kg [10].

The MTD of the q3w regimen (3.6 mg/kg) was defined based on the occurrence of Grade 4 thrombocytopenia. Serum concentrations of T-DM1 after the first 3.6 mg/kg dose reached mean C_{max} of 76.2±19.1 µg/mL at the end of infusion, with a mean elimination $t_{1/2}$ of 3.1±0.7 days, mean AUC_{inf} value of 300.3±65.8 day•µg/mL, mean systemic CL value of 12.7±3.6 mL/day/kg, and V_{ss} value of 58.4±12.4 mL/kg. The MTD of the qw regimen was determined to be 2.4 mg/kg. Serum concentrations of T-DM1 reached mean C_{max} of 54.8±12.6 µg/mL at the end of the infusion, with a mean $t_{1/2}$ of 3.3±1.1 days, mean AUC_{inf} of 198.5±54.5 day•µg/mL, mean systemic CL of 13.1±4.1 mL/day/kg, and mean V_{ss} of 55.4±13.0 mL/kg [10].

The pharmacokinetics of serum T-DM1, serum total trastuzumab, and plasma DM1 following 3.6 mg/kg q3w dosing in phase 2 studies (TDM4258g, TDM4374g and TDM4688g) in MBC were consistent with those exhibited in the 3.6 mg/kg q3w treatment cohort from the phase 1 study (TDM3569g) [10, 34].

Population Pharmacokinetics

A comprehensive population PK (popPK) analysis was conducted by using the available trastuzumab emtansine concentration data obtained across five Phase I, Phase II and Phase III studies in MBC (Studies

CONFIDENTIAL

TDM3569g, TDM4258g, TDM4374g, TDM4450g/BO21976 and TDM4370g/BO21977) to describe the PK of trastuzumab emtansine, to estimate typical PK parameter values and associated inter-individual variability and to determine the effects of demographic and pathophysiological covariates associated with PK variability. A total of 9934 concentration-time records from 671 patients were used for the model development. Data from Study TDM4688g were used for validation of the model.

A two-compartment linear model with first-order elimination from the central compartment best described the serum trastuzumab emtansine concentration–time data across the clinical dose range studied (0.3 to 4.8 mg/kg). Trastuzumab emtansine exhibited linear PK across this dose range following IV administration (although there was some evidence of faster clearance in a small number of patients who received doses ≤ 1.2 mg/kg; however, model parameter estimates did not differ with the inclusion or exclusion of these patients). The population parameter values for clearance and volume of distribution of the central compartment (V_c) for a typical person were estimated to be 0.68 L/day and 3.13 L, respectively. The popPK analysis showed a mean $t_{ő}$ of 3.94 days for trastuzumab emtansine. Steady state was reached within Cycle 1; no accumulation of trastuzumab emtansine in plasma was observed after repeated dosing.

Using baseline characteristics across studies, a number of potentially clinically relevant covariates were examined, including but not limited to, age, weight, race, region, serum creatinine, creatinine clearance, serum albumin, total protein, total bilirubin, serum AST and ALT, INR, alkaline phosphatase, baseline concentrations of HER2 extracellular domain (ECD) and trastuzumab, and tumor burden-related covariates (metastatic status, performance status, centrally-confirmed HER2 status). Body weight, albumin, sum of the longest dimension of target lesions, AST, baseline serum ECD concentration, and baseline trastuzumab concentration were identified as statistically significant covariates for trastuzumab emtansine PK. With the exception of body weight, the magnitude of effect of these covariates on trastuzumab emtansine exposure was relatively small and did not have any clinically meaningful effect on trastuzumab emtansine exposure. Therefore, the body weight-based dose of 3.6 mg/kg q3w is considered appropriate.

Interactions with Other Drugs

The use of trastuzumab emtansine in combination with pertuzumab has been studied in patients with HER2-positive LABC or MBC in Study TDM4373g/BO22495. The exposures of trastuzumab emtansine and DM1, as estimated by noncompartmental analyses, were comparable with that reported by historical single-agent studies in patients with HER2-positive MBC. Trastuzumab emtansine clearance and volume of distribution in the central compartment, as estimated by popPK analysis, were also within the ranges of historical single-agent data. Pertuzumab trough and maximal exposures for Cycle 1 and at steady state were similar with those observed in a representative historical single-agent study with the same dosing regimen. Thus the risk of therapeutic protein-drug interaction when trastuzumab emtansine is given together with pertuzumab appears to be low.

The use of T-DM1 in combination with taxanes (paclitaxel or docetaxel) is being investigated in two studies (TDM4652g and BP22572) in participants with HER2+ LABC or MBC. Results showed that the PK of paclitaxel and docetaxel were similar with and without coadministration of trastuzumab emtansine and were also similar to historical single-agent data. Trastuzumab emtansine PK parameter values in the combination regimen studies were within the ranges of historical single-agent data. The Cmax of DM1 was < 7 ng/mL in both studies, and the average Cmax values were similar to historical results following trastuzumab emtansine given as a single agent[10].

1.4.2 Animal Studies

CONFIDENTIAL

PK, PK/pharmacodynamic, and toxicokinetic studies were performed in mice, rats, and cynomolgus monkeys.

The antibody component of T-DM1 does not cross-react with the rodent ortholog of HER2. As a result, PK studies conducted in non-tumor-bearing mice and rats assessed only the antigen-independent behavior of T-DM1. Pharmacodynamic studies were performed in mice bearing human HER2–expressing tumors. T-DM1 recognizes HER2 expressed by cynomolgus monkeys; therefore, this was the non-rodent species used for PK and toxicology studies.

PK of T-DM1 were bi-exponential in all nonclinical species tested, with a terminal half-life of ~0.9–6 days, a mean serum CL ranging from ~10 to 40 mL/kg/day, and central compartment volume similar to plasma volume. T-DM1 exhibited dose-proportional PK in mice and rats. In cynomolgus monkeys, a dose-dependent decrease in CL was observed; terminal $t_{1/2}$ increased with increasing dose at the doses tested (0.3, 3.0, or 30 mg/kg, iv administration). No remarkable changes were noted with repeated dosing (q3w for up to six months). No gender differences were observed in any species studied. The presence of HER2-bearing tumors had no effect on the PK of T-DM1 in the mouse model [10].

Biodistribution studies (in rats) with [^{125}I]-trastuzumab, [^{125}I]-T-DM1, and [^{3}H]-DM1 showed that conjugation of DM1 to trastuzumab limits the distribution of DM1 to highly perfused organs. In contrast, [^{3}H]-DM1 administration results in rapid and extensive distribution to many tissues. There was no persistent accumulation in tissues [10].

Biliary excretion is the predominant route of elimination for DM1-containing catabolites or DM1 metabolites following T-DM1 and DM1 dosing in rats, with urinary elimination as a minor route. Following trastuzumab-[^{3}H]DM1 dosing, up to 80% of radioactivity was recovered in rat bile and less than 5% in rat urine, mainly as DM1 and DM1-containing catabolites such as MCC-DM1, Lys-MCC-DM1, and DM1 adducts. Following [^{3}H]DM1 dosing, nearly 100% of radioactivity was recovered in bile [10].

As with trastuzumab, T-DM1 primarily undergoes receptor mediated and non-specific endocytosis, followed by trafficking to the lysosomal compartment where proteolytic degradation/catabolism occurs. DM1, in contrast, undergoes metabolism by cytochrome P (CYP)450 isoenzymes, specifically CYP450 3A4, and to a lesser extent CYP450 3A5, but is not an inhibitor or inducer of CYP450 enzymes at concentrations up to 600 ng/mL. DM1 is a substrate but not an inhibitor of PgP when tested at 0.5 μM in MDCKII MDR1 cells. Low levels of catabolites, including lys-MCC DM1, MCC-DM1 and DM1, have been observed across species, including rats and humans. MCC-DM1 levels were higher than other catabolites (DM1 and lys-MCC DM1) but did not exceed 122 ng/mL in participants in investigational trials [10].

The anti-therapeutic antibody response to T-DM1 in cynomolgus monkeys was low (approximately 11%) and no obvious effect on T-DM1 toxicokinetics was observed [10].

2. INVESTIGATIONAL STUDY AGENT ADMINISTRATION IN THE I-SPY 2 TRIAL

Intervention will be administered on an outpatient basis. Reported clinical AEs and potential risks are described in §3.2.

2.1 Dose Regimens and Dose Groups

The dose schedule for T-DM1 and pertuzumab is:

CONFIDENTIAL

Table 2. T-DM1 (q3w × 12weeks) with Pertuzumab[a] (q3w × 12weeks); Followed by AC (q2w or q3w)

Agent	Dose	Route	Cycle[b]
**T-DM1[a]**	**3.6 mg/kg**	**IV**	**1-4**<br>(weeks 1,4,7,10)
**Pertuzumab [a]**	**840 mg (loading dose)**<br>**420 mg (thereafter)**	**IV**	1<br>2-4<br>**(weeks 4, 7, 10)**
Doxorubicin	60 mg/m^2	IV	5-8
Cyclophosphamide	600 mg/m^2	IV	5-8

[a] T-DM1 is administered one hour after completion of pertuzumab

[b]Note that each cycle for T-DM1 and pertuzumab combinations = three weeks, each cycle for AC = two or three weeks.

2.2 Additional Eligibility Criteria

There are no additional eligibility criteria for T-DM1 and pertuzumab.

NOTE: Participants must meet all other investigational agent-specific criteria as described in Appendix C §2 and the main protocol §4.1.2 in order to be eligible for the treatment phase of I-SPY 2.

2.3 Contraindications

T-DM1 is contraindicated in participants with known hypersensitivity to any component of the product. No contraindications known for Pertuzumab.

2.4 Concomitant Medications

There are no agents known to interact adversely with concomitantly administered pertuzumab and/or T-DM1. No formal drug-drug interaction studies have been conducted with trastuzumab emtansine. Strong inhibitors of CYP3A4 should be avoided, see Table 3 for complete list. In addition, participants should avoid grapefruit juice and herbal remedies, including St. John's Wort. such as erythromycin and ketoconazole. Alcohol consumption should be held to moderation during T-DM1 treatment.

Table 3. Agents to Avoid while taking T-DM1 and pertuzumab

Generic Agent Name	Agent Brand Name ®
Clarithromycin	Biaxin
Digoxin	Cardoxi, Digitek, Lanoxin, Lanoxicaps
Erythromycin	Eyrc, E-Mycin
Fluconazole	Diflucan
Fluvoxamine	Luvox
Indinavir	Crixivan
Itraconazole	Sporanox
Ketoconazole	Nizoral, Kuric, Xolegel, Extina
Mibefradil	Posicor

CONFIDENTIAL

Generic Agent Name	Agent Brand Name ®
Miconazole	Desenex, Miconazex, Monistat
Nefazodone	Serzone
Nelfinavir	Viracept
Norfluoxetine	----
Quinine	Qualaquin, QM-260
Ritonavir	Norvir
Saquinavir	Invirase, Fortovase
Sertraline	Zoloft, Lustral
Troleandomycin	TAO
Voriconazole	Vfend
Zafirlukast	Accolate

2.5 Clinical Evaluation and Procedures

Laboratory evaluations for general safety monitoring during treatment are described in the main I-SPY2 protocol §8.1–8.3 with the following modification:

- Laboratory blood tests q3w, in conjunction with T-DM1 and pertuzumab
- Breast MRI, blood draw, and core biopsy at the end of week 3, prior to second cycle of T-DM1 and pertuzumab

Additional evaluation for this agent:

- Cardiac safety (LVEF) will be monitored by ECHO or MUGA at the following time points (the same method should be used at every time point in each participant):
  - After completion of T-DM1 plus pertuzumab and before initiating AC
  - After completing the AC regimen and before surgery

  If the results are abnormal, ECHO or MUGA will be repeated one month later

2.6 Dose Modification and Management of Toxicity

T-DM1 and pertuzumab will be administered every third week for 12 weeks for a total of four doses, unless specified otherwise in Table 5 below. Missed doses of T-DM1and/or pertuzumab should be made up to complete 4 cycles (12 weeks of treatment) unless otherwise specified in Table 5 below.

Toxicity will be graded for severity using the NCI CTCAE version 4.0. Initiation of the next cycle of therapy may be delayed no more than three weeks to allow recovery from toxicity. Treatment delay of 3 weeks (21 days) or greater due to toxicity will lead to stopping all protocol therapy. Participant will remain on study for outcome assessment. Participant should proceed with additional chemotherapy or surgery at the discretion of the treating physician. If removal is due to cardiac toxicity, the participant should be followed with echocardiograms every three–four months for one year after discontinuation of therapy.

Dose adjustments are to be made according to the organ system showing the greatest degree of toxicity.

All dose reductions will be based on blood counts obtained on a planned day of chemotherapy. Nadir counts will not be measured routinely.

CONFIDENTIAL

The following dose levels will be utilized for the purpose of dose modifications for toxicity:

Table 4. Dose Adjustments for T-DM1

Dose Adjustment	T-DM1 Dose, mg/kg
Standard dose	3.6
-1	3.0
-2	2.4*

*T-DM1 may be dose reduced to a dose no lower than 2.4 mg/kg.

There will be no dose reductions for pertuzumab.

Table 5.T-DM1+ Pertuzumab

Event	T-DM1+Pertuzumab
**Neutropenia**	
≥ 1000/mm^3	No change to T-DM1 and pertuzumab.
< 1000/mm^3 -500/mm^3	Hold T-DM1 and pertuzumab until ANC ≥ 1000/mm^3. Resume T-DM1 and pertuzumab based on number of episodes and timing of recovery:<br>First Episode:<br>• No Change to T-DM1 and pertuzumab doses. Use prophylactic pegfilgrastim or filgrastim for all subsequent cycles.<br>Second Episode:<br>• Doses reduce T-DM1 by one dose level for all subsequent cycles. No change to pertuzumab.<br>Third Episode *(only applicable if dose of T-DM1is at 3.0mg/kg; if T-DM1 dose is 2.4mg/kg stop T-DM1 and pertuzumab):*<br>• Dose reduce T-DM1 by one dose level for all subsequent cycles. No change to pertuzumab.<br>Fourth Episode:<br>• Stop T-DM1and pertuzumab. Participant should proceed with additional chemotherapy or surgery at the discretion of the treating physician. Participant remains on study for outcome assessment.<br><br>G-CSF may be used between days 2–6 according to participant need, at physician discretion, and to avoid dose reduction. Pegfilgrastim or filgrastim may be used
< 500/mm3	Hold T-DM1 and pertuzumab until ANC ≥ 1000/mm3. Resume T-DM1 and pertuzumab based on number of episodes:<br>First Episode:<br>• No Change to T-DM1 and pertuzumab doses. Use prophylactic pegfilgrastim or filgrastim for all subsequent cycles.<br>Second Episode:<br>• Dose reduce T-DM-1 by one dose level for all subsequent cycles. No change to pertuzumab.<br>Third Episode:<br>• Dose reduce T-DM-1 by one dose level for all subsequent cycles. No change to pertuzumab.<br>•<br>Fourth Episode:

CONFIDENTIAL

Event	T-DM1+Pertuzumab
	• <u>Stop T-DM1and pertuzumab</u>. Participant should proceed with additional chemotherapy or surgery at the discretion of the treating physician. Participant remains on study for outcome assessment.<br>.
**Neutropenic Fever**	
ANC ≤ 1000/mm^3, fever ≥ 38.5°C	Hold T-DM1 and pertuzumab until resolved (ANC > 1000/mm^3, fever < 38.5°C. Resume T-DM1 and pertuzumab according to number of episodes:<br>• First episode: no change in the dose of T-DM1and pertuzumab. Use prophylactic pegfilgrastim or filgrastim for all subsequent cycles<br>• Second episode: Dose reduce T-DM1 by one dose level. No change to pertuzumab.<br>• Third episode: Dose reduce T-DM1 by one dose level. No change to pertuzumab.<br>• Fourth Episode: <u>Stop T-DM1and pertuzumab.</u> Participant should proceed with additional chemotherapy or surgery at the discretion of the treating physician. Participant will remain on study for outcome assessment.<br>• If T-DM1 and pertuzumab are held for 3 weeks in a row, <u>Stop T-DM1 and pertuzumab.</u> Participant should proceed with additional chemotherapy or surgery at the discretion of the treating physician. Participant remains on study for outcome assessment.
**Thrombocytopenia**	
≥ 75,000/mm^3	No change to T-DM1and pertuzumab.
< 75,000/mm^3 -25,000/mm^3	Hold T-DM1 and pertuzumab and assess weekly until ≥ 75,000/mm^3. Resume T-DM1 and pertuzumab at previous dose.<br><br>If T-DM1 and pertuzumab are held for 3 weeks (21 days) in a row, <u>stop T-DM1 and pertuzumab</u>. Participant should proceed with additional chemotherapy or surgery at the discretion of the treating physician. Participant remains on study for outcome assessment
<25,000/mm^3	Hold T-DM1 and pertuzumab and assess weekly until ≥ 75,000/mm3. Resume T-DM1 and pertuzumab based on number of episodes:<br><u>First Episode:</u><br>• Dose reduce T-DM1 by one dose level for all subsequent cycles. No change to pertuzumab.<br><u>Second Episode:</u><br>• Dose reduce T-DM-1 by one dose level for all subsequent cycles. No change to pertuzumab.<br><u>Third Episode: Stop T-DM1and pertuzumab</u>. Participant should proceed with additional chemotherapy or surgery at the discretion of the treating physician. Participant remains on study for outcome assessment
**Anemia**	
All grades	For all anemia events regardless of grade, iron studies should be checked and iron should be replaced as indicated.

CONFIDENTIAL

**Event**	**T-DM1+Pertuzumab**
	• Red blood cell transfusions can be given at the investigators discretion as needed for symptom control.
**Hepatic**	
Grade 1	No change toT-DM1 and pertuzumab.
Grade 2	Grade 2 bilirubin<br>Hold T-DM1 and pertuzumab until bilirubin resolves to ≤ grade 1. Resume T-DM1 and pertuzumab at same dose level.<br>• If T-DM1 and pertuzumab are held for 3 weeks (21 days) in a row, stop T-DM1 and pertuzumab.. Participant should proceed with additional chemotherapy or surgery at the discretion of the treating physician. Participant remains on study for outcome assessment.<br>A rise in indirect bilirubin with a normal direct bilirubin believed to be attributable to Gilbert's disease does not require change in dose or a drug hold. A note to file should be created.<br>Grade 2 AST or ALT:<br>• Hold until resolves to ≤ grade 1. No change to T-DM1 and pertuzumab dose
>Grade 2 AST or ALT **AND** Bilirubin >2 X ULN or clinical jaundice	Stop T-DM1 and pertuzumab. Participant should proceed with additional chemotherapy or surgery at the discretion of the treating physician. Participant remains on study for outcome assessment.
Grade 3	Grade 3 bilirubin (not due to Gilbert's disease):<br>Hold T-DM1 and pertuzumab until bilirubin resolves to ≤ grade 1.<br>• Dose reduce T-DM1 by one dose level for all subsequent cycles. No change to pertuzumab.<br>If T-DM1 and pertuzumab are held for 3 weeks (21 days) in a row, stop T-DM1 and pertuzumab. Participant should proceed with additional chemotherapy or surgery at the discretion of the treating physician. Participant remains on study for outcome assessment<br>Grade 3 AST or ALT e:<br>Hold T-DM1 and pertuzumab until AST/ALT resolve to ≤ grade 1.<br>• Resume T-DM1with a one dose level reduction for all subsequent cycles. No change to pertuzumab.<br>• If T-DM1 and pertuzumab are held for 3 weeks in a row, stop T-DM1 and pertuzumab. Participant should proceed with additional chemotherapy or surgery at the discretion of the treating physician. Participant remains on study for outcome assessment.
Grade 4	Grade 4 AST or ALT or bilirubin:<br>Stop T-DM1and pertuzumab. Participant should proceed with additional chemotherapy or surgery at the discretion of the treating physician. Participant remains on study for outcome assessment.
Clinical signs of liver dysfunction	Stop T-DM1and pertuzumab. Participant should proceed with additional chemotherapy or surgery at the discretion of the treating physician. Participant remains on study for outcome assessment.

CONFIDENTIAL

Event	T-DM1+Pertuzumab
	Participants should be evaluated by a hepatologist. If there are signs of portal hypertension (*e.g.*, ascites and varices), the possibility of NRH should be considered.
**Hypokalemia**	
All grades	No change to T-DM1 and pertuzumab. Potassium should be repleted to normal levels per institutional standard.<br><br>If greater than one episode occurs, contact the DCC at (1-855-889-5170).
**Nausea/Vomiting/Anorexia**	
Grade 1–2	No change to T-DM1 and pertuzumab.<br>• Nausea and/or vomiting should be controlled with adequate anti-emetic therapy. Prophylactic anti-emetic therapy (*e.g.,* aprepitant, ondansetron, palonosetron, dexamethasone) should be administered to all participants; specific agents are at the discretion of the treating physician.<br>• Participants are encouraged to take plenty of oral fluids.<br><br>If symptoms persist despite maximal anti-emetic therapy:<br>• Hold T-DM1and pertuzumab until recovery to ≤ grade 1. Resume T-DM1 and pertuzumab at same dose level.
≥ Grade 3	Hold T-DM1 and pertuzumab until resolved to ≤grade 1.Resume T-DM1 and Pertuzumab at previous dose with modification of premedications to maximal anti-emetic supportive care.<br>• For second episode ≥grade 3 despite maximal supportive care: dose reduce T-DM1 for all subsequent cycles. No change to pertuzumab.
**Diarrhea**	
≥ Grade 3	Make all reasonable medical attempts to control symptoms. If unsuccessful, hold T-DM1 and pertuzumab until diarrhea resolved to ≤grade 1. Resume T-DM1 and pertuzumab based on time to recovery:<br>• ≤1 week—no change to T-DM1and pertuzumab<br>• >1 week and <3 weeks—at physician discretion:<br>o Dose reduce T-DM1 by one dose level for all subsequent cycles. No change to pertuzumab.<br>• ≥3 weeks—stop T-DM1 and pertuzumab. Participant should proceed with additional chemotherapy or surgery at the discretion of the treating physician. Participant remains on study for outcome assessment.
**Mucositis/Stomatitis**	
Grade 1–2	No change to T-DM1 and pertuzumab.
≥ Grade 3	Hold T-DM1 and pertuzumab until symptoms have resolved to ≤ grade 1.<br>• Resume T-DM1 and pertuzumab at the previous dose with modification of premedications.<br>• Consideration should be given for the addition of G-CSF.<br><br>For persistent toxicity ≥grade 3 despite maximal supportive care:<br>• Dose reduce T-DM1 by one dose level for all subsequent doses. No change to pertuzumab.
**Cardiac**	

CONFIDENTIAL

**Event**	**T-DM1+Pertuzumab**
Asymptomatic decline in LVEF that warrants intervention<br><br>LVEF < lower limit of normal AND absolute decrease in LVEF of ≥ 10% compared with pretreatment LVEF<br><br><u>OR</u><br><br>LVEF had an absolute decrease of ≥ 15% compared with pretreatment LVEF.	StopT-DM1 and pertuzumab for 4 weeks<br><br>Reassess LVEF at 4 weeks:<br>o If LVEF has recovered to baseline or the absolute decrease from baseline is <15 percentage points, resume T-DM1 and pertuzumab at previous dose.<br>If LVEF has not either recovered to baseline or the absolute decrease from baseline is ≥15 percentage points, <u>stop T-DM1 and pertuzumab therapy</u>. Participant should proceed with additional chemotherapy or surgery at the discretion of the treating physician. Participant remains on study for outcome assessment of the treating physician.
Symptomatic cardiac heart failure	<u>Stop T-DM1 and pertuzumab therapy</u>.<br>Refer for cardiologic evaluation and appropriate management for heart failure.<br><br>Participant should proceed with additional chemotherapy or surgery at the discretion of the treating physician. Participant remains on study for outcome assessment.
**Neurotoxicity**	
Grade 0–2	No change to T-DM1 and pertuzumab.
Grade 3	Hold T-DM1 and pertuzumab until neuropathy improves to ≤ grade 2. Resume T-DM1 and pertuzumab based on number of episodes:<br><u>First Episode:</u> No change to T-DM1 and pertuzumab.<br><u>Second Episode:</u> Dose reduce T-DM1 by one dose level for all subsequent cycles. No change to pertuzumab.<br><u>Third Episode:</u> Does reduce T-DM1 by one dose level for all subsequent cycles. No change to pertuzumab.<br><u>Fourth Episode:</u> Stop T-DM1 and pertuzumab. Participant should proceed with additional chemotherapy or surgery at the discretion of the treating physician. Participant remains on study for outcome assessment.<br><br>If T-DM1 and pertuzumab are held for 3 weeks in a row (21 days), <u>stop T-DM1 and pertuzumab.</u> Participant should proceed with additional chemotherapy or surgery at the discretion of the treating physician. Participant remains on study for outcome assessment.
Grade 4	Hold T-DM1 and pertuzumab until neuropathy improves to ≤ grade 2. Resume T-DM1 and pertuzumab based on number of episodes:<br><u>First Episode:</u> No change to T-DM1 and pertuzumab.<br><u>Second Episode: Stop T-DM1 and pertuzumab</u>. Participant should proceed with additional chemotherapy or surgery at the discretion of the treating physician. Participant remains on study for outcome assessment<br>If T-DM1 and pertuzumab are held for 3 weeks in a row (21 days), <u>stop T-DM1 and pertuzumab.</u> Participant should proceed with additional chemotherapy or surgery at the discretion of the treating physician. Participant remains on study for outcome assessment

CONFIDENTIAL

Event	T-DM1+Pertuzumab
**Anaphylaxis/Hypersensitivity**	
Mild (*e.g*., mild flushing, rash, pruritis)	Mild symptoms (grade 1: *e.g.*, transient flushing, rash or fever):<br>• Complete infusion. No change to T-DM1 and /or pertuzumab.<br>• No treatment required, but observe participant at least until symptoms have resolved.
Moderate (*e.g*., moderate flushing, rash, mild dyspnea, chest discomfort)	Moderate symptoms (grade 2: *e.g.*, rash, flushing, urticaria, dyspnea, chest discomfort):<br>For T-DM1:<br>• Hold T-DM1 infusion.<br>• Give intravenous diphenhydramine 20–25 mg and intravenous dexamethasone 10 mg.<br>• Resume T-DM1 infusion after recovery of symptoms at half the previous rate for 30 minutes. If no recurrence of symptoms, the planned rate may be resumed.<br>For pertuzumab:<br>• Stop pertuzumab infusion.<br>• Treat symptomatically according to institutional guidelines.<br>• Resume pertuzumab infusion at a slower rate according to institutional guidelines after reaction has resolved. If no recurrence of symptoms, the planned rate may be resumed.<br>If symptoms recur after T-DM1 or pertuzumab re-challenge:<br>• Stop T-DM1 or pertuzumab infusion and stop all subsequent T-DM1 and pertuzumab treatment. Participant should proceed with additional chemotherapy or surgery at the discretion of the treating physician. Participant remains on study for outcome assessment.<br><br>Any moderate hypersensitivity reaction should be discussed with the protocol chair if drug is to be continued. Contact the DCC for further instructions (1-855-889-5170).
Severe (*e.g*., hypotension requiring pressers, angioedema, respiratory distress requiring bronchodilators)	Severe or life-threatening symptoms (grade 3 or 4: *e.g.,* hypotension, angioedema, respiratory distress or anaphylaxis):<br>• Stop T-DM1 or pertuzumab infusion.<br>• Administer diphenhydramine 25 mg and dexamethasone 10 mg iv. Add epinephrine or bronchodilators as needed per institutional guidelines.<br>• Stop T-DM1 and pertuzumab. Participant should proceed with additional chemotherapy or surgery at the discretion of the treating physician. Participant remains on study for outcome assessment.
**Other Clinically Significant Toxicity Excluding Fatigue, Alopecia, and Leukopenia at Physician Discretion**	
Grade 0 or 1	No change.
≥ Grade 2	Hold T-DM1 and pertuzumab.<br>• Contact the DCC for further instruction (1-855-889-5170).

Abbreviations: ANC: absolute neutrophil count; iv: intravenous injection
Grades refer to CTCAE version 4

3. INVESTIGATIONAL AGENT PHARMACEUTICAL INFORMATION

3.1 Investigational Agent (Master IND # 105,139, IND Sponsor: QLHC)

CONFIDENTIAL

Confidential pharmaceutical information for investigational study agents supplied by pharmaceutical partner is available through FDA IND cross reference letter trastuzumab emtansine.

3.2 Reported Clinical AEs and Potential Risks

3.2.1 Identified Risks for Pertuzumab

More than 6886 participants have been exposed to pertuzumab as monotherapy or in combination with various chemotherapeutic drugs (n=1549) as of the clinical cut-off date of December 7, 2014. Safety data is available from 27 separate studies, representing the majority of these participants completed or proposed for enrollment at this time. The most common and frequent AEs (>20% patients) reported for participants receiving pertuzumab monotherapy are diarrhea, fatigue, nausea, vomiting, and decreased appetite, with the majority being grade 1 or 2. Pertuzumab was well tolerated when given in combination with trastuzumab with an increase in the incidence but not the severity of the AEs seen with pertuzumab alone (notably diarrhea, rash and fatigue). When compared to the AE profile seen with the combination of trastuzumab and docetaxel, the addition of pertuzumab added little additional toxicity (predominantly diarrhea, mucosal inflammation, rash and febrile neutropenia) when all three drugs were used concurrently.

The most frequently occurring AEs during neoadjuvant treatment with pertuzumab, trastuzumab and docetaxel were alopecia, neutropenia, diarrhea, nausea, fatigue, rash, and mucosal inflammation. The tolerability of this regimen was broadly comparable to pertuzumab plus docetaxel alone. Where pertuzumab and trastuzumab were given in addition to commonly used anthracycline-based and carboplatin-based neoadjuvant regimens, the most common AEs were diarrhea, alopecia, nausea, neutropenia, vomiting, fatigue, anemia, dyspepsia, and thrombocytopenia

Serious or severe infusion-related symptoms have rarely been observed in participants receiving pertuzumab. A low level of cardiac toxicities, predominantly associated with asymptomatic declines in LVEF, have been reported. Importantly, despite targeting the same HER2 pathway, pertuzumab adds no significant cardiac toxicity when given with trastuzumab (with or without chemotherapy.

<u>Infusion-related Symptoms</u>

In participants receiving single-agent pertuzumab, 2% of participants experienced AEs during pertuzumab infusions including symptoms such as fever, chills, diarrhea, fatigue, hypotension, shortness of breath, skin rash, headache, nausea, vomiting, and/ or hypersensitivity reactions, including anaphylaxis. If a significant IAR occurs, the infusion should be slowed down or interrupted and appropriate medical therapies should be administered.

<u>Left Ventricular Dysfunction</u>

In completed studies, the incidence of left ventricular dysfunction associated with pertuzumab does not appear to be greater than that associated with trastuzumab. In participants selected for having good cardiac function at baseline and with minimal cardiac risks, the combination of trastuzumab and pertuzumab is well tolerated, even when chemotherapy is also administered. Overall, 16 symptomatic cardiac failure events (as reported in individual studies as symptomatic CHF or LVSD) have been reported in 1326 participants with advanced malignant disease or early stage breast cancer treated with pertuzumab (data from completed primary analyses). Six of these events were reported in participants with MBC, two in participants with ovarian cancer, and five in participants with EBC.

CONFIDENTIAL

Five of the participants (all with MBC) had received prior anthracyclines. Of the participants with breast cancer, all but one received pertuzumab in combination with trastuzumab. In the pivotal phase 3 study WO20698/TOC4129g a total of 24 participants (6.1%) receiving pertuzumab plus trastuzumab and docetaxel experienced a decline in LVEF, compared to 28 participants (7.4%) in the placebo-controlled arm. Participants with significant cardiac disease or a baseline LVEF below the institution's lower limit of normal should not commence treatment with pertuzumab. Monitoring of LVEF is advised while participants are receiving pertuzumab.

Diarrhea and Rash

Diarrhea was observed in approximately 57% of participants treated with single-agent pertuzumab in phase 2 studies, and between 38%–100% of participants in phase 2 and phase 3 combination therapy studies. Diarrhea was Grade 1 or 2 in the vast majority of events.

Rash was observed in approximately 24% of participants receiving single-agent pertuzumab, with the majority (20%) deemed treatment-related. Rash was observedin 11%–73% of participants in pertuzumab combination studies. The rash was generally of Grade 1 or 2 severity.

Neutropenia

In the phase 3 study WO20698/TOC4129g incidence of Grade ≥ 3 febrile neutropenia was increased in participants with MBC receiving pertuzumab, trastuzumab and docetaxel compared to those in the placebo-controlled arm (13.7% *vs.* 7.6%, respectively). No febrile neutropenia AEs started after docetaxel was discontinued.

Respiratory Symptoms

There is a potential risk of ILD because of its role in inhibiting heterodimerization with other members of the HER family, including EGFR. However, few reports of ILD have been received from participants receiving pertuzumab and these indicated alternative causes for the events (*e.g.*, concomitant medication, preceding/concurrent neutropenia with potential infection) or relevant medical history.

Immunogenicity

In the phase 1/2 trials, two of the 386 pertuzumab-treated participants (0.5%) who had at least one post-dose sample available for anti-human antibody analysis, tested positive for antibodies to pertuzumab. Both participants experienced grade 3 hypersensitivity reactions that precluded further administration of pertuzumab. In the phase 3 WO20698/TOC4129g study, antibodies to pertuzumab were detected in 6.7% of participants in the placebo, trastuzumab and docetaxel arm and 3.3% of participants in the pertuzumab, trastuzumab and docetaxel arm. None of these participants experienced anaphylactic/hypersensitivity reactions that were clearly related to anti-drug antibody development.

3.2.2 Identified Risks for T-DM1

The safety of T-DM1 has been evaluated in more than 884 patients in clinical trials. [10] Thrombocytopenia, increases in serum AST and ALT, infusion/hypersensitivity reaction, pneumonitis, cardiac toxicity, and NRH have been identified as risks with T-DM1 use. NRH is a rare liver condition characterized by widespread benign transformation of hepatic parenchyma into small regenerative nodules, and diagnosis can only be confirmed by histopathology.

Thrombocytopenia

CONFIDENTIAL

Reversible thrombocytopenia has been commonly observed in completed and ongoing studies with T-DM1. Most events were grade 1–2; grade 3–4 thrombocytopenia has been observed less commonly. Declines in platelet counts were mostly transient; platelet count nadirs occurred at around Day 8 of each cycle and generally recovered to baseline by Day 1 of the subsequent q3w cycle. The incidence of grade 1–2 thrombocytopenia gradually increased over successive cycles; there was no increase in the proportion of grade ≥3 abnormalities. The number of participants who experienced platelet count recovery to normal levels by Day 1 of the subsequent cycle decreased with successive cycles, suggesting a modest cumulative effect of T-DM1 on platelet count. There was no clear association between thrombocytopenia and severe hemorrhagic events; however, the use of platelet transfusions has been reported. No apparent correlation between worst post-baseline platelet count grade and worst grade of hemoglobin and/or worst white blood count was detected.

Thrombocytopenia has also been linked to severe bleeding events, some with a fatal outcome, including central nervous system hemorrhage, that have been reported in clinical trials with trastuzumab emtansine. A total of 36.5% of patients treated with single-agent trastuzumab emtansine had a hemorrhagic event. The vast majority of these AEs were Grade 1-2 (principally epistaxis): a total of 2.0% of patients had a Grade ≥ 3 event). There have been nine fatal hemorrhagic events reported in patients receiving trastuzumab emtansine in clinical trials (Studies TDM4997g/BO25734, Study BO27952, TDM4788g/BO22589, MO28231, TDM4652g/ GO01355, and TDM4884g/ML01356). Two of these cases (subarachnoid hemorrhage in Study TDM4997g/BO25734 and another in Study BO27952) were considered by the investigator to have a relationship to trastuzumab emtansine treatment, while the remaining seven had significant contributing medical conditions, such as underlying malignancy (eg. brain metastasis) and in some cases the patients were also receiving anticoagulation therapy. However a contributory role for trastuzumab emtansine cannot be excluded as a decrease in platelet count was reported at the time of the event in five cases (in 2 cases, platelet counts were not reported). The majority (67%) of the fatalities were due to hemorrhagic events in the CNS [10].

Monitoring with a complete blood count (CBC) should occur prior to each T-DM1 administration, as per protocol. Participants who experience thrombocytopenia should have a CBC at least weekly until recovery. Dose reductions for severe thrombocytopenia are described in the protocol. The T-DM1 dose has not been re-escalated in clinical trials conducted to date. Participants who continue to experience severe thrombocytopenia despite dose reduction of T-DM1 to 2.4 mg/kg (q3w dosing) and whose platelet counts do not recover to baseline or grade ≤1 within the allowable delay of 21 days from the participant's last dose should be discontinued from study treatment.

Increased Serum Transaminases and Hepatotoxicity

Rare cases of severe hepatotoxicity, including death due to drug-induced liver injury and hepatic encephalopathy, have been observed in participants treated with T-DM1. While there is evidence of drug-induced liver toxicity in participants treated with trastuzumab emtansine, its potential to cause liver injury with clinically meaningful changes in liver function is unclear as the observed cases were confounded by concomitant medications with known hepatotoxic potential and/or underlying conditions. Nevertheless, a contributory role of trastuzumab emtansine in these cases cannot be excluded. Therefore, severe liver injury remains an important potential risk with T-DM1.

Transient increases in serum AST and ALT have been observed in all T-DM1 studies. Grade 1–2 events have been observed frequently; Grade 3–4 events have been observed less commonly. The incidence of increased AST was substantially higher than that for increased ALT. Increases in AST and ALT were commonly observed by Day 8 of each cycle and generally returned to baseline by Day 21. The incidence of AEs potentially related to abnormalities in hepatic synthetic function (hypoalbuminemia, coagulation disorders) was negligible.

CONFIDENTIAL

Liver function should be stable before first dose of T-DM1 and monitored as described in the protocol, at least prior to each infusion. Specific guidance on monitoring, dose reductions and discontinuation for severe increases in serum AST, ALT, and total bilirubin are described in each protocol. Trastuzumab emtansine treatment should be permanently discontinued in participants with serum transaminases >3× ULN and concomitant total bilirubin >2× ULN. If laboratory parameters do not recover to baseline or Grade ≤ 1 within 42 days of the last dose, the participant should be permanently discontinued from trastuzumab emtansine.

Cases of nodular regenerative hyperplasia (NRH) of the liver have been identified from liver biopsies in participants presenting with signs and symptoms of portal hypertension. NRH was also observed in one fatal case of hepatic failure. NRH is a rare liver condition characterized by widespread benign transformation of hepatic parenchyma into small regenerative nodules; NRH may lead to non-cirrhotic portal hypertension. Diagnosis of NRH can only be confirmed by histopathology. NRH should be considered in participants who develop clinical symptoms of portal hypertension and/or a cirrhosis-like pattern seen on computed tomography (CT) scan but with normal transaminases and no other manifestations of cirrhosis or liver failure. Upon diagnosis of NRH, trastuzumab emtansine treatment must be permanently discontinued.

Infusion-Related Reaction/Hypersensitivity Reaction

Infusion-related reactions are known to occur with the administration of monoclonal antibodies and have been reported with trastuzumab emtansine. Infusion reaction AEs (comprising MedDRA preferred terms infusion-related reaction, hypersensitivity, and urticarial) occurred rarely and were mostly grade 1–2. Non-specific symptoms that occurred on the first day of T-DM1 infusion which could be associated with an infusion reaction included fatigue, nausea, chills, pyrexia and, less commonly, headache, hypertension, vomiting, and cough. The evaluation of events that may constitute infusion reactions will be performed as more data become available.

In clinical trials of trastuzumab emtansine, premedication for the first infusion has not been required. Participants who experience T-DM1 infusion-related temperature greater than 38.5ºC, or other minor infusion-related symptoms, may be treated symptomatically. Serious infusion-related events manifested by dyspnea, hypotension, wheezing, bronchospasm, tachycardia, reduced oxygen saturation, or respiratory distress should be managed with supportive therapies as clinically indicated according to standard medical practice.

If an infusion reaction or a hypersensitivity reaction occurs during an infusion of trastuzumab emtansine, the infusion should be interrupted and the participant should be monitored until complete resolution of any observed symptoms. Similarly, if a pulmonary event occurs at any time during or after T-DM1 administration, the participant should be monitored and treated with appropriate medical care. Refer to the protocol for specific guidance on premedication and rates of administration for participants who experienced an infusion-related reaction with T-DM1.

Subsequent administration of T-DM1 in participants who have previously experienced a hypersensitivity reaction has not been studied. Participants who develop a severe infusion reaction, including anaphylaxis, angioedema, or acute respiratory distress syndrome during an infusion of T-DM1 should discontinue treatment.

Pneumonitis

Severe pulmonary events including interstitial lung disease, dyspnea, pneumonitis, pulmonary infiltrates, pleural effusions, non-cardiogenic pulmonary edema, pulmonary insufficiency, hypoxia, and acute

CONFIDENTIAL

respiratory distress syndrome have been reported with the use of trastuzumab, a component of T-DM1. These events may or may not occur as sequelae of infusion reactions and occasionally resulted in fatal outcome. Participants with symptomatic intrinsic lung disease or with extensive tumor involvement of the lungs may be at greater risk of severe reactions.

Pneumonitis has been rarely reported with T-DM1. Signs, symptoms, and clinical findings include dyspnea, cough, fatigue, and pulmonary infiltrates. There were no fatalities among the cases of pneumonitis reported with T-DM1; however, in some participants with multiple lung metastases, ventilatory support (mechanical ventilation) was required. Treatment included administration of steroids, oxygen, and study drug discontinuation.

Cases of interstitial lung disease (ILD) and acute respiratory distress syndrome have also been rarely reported in T-DM1 clinical trials. However, a causal relationship between these events and T-DM1 has not been established. Participants who have experienced a pulmonary event should be carefully evaluated before commencing T-DM1 treatment.

Cardiac Toxicity

Treatment with trastuzumab, a component of trastuzumab emtansine, has resulted in subclinical and clinical cardiac dysfunction or failure manifesting as CHF, decreased LVEF, and cardiac death. The incidence and severity of cardiac dysfunction was highest in participants who received trastuzumab concurrently with anthracycline-containing chemotherapy regimens. It is recommended that, only patients with LVEF ≥ 50% and without significant cardiac history should initiate trastuzumab emtansine (refer to the protocols for study-specific eligibility criteria). Decreased left ventricular ejection fraction (LVEF) (< 40% income cases) has been observed in participants treated with trastuzumab emtansine. Participants who develop symptomatic CHF should discontinue treatment with trastuzumab emtansine.

Neurotoxicity

Peripheral neuropathy, mainly Grade 1 and predominantly sensory, has been reported in clinical trials of trastuzumab emtansine. In patients experiencing Grade 3 or 4 peripheral neuropathy, treatment with trastuzumab emtansine should be temporarily discontinued until symptoms resolve or improve to ≤ Grade 2. Patients should be clinically monitored on an ongoing basis for signs/symptoms of neurotoxicity.

Extravasation/Injection Site Reactions

In trastuzumab emtansine clinical studies, reactions secondary to extravasation have been observed, and more frequently within 24 hours of the infusion. These reactions were usually mild and included erythema, tenderness, skin irritation, pain, or swelling at the infusion site. More severe events such as cellulitis, pain (tenderness and burning sensation), and skin irritation are rare, but have been received as part of the continuing surveillance of trastuzumab emtansine safety.

3.2.3 Potential Risks and Precautions

Potential risks associated with T-DM1 described here are based on observed nonclinical toxicities, and AEs observed in clinical trials of T-DM1, as well as clinical toxicities related to its components (trastuzumab and maytansine), and other DM1-containing ADCs. The following AEs have been described with T-DM1 and therefore constitute potential risks: cardiac dysfunction, peripheral neuropathy, hypokalemia, renal disorders, nodular regenerative hyperplasia and immunogenicity. Extravasation was observed in clinical trials with maytansine and cannot be excluded as a potential risk with T-DM1.

Overdose

CONFIDENTIAL

There is no known antidote for trastuzumab emtansine overdose. In case of overdose, the participant should be closely monitored. Cases of overdose have been reported with trastuzumab emtansine treatment, most associated with thrombocytopenia that resolved in a few days, but there was one death. In the fatal case, the participant incorrectly received trastuzumab emtansine 6 mg/kg (1.67 times the dose used in I-SPY2) and died approximately three weeks following the overdose; a cause of death and a causal relationship to trastuzumab emtansine were not established.

3.3 Investigational Agent Availability

Pertuzumab is manufactured by Genentech. The drug product is provided in 20-mL vial which contains 420 mg of pertuzumab (14.0 mL/vial). The sterile, preservative-free liquid concentrate is supplied at 30 mg/ml pertuzumab in 20 mM L-histidine acetate (pH 6.0), 120 mM sucrose and 0.02% polysorbate 20. T-DM1 is in development by Genentech under a collaboration agreement with ImmunoGen. It is provided as lyophilized product to be reconstituted in its single-use vial to produce liquid T-DM1 at a concentration of 20 mg/mL.

The investigator or designee will record the lot number, expiration date and the amount of study medications dispensed.

The study agents are provided to the QLHC under a CTA between Agent Manufacturer and the QLHC.

3.4 Investigational Agent Distribution

Agents will only be released by sponsor after documentation of IRB approval of the sponsor-approved protocol and consent is provided to sponsor and the collection of all Essential Documents is complete.

Sponsor-supplied agents may be requested by the investigator (or their authorized designees) at each organization. Sponsor guidelines require that the agent be shipped directly to the institution or site where the agent will be prepared and administered. Sponsor does not permit the transfer of agents between institutions (unless prior approval from sponsor is obtained). Agents are requested by completing the sponsor Clinical Drug Request form (to include complete shipping contact information). Please refer to the I-SPY 2 Manual of Operations for completion and submission guidelines.

NCI's procedures for agent distribution and the required forms are available in the I-SPY 2 Study Coordinator Manual.

3.5 Investigational Agent Preparation and Handling

Vials should be visually inspected upon receipt to ensure that they are intact without exterior contamination.

The solution of pertuzumab for infusion, diluted in PVC or non-PVC polyolefin bags containing 0.9% Sodium Chloride Injection, USP, may be stored for up to 24 hours prior to use. The diluted solution should be stored refrigerated (2°C–8°C). Because the formulation does not contain a preservative, the vial seal may only be punctured once. Any remaining solution should be discarded.

T-DM1 is provided as a lyophilized drug product and should be reconstituted using sWFI. The lyophilized drug product, after reconstitution with sWFI, contains 20 mg/mL T-DM1. The reconstituted product contains no preservative and is intended for single use only.

CONFIDENTIAL

Using a new syringe, add 8.0 mL of sWFI to the 20-mL (160 mg) T-DM1 vial configuration, or 5.0 mL of sWFI to the 15-mL (100 mg) trastuzumab configuration vial. Swirl gently until completely dissolved (do not shake vigorously). Inspect the vials to ensure the product is clear and free of particulates before proceeding. The stopper of lyophilized product vials should be punctured once to introduce the SWFI and once to remove the reconstituted product. As the reconstituted vials do not contain any preservative, they should be used within 1 hour of reconstitution. (See §3.8 for storage requirements.)

Vials that have been used for one participant may not be used for any other participant.

To prepare the infusion solution from the reconstituted lyophilized product, using a new syringe, remove the indicated volume of product from the vials, based on participant weight, and add to the iv bag. Gently invert the bag to mix the solution. Do not shake vigorously. Reconstituted T-DM1 should be diluted into polyvinyl chloride (PVC), latex-free PVC-free polyolefin bags (PO), polypropylene (PP) or polyethylene (PE) bags containing 0.45% or 0.9% sodium chloride injection (minimum volume of 250 mL). The use of PVC, PO, PP or PE bags containing 0.45% sodium chloride is preferred. When PVC, PO, PP or PE bags containing 0.9% sodium chloride are used, a 0.22 μm in-line non-protein adsorptive polyethersulfone (PES) filter is required. It is recommended to use 0.22 μm in-line PES filters also when using PVC, PO, PP or PE bags containing 0.45% sodium chloride.

From a microbiological point of view, the diluted T-DM1 in infusion bags should be used immediately. If not used immediately, in-use storage times and conditions are the responsibility of the user. Discard any vials containing unused product whose septum has been pierced, as the product does not contain preservative. See T-DM1 Investigator's Brochure [10]§3.3 for complete instructions regarding appropriate dose solution preparation and storage conditions.

3.6 Investigational Agent Accountability

The investigator, or a responsible party designated by the investigator, must maintain a careful record of the inventory and disposition of all agents received from manufacturer using the Drug Accountability Record Form. Copy of the form is available in the I-SPY 2 Study Coordinator Manual. The investigator is required to maintain adequate records of receipt, dispensing and final disposition of study agent. Include on receipt record from whom the agent was received and to whom study agent was shipped, date, quantity and batch or lot number. On dispensing record, note quantities and dates study agent was dispensed to and returned by each participant.

3.7 Investigational Agent Packaging And Labels

Pertuzumab and T-DM1 are packaged and labeled by Genentech according to their established procedures. Labels are printed and attached to the study drug vial or other packaging container prior to shipping to the site. Each will be labeled with a single panel label that will include, but is not limited to, the following information:

- Blank spaces to write the study number and investigator name
- IND caution statement
- Drug identification
- Lot number
- Storage conditions
- Dosing instructions
- Blank spaces to write the participant's identification number, initials and date dispensed

Each label must remain affixed to the bottle or vial.

CONFIDENTIAL

3.8 Storage

Pertuzumab vials are to be refrigerated at 2°C–8°C (36°F–46°F). Vial contents should be protected from light, and should not be frozen. Vials are single use.

T-DM1 vials are to be refrigerated at 2°C–8°C (36°F–46°F) and should remain refrigerated until use. Do not freeze vials. Protect the vials from light. T-DM1 vials should not be used beyond the expiration date provided by the manufacturer.

The reconstituted lyophilized vials should be used within 1 hour of reconstitution with SWFI. If not used within this time frame, the reconstituted vials can be stored for up to 24 hours in a refrigerator at 2°C–8°C. Vials stored beyond this time period should be discarded.

Once reconstituted, T-DM1 solution is diluted into PVC, latex-free PVC-free, PO, PP or PE bags containing 0.45% or 0.9% sodium chloride injection. From a microbiological point of view, the diluted T-DM1 in infusion bags should be used immediately. If not used immediately, in-use storage times and conditions are the responsibility of the user and should not be longer than 24 hours at 2°C–8°C.

Please note that both the trastuzumab 150 mg vial and the trastuzumab emtansine 160 mg vial are available in 20 mL vials and contain a lyophilized product. The vials have different plastic flip-off cap colors: the trastuzumab 150 mg vial is red while the trastuzumab emtansine is white or purple. Both require reconstitution with sterile water for injection, which is provided by the investigator (8.0 mL for trastuzumab emtansine 160 mg and 7.2 mL for trastuzumab). The two vials are similar in appearance and it is important to check the box and vial labels to ensure that the product being administered is consistent with what has been assigned to the patient.

3.9 Agent Destruction/Disposal

Once drug accountability is performed for all returned study agent bottles/containers, the unused study agent will be returned to Genentech according to the Genentech's procedures. This information is also available in the I-SPY 2 Study Coordinator Manual. The unused study agent material will be destroyed by Genentech according to their procedures.

CONFIDENTIAL

References

1. Franklin MC, Carey KD, Vajdos FF, Leahy DJ, de Vos AM, Sliwkowski MX. Insights into ErbB signaling from the structure of the ErbB2-pertuzumab complex. Cancer Cell 2004;5:317–28.
2. Agus DB, Gordon MS, Taylor C, Natale RB, Karlan B, Mendelson DS, Press MF, Allison DE, Sliwkowski MX, Lieberman G, Kelsey SM, Fyfe G. Phase I clinical study of pertuzumab, a novel HER dimerization inhibitor, in patients with advanced cancer. c 2005;23:2534–43.
3. Scheuer W, Friess T, Burtscher H, Bossenmaier B, Endl J, Hasmann M. Strongly enhanced antitumor activity of trastuzumab and pertuzumab combination treatment on HER2-positive human xenograft tumor models. Cancer Res 2009;69:9330–36.
4. Baselga J, Gelmon KA, Verma S, Wardley A, Conte P, Miles D, Bianchi G, Cortes J, McNally VA, Ross GA, Fumoleau P, Gianni L. Phase II trial of pertuzumab and trastuzumab in patients with human epidermal growth factor receptor 2-positive metastatic breast cancer that progressed during prior trastuzumab therapy. J Clin Oncol 2010;28:1138–44.
5. Gianni L, Pienkowski T, Im Y-H, Roman L, Tseng L-M, Liu M-C, Lluch A, Staroslawska E, de la Haba-Rodriguez J, Im S-A, Pedrini JL, Poirier B, Morandi P, Semiglazov V, Srimuninnimit V, Bianchi G, Szado T, Ratnayake J. Efficacy and safety of neoadjuvant pertuzumab and trastuzumab in women with locally advanced, inflammatory, or early HER2-positive breast cancer (NeoSphere): A randomised multicentre, open-label, phase 2 trial. Lancet Oncol 2012;13:25–32.
6. Fan Z, Mendelsohn J. Therapeutic application of anti-growth factor receptor antibodies. Curr Opin Oncol 1998;10:67–73.
7. Sithanandam G, Anderson LM. The ERBB3 receptor in cancer and cancer gene therapy. Cancer Gene Ther 2008;15:413–48.
8. Riese DJ 2nd, van Raaij TM, Plowman GD, Andrews GC, Stern DF. The cellular response to neuregulins is governed by complex interactions of the erbB receptor family. Mol Cell Biol 1995;15:5770–76.
9. Junttila TT, Akita RW, Parsons K, Fields C, Lewis Phillips GD, Friedman LS, Sampath D, Sliwkowski MX. Ligand-independent HER2/HER3/PI3K complex is disrupted by trastuzumab and is effectively inhibited by the PI3K inhibitor GDC-0941. Cancer Cell 2009;15:429–40.
10. Genentech Inc., F. Hoffmann-La Roche Ltd. Investigator's brochure: Trastuzumab emtansine (T-DM1, trastuzumab-MCC-DM1, PRO132365, RO5304020). 9th version, December 2014.
11. Jordan MA, Wendell K, Gardiner S, Derry WB, Copp H, Wilson L. Mitotic block induced in HeLa cells by low concentrations of paclitaxel (Taxol) results in abnormal mitotic exit and apoptotic cell death. Cancer Res 1996;56:816–25.
12. Weaver BA, Cleveland DW. Decoding the links between mitosis, cancer, and chemotherapy: The mitotic checkpoint, adaptation, and cell death. Cancer Cell 2005;8:7–12.
13. Cassady JM, Chan KK, Floss HG, Leistner E. Recent developments in the maytansinoid antitumor agents. Chem Pharm Bull 2004;52:1–26.
14. Slamon DJ, Clark GM, Wong SG, Levin WJ, Ullrich A, McGuire WL. Human breast cancer: Correlation of relapse and survival with amplification of the HER-2/*neu* oncogene. Science 1987;235:177–82.
15. Slamon DJ, Leyland-Jones B, Shak S, Fuchs H, Paton V, Bajamonde A, Fleming T, Eiermann W, Wolter J, Pegram M, Baselga J, Norton L. Use of chemotherapy plus a monoclonal antibody against HER2 for metastatic breast cancer that overexpresses HER2. N Engl J Med 2001;344:783–92.
16. Lewis Phillips GD, La Fleur M, Totpal K, Chiari RJ, Sliwkowski MX. *In vitro* efficacy of herceptin-maytansinoid conjugate on human breast tumor cells. Proc AACR-NCI-EORTC 2001;abst. No. 664.
17. Erickson HK, Park PU, Widdison WC, Kovtun YV, Garrett LM, Hoffman K, Lutz RJ, Goldmacher VS, Blattler WA. Antibody-maytansinoid conjugates are activated in targeted cancer cells by lysosomal degradation and linker-dependent intracellular processing. Cancer Res 2006;66:4426–33.
18. Neidhart JA, Laufman LR, Vaughn C, McCracken JD. Minimal single-agent activity of maytansine in refractory breast cancer: A Southwest Oncology Group study. Cancer Treat Rep 1980;64:675–77.
19. Edmonson JH, Rubin J, Kvols LK, O'Connell MJ, Frytak S, Green SJ. Phase II study of maytansine in advanced breast cancer. Cancer Treat Rep 1981;65:536–37.
20. Cabanillas F, Bodey GP, Burgess MA, Freireich EJ. Results of a phase II study of maytansine in patients with breast carcinoma and melanoma. Cancer Treat Rep 1979;63:507–09.
21. Mathew J, Perez EA. Trastuzumab emtansine in human epidermal growth factor receptor 2-positive breast cancer: A review. Curr Opin Oncol 2011;23:594–600.

CONFIDENTIAL

22. Junttila TT, Li G, Parsons K, Phillips GL, Sliwkowski MX. Trastuzumab-DM1 (T-DM1) retains all the mechanisms of action of trastuzumab and efficiently inhibits growth of lapatinib insensitive breast cancer. Breast Cancer Res Treat 2011;128:347–56.
23. Krop IE, Beeram M, Modi S, Jones SF, Holden SN, Yu W, Girish S, Tibbitts J, Yi J-H, Sliwkowski MX, Jacobson F, Lutzker SG, Burris HA. Phase I study of trastuzumab-DM1, an HER2 antibody-drug conjugate, given every 3 weeks to patients with HER2-positive metastatic breast cancer. J Clin Oncol 2010;28:2698–704.
24. Burris HA 3rd, Rugo HS, Vukelja SJ, Vogel CL, Borson RA, Limentani S, Tan-Chiu E, Krop IE, Michaelson RA, Girish S, Amler L, Zheng M, Chu Y-W, Klencke B, O'Shaughnessy JA. Phase II study of the antibody drug conjugate trastuzumab-DM1 for the treatment of human epidermal growth factor receptor 2 (HER2)-positive breast cancer after prior HER2-directed therapy. J Clin Oncol 2011;29:398–405.
25. Verma S, Miles D, Gianni L, Krop IE, Welslau M, Baselga J, Pegram M, Oh D-Y, Dieras V, Guardino E, Fang L, Lu MW, Olsen S, Blackwell K. Trastuzumab emtansine for HER2-positive advanced breast cancer. N Engl J Med 2012;367:1783–91.
26. Olson EM, Lin NU, DiPiro PJ, Najita JS, Krop IE, Winer EP, Burstein HJ. Responses to subsequent anti-HER2 therapy after treatment with trastuzumab-DM1 in women with HER2-positive metastatic breast cancer. Ann Oncol 2012;23:93–97
27. ClinicalTrials.gov. ClinicalTrials.gov: A service of the U.S. National Institutes of Health. Accessed on 03/12/2015 at http://clinicaltrials.gov.
28. Ellis PA, Barrios CH, Im Y, Patre M, Branle F, Perez EA. MARIANNE: A phase III, randomized study of trastuzumab-DM1 (T-DM1) with or without pertuzumab (P) compared with trastuzumab (H) plus taxane for first-line treatment of HER2-positive, progressive, or recurrent locally advanced or metastatic breast cancer (MBC). J Clin Oncol 2011;29 (Suppl.):abst. No. TPS102.
29. Dieras V, Harbeck N, Budd GT, Greenson JK, Guardino E, Samant M, Chernyukhin N, Smitt M, Krop IE. Trastuzumab emtansine (T-DM1) in human epidermal growth factor receptor 2-positive metastatic breast cancer: Pooled safety analysis. San Antonio Breast Cancer Symposium 2012;N/A:abstr P5-18-06.
30. Genentech Inc., F. Hoffmann-La Roche Ltd. Investigator's brochure: Trastuzumab emtansine (T-DM1, trastuzumab-MCC-DM1, PRO132365, RO5304020). 7th version, December 2012.
31. Dang CT, Gianni L, Romieu G, Dirix L, Campone M, Citron ML, Zamagni C, Krop IE, Xu N, Smitt M, Suter T. Cardiac safety in a phase II study of trastuzumab emtansine (T-DM1) following anthracycline-based chemotherapy as adjuvant or neoadjuvant therapy for early-stage HER2-positive breast cancer. J Clin Oncol 2012;30 (suppl):abst. No. 532.
32. Beeram M, Burris HA, Modi S, Birkner M, Girish S, Tibbitts J, Holden SN, Lutzker SG, Krop IE. A phase I study of trastuzumab-DM1 (T-DM1), a first-in-class HER2 antibody-drug conjugate (ADC), in patients (pts) with advanced HER2+ breast cancer (BC). J Clin Oncol 2008;26 (suppl):abst. No. 1028.
33. Holden SN, Beeram M, Krop IE, Burris HA, Birkner M, Girish S, Tibbitts J, Lutzker SG, Modi S. A phase I study of weekly dosing of trastuzumab-DM1 (T-DM1) in patients (pts) with advanced HER2+ breast cancer (BC). J Clin Oncol 2008;26 (suppl):abst. No. 1029.
34. Girish S, Gupta M, Wang B, Lu D, Krop IE, Vogel CL, Burris Iii HA, Lorusso PM, Yi JH, Saad O, Tong B, Chu YW, Holden S, Joshi A. Clinical pharmacology of trastuzumab emtansine (T-DM1): an antibody-drug conjugate in development for the treatment of HER2-positive cancer. Cancer Chemother Pharmacol 2012;Jan 20:Epub ahead of print.
35. Gupta M, Lorusso PM, Wang B, Yi JH, Burris HA r, Beeram M, Modi S, Chu YW, Agresta S, Klencke B, Joshi A, Girish S. Clinical implications of pathophysiological and demographic covariates on the population pharmacokinetics of trastuzumab emtansine, a HER2-targeted antibody-drug conjugate, in patients with HER2-positive metastatic breast cancer. J Clin Pharmacol 2011;Sep 27:Epub ahead of print.

CONFIDENTIAL

CONFIDENTIAL

Quality of Life Survey

EORTC 30

**During the past week:**	**Not at all**	**A Little**	**Quite a bit**	**Very Much**
1. Do you have any trouble doing strenuous activities, like carrying a heavy shopping bag or suitcase?	1	2	3	4
2. Do you have any trouble taking a long walk?	1	2	3	4
3. Do you have any trouble taking a short walk outside of the house?	1	2	3	4
4. Do you need to stay in bed or a chair during the day?	1	2	3	4
5. Do you need help with eating, dressing, washing yourself or using the toilet?	1	2	3	4
6. Were you limited in doing either your work or other daily activities?	1	2	3	4
7. Were you limited in pursuing your hobbies or other leisure time activities?	1	2	3	4
8. Were you short of breath?	1	2	3	4
9. Have you had pain?	1	2	3	4
10. Did you need to rest?	1	2	3	4
11. Have you had trouble sleeping?	1	2	3	4
12. Have you felt weak?	1	2	3	4
13. Have you lacked appetite?	1	2	3	4
14. Have you felt nauseated?	1	2	3	4
15. Have you vomited?	1	2	3	4
16. Have you been constipated?	1	2	3	4
17. Have you had diarrhea?	1	2	3	4
18. Were you tired?	1	2	3	4
19. Did pain interfere with your daily activities?	1	2	3	4
20. Have you had difficulty in concentrating on things, like reading a newspaper or watching television?	1	2	3	4
21. Did you feel tense?	1	2	3	4
22. Did you worry?	1	2	3	4
23. Did you feel irritable?	1	2	3	4
24. Did you feel depressed?	1	2	3	4
25. Have you had difficulty remembering things?	1	2	3	4
26. Has your physical condition or medical treatment interfered with your family life?	1	2	3	4
27. Has your physical condition or medical treatment interfered with your social activities?	1	2	3	4
28. Has your physical condition or medical treatment caused you financial difficulties?	1	2	3	4

For the following questions please circle the number between 1 and 7 that best applies to you

29. How would you rate your overall health during the past week?

1 2 3 4 5 6 7

Very poor Excellent

30. How would you rate your overall quality of life during the past week?

1 2 3 4 5 6 7

Very poor Excellent

CONFIDENTIAL

EORTC BR23

During the past week	Not at All	A Little	Quite a Bit	Very Much
Did you have a dry mouth?	1	2	3	4
Did food and drink taste different than usual?	1	2	3	4
Were your eyes painful, irritated or watery?	1	2	3	4
Have you lost any hair?	1	2	3	4
Answer this question only if you had any hair loss: Were you upset by the loss of your hair?	1	2	3	4
Did you feel ill or unwell?	1	2	3	4
Did you have hot flashes?	1	2	3	4
Did you have headaches?	1	2	3	4
Have you felt physically less attractive as a result of your disease or treatment?	1	2	3	4
Have you been feeling less feminine as a result of your disease or treatment?	1	2	3	4
Did you find it difficult to look at yourself naked?	1	2	3	4
Have you been dissatisfied with your body?	1	2	3	4
Were you worried about your health in the future?	1	2	3	4

During the past 4 weeks:	Not at All	A Little	Quite a Bit	Very Much
To what extent were you interested in sex?	1	2	3	4
To what extent were you sexually active? (with or without intercourse)?	1	2	3	4
To what extent was sex enjoyable for you?	1	2	3	4

During the past week:	Not at All	A Little	Quite a Bit	Very Much
Did you have any pain in your arm or shoulder?	1	2	3	4
Did you have a swollen arm or hand?	1	2	3	4
Was it difficult to raise your arm or to move it sideways?	1	2	3	4
Have you had any pain in the area of your affected breast?	1	2	3	4
Was the area of your affected breast swollen?	1	2	3	4
Was the area of your affected breast oversensitive?	1	2	3	4
Have you had skin problems on or in the area of your affected breast (e.g., itchy, dry, flaky)?	1	2	3	4

Fear of Recurrence Scale (Only items 1 and 2 will be used preoperatively)

1. Because cancer is unpredictable, I feel I cannot plan for the future
2. My cancer will probably come back in 5 years or I will probably have a relapse in the next 5 years.
3. My fear of having my cancer coming back gets in the way of my enjoying life.
4. I am afraid of my cancer coming back.
5. I am certain I have been cured of cancer.

CONFIDENTIAL

PROMIS Physical Functioning					
**During the past week:**	**Without any difficulty**	**With a little difficulty**	**With some difficulty**	**With much difficulty**	**Not at all**
Are you able to do chores such as vacuuming or yard work?	1	2	3	4	5
Are you able to go up and down stairs at a normal pace?	1	2	3	4	5
Are you able to go for a walk of at least 15 minutes?	1	2	3	4	5
Are you able to run errands and shop?	1	2	5	4	5

PROMIS Anxiety					
**During the past week:**	**Never**	**Rarely**	**Sometimes**	**Often**	**Always**
I felt fearful	1	2	3	4	5
I found it hard to focus on anything other than my anxiety	1	2	3	4	5
My worries overwhelmed me	1	2	3	4	5
I felt nervous	1	2	3	4	5
I felt like I needed help for my anxiety	1	2	3	4	5
I had sudden feelings of panic	1	2	3	4	5
I felt indecisive	1	2	3	4	5
I had difficulty calming down	1	2	3	4	5

PROMIS Depression					
**During the past week**	**Never**	**Rarely**	**Sometimes**	**Often**	**Always**
I felt worthless	1	2	3	4	5
I felt helpless	1	2	3	4	5
I felt depressed	1	2	3	4	5
I felt hopeless	1	2	3	4	5
I felt unhappy	1	2	3	4	5
I felt that I had nothing to look forward to	1	2	3	4	5
I withdrew from other people	1	2	3	4	5
I found that things in my life were overwhelming	1	2	3	4	5
I felt worthless	1	2	3	4	5

PROMIS Fatigue					
**During the past week**	**Never**	**Rarely**	**Sometimes**	**Often**	**Always**
I feel fatigued	1	2	3	4	5
How much were you bothered by your fatigue on average?	1	2	3	4	5
To what degree did your fatigue interfere with your physical functioning?	1	2	3	4	5
To what degree did you have to push your get things done because of your fatigue?	1	2	3	4	5
Did fatigue make you less effective at home?	1	2	3	4	5
How exhausted were you on average?	1	2	3	4	5
How often did you feel tired even when you hadn't done anything?	1	2	3	4	5

CONFIDENTIAL

PROMIS Fatigue					
**During the past week**	**Never**	**Rarely**	**Sometimes**	**Often**	**Always**
How often were you too tired to think clearly?	1	2	3	4	5
I feel fatigued	1	2	3	4	5

PROMIS Applied Cognition					
	**Never**	**Rarely (once)**	**Sometimes (2 or 3 times)**	**Often (about once a day)**	**Always (several times a day)**
My thinking has been slow	1	2	3	4	5
It has seemed like my brain was not working as well as usual	1	2	3	4	5
I have had to work harder than usual to keep track of what I was doing	1	2	3	4	5
I have had trouble shifting back and forth between different activities that require thinking	1	2	3	4	5
I have had trouble concentrating	1	2	3	4	5
I have had to work really hard to pay attention or I would make a mistake	1	2	3	4	5
I have had trouble forming thoughts	1	2	3	4	5
My problems with memory, concentration, or making mental mistakes have interfered with the quality of my life	1	2	3	4	5
My thinking has been slow	1	2	3	4	5

PROMIS Social Roles					
**During the past week (last 7 days)**	**Not at all**	**A little bit**	**Somewhat**	**Quite a bit**	**Very much**
I am satisfied with my ability to meet the needs of those who depend on me					
I am satisfied with how much work I can do (include work at home)					
I am satisfied with my ability to do household chores/tasks					
I am satisfied with the amount of time I spend performing my daily routines					

PROMIS Female Sexual Functioning						
**During the past 30 days (4 weeks)**		**Not at all**	**A little bit**	**Somewhat**	**Quite a bit**	**Always**
How interested have you been in sexual activity?		1	2	3	4	5
**During the past 30 days (4 weeks)**		**Never**	**Rarely**	**Sometimes**	**Often**	**Always**
How often have you felt like you wanted to have sex?		1	2	3	4	5

CONFIDENTIAL

PROMIS Female Sexual Functioning						
	**No sexual activity**	**Almost always or always**	**Most times (more than half the time)**	**Sometimes (about half the time)**	**A few times (less than half the time)**	**Almost never or never**
How often did you become lubricated ("wet") during sexual activity or intercourse?	0	5	4	3	2	1
	**Have not tried to get lubricated in the past 30 days**	**Not at all**	**A little bit**	**Somewhat**	**Quite a bit**	**Very**
How difficult has it been for your vagina to get lubricated ("wet") when you wanted it to?	0	5	4	3	2	1
	**Have not had any sexual activity in the past 30 days**	**Very comfort-able**	**Comfort-able**	**Uncom-fortable**	**Very uncomfort-able**	
How would you describe the comfort of your vagina during sexual activity?	0	1	2	3	4	
	**Have not had any sexual activity in the past 30 days**	**Never**	**Rarely**	**Sometimes**	**Often**	**Always**
How often have you had difficulty with sexual activity because of discomfort or pain in your vagina?	0	1	2	3	4	5
How often have you stopped sexual activity because of discomfort or pain in your vagina?	0	1	2	3	4	5
	**Have not tried to have an orgasm/ climax in the past 30 days**	**Excellent**	**Very good**	**Good**	**Fair**	**Poor**
How would you rate your ability to have a satisfying orgasm/climax?	0	5	4	3	2	1
	**Have not had sexual activity in the past 30 days**	**Not at all**	**A little bit**	**Somewhat**	**Quite a bit**	**Very Much**
When you have had sexual activity, how much have you enjoyed it?	0	1	2	3	4	5

CONFIDENTIAL

PROMIS Female Sexual Functioning						
	**Have not had sexual activity in the past 30 days**	**Not at all**	**A little bit**	**Somewhat**	**Quite a bit**	**Very**
When you have had sexual activity, how satisfying has it been?	0	1	2	3	4	5

CONFIDENTIAL

Supplement 3
SURMOUNT study within the I-SPY 2 Study

SURMOUNT: Surveillance Markers of Utility for Recurrence after Neoadujvant Therapy for Breast Cancer

1.0 Background

Adjuvant chemotherapy has been clearly established to improve cure rates in early stage breast cancer, but is not universally effective. Despite this therapy, many women subsequently relapse over years to decades following diagnosis. Neoadjuvant chemotherapy is increasingly used in the primary treatment of non-metastatic breast cancer because of its ability to downstage patients for improved surgical outcomes and provides an in vivo assessment of tumor chemosensitivity. Several measures, including pathological response (pCR) and residual cancer burden (RCB) provide proximate surrogate endpoints that reflect later outcomes [1]. While those with pCR or RCB 0/1 have excellent prognosis overall, less than 50% of patients attain this status, and those without such response have a high likelihood of recurrence. Patients with Her2+ or triple negative breast cancer who do not achieve pCR (approximately 40% and 65% of patients, respectively) have a particularly poor prognosis, with greater than 50% of patients demonstrating distant relapse within 5 years [2].

1.1. DTCs and CTCs as Prognostic Markers in Early Breast Cancer

Currently, there is no useful test or biomarker that can be used after standard (neo)adjuvant therapy to monitor patients and accurately identify those who will recur before the appearance of overt metastatic disease. Radiologic scanning and circulating tumor markers, such as CA27-29 and CA15-3, are not effective and currently not recommended by the American Society of Clinical Oncology for use in the surveillance period[3]. This lack of ability to monitor for residual or recurrent cells at a point where intervention could be successful leads to enormous distress for patients [4-6] and limits the ability to fully utilize the arsenal of effective agents to prevent subsequently incurable metastatic disease. Thus, there is critical need for accurate and effective biomarkers for recurrence in the surveillance period.

New and emerging technologies make it possible to sensitively detect circulating tumor cells (CTCs) in peripheral blood and/or disseminated tumor cells (DTCs) in bone marrow of patients with cancer. Residual cancer cells in the bone marrow have proven clinical relevance for relapse based on the analysis of thousands of breast cancer patients[10,11]. Accordingly, at present bone marrow DTCs represent the best biological surrogate for the population of cells that ultimately gives rise to recurrence. Early evidence for the prognostic significance of DTCs in breast cancer patients emerged from a pooled analysis involving 4,703 patients with stage 1, 2, or 3 breast cancer in whom DTCs were identified in 31% of patients[12]. This study demonstrated that the presence of DTCs in bone marrow predicts poor breast cancer-specific survival, poor overall survival, and a high risk of local and loco-regional recurrence[12-14]. In fact, in multivariate analysis, bone marrow status was the strongest independent predictor of disease-free and overall survival. Additional studies have revealed that the presence and persistence of DTCs in bone marrow after adjuvant therapy predicts poor prognosis[15-18].

However, since serial bone marrow biopsy is a painful and challenging clinical endpoint to incorporate into therapeutic trials and routine clinical care, attention has turned to circulating tumor cells (CTCs) as an alternative[19-21]. CTCs have proven prognostic relevance in metastatic breast cancer[19,22] and their clinical value for monitoring therapy is a topic of intensive investigation[23,24]. Indeed, the CellSearch™ system has been approved by the FDA for this purpose and CTCs detected by this method have proven prognostic relevance in metastatic breast cancer[19,22]. For example, in pioneering work Cristofanilli et al. demonstrated that the presence of ≥5 CTC per 7.5 mL blood detected using the CellSearch™ system was associated with poor prognosis in metastatic breast cancer patients[19]. Unfavorable CTC counts (≥ 5 CTC/7.5 mL blood)

CONFIDENTIAL

that persisted after the first cycle of chemotherapy also predicted poor clinical outcome[22]. Numerous studies in the metastatic setting have demonstrated the presence of CTCs and correlated their presence with response to therapy and outcome[19,25,26].

More recently, studies have focused on the identification of CTCs and DTCs in women with early breast cancer in an effort to identify those at high risk of relapse[27,28]. However, the rate of detection of CTCs using EPCAM-based capture technologies in these studies has been low, typically on the order of 10-15%, reflecting the rarity of these cells in low-risk populations with favorable prognosis[11]. Slade et al. demonstrated that a significant proportion of primary breast cancer patients with poor prognosis (>3 positive nodes) have detectable CTCs[31]. Recently, clearcut data have emerged demonstrating the relevance of CTCs in primary breast cancer patients for predicting early relapse (<5 years after initial diagnosis)[32, 33]. The largest of these studies is the multicenter German SUCCESS trial. This trial is the largest adjuvant breast cancer study to date to monitor CTCs and it found that the presence of CTCs detected by CellSearch™ prior to systemic treatment is an independent predictor for recurrence-free and overall survival for women within 5 years of diagnosis[33]. This trial enrolled 3753 patients, analyzed blood from 2026 patients for CTCs, and banked CTCs, serum, and plasma for molecular analyses. CTCs were analyzed following complete resection of the primary tumor and prior to the initiation of systemic adjuvant treatment. All patients were randomized in the SUCCESS A Study to receive 3 cycles of epirubicin, fluorouracil and cyclophosphamide chemotherapy followed by 3 cycles of either docetaxel (FEC-D) or docetaxel and gemcitabine (FEC-DG). Patients were followed for a median of 35 months (range 0 to 54 months). Cox regression models were used to assess the prognostic significance of CTCs for disease-free and overall survival. CTCs were detected in 21.5% of patients (n=435; median 1.3, range 1-827). 114 patients developed recurrent breast cancer and 66 patients died of their disease. The presence of CTCs (≥1 CTC per 23 ml blood) was an independent predictor of poor disease-free survival (p<0.0001), distant disease-free survival (p<0.001) and overall survival (p=0.0002). Multivariate analysis revealed that CTC detection was an independent prognostic factor with worst prognosis in patients with ≥ 5 CTCs (HR 4.0 [95%CI 2.21-7.07] for DFS; HR 3.1 [95%CI 1.51-8.28] for OAS). This is the first study to demonstrate the independent prognostic relevance of CTCs for early relapse in a large prospective study of primary breast cancer patients.

1.2. DTCs and CTCs as Prognostic Markers in the Neoadjuvant Setting

In neoadjuvant trials, where participants typically have larger tumors with more aggressive features, reported CTC detection rates are higher, on the order of 20-30%[20,29,30]. In this patient population, two of three studies showed that the presence of CTCs before and after neoadjvuant therapy is prognostic for outcome[20,30] and this endpoint is increasingly being incorporated into neoadjuvant trials. In the neoadjuvant setting, both pCR and RCB have been validated as prognostic surrogates for relapse-free survival, but little is known about the relationship between pathologic response, presence/absence of DTCs or CTCs and clinical outcome. In the recently completed German neoadjuvant trials "GeparQuattro" and "GeparQuinto", which represent the largest published trials to date in this context, CTC determinations were performed before and after primary systemic chemotherapy. The positivity rate, defined as the detection of one or more CTCs/7.5 mL blood, was 22% before chemotherapy and 11% after chemotherapy[29,34]. Mathieson and colleagues also have examined the presence of and alterations in DTC and CTC status in locally advanced breast cancer patients undergoing neoadjuvant chemotherapy and to evaluate their prognostic impact, albeit with NACT consisting of either single-agent epirubicin or paclitaxel, regimens not currently the standard of care [35,36]. Bone marrow and peripheral blood were collected before NACT (BM1: n = 231/PB1: n = 219), at surgery (BM2: n = 69/PB2: n = 71), and after 12 months from start of NACT (BM3: n = 162/PB3: n = 141). Patients were included from 1997 to 2003 and followed until 2009 (or ten years follow-up). DTC- and CTC-status were determined by morphological evaluation of immunocytochemically detected cytokeratin-positive cells. The prognostic significance of DTCs/CTCs was assessed by univariate and multivariate Cox-regression analyses. Before NACT, DTCs and CTCs were detected in 21.2% and 4.9% of the patients, respectively. At surgery, 15.9% and 1.4% had

CONFIDENTIAL

DTC- and CTC-presence, compared to 26.5% and 4.3% at 12 months from start of NACT. Of patients for whom DTC results both before NACT and at 12 months were available, concordant results were observed in 68%, and 14 out of 65 had positive DTC-status at both time points. Presence of ≥ 1 DTC 12 months from start of NACT, but not at other time points, predicted reduced disease-free survival (DFS; HR 2.3, $p = 0.003$), breast cancer-specific survival (BCSS; HR 3.0, $p < 0.001$) and overall survival (OS; HR 2.8, $p < 0.001$). Before NACT, presence of ≥ 3 DTCs was also associated with unfavorable outcome, and reduced BCSS was observed for CTC-positive patients (HR 2.2, $p = 0.046$). In multivariate analysis, DTC status (</≥ 1 DTC) at 12 months after start of NACT remained as a prognostic factor for both DFS (HR 2.2, $p = 0.005$), BCSS (HR 2.6, $p = 0.002$) and OS (HR 2.6, $p = 0.002$). The survival for patients with change in DTC-status was determined by the DTC-status at 12 months. Understanding the relationship between these surrogates, DTCs or CTCs and disease recurrence would be invaluable as a way to identify those patients likely to have early relapse, as well as providing the opportunity to intervene before overt metastatic disease occurs.

1.3. Linking Surveillance Markers to Metastatic Biology

Current theories of metastasis suggest an important role for tumor cell dissemination from the primary tumor, though precise mechanisms remain unclear [7-9]. It is increasingly recognized that tumor biology of metastatic relapse differs from the primary tumor in a subset of cases. This can range from discordance in ER/PR or Her2 expression[37,38] to extensive genomic differences [39]. However, it is currently unknown whether residual tumors after neoadjuvant chemotherapy more closely resemble cells that are circulating after therapy and/or those that subsequently manifest as distant metastases. Preclinical studies in transgenic mouse models from the laboratory of Dr. Lewis Chodosh demonstrate a distinct population of cells that arise after complete regression of primary tumors and cessation of oncogenic blockade. These cells demonstrate a variety of molecular features that differ from both the primary tumor and subsequent metastases. Preliminary human studies have recently shown that CTCs and metastatic tumors in patients after relapse can share some features, but data remain sparse in this area. We have demonstrated that potential targets can be identified within residual tumors of patients from I-SPY1 (unpublished data), and that these tumors are fundamentally different in their gene expression patterns than the primary tumors from which they arose. For example, a comparison of baseline (T1) and residual tumors (T4) revealed differential expression of 41 probes (34 unique annotated genes) (n=44, paired t-test, limited to probes with at least 3 evaluable pairs, with Benjamini Hochberg FDR-corrected $p < 0.05$). Genes found to be more highly expressed in T4 relative to T1 included Jun, Fos, EGFR, CYR61 and CTGF, while those found to have decreased expression at T4 included CD6, IL24, and TRAF3.

1.4. Development of novel approaches to the isolation and analysis of CTCs, DTCs and CTMs to compare to residual tumors

While CellSearch™ is the most well-validated approach to enumerating circulating tumor cells to date, other platforms are being developed for improved capture and the molecular and genetic analysis of these cells and DTCs. For example, CellSearch detects cells based upon expression of EPCAM, limiting detection of cells that may be undergoing EMT. Many alternative capture methods are focused on either increasing capture rate through expanded antibody approaches or identifying cells that have undergone EMT, and therefore no longer express epithelial markers. The former would be useful to reduce the amount of blood currently needed to perform the CellSearch assay. The latter holds appeal given experimental evidence that minimal residual disease and dormant cancer cells pass through a period of EMT that provides mechanisms of self-renewal and resistance to chemotherapy [40,41].

In addition, numerous alternative technologies that negate the need to capture whole cells are also currently under development or undergoing validation, including the isolation of micro-RNA[42-44] and cell-free DNA (cfDNA) [45]. These alternative approaches to measuring circulating tumor markers (CTMs) have advantages

CONFIDENTIAL

and disadvantages. Early studies have shown that cfDNA is a sensitive marker of disease in the metastatic breast cancer setting, and may be better than CA-27.29 or CA15.3 in determining changes in tumor burden[45]. Pilot studies have determined that cfDNA can identify known genetic mutations seen in primary tumors. However, controversy exists as to whether this material is viable, and thus whether it constitutes a reservoir of material that ultimately gives rise to metastatic disease.

The goals of this study are to examine all of these types of circulating material to determine the extent to which each reflects the primary or residual tumor in the breast, to determine whether there are unique molecular markers in these cells that represent markers of tumor dormancy and whether the presence of these markers adds prognostic information over and above that obtained from the surgical status of the primary tumor after neoadjuvant therapy. These primary objectives are a necessary foundation to the development of therapeutic approaches in patients with incomplete response to neoadjuvant chemotherapy who are at high risk of recurrence.

2.0 Study Objectives

2.1. Primary Objective

2.1.1. To determine the incidence and frequency of bone marrow and circulating tumor biomarkers in patients who are undergoing neoadjuvant chemotherapy for primary breast cancer and are participating in the I-SPY 2 TRIAL.

2.2. Secondary Objectives

2.2.1. To compare the molecular and genetic features between primary tumor, residual tumor, and DTCs/CTCs/CTMs (Circulating Tumor Markers) in those participants in whom they are detectable

2.2.2. To determine the relationship between detection of DTCs, CTCs, or CTMs and 3-year relapse free survival (RFS), by pathologic response status and receptor status

2.2.3. To determine if the presence or absence of DTCs, CTCs or CTMs adds independent prognostic information to tumor pathologic response (RCB).

3.0. Study Population

3.1. Inclusion Criteria:

Eligible participants will be drawn from those participating in the I-SPY 2 TRIAL who meet the following criteria:

3.1.1. Consented to the treatment phase of the I-SPY 2 TRIAL. Participants who have discontinued assigned treatment on I-SPY 2 are still considered eligible to participate and complete study procedures.

3.1.2. Willing to undergo bone marrow aspiration and blood specimen collection per protocol specifications

3.1.3. No clinical evidence of distant metastatic disease. Pre-chemotherapy staging scans are sufficient in the absence of any symptoms or subsequent clinical evidence suggesting distant metastases

CONFIDENTIAL

3.2. Subject Recruitment and Screening
The study team will describe the SURMOUNT study to patients at any point prior to the patient's final ISPY scheduled surgical procedure. Patients will need to be consented at a visit prior to the scheduled surgery in order to collect the pre-operative research blood..

3.3. Consent Process
Patients can be approached for consent as soon as the site has obtained IRB approval is obtained. The study team will discuss the study and study procedures with the study participant and ask if the potential study participant has any questions. After answering any questions, the study team will ask the potential study participant to provide consent to participate. .

3.4. Early Withdrawal of Participants

At any time a study participant may request to discontinue SURMOUNT participation, either independently or in the context of withdrawing consent from the ISPY2 TRIAL. Participants may elect to (1) Suspend future participation only or (2) Suspend participation and have her specimens destroyed. If the participant elects option 1, the study coordinator will document the participant's decision within the study file. The participant will no longer be contacted for SURMOUNT follow-up, but use of all data collected up to that time, and ongoing medical record reviews may continue. Should the participant opt for option 2, the participant must make this request directly to the Principal Investigator of the SURMOUNT, Angela DeMichele, MD, MSCE or other designated study staff (i.e. project manager or research coordinator or other site principal investigator/co-investigator). The participant has several options for withdrawal. The participant must request in writing that their specimens be destroyed. If data has already been generated from their specimens, the data will remain in the study database. If the specimens have not been used at the time of the request, the specimens will be destroyed. The requests for withdrawal are required to be documented in the RedCap Database and the letter is required to be submitted within 5 business days to the study lead investigator and the project manager. Additional procedures for withdrawal are outlined in the study manual of operations.

Requests for withdrawal of consent from SURMOUNT may be submitted in writing to:

Angela DeMichele, M.D., M.S.C.E.
Rowan Breast Center of the University of Pennsylvania
Perelman Center, 3rd Floor (West)
3400 Civic Center Blvd
Philadelphia, PA 19104

3.5 Return of Research Results
Study participants will be offered the opportunity to receive research results at the completion of the study. If the study participant would like to receive their research results, they will provide consent and address for which to send the results at the completion of the study. All study participants will be informed that the research testing performed in this study is not performed in a CLIA approved laboratory and should not be used to make treatment decisions. They will be informed that these research findings have not been validated and will not be placed in the medical record.

4.0 Study Procedures:

4.1. General Procedures and Study Time-points

CONFIDENTIAL

There is no therapeutic component to the SURMOUNT substudy. Study participants will provide clinical data, blood and bone marrow samples according to the schedule of study activities shown below in Table 1. Table 1 shows the samples that will be collected at each time-point.

Table 1: Schedule of Study Activities:

Study Visit/Timepoint					
**Activity**	Pre-op	Surgery	Post-op	Follow Up	Recurrence
Informed Consent	X				
Blood Draw	X<br><br>CellSave Tube (2 x 10ml)	X<br><br>EDTA Tube (3 x 10ml)	X<br><br>Serum Separator Tube (1x 8.5ml)<br><br>EDTA Tube (1 x 10ml)	X<br><br>Cell Save Tube (1x 10ml)<br><br>Serum Separator Tube (1x 8.5ml)<br><br>EDTA Tube (2 x10ml)	X<br><br>Cell Save Tube (1x 10ml)<br><br>Serum Separator Tube (1x 8.5ml)<br><br>EDTA Tube (2 x10ml)
Bone Marrow Aspirate*		X<br>EDTA Tube<br>1 x 10ml			X<br>EDTA Tube<br>1 x 10ml
Medical Chart Abstraction/Clinical Data Collection				X (Beginning year 6)	X

* Bone marrow aspirates may be collected in a setting outside of the surgical setting. This decision will be at the discretion of the clinical team and patient

CONFIDENTIAL

4.2. Study Visits

Consented participants will be asked to provide a blood sample at several pre-specified time points: 1) “Pre-op” (at the completion of neoadjuvant chemotherapy before surgery), 2) “Surgery” (a blood sample in the operative setting of the participants’ scheduled surgery), 3) “Post-op” (a blood sample during the post-operative visit), 4) “Follow up” (annual blood draws), and 5) “Recurrence” (the collection of blood, and bone marrow if a recurrence occurs). Shipping instructions for all samples are included in the SURMOUNT study Manual of Operations (MOP). The study participant will also be asked to provide a bone marrow aspirate sample at the time of the scheduled surgical procedure removing the residual breast tumor. The participant’s clinical data will be updated through data collected on the I-SPY 2 TRIAL and by SURMOUNT study personnel. If the participant consents, they may also be contacted in the future regarding participation in other research studies for which they may qualify. The study participant will also be asked to allow residual specimens to be kept and banked.

4.2.1. Pre-Operative Visit

This study visit will occur at any time after the last dose of chemotherapy and before the surgical date. Pertinent tracking information, as outlined in the study Manual of Operations, will be subsequently entered into the SURMOUNT Tracking Databases. The study participant will have the pre-operative study blood drawn during the pre-operative visit. Of note, this blood draw can occur at the same time as the required I-SPY 2 pre-surgical blood draw. With the clinical guidance of the treating physician, study participant will decide whether or not to have their bone marrow aspirate as an in-clinic procedure or during their scheduled surgical procedure.

4.2.2. Surgery Visit
This study visit will occur on the day of surgery. The blood specimen and the bone marrow aspirate may be collected before or after surgical removal of the breast tissue. These will be handed off from the OR team to the study team for processing and shipping. Residual tumor tissue will be handled as outlined in the I-SPY 2 protocol; separate tissue will NOT be collected for SURMOUNT.

4.2.3. Post Operative Visit
This visit will take place after surgery but prior to the commencement of radiation therapy. It is recommended that specimen collection occur at the time of the 30-day I-SPY 2 TRIAL follow-up appointment. However, if the site is unable to accommodate the draw at this time, this research blood draw should take place no earlier than 2 weeks post-surgery and no later than 8 weeks post-surgery. At the study participant's post-operative appointment, research blood will be drawn according to Table 1.

4.2.4. Follow-Up Visits
This study visit will occur during the participants annual surveillance follow up visit. The annual visit can take place +/- 60 days from the study participant's yearly surgical anniversary. Participants will be followed annually in order to be evaluated for cancer recurrence events. At annual follow-up, study participants will be asked to provide blood specimens as outlined in the specimen collection table (Table 1). This will continue for a period of 10 years or until recurrence of breast cancer, whichever occurs first.

4.2.5. Recurrence Visit
This visit will occur in the event that the participant develops a local or distant recurrence of breast cancer. Participants will be asked to provide the study team with blood specimens and a bone marrow aspirate specimen. Tissue collection will take place as outlined in the I-SPY 2 TRIAL protocol. These collections are outlined in the schedule of specimen collection table (Table 1).

5.0 Specimen Processing and Laboratory Procedures
The manual of operations will detail the exact methods by which the specimens will be collected, processed, shipped and delivered to the appropriate labs of the study collaborators. Specimen and participant tracking procedures are outlined in the SURMOUNT MOP.

5.1. Whole Blood – Plasma, Buffy Coat, and Serum
Participants will have blood collected at specified time points. The details of blood collection are shown in Table 2. Notably, no study participant will have more than 50 ml of blood drawn at any given specimen collection time point. Blood samples will be collected from every consenting participant enrolled in this study. The blood specimens will be collected for research purposes. In addition to nucleic acid collection, serum, buffy coat, and plasma will be extracted from blood specimens. Study participants have the option to consent to allow the researchers to save leftover blood, serum, buffy coat and plasma samples for future research. Leftover specimens will remain at study laboratories until central study storage has been implemented. Central study specimen storage will be housed at the University of Pennsylvania.

5.2. Bone Marrow Aspiration

CONFIDENTIAL

The bone marrow sample will be obtained either in a non-operative setting (i.e. clinic) by a qualified/trained healthcare provider or at the time of the study subject's scheduled surgery. Additionally, if the participant recurs, a bone marrow aspirate will be collected using the same procedures. If the participant and physician choose to collect the bone marrow sample at the time of breast surgery, it will happen while the participant is already under anesthesia. The participant will be turned to a decubitus position and bone marrow aspirates will be obtained from the posterior iliac crest. Approximately 10mLof aspirate fluid will be obtained in an EDTA-containing tube. Specimens will be shipped overnight as outlined in the SURMOUNT MOP

5.3. Banked Specimens

While the proposed assays will be sufficient to address the objectives of the current study, our understanding of circulating and disseminated tumor biomarkers is constantly evolving, and new technologies are in development and validation. miRNA and cell-free DNA will be assessed at a later point when new technologies have been optimized. Additional banking procedures and changes in preparation and storage procedures may occur as new technologies and techniques evolve. For this reason, data and specimens obtained from study participants will be kept and stored indefinitely.

5.4. Laboratory Assays

Table 2 lists the planned study assays and the laboratories that will be performing them.. Additional details regarding specimen tracking and shipping can be found in the SURMOUNT MOP.

5.4.1. Cell Search (Janssen, Inc)

The CellSearch Epithelial Cell Test will be applied to all samples for the enrichment and enumeration of CTCs. CTCs will be captured from the peripheral blood by anti-EpCAM antibody-bearing ferrofluid and subsequently identified by cytokeratin-positivity plus negativity for the leukocyte common antigen CD45 and 4'6'-diamidino-2-phenylindole (DAPI) staining to ensure the integrity of the nucleus. We will further characterize these cells for ER, Her2 and c-MET expression within the CellSearch system by the addition of FITC-labeled antibodies and use of the additional free channels in the system. Janssen Diagnostics, LLC is providing support for some of the analysis that will be conducted on the study participant's blood samples. This has been disclosed in the informed consent document. Additionally the company may receive de-identified data from this study in accordance with the I-SPY 2 TRIAL clinical data sharing policies and guidelines. We will not be sharing with the company any of the study participant's personal and private information.

5.4.2. IE/FACS and Molecular Profiling

CTCs identified in peripheral blood by the CellSearch™ screen and all bone marrow aspirates will undergo further evaluation for genetic and expression profiling.Tumor cells will be isolated from peripheral blood using IE/FACS as previously reported[49]. We have elected to perform this procedure only on those specimens positive by CellSearch™ screen for efficiency and cost containment, given the high correlation between the CellSearch™ system and that of the Park laboratory. IE/FACs is performed using magnetic beads coated with EpCAM mAb for initial enrichment; the enriched sample is then subjected to FACS using differentially labeled mAbs to distinguish tumor cells (EpCAM+) from leukocytes (CD45+) during sorting.

5.4.3. Molecular profiling of isolated cells:

DNA from isolated tumor cells will be subjected to whole genome amplification (WGA) followed by comparative genomic hybridization (CGH). In addition, we will isolate mRNA for expression profiling across a panel of candidate genes associated with breast cancer progression. We have also used large-scale cDNA arrays to evaluate the transcriptome of CTCs and DTCs. Briefly, total RNA from isolated CTCs or DTCs will be reverse-transcribed into cDNA. 64 genes will be chosen a priori for expression analysis, based on preliminary studies from mouse models. cDNAs of these genes will be pre-amplified and analyzed in triplicate via Taqman®™-based RT-PCR in a low-density array format. Statistical analysis of gene expression data will be performed using Realtime Statminer®. In addition, we will utilize ABI systems

CONFIDENTIAL

customized arrays to expand our assessment to genes of interest from both the gene expression arrays performed on residual tumors. We will only be conducting genetic testing on the tumor and will not be performing germline testing.

Table 2: Study Assays

Sample Type	Assay(s)	Time point(s)	Possible Biomarkers Obtained
Whole Blood	Cell Search	PreOp, Annual F/U, Recurrence	Enumeration (EPCAM markers), ER, Her2, c-MET
Bone Marrow Aspirate	IE/FACS	Surgery, Recurrence	Enumeration, genetic evaluation
Whole Blood	IE/FACS	Surgery, Annual F/U, Recurrence	Enumeration, genetic evaluation
Whole Blood Serum aliquots	Banked for miRNA and others	Post Op Visit, Annual F/U, Recurrence	miRNA profile DNA profile/mutations
Whole Blood Plasma and Buffy coat aliquots	Banked for cell-free DNA, and others	Post Op Visit Annual F/U, Recurrence	DNA profile/mutations

6.0 Clinical Data Management

6.1. Data Obtained from the Electronic Medical Record

Clinical data for SURMOUNT will be collected in the I-SPY 2 TRIAL database system using the online platform SalesForce as outlined in the study protocol. The participant's clinical data will be updated by the I-SPY 2 TRIAL in years 1 to 5 of follow up. At the completion of year 5 of follow-up, SURMOUNT study personnel will update the participant's clinical date during years 6 to 10 of the follow-up period, or at recurrence, if it occurs.

6.2. Case Report Forms

The study case report forms (CRF) are the primary data collection instrument for the study. The CRFs for this study will be completed electronically using a RedCap database system accessible to all study sites. Training will be provided to all sites for proper use of the REDCap Database. The SURMOUNT MOP contains additional details for completing the forms in REDCap.

6.3. Records Retention

Records and specimens will be retained for the entire length of this study. As the relapse rate for many breast cancers remains elevated for over 10 years, participants in this study will be followed annually for 10 years after enrollment. Study documents will be retained by the investigator in a secure storage facility in compliance with Health Insurance Portability and Accountability Act (HIPPA), and Office of Human Research Protections (OHRP), unless the standard at the site is more stringent. Additionally, data and specimens obtained from study participants will be kept and stored indefinitely

CONFIDENTIAL

7.0 Statistical Analysis Plan

7.1. Sample Size Determination

We anticipate enrolling and following 350 women from across the I-SPY 2 study sites. We expect 50% of these participants to have a complete pathological response (pCR or RCB=0) and approximately 50%, or 175, to have residual disease (i.e., RCB > 0). Our primary goal is to estimate the proportion of women in each RCB class with detectable CTCs and DTCs. We are primarily interested in the women with residual disease; if the rate is higher than 50% and we have accrued 175 such women before reaching the target of 350 total, we will suspend accrual. With 175 women with residual disease, the width of the 95% confidence interval around the detection rate will be no more than 0.15 units wide. We expect that among these 175 women, the proportions in the three RCB classes will be 0.35, 0.4, and 0.25, respectively. The maximum width of the 95% CIs in each group will therefore be no more than 0.25, 0.23, and 0.30 units wide, respectively. We also wish to compare the detection rate in these groups. Assuming that women in the RCB 2-3 group will have a detection rate of approximately 40%, we have more than 80% power to detect a decrease in this rate to about 18% in the RCB 1 group. Additional aims include correlation of features of DTCs/CTCs and primary or residual tumor cells. We anticipate that approximately 60% of the women with residual disease, or 105 women, will have detectable DTCs, and that approximately 20%, or 35 women, will have detectable CTCs. With 35 women, we have more than 80% power to detect a correlation coefficient of 0.5 or greater, using a two-sided, 0.05-level test. Calculations assume two-sided alpha level of 0.05 and 80% power.

7.2. Interim Analysis

We will conduct an interim analysis when we have enrolled 30 patients in order to determine if the detection rate for CTCs and DTCs is sufficiently high to continue to enroll participants. If the upper bound of the 95% confidence interval for the detection rate falls below the minimum threshold of 10%, we will attempt to restrict future enrollees using radiographic evidence of residual tumor at the completion of chemotherapy.

7.3. Final Analyses:

7.3.1 Statistical Analysis for Aim 1

In Aim 1, we will estimate and compare the CTC and DTC detection rates in several populations. We will compare detection rates in women with different RCB levels; the maximum CI widths in these groups are given above. We will also describe the detection rates in subgroups defined by receptor status, estimating the detection rates separately for hormone receptor positive patients, Her2+ patients, and triple negative patients. We will conduct exploratory logistic regression modeling to determine what additional factors may predict DTC/CTC detection. We will also consider changes in DTC/CTC detection over time using repeated measures logistic regression models.

7.3.2. Statistical Analysis for Aim 2.

In Aim 2, we will characterize the molecular and genetic features of the DTCs, CTCs, cells from the residual tumor, and cells from the primary tumor. We will estimate correlation coefficients for various features between cells of different types (e.g., DTCs and primary tumor) within the same patient.

7.3.3. Statistical Analysis for Aim 3

In Aim 3, we will estimate 3-year relapse-free survival in participants with and without detection of DTCs/CTCs at surgery. We will also explore Cox regression models using DTC/CTC detection as a predictor, first in a univariate model and then in multivariate models that include other known prognostic factors. We recognize that power is limited in this cohort to detect anything but quite large effects; these analyses are thus considered exploratory and will provide important preliminary estimates for future study design and hypothesis generation. We will further explore characterizing DTC/CTC detection as a time-varying covariate using values at follow-up assessments in addition to the status at the time of surgery.

CONFIDENTIAL

7.3.4. Statistical Analysis for Aim 4

In Aim 4, we will explore the value of DTC/CTC levels in predicting RCB class at the time of surgery. Initially we will use binary logistic regression to predict RCB 0/1 vs. RCB 2/3; if we find that CTC and DTC levels are useful, we will expand this model to an ordered logistic regression model and predicted membership in each class separately. Models will be assessed using the area under the ROC curve (AUC) and other measures of correct classification. These will be exploratory models that, if promising, will be validated in future independent data sets.

8.0 References

1 Bear HD, Anderson S, Smith RE, et al. Sequential preoperative or postoperative docetaxel added to preoperative doxorubicin plus cyclophosphamide for operable breast cancer:National Surgical Adjuvant Breast and Bowel Project Protocol B-27. J Clin Oncol 2006;24:2019-27.

2. Esserman L, Berry D, DeMichele A, et al. Pathologic Complete Response Predicts Recurrence-Free Survival More Effectively by Cancer Subset: Results from the I-SPY 1 TRIAL (CALGB 150007/150012; ACRIN 6657). Journal of Clinical Oncology 2012;In press.

3. Bast RC, Jr., Ravdin P, Hayes DF, et al. 2000 update of recommendations for the use of tumor markers in breast and colorectal cancer: clinical practice guidelines of the American Society of Clinical Oncology. J Clin Oncol 2001;19:1865-78.

4. Earle CC, Neville BA, Fletcher R. Mental health service utilization among long-term cancer survivors. Journal of cancer survivorship : research and practice 2007;1:156-60.

5. Waldrop DP, O'Connor TL, Trabold N. "Waiting for the other shoe to drop:" distress and coping during and after treatment for breast cancer. Journal of psychosocial oncology 2011;29:450-73.

6. Armes J, Crowe M, Colbourne L, et al. Patients' supportive care needs beyond the end of cancer treatment: a prospective, longitudinal survey. J Clin Oncol 2009;27:6172-9.

7. Kim MY, Oskarsson T, Acharyya S, et al. Tumor self-seeding by circulating cancer cells. Cell 2009;139:1315-26.

8. Chaffer CL, Weinberg RA. A perspective on cancer cell metastasis. Science 2011;331:1559-64.

9. Bonnomet A, Brysse A, Tachsidis A, et al. Epithelial-to-mesenchymal transitions and circulating tumor cells. J Mammary Gland Biol Neoplasia 2010;15:261-73.

10. Braun S, Naume B. Circulating and disseminated tumor cells. J Clin Oncol 2005;23:1623-6.

11. Giuliano AE, Hawes D, Ballman KV, et al. Association of occult metastases in sentinel lymph nodes and bone marrow with survival among women with early-stage invasive breast cancer. JAMA 2011;306:385-93.

12. Braun S, Vogl FD, Naume B, et al. A pooled analysis of bone marrow micrometastasis in breast cancer. N Engl J Med 2005;353:793-802.

13. Bidard FC, Vincent-Salomon A, Gomme S, et al. Disseminated tumor cells of breast cancer patients: a strong prognostic factor for distant and local relapse. Clin Cancer Res 2008;14:3306-11.

CONFIDENTIAL

14. Bidard FC, Kirova YM, Vincent-Salomon A, et al. Disseminated tumor cells and the risk of locoregional recurrence in nonmetastatic breast cancer. Ann Oncol 2009;20:1836-41.

15. Janni W, Rack B, Schindlbeck C, et al. The persistence of isolated tumor cells in bone marrow from patients with breast carcinoma predicts an increased risk for recurrence. Cancer 2005;103:884-91.

16. Janni W, Vogl FD, Wiedswang G, et al. Persistence of disseminated tumor cells in the bone marrow of breast cancer patients predicts increased risk for relapse--a European pooled analysis. Clin Cancer Res 2011;17:2967-76.

17. Braun S, Kentenich C, Janni W, et al. Lack of effect of adjuvant chemotherapy on the elimination of single dormant tumor cells in bone marrow of high-risk breast cancer patients. Journal of clinical oncology : official journal of the American Society of Clinical Oncology 2000;18:80-6.

18. Wiedswang G, Borgen E, Karesen R, et al. Isolated tumor cells in bone marrow three years after diagnosis in disease-free breast cancer patients predict unfavorable clinical outcome. Clin Cancer Res 2004;10:5342-8.

19. Cristofanilli M, Budd GT, Ellis MJ, et al. Circulating tumor cells, disease progression, and survival in metastatic breast cancer. N Engl J Med 2004;351:781-91.

20. Pierga JY, Bidard FC, Mathiot C, et al. Circulating tumor cell detection predicts early metastatic relapse after neoadjuvant chemotherapy in large operable and locally advanced breast cancer in a phase II randomized trial. Clin Cancer Res 2008;14:7004-10.

21. Krishnamurthy S, Cristofanilli M, Singh B, et al. Detection of minimal residual disease in blood and bone marrow in early stage breast cancer. Cancer 2010;116:3330-7.

22. Hayes DF, Cristofanilli M, Budd GT, et al. Circulating tumor cells at each follow-up time point during therapy of metastatic breast cancer patients predict progression-free and overall survival. Clin Cancer Res 2006;12:4218-24.

23. Pantel K, Brakenhoff RH, Brandt B. Detection, clinical relevance and specific biological properties of disseminating tumour cells. Nat Rev Cancer 2008;8:329-40.

24. Alix-Panabieres C, Schwarzenbach H, Pantel K. Circulating Tumor Cells and Circulating Tumor DNA. Annu Rev Med.

25. Bidard FC, Mathiot C, Degeorges A, et al. Clinical value of circulating endothelial cells and circulating tumor cells in metastatic breast cancer patients treated first line with bevacizumab and chemotherapy. Ann Oncol 2010;21:1765-71.

26. Muller V, Stahmann N, Riethdorf S, et al. Circulating tumor cells in breast cancer: correlation to bone marrow micrometastases, heterogeneous response to systemic therapy and low proliferative activity. Clin Cancer Res 2005;11:3678-85.

27. Rack B, Schindlbeck C, Andergassen U, et al. Prognostic relevence of circulating tumor cells in the peripheral blood of primary breast cancer patients. In: San Antonio Breast Cancer Symposium; 2010; San Antonio, TX: Breast Cancer Research; 2010.

28. Fehm T, Hoffmann O, Aktas B, et al. Detection and characterization of circulating tumor cells in

CONFIDENTIAL

blood of primary breast cancer patients by RT-PCR and comparison to status of bone marrow disseminated cells. Breast Cancer Res 2009;11:R59.

29. Riethdorf S, Muller V, Zhang L, et al. Detection and HER2 expression of circulating tumor cells: prospective monitoring in breast cancer patients treated in the neoadjuvant GeparQuattro trial. Clin Cancer Res 2010;16:2634-45.

30. Bidard FC, Mathiot C, Delaloge S, et al. Single circulating tumor cell detection and overall survival in nonmetastatic breast cancer. Ann Oncol 2010;21:729-33.

31. Slade MJ, Payne R, Riethdorf S, et al. Comparison of bone marrow, disseminated tumour cells and blood-circulating tumour cells in breast cancer patients after primary treatment. Br J Cancer 2009;100:160-6.

32. Rack B, Schindlbeck C, Andergassen U, et al. Prognostic relevance of circulating tumor cells in the peripheral blood of primary breast cancer patients. Cancer Res 2010;70(24 Suppl.):93s.

33. Rack B, Schindlbeck C, Jueckstock J, et al. Circulating tumor cells predict survival in early breast cancer patients. Journal of clinical oncology : official journal of the American Society of Clinical Oncology in revision.

34. Riethdorf S, Mueller V, Mauermann O, et al. Changes in circulating tumor and endothelial cells in peripheral blood of patients treated in the neoadjuvant chemotherapy plus targeted treatment breast cancer study "GeparQuinto". Cancer Res 2010;70(24 Suppl.):107s.

35. Mathiesen RR, Borgen E, Renolen A, et al. Persistence of disseminated tumor cells after neoadjuvant treatment for locally advanced breast cancer predicts poor survival. Breast Cancer Res 2012;14:R117.

36. Mathiesen RR, Fjelldal R, Liestol K, et al. High-resolution analyses of copy number changes in disseminated tumor cells of patients with breast cancer. Int J Cancer 2012;131:E405-15.

37. Amir E, Miller N, Geddie W, et al. Prospective Study Evaluating the Impact of Tissue Confirmation of Metastatic Disease in Patients With Breast Cancer. J Clin Oncol 2011.

38. Niikura N, Liu J, Hayashi N, et al. Loss of Human Epidermal Growth Factor Receptor 2 (HER2) Expression in Metastatic Sites of HER2-Overexpressing Primary Breast Tumors. J Clin Oncol 2011.

39. Poplawski AB, Jankowski M, Erickson SW, et al. Frequent genetic differences between matched primary and metastatic breast cancer provide an approach to identification of biomarkers for disease progression. European journal of human genetics : EJHG 2010;18:560-8.

40. Mego M, Gao H, Lee BN, et al. Prognostic Value of EMT-Circulating Tumor Cells in Metastatic Breast Cancer Patients Undergoing High-Dose Chemotherapy with Autologous Hematopoietic Stem Cell Transplantation. J Cancer 2012;3:369-80.

41. Yu M, Bardia A, Wittner BS, et al. Circulating breast tumor cells exhibit dynamic changes in epithelial and mesenchymal composition. Science 2013;339:580-4.

42. Mar-Aguilar F, Mendoza-Ramirez JA, Malagon-Santiago I, et al. Serum circulating microRNA profiling for identification of potential breast cancer biomarkers. Dis Markers 2013;34:163-9.

CONFIDENTIAL

43. Si H, Sun X, Chen Y, et al. Circulating microRNA-92a and microRNA-21 as novel minimally invasive biomarkers for primary breast cancer. J Cancer Res Clin Oncol 2013;139:223-9.

44. Sieuwerts AM, Mostert B, Bolt-de Vries J, et al. mRNA and microRNA expression profiles in circulating tumor cells and primary tumors of metastatic breast cancer patients. Clin Cancer Res 2011;17:3600-18.

45. Dawson SJ, Tsui DW, Murtaza M, et al. Analysis of circulating tumor DNA to monitor metastatic breast cancer. N Engl J Med 2013;368:1199-209.

46. Barker AD, Sigman CC, Kelloff GJ, Hylton NM, Berry DA, Esserman LJ. I-SPY 2: an adaptive breast cancer trial design in the setting of neoadjuvant chemotherapy. Clin Pharmacol Ther 2009;86:97-100.

47. Symmans WF, Peintinger F, Hatzis C, et al. Measurement of residual breast cancer burden to predict survival after neoadjuvant chemotherapy. J Clin Oncol 2007;25:4414-22.

48. Gleghorn JP, Pratt ED, Denning D, et al. Capture of circulating tumor cells from whole blood of prostate cancer patients using geometrically enhanced differential immunocapture (GEDI) and a prostate-specific antibody. Lab Chip 2010;10:27-9.

49. Magbanua MJ, Sosa EV, Roy R, et al. Genomic profiling of isolated circulating tumor cells from metastatic breast cancer patients. Cancer Res 2013;73:30-40.

CONFIDENTIAL
