# Supplementary material for: Neoadjuvant T-DM1/pertuzumab and paclitaxel/trastuzumab/pertuzumab for HER2+ breast cancer in the adaptively randomized I-SPY2 trial
Source: Nat Commun. 2021 Nov 5;12:6428. doi: 10.1038/s41467-021-26019-y (PMC8571284; doi:10.1038/s41467-021-26019-y)
Supplement: Supplementary file 3 — Description of Additional Supplementary Files [file 41467_2021_26019_MOESM3_ESM.docx]

Description of Additional Supplementary Files

Title: Supplementary Data 1

Description: Raw data for all outcomes except adverse events

Title: Supplementary Data 2

Description: Summary of all adverse events observed
