# Supplementary material for: Neoadjuvant T-DM1/pertuzumab and paclitaxel/trastuzumab/pertuzumab for HER2+ breast cancer in the adaptively randomized I-SPY2 trial
Source: Nat Commun. 2021 Nov 5;12:6428. doi: 10.1038/s41467-021-26019-y (PMC8571284; doi:10.1038/s41467-021-26019-y)
Supplement: Supplementary file 6 — Reporting Summary [file 41467_2021_26019_MOESM6_ESM.pdf]

nature research

Corresponding author(s): Amy S Clark

Last updated by author(s): July 21, 2021

Reporting Summary

Nature Research wishes to improve the reproducibility of the work that we publish. This form provides structure for consistency and transparency in reporting. For further information on Nature Research policies, see our Editorial Policies and the Editorial Policy Checklist.

Statistics

For all statistical analyses, confirm that the following items are present in the figure legend, table legend, main text, or Methods section.

n/a	Confirmed	
☐	☒	The exact sample size (n) for each experimental group/condition, given as a discrete number and unit of measurement
☐	☒	A statement on whether measurements were taken from distinct samples or whether the same sample was measured repeatedly
☐	☒	The statistical test(s) used AND whether they are one- or two-sided<br>*Only common tests should be described solely by name; describe more complex techniques in the Methods section.*
☐	☒	A description of all covariates tested
☐	☒	A description of any assumptions or corrections, such as tests of normality and adjustment for multiple comparisons
☐	☒	A full description of the statistical parameters including central tendency (e.g. means) or other basic estimates (e.g. regression coefficient) AND variation (e.g. standard deviation) or associated estimates of uncertainty (e.g. confidence intervals)
☐	☒	For null hypothesis testing, the test statistic (e.g. F, t, r) with confidence intervals, effect sizes, degrees of freedom and P value noted<br>*Give P values as exact values whenever suitable.*
☐	☒	For Bayesian analysis, information on the choice of priors and Markov chain Monte Carlo settings
☒	☐	For hierarchical and complex designs, identification of the appropriate level for tests and full reporting of outcomes
☒	☐	Estimates of effect sizes (e.g. Cohen's d, Pearson's r), indicating how they were calculated

Our web collection on statistics for biologists contains articles on many of the points above.

Software and code

Policy information about availability of computer code

Data collection	Clinical data was collected at each site using the Electronic Data Capture system for I SPY 2, and reviewed by central research coordinators for completeness and accuracy. Data review was under the auspices of QuantumLeap Healthcare Collaborative, the sponsor of I SPY 2. Gene expression microarray intensity values are derived from Agilent Feature Extraction files. DataPrint, Agendia's proprietary software for extracting data from Agilent Feature Extraction files, performs log2 transformation on the gMeanSignal. The 75th quantile is then subtracted from the entire array, and a fixed value of 9.5 added.
Data analysis	The randomization engine and Bayesian analytic software used in efficacy analysis are used under license from Berry Consultants, LLC; requests for code should be directed to don@berryconsultants.com. Biomarker data analysis was performed using R version 3.4.3 and Bioconductor; code available upon request from ispyadmin@ucsf.edu.

For manuscripts utilizing custom algorithms or software that are central to the research but not yet described in published literature, software must be made available to editors and reviewers. We strongly encourage code deposition in a community repository (e.g. GitHub). See the Nature Research guidelines for submitting code & software for further information.

Data

Policy information about availability of data

All manuscripts must include a data availability statement. This statement should provide the following information, where applicable:

- Accession codes, unique identifiers, or web links for publicly available datasets
- A list of figures that have associated raw data
- A description of any restrictions on data availability

Clinical datasets that support the consort diagram in figure 1 and the toxicity data in Table 3 and Supplemental Table 2 are available upon request by email to ispyadmin@ucsf.edu. All clinical and biomarker data supporting other figures and tables have been provided as a supplementary file. In addition, we are depositing

gene expression and base clinical variables in GEO under accession number XXX.

Field-specific reporting

Please select the one below that is the best fit for your research. If you are not sure, read the appropriate sections before making your selection.

☒ Life sciences ☐ Behavioural & social sciences ☐ Ecological, evolutionary & environmental sciences

For a reference copy of the document with all sections, see nature.com/documents/nr-reporting-summary-flat.pdf

Life sciences study design

All studies must disclose on these points even when the disclosure is negative.

Sample size	I-SPY2 is adaptively randomized, so number of participants in arm is not fixed. While accrual is ongoing, a statistical engine assesses the accumulating pathologic and MRI responses at weeks 3 and 12 and continuously reestimates the probabilities of an experimental arm being superior to the control in each HR/HER2/MP defined biomarker signature. The efficacy engine uses data from all control arm patients enrolled since the beginning of the trial. Experimental regimens have various sample sizes; and may exit the trial due to futility (predictive probably of phase 3 success < 10% for all signature), graduation (predictive probability of phase 3 success >= 85%), reaching maximal accrual (n=75) if predictive probability of phase 3 success between 10% and 85%, or as recommended by the I-SPY 2 DSMB (for safety).
Data exclusions	No data exclusions
Replication	No replication / Not applicable (Data generated from patient samples from clinical trial). Each patient can only be assigned to receive therapy on one experimental arm, and they either respond to treatment or not. Replicating microarray measurement is possible; but pre-treatment patient tumor sample is limited and precious; and therefore we have a single pre-treatment array for each patient.
Randomization	Biomarker assessments at screening are used to assess eligibility and classify patients into one of eight subtypes based on hormone receptor (HR), HER2-receptor and Mammaprint (high vs. ultra-high) status. The adaptive randomization engine preferentially assigns patients to agents based on continually updated Bayesian probabilities of pCR rates within predefined biomarker signatures; 20% of patients are randomized to the control arm.
Blinding	Investigators are not blinded to randomization results, but are blinded to efficacy data until announcement that experimental regimens have exited the trial. I SPY-2 is not a double-blind trial. Physicians and patients are aware of the allocated arm. Each agent has very specific safety and management considerations, so for safety and feasibility, the arm allocation is not blinded to the physician. However, no investigator is aware of how many patients are accrued to each arm, nor are they privy to any of the aggregate results until every patient on study has completed therapy and gone to surgery. Over the course of the trial, 16-20 sites were open to enrollment and up to 5 arms were being enrolled simultaneously. Investigators do not have any information about the status of the ongoing arms in the trial. Only the stats team and DSMB have access to the data and see the results of the adaptive randomization.

Reporting for specific materials, systems and methods

We require information from authors about some types of materials, experimental systems and methods used in many studies. Here, indicate whether each material, system or method listed is relevant to your study. If you are not sure if a list item applies to your research, read the appropriate section before selecting a response.

Materials & experimental systems

n/a	Involved in the study	
☒	☐	Antibodies
☒	☐	Eukaryotic cell lines
☒	☐	Palaeontology and archaeology
☒	☐	Animals and other organisms
☐	☒	Human research participants
☐	☒	Clinical data
☒	☐	Dual use research of concern

Methods

n/a	Involved in the study	
☒	☐	ChIP-seq
☒	☐	Flow cytometry
☒	☐	MRI-based neuroimaging

Human research participants

Policy information about studies involving human research participants

Population characteristics	The population from the I-SPY 2 study included patients 18 years or older, with HER2 positive invasive breast cancer histologically confirmed at diagnosis measuring at least 3 cm by clinical examination or imaging, with no evidence of distant metastatic disease. The I-SPY2 population had not been exposed to previous chemo- or radiation therapy. Between June 6, 2013 and August 17, 2015, 52 patients with HER2-positive tumors received TDM1/P, 45 received THP, while 31 contemporary control patients (derived from the start of the trial in March 2010 through deactivation of the TDM1/P and THP arms in February 2015) received TH (Figure 1). All patients received subsequent AC.
Recruitment	Recruited by individual investigators from local patient populations at their clinical site; Patients completed informed consent twice -- before baseline assessment and following randomization (assignment of arm/treatment). Biomarker assessments at screening are used to assess eligibility and classify patients into one of eight subtypes based on hormone receptor (HR), HER2-receptor and Mammaprint (high vs. ultra-high) status. The adaptive randomization engine preferentially assigns patients to agents based on continually updated Bayesian probabilities of pCR rates within predefined biomarker signatures; 20% of patients are randomized to the control arm.
Ethics oversight	I-SPY 2 complied with all relevant ethical regulations for work with human participants; and informed consent was obtained. The following sites recruited patients included in this study; and the protocol was approved by the site IRBs (see attached list)

Note that full information on the approval of the study protocol must also be provided in the manuscript.

Clinical data

Policy information about clinical studies

All manuscripts should comply with the ICMJEguidelines for publication of clinical research and a completedCONSORT checklist must be included with all submissions.

Clinical trial registration	NCT01042379
Study protocol	Trial protocol has been provided as a supplement with the manuscript submission
Data collection	The TH control arm was open for enrollment from 3/2010 to 1/2014; and the TDM1/P and THP arms are open to enrollment from 6/2013 to 8/2015. Clinical data was collected at each site using the Electronic Data Capture system for I SPY 2, and reviewed by central research coordinators for completeness and accuracy. Data review was under the auspices of QuantumLeap Healthcare Collaborative, the sponsor of I SPY 2. The I-SPY 2 sites that enrolled patients in the study where data was collected are provided (we have provided a spreadsheet that documents all sites and their associated IRBs.
Outcomes	The primary endpoint is pathological complete response (pCR) is assessed at the time of surgery and is defined as the absence of invasive tumor in breast and regional nodes (ypT0/is and ypN0). In the event a participant switches to a non-protocol assigned therapy, forgoes surgery, or withdraws from the trial, they are considered “non-pCR” during analysis. Secondary endpoints include residual cancer burden (RCB) and 3-year event-free survival (EFS). RCB is assessed using the RCB method (Symmans et al, JCO 2007) at the time of surgery to quantitate the extent of residual disease, where an RCB of 0 is a pCR. Patients are followed for long term outcomes (local recurrence, distant recurrence or death); and follow-up data is collected and updated yearly. EFS is calculated as the time between treatment consent and first local or distant recurrence or death; and patients without event were censored at time to last follow-up.
